# Supplementary material for: Perceptions of self-monitoring dietary intake according to a plate-based approach: A qualitative study
Source: PLoS One. 2023 Nov 28;18(11):e0294652. doi: 10.1371/journal.pone.0294652 (PMC10683993; doi:10.1371/journal.pone.0294652)
Supplement: S2 Appendix — (DOCX) [file pone.0294652.s002.docx]

# **Supplementary File 2- Focus Group Guide.**

### **Section 1: Perceptions on the 2019 CFG**

1. What do you/ *your clients* know about Canada Food Guide? What makes it easy/hard for your clients to eat in accordance to the plate-based approach that mirrors CFG?

### **Section 2: History of using dietary self-monitoring tools**

1. Which diet-tracking methods or applications have you ever used/ *suggested to your clients* (if any)? What makes it easy or hard for you/ *your clients* to use the tools you have experience with?
2. Do you know of any diet tracking tools that currently resembles the CFG or the plate-based approach? Which applications?

**[Showing a brief video of the prototype iCANPlate application]**

### **Section 3: Content and features of the proposed dietary self-monitoring application**

1. What did/didn’t you like about the plate-based dietary self-monitoring application app?
2. How do you view the application working to record all meals throughout the day? (Consider breakfast, lunch, supper and snacks)
3. What would be considered a successful day? What proportion indicates a balanced, healthy diet? How should improvements be defined for the purposes of the application?
4. Many “other foods” are not shown on the CFG. Which foods can you think of that your clients would find difficult to represent on the plate? How do you suggest they be tracked on the app? How could they be classified within the application?
5. How do you suggest beverages be tracked within the application? Should beverages be included on the plate? Should beverages be included in “other foods”? Should there be different classifications for beverages?
6. How do you suggest dairy (specifically liquid milk) products be tracked on the application?
7. What other eating behaviours or elements of the CFG should be included in this application (E.g., tracking mood, feelings, etc.)
8. Which instructions and support should be provided to the users to support and enhance your/ your clients use of the application?
9. What features can facilitate social support and enhance user adherence to the application?
10. Which features in the app could improve user’s confidence when tracking their food intake?
11. What features are required to ensure accessibility for all users?
12. Which other features of a dietary self-monitoring tool could be helpful in mirroring new CFG that we have not discussed yet?
