# Supplementary material for: Perceptions of self-monitoring dietary intake according to a plate-based approach: A qualitative study
Source: PLoS One. 2023 Nov 28;18(11):e0294652. doi: 10.1371/journal.pone.0294652 (PMC10683993; doi:10.1371/journal.pone.0294652)
Supplement: S3 Appendix — (DOCX) [file pone.0294652.s003.docx]

**Supplementary File 3- Components to Include in Future Iterations of iCANPlate^TM^**

## **Theme 3- Essential Component to Add in Future Iterations of iCANPlate**

### **Subtheme 6- Educational Content and Tutorials**

General public participants highlighted basic nutritional knowledge (i.e., categorizing different food groups) is needed to use the plate-based approach effectively. One member of the general public stated:

*“What about people who don’t have the education level required? There could be a section, something like a quick introduction to nutrients: This is what grains or proteins are, this is what they do, this is why it’s important to eat them.”* [General public -focus group 2- participant 4]

RDs discussed the possibility that the general public could misinterpret information in the 2019 CFG. They recommended adding guidance to this plate-based dietary self-monitoring application to enhance the end-user's comprehension of the CFG.

### **Subtheme 7- Report Dashboard**

Participants from both groups suggested this dietary self-monitoring application should automatically summarize inputted data over specific periods. They recommended the application provide a report dashboard of users' dietary intake to enhance their self-awareness of dietary behaviours, identify areas for improvement, and better understand their eating patterns. Additionally, RDs emphasized the importance of incorporating a report dashboard within iCANPlate that can be compared to dietary recommendations provided by the CFG. An RD mentioned the role of dietitians to review their client's summaries within iCANPlate and observe changes in their dietary patterns and said:

“*For example, here's what was changing their dietary pattern over these days of these weeks. We do it in the spring, summer and fall and see how different their dietary patterns are and how well they meet food guide recommendations.”* [RD- focus group 2- participant 1]

### **Subtheme 8- Accessibility**

All focus groups (general public and RDs) suggested key accessibility features for visual impairment and language to be incorporated into the dietary self-monitoring application. Haptic feedback (e.g., vibration), voice control, text-to-speech, high contrast mode, and colour blinding patterns were the suggested features to improve the accessibility of iCANPlate to individuals with visual impairments. One member of the general public said:

*“I know a lot of blind people use smartphones, which sounds very counterintuitive, but your phone can voice out whatever you press on your screen. So, it might be made compatible with that, the app.”* [General public -focus group 6- participant 1]

Considering diverse populations living in Canada, participants in all focus groups (general public and RDs) suggested this plate-based dietary self-monitoring application should support multiple languages, particularly official languages of French and English.

## **Theme 4- Optional components to add in Future Iterations of iCANPlate**

**Subtheme 9- Personalization**Participants in both general public and RD groups suggested personalization of the user's profile and goals is necessary. Particularly, they mentioned the profile and goal-setting pages could be customized based on age, gender, body measurements, activity level, culture, language, chronic conditions, and dietary preferences and restrictions. One member of the general public, an application developer, stressed the importance of providing customizable options for users and said:

*“The more you let people choose their desired features, you're looking at a happy user.”* [General public -focus group 9- participant 2]

RDs highlighted balance should be established between annoyance and usefulness, ensuring iCANPlate provides valuable information and feedback to the user without being excessively disruptive or intrusive. Providing this balance would differ depending on individuals’ personal preferences. Therefore, customization of notifications and some features of the application (such as optional components to add to iCANPlate) were also suggested.

**Subtheme 10- Automatic food logging**
Participants in both groups were particularly vocal about having alternatives to make data input more user-friendly in iCANPlate. Notably, using the mobile’s camera to scan their plate or a barcode was proposed to make the process less *“tedious*”. Some proposed having artificial intelligence built in the application to analyze what was present on their plate. A barcode feature could also facilitate usage of the application by making it quicker for packaged foods. Participants also suggested having the option to overlay the proportions on the application over what they are eating with the camera. This could then assist them in determining how to properly fill out the application.

*“There’s a lot of imaging technology that could be part of this new app where all you have to do is with your phone you just take a picture right? And then the picture can analyze what’s on your plate or on your bowl or whatever.”* [General public -focus group 3- participant 2]

**Subtheme 11- Recording other eating behaviours**When asked what other elements of eating behaviors, such as tracking mood while eating, should be included in iCANPlate, general public members stressed the need to keep the application as simple as possible, avoiding any additional features that would burden and discourage users. RDs, emphasized the importance of incorporating other eating behaviors into iCANPlate considering them as an integral component of the 2019 CFG. Topics raised included adding questions related to mindfulness in eating and intuitive eating principles (e.g., internal huger cues) into iCANPlate. RDs suggested adding a feature to ask questions about end-users’ feelings would potentially increase awareness on their eating behaviors:

“*So by including questions on their sense of fullness, how hungry they were, the satiety, like the clients really get into it and it motivates them to keep going and then they start to see patterns.”* [RD-focus group 2- participant 3]

Dietitians made it clear that in order to avoid making the application overwhelming these features should be optional, according to the end-users’ preferences.

**Subtheme 12- Social interaction**The general public and RD groups both expressed a desire for iCANPlate to include social interaction features. Specifically, some members of the general public suggested a feature to connect with friends and family, while others preferred the option to link the application to their existing social media platforms. Although some general public participants raised concerns about the discouraging effects of competition, many agreed sharing progress and dietary data could help them stay on track and could be included in the application as an optional feature.

*“So I think something that would help us be in a group so that we can – in a group that all of us have this challenge it would be really helpful for us. I don’t know, it would work for me a lot.”* [General public- focus group 6- participant 4]

The integration of social interaction features into the iCANPlate application was also voiced by RDs who suggested it would motivate individuals to actively engage with iCANPlate, compare their dietary habits with others, and potentially explore new eating styles and foods.

**Subtheme 13- Professional support**
The notion of connecting with professional support, such as dietitians or health coaches, was a recurring suggestion among the general public focus groups. They concurred real-time access to healthcare professionals may be more advantageous than artificial intelligence, but also noted the cost could be a hindrance for many end-users.

All the RD focus groups stressed the importance of offering a professional support option, allowing end-users to connect with RDs for more personalized advice and support. They suggested this connection could promote healthier dietary choices, prevent disordered eating, and improve overall health outcomes.

Furthermore, RDs suggested the inclusion of a support chat feature in iCANPlate application, where end-users could ask both an artificial intelligence and a real-time expert (i.e., an RD) for answers to frequently asked questions as well as for more in-depth queries. An RD said:

*“Probably having like, online live dietitian Q&A. I chat box just as that's what I am craving for when I – not about diet. But when I'm searching on other websites, and there are certain things I have no idea of and I really hope I can chat with expert on that topic, real time so that I can't get answers to my questions. That would be really luxurious.”* [RD- focus group 4- participant 1]

### **Subtheme 14- Interactivity**

Both members of the general public and RDs recommended the iCANPlate application should provide interactive features for users. In all focus groups, the need for analyzing the recorded dietary intakes to generate meaningful cues to action, reminders, and feedback on end-users’ eating behaviors was raised. Specifically, participants discussed the application should provide suggestions for foods in each food category, food alternatives, and recipes to help end-users make healthier choices.

Additionally, reminders were discussed to be helpful for end-users achieve their determined goals, such as reminders on when to use the application, or to drink water. For feedback, participants suggested analyzing eating behaviors using artificial intelligence. In this context, participants found positive and encouraging quotes such as "*Good job, you're on track today*" to be valuable in helping them achieve their goals while not evoking guilt.

*“I wonder if you could put in suggestions. Like, try and choose whole grains, or try and have mostly water as your fluid, things like that. To get people thinking about their habits, and prompt them because I know it's easy to forget these sorts of things.”* [RD-focus group 3- participant 2]

**Subtheme 15- Incentivization**
Several suggestions were made by both RD and general public participants regarding features such as gamification and material incentives and reward (e.g., monetary values) to encourage end-users to continue using iCANPlate and striving to meet their dietary goals. Participants discussed end-users could be incentivized upon completion of their set goals such as increasing the intake of specific nutrients (e.g., protein or vegetables). There were some discussions within the general public groups that real monetary values, and gamification would improve adherence to use of the iCANPlate. A general public member said:

*“I don’t know whether the app or whatever would be able to afford this but like make it that you win little emoji’s or whatever and you have to collect a certain amount to get into a draw to win something like a $50 gift card or something. I know that usually always works for me.”* [General public- focus group 4- participant 2]

However, many RD participants specifically suggested non-specific incentives (e.g., virtual rewards) and not the material incentives.
