# Supplementary material for: Perceptions of self-monitoring dietary intake according to a plate-based approach: A qualitative study
Source: PLoS One. 2023 Nov 28;18(11):e0294652. doi: 10.1371/journal.pone.0294652 (PMC10683993; doi:10.1371/journal.pone.0294652)
Supplement: S4 Appendix — (ZIP) [file pone.0294652.s004.zip › Anonymized GP Transcripts/iCANPlate-GP-focus-Group-7.docx]

**iCANPlate-GP-focus-Group-7**

[Start of recorded material]

Interviewer: Yeah perfect. This is the iCANPlate focus group with members of the general public on August 2 at 2pm eastern time. So this first part of this focus group we’ll be talking about behavioural techniques and self-monitoring applications. So my question for everyone, if you guys remember the Canadian food guide, actually I’ll get Meriam to show what the Canadian food guide looks like again onto your screen. But do you guys personally think it would be easy or hard to follow the Plate method seen in the new Canadian food guide?

Participant 1: To find what, sorry?

Interviewer: Do you think it would be easy or hard to follow the Plate method personally for you if you were to follow this? Do you think it would -?

Participant 1: Oh to follow, OK.

Interviewer: Yeah.

Participant 1: Yeah for me it would be easy, it's generally something we already know and it's a good sense which, for me it's nothing new it's something we hear from yours and it's just summed up in a book, your guide.

Interviewer: Perfect so do you think when you eat your meals it would be easy for you to just follow whatever this plate is showing you right now?

Participant 1: Yes.

Participant 2: Yeah.

Interviewer: Perfect.

Participant 2: Yes.

Interviewer: Does anyone else think it would be difficult?

Participant 3: Yes, well I think it would be difficult because I'm struggling most of the time, day to day where sometimes I don’t even have much money to eat, hardly anything. So it's very hard for me to follow the food guide because of all those, just problems in life you know what I mean. I have a disability and I'm on a fixed income and it's really hard when it comes to that.

Interviewer: Definitely yeah. I remember you mentioning earlier, your favorite food, I mean the food that you eat, you would eat the most everyday it would probably look more like just the wholegrain I guess side of the plate. So it would be the noodles or the rice like you mentioned before, correct?

Participant 3: Yeah.

Interviewer: Yeah for sure. And I also saw that Participant 4 wanted to say something as well.

Participant 4: Yeah I just wanted to say that it is at least for me it's not like exactly half fruits and veggies and quarter grain and a quarter protein it's like, I have a variety of foods across the entire day. So I guess kind of like quantifying that into over the entire day I had had fruit and veggies and a quarter grain and quarter protein would be difficult since I'm not eating exactly those proportions for every meal.

Interviewer: Yeah for sure. So it would be hard to calculate over the day what you actually eat for sure.

Participant 4: Yeah exactly.

Interviewer: Yeah but thank you Participant 4 and Rian for sharing your difficulties with following the food guide. Does anyone else want to add onto what they have said so far?

Participant 5: Does the food guide come with also a proportion like in terms of size not just in your plate because I sort of agree also with Participant 4 I think, I'm sorry I don’t remember the name but not every meal is structured this way then how do you know if you’re getting enough or not enough of what it is that you’re eating.

Interviewer: Yeah so this is definitely different from the 2007 version where it shows the actual proportions and sizes. Yeah this one does not specify any portions, it doesn’t focus on it as well it just shows like proportioning of what your meal would look like throughout the day. Yes Participant 6.

Participant 6: I think if I were to follow it the way it is I wouldn’t every day but I think overall the content then would be equal on say a weekly basis.

Interviewer: Yeah for sure. And I also think Participant 7 raised their hand at the same time that Ivan has raised his, so yes please go ahead.

Participant 7: Because me, since I'm a cook, what if, I actually don’t follow the proportionate because sometimes I mix the carbs with vegetables itself, certain things. So definitely I can follow with -

Interviewer: Yeah it would be hard to pick it out right once you’ve combined everything especially like I –

Participant 7: Yeah.

Interviewer: Yeah I know as well because with Filipino food especially it would be hard to pick out because everything’s just mixed into kind of like a stew probably.

Participant 7: Yeah.

Interviewer: Yeah was there someone else that wanted to say something, I think Participant 2 wanted to speak, or maybe it was just a sound on his end or her end.

Participant 2: Well I think you heard me talking to my dog.

Interviewer: Oh OK yes.

Participant 2: Sorry.

Interviewer: Yeah no that’s fine, say hello to your dog for me.

Participant 2: Thank you, hi Sky.

Interviewer: But yeah if there’s any other last minute questions before, I mean last minute answers before I go to the next question.

Participant 8: But for me, as in right now I'm just a student. So I'm so busy with my research. So if I spend so much time on cooking each portion of the food it will be time consuming, so actually it's very difficult for me to make, completely follow this guide because it's quite time consuming. If I spend so much time on cooking then I couldn’t graduate.

Interviewer: Yeah for sure, I know how you feel.

Participant 8: Yeah so it's kind of life’s value balance.

Interviewer: Definitely. But yeah so going off on that. If you were all to make changes to your diets, do you guys have any techniques or any tricks that would help you to change your diet in any way? And it doesn’t have to be necessarily for the Canadian food guide just in general if you guys have ever done a strict diet due to medical conditions or you wanted to change something in your diet because of for personal reasons, do you have any techniques that you have used before?

Participant 1: It's not a matter of technique, it's when you want to be on a diet.

Participant 5: I use many techniques, I've -

Interviewer: Oh sorry it's –

Participant 5: Commitment, its commitment. It's just a commitment to change or a choice you make every day of what you choose to eat and understand that what it is that you’re eating will directly affect, the way you feel, the way you look, the way you perform, the way you sleep. I think it's just a general decision that you make.

Interviewer: Yeah so you’re saying it's kind of like your mentality kind of right?

Participant 5: It's definitely mindsets yeah.

Interviewer: Yeah mindsets.

Participant 5: Yeah mindsets yeah.

Interviewer: Did Participant 7 want to say something?

Participant 7: Yeah, I think know the food that you’re eating because I guess there’s a lot of cheaper options for instance on vegetables that are affordable enough for you. So I guess it's just know the food that you’re eating.

Interviewer: Definitely.

Participant 1: I agree with we’re in charge the matter of willingness and commitment because it's easy to say, in practice it's not always easy because for example sugar is everywhere. It's very, when you are not diabetic it's very hard to avoid completely sugar. But you can take your coffee without sugar, you can take with half sugar, you can take, you can eat less fruity fruits for example, it's stands of this but the very severe diet or strict diet for me it's difficult but you can have techniques, you can have the word, commitment to avoid some things and to eat what you like.

Interviewer: Yeah definitely, it's definitely a good mindset to -

Participant 1: It's the taste, the taste is important in food. You will not eat something you don’t like, if you don’t like it when you are a little kid you, it's very hard to like it after, when you don’t like, you don’t like and generally you will try to eat what you like and that they balanced.

Interviewer: Yeah definitely for sure. Its one thing saying I’ll do it but doing it is just very difficult, especially if you have a lot of barriers especially time barriers or even monitory barriers as well. But yeah I would like to definitely hear from other people and what they think about changing your diet, if you ever tried to actually change it.

Participant 3: It's very hard for me to change my diet, and I know I might be going off topic here -

Interviewer: Oh no definitely it's fine.

Participant 3: When I was thinking about your application I didn’t really know what it was about but I think it would be cool if there was an application that helped you, it would give you different commitments of how into it you were and then it would help you make a diet, yeah I don’t know how to explain it. It's, I would need help to change my diet, I need somebody to council me kind of and if the App had that in it and that could kind of help you change your diet slowly I think that would be interesting or helpful.

Interviewer: Yeah definitely for sure, like kind of having someone to guide you.

Participant 3: I'm sorry if that sounded stupid, I'm just I don’t know.

Interviewer: No, no, no that was definitely the, a good suggestion. Some people actually have suggested that before in other focus groups so you’re not the only person who’s said that before.

Participant 3: OK.

Interviewer: Yes Participant 6.

Participant 6: I kind of eat all the things suggested by the Canada food guide. I can’t tell you every day it's proportionately correct.

Interviewer: Oh definitely.

Participant 6: I do include all of those over time, never been on a diet so I don’t know what I would do if I had to go on one. But so far so good.

Interviewer: Definitely for sure. I mean if it works for you then why do you need to change it. Yes Participant 1?

Participant 1: Yes yeah I saw the question on the chat.

Interviewer: Oh yes.

Participant 1: So you speak about rewards system, I'm thinking about some companies like weight watchers to lose weight and I had a friend in France which had the system of rewards when you have a diary and you put everything you eat, it's pointed and then you have a reward system. Personally I never tried it but it can be something encouraging to do that or group support. But it's a matter of, it's very personal I think you can do it by yourself.

Interviewer: Yeah we’re definitely going to go into the topic of using diet track Apps but yeah this question was more -

Participant 1: Oh this diet track, I already tried. But the problem is you do this in the beginning but after it's, it's like you stop one moment, you stop because you can’t do that for two or three months it's a lot of discipline and you don’t always have time for that.

Interviewer: For sure. Yeah did anyone have any else to add about techniques because this question is more of like a personal thing, like a reward system for yourself if you followed the diet a certain way you would reward yourself with maybe like a little treat or something. Yeah some of the things listed in the question in the chat were just some of the examples that people have used before to help them change their diets. So I was wondering if anyone, if it spoke to anyone in this discussion group? I guess no, not in particular.

Participant 1: Well for me I just avoided for example the all what is industrial food in the very, the dishes which are prepared in the supermarkets which are very salty and a lot of artificial things. I used to eat that for youth when I was student, in the beginning of work in life for a matter of time and also money but after when I heard all the harm it does now I'm not taking that or very, very few.

Interviewer: Definitely yeah. So you’ve, Participant 1 you’ve actually mentioned that you’ve used a diet track application before. Can I ask more about it, what the App was called and how, what, how you used it briefly?

Participant 1: No it's not an application it's like a board, you know a -

Interviewer: Board book yeah.

Participant 1: A board, in French we say tablot bog, it's like a common diary like you, on paper. Yes now it would be like an application but in the time I use it -

Interviewer: Yeah it's very time consuming for sure.

Participant 1: I'm not old, but in the year I used it maybe virtual applications will not be so developed as now and I used it like the old fashioned way of paper and a diary and a calendar of what you eat and the day etcetera. It's not a specific application but I know there are a lot of them now but I don’t know all the names but maybe it's easier today because everything is on phone or computer so it can be more -

Interviewer: More accessible.

Participant 1: Now you can put it on paper yes.

Interviewer: Yeah for sure. Did anyone -

Participant 7: May I say something?

Interviewer: Yeah for sure, go ahead.

Participant 7: May I ask a question?

Interviewer: Yeah.

Participant 7: Does your App includes – because one of my friend he’s working out and then he told me that he has this thing that he’s going to scan the food that he’s eating to monitor what’s in it and I don’t know if it's an accurate thing. So if he just take a photo of the food that he will be eating and I'm not sure if it's really working or I don’t know if it really tells you the exact calories his intake. I'm not really sure about that, how about your App?

Interviewer: Our App doesn’t have that function yet but a lot of people actually have suggested and we will be trying, we will be discussing it and considering that feature in our Apps since a lot of people have talked about that. But usually when people take pictures of their food it's more of an estimate, not exactly exact measurement of calories and portions. Especially if the picture is hard to decipher, especially if it's like an international food for example it will be hard to guess what types of foods are contained within the plate for sure. But yeah, has anyone used any type of diet tracking Apps like even from my fitness pound stuff. Oh Participant 4 yeah.

Participant 4: Yeah I've LNA just at my fitness spa. I used it very briefly and I used it mostly to track calories. So I was only inputting the higher calorie foods that I was eating. I wasn’t putting in fruits and vegetables that would just take too long so just rice and proteins and stuff like that. And yeah I thought it worked pretty good although I did get a bit lazy and I stopped using it, but yeah.

Interviewer: Yeah for sure, I guess pretty tedious over time.

Participant 4: Yeah exactly.

Interviewer: Yeah Participant 5.

Participant 5: I've used many, I've done the diet App so know my weight watchers and all that, I've done that in the past. For me the only thing that worked was not necessarily because I get fed up, like Participant 4 said it's, you get tired of using it obviously and you can’t be on a diet forever it just, it's unrealistic and it's just not good anyway. For me anyway in terms of change and choice because I've lost a lot of weight in the last couple of years, only came because I had a coach, a live coach someone who taught me what to do even to track, to learn how to track. I used fitness pal for two years now, I use it all the time, it's become a habit I think for me it's automatic.

I'm not currently on a live feed and choosing to be on a calorie deficit is to lose weight and then choosing the right food is because you want to be healthy. The word diet is overused and badly used in my opinion but [unintelligible 00:18:59] because not everybody and how to track it is important. So we could put it on [unintelligible 00:19:17] –

Participant 3: You guys are all breaking up real badly.

Interviewer: I think it's your internet Participant 3.

Participant 7: No I think Participant 5’s internet because –

Participant 6: Mine’s breaking up too.

Participant 7: Yeah I think Participant 5, Participant 5 does not have a stable internet connection so yeah.

Participant 5: I'm on data, do you hear me better?

Interviewer: Yeah.

Participant 7: Yeah it's better now.

Interviewer: Thank you, yeah sorry for making you use your data.

Participant 5: No, no, no it's fine, it's fine as long as you hear me well. Yeah so that’s my opinion. I think it's good to have coaching for that and you’ve got to find coaching but coaching’s important.

Participant 8: Yeah for me, like during the Covid 19 I self-isolation I gained a lot of weight, almost like a time cheap hands. So after Covid 19 I just went to a gym and get a coach and her just to help me to do some, like work hard and help to monitor my daily food, what kind of food I eat every day but I found that I, because this not a sustainable way because having a coach is very expensive. So we can just afford like, for me it was three months or four months. But before the application I used to want but it's in Chinese, it can do if you input your weight and your weight inside of that application then it will calculate how much calories you need in your whole day.

And then they will give some recipes for you to say, you allowed to what the kind of food you can eat and it's a kind of like say AI nutrition to help you but for me I'm also very lazy maybe I can like the follow age of four two weeks or one week then I don’t know, that keep take as I say oh my god life is so struggling I need to eat more, I need to eat more cake and sugars.

Interviewer: Yeah so good sugar.

Participant 8: Yeah so this kind of or partial pressure eating, yeah.

Participant 5: There’s age too that factors into this because I remember being 20 years old and I pretty much could eat anything and felt great the next day. But I'm 44 now and if I eat late at night or I eat badly for a few days I will feel it afterwards. So my motivation is not necessarily only physical, the way I look it's a lot about the way I feel also which you don’t get in your 20’s. Well not the same way anyway yeah.

Interviewer: Yeah has anyone had any other Apps that they wanted to mention? Yes Mil.

Participant 7: By the way –

Interviewer: Oh yeah.

Participant 7: I saw this online it's, if I'm not mistaken it's called Pink Dragon Studio and she said she mentioned there in the video that each one has different metabolic vibes based on for instance having a diet so they have to know which metabolic types they have in order to work on it in certain areas. And I'm not really sure if – do you have that kind of thing in your application whereas we know what metabolic type we have?

Interviewer: That’s actually a really good idea, unfortunately our App is like a very, very simplistic so I know that we will definitely show you the prototype in just a bit, we just wanted to gather some information about different Apps and how it worked for your guys and if you liked using the App as well. So this could give us a good idea on what to work on for ours for sure. But yeah thank you for bringing that up that is such a good idea. Now on the topic of diet applications or self-monitoring tools in general. Do you guys, have you guys seen any that look like the plate in the Canadian food guide? Do they, do any of those monitoring tools look like a Plate? Nope?

Participant 5: The one that’s the most similar is Noom. I don’t know if you’ve seen Noom, yeah Noom has a lot of very, very simple ways to calculate your portions. Not necessarily a plate but it's a lot like fist size, palm size, finger size right so very factile ways of calculating your portions. My Fitness Pal is probably the least, it works a lot with macros and calorie intake. So but I’d say user friendly would be Noom. And Participant 7 mentioned before about the taking a picture and getting all the information, Noom and Weight Watchers and I think my Fitness Pal too started doing this, you have to scan the barcode on the food.

So instead of going into, looking at the picture, what they do is they make you scan the barcode and then it will defile proportion how many points for example for Weight Watchers because Weight Watchers is a point system. And then everything else will go with calorie intake and proportion right. How many carbs, how much fat, how much protein all that jazz. So if you can scan the barcode then that works very, very well and it's done pretty quick, yeah.

Interviewer: For sure yeah. So on that note since we’ve seen the App, how do you guys, if you guys were to use this App how would you see the application working so that you could record all your meals throughout the day?

Participant 5: What is this, this is what I'm eating or what it tells me to eat?

Interviewer: Yeah.

Participant 5: What I'm eating.

Interviewer: What you’re eating yeah. So if you were to track it yourself starting from like a blank plate how would you see yourself recording your meals using that App?

Participant 3: I think it would day at least a day or so of trial before you would really know if, how –

Participant 7: May I ask a question?

Interviewer: Oh yeah sure.

Participant 7: Since I'm tracking my meal, is there a button that I can drag down, for instance I eat more carbs so is there a button that I can maximize the ratio of it?

Interviewer: Yeah so I don’t remember if you saw in the mock-up for this, the little small circles on the bottom, it's kind of like a legend of the colours. You can actually use those colours to increase the sizes of that certain proportion so if you wanted to increase your grains you could just use the little button on the bottom to increase the amount of grains in the plate, like maximizing it yeah.

Participant 7: So basically your App is just like monitoring your food intake?

Interviewer: Yeah it's just recording it yeah.

Participant 7: OK.

Interviewer: And based on the three food groups on the food guide.

Participant 1: So it is, if you are following all of the category food kind. With each food you eat it recalls you, it tells you, you are on the goals or you are not in the goals?

Interviewer: So you can, once you fill your plate up you can easily compare it to the food guide which is pretty much, yeah.

Participant 1: Oh, OK. OK so you compare to what you are, compare to food guide.

Interviewer: Pretty much yeah.

Participant 5: And there’s no calculation of anything other than the visual approach of it.

Interviewer: Yeah, at the moment the Canadian food guide and the App don’t focus on calorie intake which is a very surprising to most people because we’re so used to taking count of all the calories throughout the day or even the portions.

Participant 5: What happens if in my grain portion I'm having, I don’t know a piece of crusty bread or I'm eating a cookie? I fail to see the, for me anyway, how would it differentiate from a bowl of Quinoa as a whole grain and a cookie that in the end it's also grain and I understand it's my choice of what I eat but I think that if I'm going to use an App it's going to be for some kind of accountability to myself but accountability none the less.

Interviewer: Yes.

Participant 5: If it doesn’t differentiate from grain, also I'm a protein eater, I'm not necessarily plant base only I do, the glumes and all that too I'm good with that but I’ll have red meat, chicken I’ll have all that as well so the food guide or that App can’t tell me you had too much animal protein or you should eat less whatever it is.

Interviewer: Definitely yeah, it's hard to differentiate just what we have so far yeah.

Participant 1: So far so it would be maybe –

Interviewer: So far yes, sorry we have a lot of work to do.

Participant 5: But I think it's great that you’re doing this. I think that right now everyone is going on their electronics, it's not more like Participant 1 said that it's written down and all that jazz, they don’t do that anymore. But I think there should be some quantifying something.

Interviewer: Like GTOS yeah,

Participant 5: I don’t know what it is, yes, yes to make it a bit more –

Interviewer: Yes Participant 4?

Participant 1: But only to compare that –

Interviewer: Oh sorry Participant 1.

Participant 1: Yes we have to, in the future the habiliment of calculation and of quantified goals and targets. And not a lot but a minimum because there are BP applications that are too complicated because it's full of figures and graphs and no, not that. But if it's also very basic and simple it will not meet all the needs, should be between.

Interviewer: Yeah it needs a balance because it could be either overwhelming or underwhelming.

Participant 1: Yes exactly.

Interviewer: Yeah. Yeah Participant 4, sorry.

Participant 4: Yeah I agree with Participant 1 on that and that I like the simplicity of it that you don’t really have to calculate anything it's just looking at your food and, oh I'm eating this much of that. Because with a lot of diet applications you know calculating I'm eating a cup of rice or half a cup of rice, or this much meat, a 100g of meat or whatever it gets pretty mundane and tiring really fast. So I agree with Participant 1 that a simpler way is better but also some detail would also be nice but I get that that’s also really hard to do. Another difficulty I think is that when you eat food that’s all mixed together, a stir fry right, I guess it's hard to visualize what proportion of each there is right.

Interviewer: Yeah unless you cooked it yourself yeah definitely.

Participant 4: Exactly, you could be way off the mark in what you put on the application right.

Participant 5: Portion distortion. It's an actual thing, portion distortion is an actual thing.

Interviewer: Yeah so what I'm getting so far is that people like the simplicity because it's very visual but in the end there are some details to put into it for sure yes. How about Participant 6 or Participant 8 do you have anything to add?

Participant 6: Yeah I'm still wondering what the ultimate objective is with this App because it does things like wrong and OK so I eat so much protein and it hasn’t been clear on how much to set on the plate right. Some of this that and the other.

Interviewer: Yeah so if Meriam wanted to add onto what I have to say, so generally the goal of this App for the general public is just to be able to track all their food throughout the day and see, I guess when you finish your meal just to see the general look of your plate and see if it compares to the Canadian food guide. It's like a very general goal at the moment.

Participant 8: OK. For me as a, maybe it's too like kind of simple maybe you have more work to work on that. As in maybe you can add some rewarding system inside it to like a new plan system for one week then the system will give you one bunch or like a rewarding system to make it more interesting.

Interviewer: Yeah definitely. I would definitely like to hear from Participant 3 since you seem to be very excited about seeing the App especially from, maybe for Morgan too if you wanted to chime in?

Participant 3: I'm listening I'm not just trying to waste your time.

Interviewer: Oh no, no, no.

Participant 3: I have some learning disabilities and I'm just, sometimes when I try and give my thoughts out and try to explain what I'm thinking it's very hard for me do.

Interviewer: Oh no that’s fine, definitely we would love to hear your thought process though.

Participant 3: OK. I’ll try to put it into words and then I’ll come back in a second and I’ll let you know OK.

Interviewer: I’ll give you some time yes.

Participant 3: I'm really sorry, I hope I – I hope you don’t feel like [unintelligible 00:34:36].

Interviewer: Oh no, no, no. Oh no that’s fine, definitely just take your time and even just –

Participant 3: [unintelligible 00:34:45] or nothing like that so.

Interviewer: Participant 2 do you have anything so say?

Participant 2: I think my problem is the quantity.

Interviewer: Definitely.

Participant 2: I tend to have bigger portions that what the Canada food guide says.

Interviewer: Yeah for sure.

Participant 2: And I've got to get rid of my belly.

Participant 3: OK. I’ll say something, a lot of times when I'm trying to eat a meal and that kind of balance out I will create the one portion of the meal and I have a hard time of eating slow, I eat very fast and then I’ll just stuff myself before I get to the other helping, other portion of food and then half an hour after I'm done I’ll be starving again, after I'm done eating.

Interviewer: Oh yeah I definitely can see that yeah.

Participant 3: Then it's really hard for me.

Interviewer: Yeah.

Participant 3: And then sometimes I’ll crave sugar or something or chocolate for a month and then for another month I won’t crave it at all. My diets all over the place I don’t really have a diet that’s why I wanted to join your group because I thought maybe the App would be something like to help me get into a diet or whatever that’s why, but yeah.

Interviewer: Yeah unfortunately it's not developed at the moment but when it is developed in the future if you would like we could definitely contact you and then you can help pilot the study for sure, pilot the App for one of the studies.

Participant 3: Yeah like I said, I think one of the best things for the App and not just for me for people, it would be like if there was different levels of motivation that you were into doing your diet and then you could say where you were and the App would help you, well I'm not in charge there, it could help you probably be motivated. You were trying to direct you and so it wasn’t personally too hard and whatever and help you figure out how your diet – and that would be really cool.

Interviewer: Oh like helping you reach your personal goals.

Participant 3: Yeah on, but then that would be cool.

Interviewer: Yeah that would be cool, thank you so much for sharing. Yes Participant 1 did you want to say something?

Participant 1: No I just had a question for you please?

Interviewer: Yeah, yeah go ahead.

Participant 1: Is particularly because I saw one version once but, is the Canada food guide only the general view of what should a plate be or it is full of some details because the applications follows the Canadian food guide? We saved more details in the future. Is the Canadian food guide only the picture of the plate and the team send advices or it's something as we didn’t see in the beginning? So to because to build this application following the Canadian food guide we should see if the Canadian food guide only gives you, you should take half this, quarter of this, quarter of this or if it goes more deeply to say, OK in this category it's going to be that and that etcetera.

Interviewer: Yeah so –

Participant 1: That’s the detail.

Interviewer: That’s a really good question. So when we showed you the two sided poster, that is the Canadian food guide although if you go onto their website there are actually a few resources and details that are added to the food guide like recipes and stuff –

Participant 1: Oh OK.

Interviewer: For people to use, yeah. And also to add onto that the App is going to be based on the food guide but using these focus groups they’re thinking of kind of like expanding on it and adding other details based on what the general public say and also arranged dieticians.

Participant 1: Yes because it's good this update, will have it not only to start your meal but before you purchase the ingredients for your meal at the supermarket to already see what you buy. You know we always see some people scanning some, as we say some items and articles in the supermarket, I don’t know if it's for marketing or for health. The application should be before the, before what you eat and when you prepare your meal so it must be global, not only all the process from purchasing to eating.

Interviewer: Oh yeah for sure, from getting it and then cooking it and eating it yeah.

Participant 1: Yes.

Interviewer: That makes sense yeah, thank you for that. And then so in, I guess pertaining to I guess adding your meals to the plate we were wondering what kind of references would you use to help you represent the different meal portioning on the plate if you were to record your meals?

Participant 1: How would you present?

Participant 5: What was your question exactly, like which way would help us the most?

Interviewer: Yeah so I remember you mentioned in Noom I think they use reference of purchase like fist size, or finger size or something like that so should we, do you think we should do something like that or use standard measurements but just like a cup that’s 250ml exactly, or a bowl that contains this much of liquid or solid, something like that?

Participant 5: I mean I'm not a type A or anything so I'm not very, I don’t really care about specifics but I think that if you’re out and I think that’s where we need the most help is not necessarily when you’re home and you could actually calculate your stuff but when you’re out you should be able to estimate very quickly what you’re eating. Maybe it won’t be exactly precise but there’s a difference between a whole hand and a palm of a hand right. So I definitely enjoyed that way of measuring way more than I have of late with all the macros and all that where I have to weight my foods.

And I didn’t do that for long I just, I did it for six months just to get into learning about portion size and all that and unfortunately when you’re in that phase and you do have to lose weight and you do have to the work because you’re sick or for whatever reason it's important to understand that you need the tools at the time, it's not a forever thing but it's a sometimes thing and it's for a little while thing. But in general if you’re able to have a quick reference I think that can help everybody, in my opinion.

Interviewer: OK thank you. Participant 1 had his hand up.

Participant 1: Oh sorry, no, no I forgot to – but yeah it could make sense here because maybe it's more useful outside the house than inside when you go to restaurants or you are on trip or you, I don’t know you eat – we forgot that we have the Covid but after one year in the house, no friends, no family or being in the restaurants. So this is the places where it will be useful because to give you the, yes so for me for the measurement of the measure of the portion it should be something standard. Like I don’t know, half a dish or not with fingers because you cannot, it should be easy to compare between maybe references. Not it refers a way of measure, it would be too complicated.

Interviewer: Yeah, oh Participant 4 do you want to –

Participant 4: Yeah can I go next yeah. I definitely agree with Participant 5 and Participant 1 that a more quality of method would be a lot better. Especially since the plate itself is pretty qualitative, I mean it's not like very specific so I think if the reference is like, are as specific, just as an add up it doesn’t really make sense. So I definitely yeah, I think qualitative is better but also it might be a matter of preference. So each person might have a different preference of how they want to measure their foods, I guess if you’re a chef you might be a lot more comfortable using grams and cups and stuff like that.

So I think maybe having a feature where you could change in between the measurement I guess like units. Kind of like how some Apps changes kilometres and miles or imperial and metric, they change between grams and kilograms, I don’t know, the size of a hand or the finger whatever yeah.

Interviewer: Yeah or even the palms too yeah

Participant 4: Exactly yeah.

Participant 1: I completely agree, I prefer the, yes the half cup or half dish that it's graphs or external graphs or A, because it's not the same representation for one people to another people, it should be something you can figure out easily. Not a figure where you cannot – for me 250g for another person it's another illustration it's not the same.

Interviewer: For sure. But yeah in the interest of time I'm just going to ask everyone in general who haven’t added anything yet, do you guys have any suggestions that you wanted to voice out about references? Oh yeah Participant 8.

Participant 2: Yeah I give -

Interviewer: Oh yeah Participant 2 yeah.

Participant 2: I think I like the idea of proportions by the hand, by the palm, by the fist full, by the finger because who has, who’s going to measure their food honestly.

Interviewer: Every single time yeah. Yeah and then Participant 8 I think.

Participant 8: Yes actually –

Participant 2: Wait for me –

Participant 8: OK Participant 2 go ahead.

Participant 3: I was going to say I know, it's really hard for me follow a diet but I've been in a mental hospital before right and I've been in places like that and when they feed me it's always, it's very by the book right. It's very how it's supposed to be and I don’t mind it, I actually enjoy it you know what I mean. I just don’t have the knowledge or anything to do it myself but it's not that I don’t like eating like that, you know what I mean. If I could do it then I would it's just I just find it difficult.

Interviewer: Yeah, definitely for sure and it's very time consuming too. Yeah sorry Participant 8 yeah, Participant 8 wanted to go.

Participant 8: OK, finally. Actually for me, I react, agree with Participant 5 that’s also how my gym coach told me, like you can use your hand to measure the size of your food. But the rest I get some hints from a YouTuber and he now got told me if he and I get chance together maybe we can weigh the food on, or before eating. And is any, you can get a kind of a sense, actually I tried this way from last week actually it's very good you can weigh your food, as is there, for the balance and then you can know how much you eat every meal. I think besides taking that it also can give you a sense or like, you can’t feel like you can’t control your life. It's the, you can’t bring like a huge sense of achievement for you so I really like this one.

Interviewer: Yeah definitely.

Participant 5: I like that, the sense of achievement is what makes you want to do it again the next day. I love that yeah.

Interviewer: And Participant 6 you wanted to add anything?

Participant 6: No I just wanted to ask if at some point regardless what form of measurement you use, is there a feedback. Well you know you’re a little short on protein, you should up the veggies a little more. Are you looking at something like that in your App?

Interviewer: Yeah since a lot of people have been asking for that we are definitely going to look into that for the App for sure.

Participant 6: Thank you.

Interviewer: Yeah thank you for the suggestion. And as well I guess we’ve mentioned things like cookies before, we were wondering there are many other foods besides cookies that are not shown on the Canadian food guide. So would those types of foods, what do you think you would, which kinds of foods do you think would, you would find difficult besides cookies to retract in the Canadian food guide?

Participant 7: Ice cream.

Interviewer: Ice cream yeah, ice cream.

Participant 7: I don’t know I mean anything that isn’t perfect food. It's very, it's really, it's very interesting that I'm in this thing because I find it absolutely unrealistic to ask a human in this world right now that we live in to not ever eat anything that is not perfectly health for you.

Participant 1: Oh yes.

Participant 7: OK to allow for fun foods. I have twins, they’re 11 years old and I pretty much helped myself raise them with Sesame Street OK. And I remember John Legend having a Sesame Street song with cookie monster and he called it the sometimes food. And I absolutely love that idea, my children are 11 and to this day they still tell me is it the day for the sometimes food. And I absolutely love that and that should definitely be there because it's OK so sometimes scoop on the tangent and make it OK because a lot of us, women in particular, I'm going to put myself in there because I've read and learned and listened to podcasts and podcasts and all that. And we’re very hard on ourselves because we decide to have a bit of ice cream. That’s not right, it should be OK sometimes.

Interviewer: Yeah and it's good to enjoy definitely, cookies feel good.

Participant 7: Yes that’s right, it does, it does. And I lost weight and I eat ice cream. So it's OK to sometimes eat ice cream and you could still feel good and still feel strong and still feel healthy even if you eat sometimes food.

Participant 1: Yes of course.

Interviewer: I love that.

Participant 1: Yes and thinking about, because even the, even fast food, we see now in the McDonald's store, Thornton’s store everything, they I don’t know for how many years, they put nutritional information on all their food, all their menu in the world and so, and it's junk food or fast food, not, it's bad food or sometimes food. And maybe this application, because it's not only the healthy food we do at home, but in the fast food you can find something healthy, it's not all unhealthy. So it could be relevant to follow this example of the fast food giving nutritional information about their food in terms of calorie or in terms of other characteristics, not only calories.

Interviewer: Yeah definitely. Participant 2?

Participant 2: No sorry that was my dog again.

Interviewer: Oh that’s OK, all good.

Participant 2: Sorry.

Interviewer: Yeah and it's like, it's just a lot of –

Participant 3: I just wanted to interrupt one more time.

Interviewer: Yeah, no problem.

Participant 3: And say I know I didn’t speak too much but I really enjoyed being part of your group and I'm sorry if I didn’t give more input or anything like that.

Interviewer: Oh no that’s fine, and it's great for you to listen as well just so you can hear other people’s experiences.

Participant 3: But one thing for sure, keep my name on the list for when the App comes out, I’d love to test it out.

Interviewer: I, we’d love to have you test it out yeah for sure I’ll keep you on the list anyway so just so I can remember.

Participant 4: Can I say something?

Interviewer: Yeah.

Participant 4: I think foods generally foods like cookies like you mentioned or ice cream or like Coca-Cola and stuff like that, I think they definitely pack a bigger punch than what they would like. A cup of soda is pretty small but it has like 40 grams of sugar, so I think just using size to kind of proportion plates, you might run into issues with the foods like that that are really high in sugar or really high in calories and stuff like that.

Interviewer: Definitely.

Participant 4: And that might be a bit more difficult to track I guess. I don’t really have a solution for that but yeah.

Interviewer: Yeah but definitely the same and as well there’s more than just like deserts and sweets that are not part of the other foods that are not found on the Canadian food guide whereas foods like, even Kimchee or international foods it's going to be very hard to track those. Do you guys think it would be good to have another category I guess outside of the plate, like other foods and the sometimes foods and like –

Participant 5: Yes the diet App has that. If you go into the bio dats and you enter Rumen it's in there. It's in there to calculate, general cal whatever. The foods you choose to eat are in there right and you pick what it is you want to eat. It's also in my opinion your demographic that will actually be using this App, there is someone who is already somewhat conscious of what they’re eating to begin with because somebody who doesn’t necessarily have that mindset or care at the moment, because we don’t always care what we eat I mean I've eaten big macs and cheese burgers and all that when I was young for sure and I didn’t care right.

So it is what it is, but so you’re already reaching a demographic that has some kind of consciousness of what needs to be eaten and so make it maybe more obtainable in terms of, like you said including the foods that are not on that little plate with the perfect squares.

Interviewer: Yeah definitely.

Participant 1: Or the perfect foods.

Interviewer: I love that, I love that you call it the perfect food. But yeah so anyone else want to add onto that about other foods?

Participant 8: What kind of desert, like cake, cheesecake somehow?

Interviewer: You guys are making me hungry.

Participant 8: Yeah because of all people like me, I'm absolutely a sweetie tooth and I couldn’t live without desert. So I needed the food guide to tell me how much desert I should eat and like reason why I weight it. You know weights out in decreasing so much my health.

Interviewer: Yeah definitely we always have those questions, how much is too much.

Participant 8: Because we don’t want to be a perfect human being we just want to live healthy, a little bit is as before so it's acceptable for us to eat some unhealthy food.

Interviewer: Definitely yeah because I am, yeah. And as well, on the topic of Coca-Cola and stuff, how do you suggest beverages should be tracked with this, these are pretty difficult questions but yeah how do you guys think we should track those type of beverages? Like coffee, coke, eateries like all those different things?

Participant 1: For me, I see – OK go, go.

Participant 8: Oh no, no, no I say like a bowl bachy, like the [unintelligible 00:57:28].

Interviewer: Oh yeah the [unintelligible 00:57:29] so good. Yeah Participant 1 go.

Participant 8: Participant 1.

Participant 1: For me I would not put the beverages in this application. No because I would restrict it to food because beverages it's easier than food. I mean you know that sodas it's full of sugar, you know that coffee it's full of that, it will just make the application more, how can I say, loud, more too much information. I wear a 52 foot but it's a personal opinion, if I do for beverages I put another application for that. But it's less useful than for food. Beverages generally we know that sodas is not good, juice is full of this and it's not as relevant as for food. Food is more complicated to track what is good, not good and the nutritional content than for beverages.

Interviewer: Yeah.

Participant 5: Also three quarter of the planet don’t drink enough water so it should just be kept to water.

Participant 1: Beverages, the Canadian food guide the safe thing here its water, it's not the others so it's not very interesting to add it in this application for me.

Interviewer: Yeah Participant 7 actually has her hand up and I was wondering if you wanted to say something?

Participant 7: Yeah Participant 1 is right, it would be hard to add the beverages because you still have to work on more about the food application itself so why not just focus more on that because just like for instance coffee we don’t know how the people put more sugar on it so it's so hard to track.

Participant 1: Exactly.

Interviewer: Yeah and as Participant 6 also pointed out in the comment section, all the other beverages besides water have actually been, not mentioned in the food guide. So yeah I feel like all of you have pretty good points about just keeping it to water. Is there anyone else who objects to actually - anyone who actually wants to include beverages in this app?

Participant 8: I think black coffee and a tea it's is a healthy, right. So if you didn't put any sugar inside it, it's super healthy. Even the black coffee can help you to increase your [unintelligible 01:00:24] metabolism handle.

Interviewer: Did Participant 2 – oh, sorry.

Participant 4: I'm just going to make a quick one. What Participant 5 said earlier that -

Participant 5: Yeah, I'm here.

Interviewer: All right. Sounds good.

Participant 4: Just to add onto what Participant 5 said earlier about how people who get this app are already mindful of what they eat, and they're looking to attracted diet. It's unlikely that they drink like a Coca Cola every day. They're more mindful of what they drink. And as Participant 1 said, keeping track of what you drink is a lot easier than what you eat, because it's more clear.

Interviewer: Definitely. Yeah, for sure. Participant 2, since you're still here, I was wondering if you wanted to add anything.

Participant 2: Yes I can. Can you hear me?

Interviewer: Yes, I can hear you.

Participant 2: Can you hear me?

Interviewer: Yes.

Participant 2: Hello?

Interviewer: Yes.

Participant 2: Hello.

Interviewer: Hello?

Participant 2: Hello?

Interviewer: Oh, no I can hear you. I think the beverages should be included, because that's part of your food intake. If you're having a milkshake with your dinner, you need to include that in your intake. I think beverages should be included.

Interviewer: That's a great point. Yeah, wow. Thank you for that. Yeah, so on the topic of milk, I don't know if you guys realised or like saw, but milk is actually not included in the food guide this time. So how do you guys think that should be classified? Should that be classified as a beverage, or even a protein or something?

Participant 5: Is there cheese, or is it all dairy or just milk?

Interviewer: I think, well, yoghurt is on there. But I did not see cheese on it.

Participant 5: Because plain yoghurt is considered a protein. It's not considered a dairy product, because of the bacteria in it. But the dairy, it's yeah, there's no cheese. I noticed.

Interviewer: Yeah, there was no cheese. I noticed it too. I love my cheeses.

Participant 5: Is it realistic? I mean, I don't know. A grilled cheese sandwich, who didn't grow up on a grilled cheese sandwich? That's the fear in that is that the unrealistic approach to it won't make the app user friendly. There has to be something that the regular folk could just relate to. It’s important.

Interviewer: That’s true. Yeah, because we don't want people to be afraid to use the app and feel like, oh, I shouldn't be eating this at all. But then make them feel anxiety about eating their food.

Participant 2: Yes.

Participant 5: Exactly.

Interviewer: Yeah, definitely. I think it would be a good idea to add dairy. But do you think it should be in a food group or as a beverage? Or maybe just depends on the type of dairy, I guess?

Participant 5: I mean, if you look at many diets, restrictions, dairy is not often included, it's a fat. And based on what the Canadian Food Guide, I think, has always been, they haven't taken into consideration the actual macro of the food, but more so the type of food it is and what category it's in. So I think that's why dairy is rarely included in certain diets. It's very high in fat, and only certain cheeses could be considered somewhat protein, it's mostly fat. But yeah, so it would be very difficult to include that in there. But I think because you have an app, there should be an allowance for that also somewhere.

So the dairy portion of it is not considered on the Canadian Food Guide, but you have this allowance during the day, that is considered a healthy amount for you to eat. In general, 30 grams of a healthy cheese is considered a good portion, a healthy portion of cheese, like an Allegro cheese or a light gouda, or mozzarella, something that's very light in texture also. But milk is never really included in any diet. It's fat, it's a fat. And it's very hard to digest. Very difficult to digest dairy for many people.

Interviewer: Yeah. So I guess it would be fair to say that you would probably suggest having even a fourth food group called dairy, maybe?

Participant 8: Yes, I agree with Participant 5. I will put it – beverages no, but the dairy yes. Because for example, we need calcium and to have our portion of calcium, our daily calcium need. We have to eat at least one dairy product in the day.

Interviewer: Definitely, yeah. And just also in the interest of time, yeah, sorry, there's a lot of questions. And a lot of people have so much to say about every question. I just wanted to let everyone know that we are hitting - going to hit the 90 minute mark. We still have a few questions to ask. But if you guys have to go, please feel free to leave the focus group. But if you are able to stay, we would love to have you guys stay and voice out more of your opinions. But before we do hit the 90 minute mark, there is one question that I really wanted to ask everyone since we have someone who's a parent, we have someone who is from a different cultural background, we have someone with a learning disability, and some people who are young and old.

So I was wondering on the topic of accessibility, what features do you think would we need to make this app accessible to different people of different demographics and different abilities?

Participant 3: One way that it could work for all different people is - so some apps I can imagine, are probably pretty strict and they're probably really made for those people who are always dieting. But if it was made for people on both sides of the field, that would be a good thing.

Interviewer: Definitely, for sure. Especially people with impairments, like people who live with blindness or even deafness, it would be good to have some features to help them use the app. Yes, Participant 7.

Participant 7: In my opinion, is it possible, if you have a particular setting to adjust, for instance, the font for people who have difficulty working on the font or the text itself? Or if people chose to have the audio? So at least to help us?

Interviewer: Yeah, for sure. So when someone presses a button, they can just say - you can have some audio to say, “Oh, you press this button” and stuff, yeah. Yeah, and I remember in a focus group, someone mentioned that even using the app as a parent would be difficult, because kids can't track their own diet sometimes, because they're not the ones in charge. So I was wondering, what parents would think about the app and how to make it more easy, especially since parents are so busy, right? So it'd be very time consuming to use the app for every child or even themselves as well.

Participant 6: You mean to have the children use the app themselves?

Interviewer: Or track it for them, or even yourself as a parent.

Participant 5: I mean, the colours are nice and easy, it would be good to - I think that's, I mean, relatively simple, the way that you've done it. I would imagine, let's say I was to input a fist size of chicken, if I was to be able to input that in and the colour would pop up on its own. So if you put 100 grams of chicken and 200 grams of salad and I don't know, 50 grams of rice, what does that look like on your plate? And that the representation of that measurement would come out drawn on the plate. So for example, let's say Participant 4, you're making your stir fry, you said the other day earlier. You don't know what's in there.

Well, if you know that you used one pepper, and one carrot and an onion. What does that represent in terms of my plate? So maybe like a conversion somewhat, like a - maybe not an exact measurement, but just an idea of what it looks like on the plate.

Interviewer: Definitely. And Participant 2, you want to say something?

Participant 2: Yes. I'm very interested in seeing how this app works. And I'd really like to know more. But unfortunately, I have to leave because I have to be – I have an appointment at four o'clock.

Interviewer: Oh yeah, definitely. Please go. And just to let you guys all know, as well, since you guys all participated in this focus group, you guys will have your compensatory gift cards within the next week. Yeah, just wanted to let you know before you go –

Participant 2: Is there any way to have it today?

Interviewer: We can send it to you to your email, because it will all be electronic. So we'll just send it to your email.

Participant 2: Pardon?

Interviewer: It will be through your email, we will send it to your email.

Participant 2: Wonderful, thank you very much.

Interviewer: Thank you as well. Bye-bye.

Participant 2: This was very interesting.

Interviewer: Yes. Thank you for staying.

Participant 7: Thank you.

Interviewer: Thank you.

Participant 2: Bye-bye.

Interviewer: Yeah, goodbye. But yeah, if anyone else –

Participant 4: Hey miss. If you guys really need your budget, you can keep it because –

Interviewer: Oh no.

Participant 4: OK, well, I just wanted to –

Interviewer: It’s fine.

Participant 4: OK, thank you. I know, I didn't really participate too much. But thank you for letting me anyways.

Interviewer: Yes, for sure. Definitely, we’ll send yours.

Participant 4: But please make sure that you send me the app, because I’d love to test it out.

Interviewer: Oh, yes, for sure. Thank you so much for your interest.

Participant 4: And I have three brothers and a sister. And I'm sure they would all test them for you, too. So that's the whole group, but just so you know, OK.

Interviewer: Thank you. He’s so sweet. But yeah, there are a few questions that I know will go over time. So I want to be mindful of everyone's time, especially since it's a holiday for most provinces. So if you guys wanted to leave, go ahead. But if you want to stay, we would love that, for sure. Yeah, we have another question. So if you guys remember, the Canadian Food Guide also had the backside that had the qualitative aspects of foods. So being mindful, eating with others cooking more often. Do you think that those type of things or elements should be added to the app, or would it – do you guys think it would be - it would make sense to have those on the app?

Participant 5: Yes, why not?

Interviewer: All right. Sounds good. Do you think even tracking your mood before and after eating would make sense too?

Participant 5: I mean, I've done that for a long time. And I think someone, like I said earlier, who's beginning a mindful journey to healthy eating. It all starts in your head. It's just in your head, it's not what's on the plate, it's not the physical action. It's an emotional response to what you choose to eat. So yes.

Interviewer: Yeh definitely, for sure. Did Participant 1, or even Participant 6 want to add to that?

Participant 6: No.

Interviewer: Participant 8 or Participant 4?

Participant 4: No actually. I don't really know what you mean by mood. But I think these ones that they make here are important. And if they're included, just as a kind of reminder in the app, I think that would be good as well. You don't need to do much with it, I don't think.

Interviewer: All right. Sounds good. Thank you. Also, when using the app, I guess, if we were going to add all the features that you guys wanted to have on your - on the application, do you think, or what type of instructions and support do you think should be provided for users to have when they have the application downloaded on their phone? Should there be tutorials or a help page, or like a tips page?

Participant 4: Oh, one thing I think that could be good is you mentioned earlier that in the actual Canada Food Guide, there are links and other references for people who want to know more about it. So I think just having a page that also includes those links, will be helpful for people who want to do some of their own research or whatever.

Interviewer: So resources for them to go to and learn more about nutrition and stuff?

Participant 4: Yeah, exactly.

Interviewer: I see. Perfect. That would be great. Yeah, I feel like Participant 5, because I know you've used a quite a few diets before. Did you need to use any instructions or tutorials to get familiar?

Participant 5: No. What I liked in all the apps, it was extremely user friendly. So nothing too complicated. There was no tutorial, it was more like, from the beginning, they'll ask you very simple questions to get you used to using the app. So you'll notice, like, click here for this, so you get used to what the buttons look like, and all that stuff. So that was, it was kind of included in your starter kits.

Interviewer: Oh, like a walkthrough, type of thing.

Participant 5: Like a walkthrough. Yeah, sort of.

Interviewer: Yeah, OK perfect. And Participant 6?

Participant 6: Yeah, I'm going back to my original about the objective, because I don't know why I would want to use this app. What is it telling me? Or is it just for my own discipline that I want to track what I'm eating?

Participant 5: I think that's exactly what it is, like self accountability or self-awareness of what it is that you're choosing to eat.

Participant 6: OK, thank you.

Interviewer: Yeah, thank you for answering for me.

Participant 5: I'm sorry.

Interviewer: Oh no, it's all good. I love that how you actually understand, because it means I'm hopefully doing the focus group properly. So as well, what features in the app do you think would kind of improve users’ confidence when tracking their food intake?

Participant 5: You need to have an incentive at the end of the day. So after you've tracked three meals in a 24 hour period, or two snacks, or whatever it is, there's a message that comes into your email, “Way to go, you have successfully eaten your quota for protein for the day. And eating protein for the day will give you blah, blah, blah”, right. So not only understanding that you've eaten what you need to eat, but why you're eating it is important, because most of us just know the mechanics of it. We don't understand that once you've eaten it, what happens to your body when you eat that? And what will it do for you?

So like a fun fact, every time you've reached your goal, or once a week, something that comes into your inbox about grains, about vegetables, about something like that. I think it would keep people coming back too.

Interviewer: Yeah, for sure. For, I guess, even I'll ask Participant 1, if you wanted to add anything? I guess he's muted. So does Participant 8 or even Participant 4 have anything to say?

Participant 8: Maybe some, like self rewarding system.

Interviewer: Self rewarding system, yeah for sure.

Participant 8: Yeah. Because I use an app named the Forest Tree, and it can monitor you to study without using the mobile phone. And after studying for one hour, you can get a tree. That’s very interesting when you –

Interviewer: Yeah, I had that too.

Participant 8: Yeah. When you see the Forest Tree inside your app, you're like, oh my God, I did it. So yeah, my [unintelligible 01:18:51] system will be more interesting for this app.

Participant 4: Yeah, I've seen the app. I've also seen that Participant 8 talked about. I think it's pretty cool. I think it's a good reward to get people incentivized, like study. But also I think, in general, it’s just hard to get rewards that people might actually like care about getting. It's just sometimes it might come off as really tacky or whatever.

Interviewer: Yeah, that's true. Participant 6 what do you think? Do you think having that type of encouragement from the app would be helpful or just not?

Participant 6: Well, not from me, but perhaps – I'm guessing from what I'm hearing, it would be for other people. It won't work for me.

Interviewer: Yeah. Do you think it – were there any things that would work for you, or was it more like a mentality sort of thing?

Participant 6: Yeah. Well, if there was something that told me at some point, you're not eating enough of this or you're eating too much of that, that would be good for me. But other kinds of incentives I don't need.

Interviewer: Oh, so having the feedback for you would definitely be helpful.

Participant 6: Yeah.

Interviewer: OK, perfect. I'll write that down. And oh yeah, Participant 1?

Participant 1: Yes. For me it’s a -I'm sorry, I changed my iPhone and I forgot to switch on the camera and the audio. Yes. I'm completely for the concept of Participant 5, because you have to appeal people to attract them and to make them - to fit the life then for this. So you have some kind of reward or encouragement or congratulation of incentive or what you mean, to make people - they want to –

Interviewer: Use it and come back.

Participant 1: Go and come back. Yes.

Participant 5: Yeah, it's a marketing strategy really. You have to, if you want to put it out there, you have to be able to market it like any other app does.

Participant 1: Definitely.

Interviewer: Yeah. So I just want to thank you guys all for your inputs and your answers. We did go a little over time, over the 90 minute mark. Thank you for staying back. Before I ask the last question, it was more of like an open, common type of question. But I just wanted to say thank you again. We will be piloting another - we'll be having another study to pilot the app. So please let us know if you guys would be interested in that. But it will be very later on in the future, since there is a lot to work on. But yeah, do you guys have anything else that you wanted to suggest for the app that you would personally love to have, if you were going to use it?

Participant 5: For me, I would have to have some kind of guideline in terms of, I'm doing this for a purpose. I don't know how that would work with just it being the Food Guide, and not necessarily some type of healthy eating, get your food on track, kind of approach to the app. But if I was to use it, it would be beyond just knowing that I'm fulfilling what the Canadian Food Guide is telling me to do. I would need something else.

Interviewer: Participant 1, I think you had your hand up.

Participant 1: Sorry. I once again forgot to remove it. Sorry. I'm sorry.

Interviewer: No, it’s fine.

Participant 1: No, it’s OK for me. Nothing else to add.

Interviewer: Does anyone else want to add anything?

Participant 4: I just have one thing to say. I think especially with diet. I think a healthy diet varies between person to person, something might be healthy for one person, but not someone else. And I’ll leave this to you dieticians. Because you guys obviously know a lot more about it than me. But yeah, I think just having that in mind when making the app is important. And I'm sure you guys do, but yeah.

Interviewer: Yeah, I'm getting from a lot of people. I feel like having customizability, having it personalised for each person would be helpful.

Participant 4: Yeah.

Participant 5: Yeah. Let us input our age, our weights, our goal. The reason why we're using the app, what made us use the app, some type of a drop down menu of some sorts.

Interviewer: Yeah. I can see Participant 6 agreeing. She's like, yes, definitely. Was there anything you wanted to add Participant 6, besides those or all good?

Participant 6: No, I'm fine at this point, thank you.

Interviewer: Yeah. So I guess we've reached the end of the focus group. We were 15 minutes over, but thank you. Thank you so much for staying. Yeah, well, just a reminder, we'll be sending those gift cards to you guys.

[End of recorded material]
