# Supplementary material for: Perceptions of self-monitoring dietary intake according to a plate-based approach: A qualitative study
Source: PLoS One. 2023 Nov 28;18(11):e0294652. doi: 10.1371/journal.pone.0294652 (PMC10683993; doi:10.1371/journal.pone.0294652)
Supplement: S4 Appendix — (ZIP) [file pone.0294652.s004.zip › Anonymized GP Transcripts/iCANPlate-GP-focus-Group-1.docx]

**iCANPlate-GP-focus-Group-1**

[Start of recorded material]

Facilitator: So, first off, in your opinion, what do you think makes it, what makes tracking what you eat easy or hard according to the new Plate model? So again, understanding that there’s lots of apps out there that ask you to itemise all your foods; there’s MyFitnessPal, Noom, Weight Watchers has one, they, those apps make you write it all out. So, now we’re going to try to flip the focus and get you just to track in terms of the plate. So, if you think even what you just ate, in terms of your meal, and knowing that half of that plate has to be the vegetables and fruit, the other half, you know, protein and carb, do you think that’s even possible in an app? … R1, I see you’re thinking, I’m totally going to be that person and ask you first [laughs].

R1: I was thinking, like I got, I don’t know if I really –

Facilitator: Because you were like [sshh 00:01:03].

R1: I don’t know if I can really grasp or understand the question as it’s being asked. I guess, what would make it easy or hard for an app to help me plan or schedule my meals, is that what’s sort of being asked?

Facilitator: No. So, just eating according to the food guide, eating –

R1: Just eating according –

Facilitator: – eating according to the plate. Do you think that your diet right now, and it might not conform or not, but if you were asked to track what you’re eating according to the plate, do you think that you’d be able to do it?

R1: Yes, most definitely. It looks like it is pretty easy, like you said, you know, every plate that you sit down for a meal, it’s going to be like a half of the fruit, vegetables and then a quarter or meat and protein and a quarter of the other. So, it seems like, if I were to sit, like I don’t know if I actually follow the pate right now, I definitely don’t. I eat like lots of granola bars, like I go out on, like I pig out on snacks more than I do on, and then, you know, like vegetables and other things like that, so.

Facilitator: Totally. So yeah –

R1: Yeah.

Facilitator: – like it’d be hard to track snacks then, like where the heck do those fall; there’s no mention of snacks.

R1: Exactly.

Facilitator: Agreed, totally agreed. Yeah, R2, you’re nodding, anything to add?

R2: I would say, like for me I wouldn’t say, like I’m conscious of what I eat, definitely, and what my family eat, it’s always on our mind. We try to incorporate a little of everything into our diet daily if possible. I know we’re not big fast-food eaters, we cook the majority of our food at home, we do, we have decreased our snacking. But in order to mimic what the Canadian Food Guide is trying to tell us to do on a daily basis, I think for us, we can do it, but it’s like you, you know, you got to make it your routine.

It has to become your lifestyle just following that to the T and just building it into the way, you know, your breakfast, your lunch, your dinner, it has to be an every day thing, for me to make it work. And I think we haven’t gone that route as yet, we have gone just making sure we’re not like over indulging in like sweets or slaty snacks or, and stuff like that. But and making sure that we keep within our weight class that we, you know, we like for ourself, right. So, I think that’s what we’re doing as a family, but when it comes to Canadian Food Guide, no, I would say definitely we’re not following it.

Facilitator: Yeah, so just building off of Shawn’s comments about snacks and, you know, enjoying food in terms of granola bars or whatever else, you’ve also brought up the fast-food.

R2: Yeah.

Facilitator: It’s true, like where, how do you track like a burger if, you know –

R2: Right.

Facilitator: – it’s like conceptualising as an example, that as a food component on the guide get’s very –

R2: Yeah.

Facilitator: – complex.

R2: Yeah, definitely. And you wouldn’t even want to know how much calories you’re consuming [laughs] in that fast-food burger –

Facilitator: I was thinking about that, you can get to know, because I –

R2: – don’t even go there [laughs].

Facilitator: – well I’m all about, yeah, but even that, yeah. So, those are two interesting points, I totally, yeah. yeah, great points brought up. Anything to add [R3 00:04:30], like just reflecting on you, like barriers, like what’s challenges or what’s easy to eat according to that plate?

R3: Well, if we’re just talking about the raw ingredients, it’s not really issue I think, it’s when you buy something that is part of that guide, but there’s a huge difference between different products. You say you can have cheese, but how fat, how salty, it’s not necessarily the same thing, if I drink milk, should I go to 1%, 2%, whole milk. So, the portions and the types of items I would buy at the supermarket wouldn’t be all equal in that guide and that – Like if you ask me to drink one glass of milk, that’s not really issue, but there’s a huge difference between the types of milk or butter or yoghurt or things like that; sugary yoghurt, plain yoghurt, things like that. I think that thinking about all of this, it makes it hard to really follow closely a diet, because you could be extremely wrong, even though you’re making the right choices.

Facilitator: Yeah, yeah. And that’s where those little details that you might get from itemising it –

R3: Mm-hmm.

Facilitator: – bring it out, and that’s such a great point in terms of, you know, those fine changes in, when you think of yoghurt, like a 9% versus even one or two, a big, big difference. And that’s something you would lose, excellent point.

R3: Yeah.

Facilitator: Yeah. [R4 00:06:08] anything to add on that from your perspective, just like challenges and, or what’s easy for you personally to eat according to the guide?

R4: Hmm, I don’t really have much to add, but I can say that I don’t try to use that guide and I do see that the guide is helpful when you follow it, but it’s hard to maintain it, be on that guide every day.

Facilitator: OK, excellent points. And that’s very normal [laughs], just so you know, that you and everyone else, it’s the biggest challenge is staying to course, right. OK, moving on, I can go off on that. I’m curious to know what techniques have you used to monitor what you eat or track what you eat? Like when I mentioned those applications, did any of that speak to you, maybe not?

R1: I’ll, I have some input on that. From what I gather, for me I’m a type 1 diabetic, so diet is very important and sticking to a routine is also important. And I just want to like reverse real fast, there are barriers involved with sticking to this food guide and that would be in like a multifamily or a multi, like in a unit, in a dwelling. And you have multiple people going out and shopping and there’s going to be like different, you know, items are going to be brought back and that might, also being a barrier there too. But back to the question, the, for me, I don’t find that that routine, I got off topic there and I totally lost what I was –

Facilitator: But you know what, I’m going to gear you off, because I’m interested in this –

R1: OK.

Facilitator: – so what, knowing that you track and you’re obviously very familiar with diet and that kind of stuff, what have you used to help you change your diet or stay to course, like any techniques that you have used? So –

R1: Money.

Facilitator: – I mean like have you journaled –?

R1: Money is a big one. Journals, yes.

Facilitator: What’s that?

R1: Money and journals.

Facilitator: OK.

R1: So, like budgeting a certain amount, because again, snacks do play a big part in my daily routine, you know, having something, like eating every couple of hours just to keep my sugars stable, definitely helps. So, when we’re talking about how this, like what helps on planning on that, and money definitely helps, like budgeting what it is that you’re going to be paying, like weekly or whatever on your groceries.

Facilitator: So, I hear that as planning.

R1: Yeah.

Facilitator: You know, like yeah, planning the meals and journaling, so you’ve mentioned journaling.

R1: Yeah.

Facilitator: Is that paper to pen or an application?

R1: Paper and pen.

Facilitator: Great.

R1: Paper and pen was the way to go, I have actually had a visit with the dietician and we never actually had an app or anything like that at that moment in time, so.

Facilitator: Great, paper to pen is great.

R1: Yeah.

Facilitator: Anyone else, has anyone tracked what they ate, just sort of wrote it on [crib 00:09:21] notes or anything in that sense to keep to course with any diet changes that you’ve done?

R2: I know for me personally, I have never followed any diet, I just tried to portion, like eat within a certain amount and not over eat, like go back for seconds or overindulge or over snack throughout the day, like maybe one snack and then cut off kind of thing. As I mentioned before, like not eating out frequently, maybe once a week or month if that, again it’s not something I crave, so for me it’s easy not to eat out. But I know my husband actually has done the counting the calories, how much calories he should eat per day and he actually did it and he lost quite a few weight by doing that. So, I saw that worked in a sense, as opposed to following the Canadian Food Guide, I believe has –

I am agreeing with R3 here that again yoghurt and the percentage of fat, you know, carbs and all this kind of stuff, is not taken into account. Therefore, you have to track the amount of calories you’re consuming on a daily basis, based under height and weight, male, female sort of thing in order to get an accurate, if you want to lose weight or gain weight, vice versa. So, I think that to me in a sense works and it’s proven, I’ve seen results and its positive results. Yes, it’s a forever thing that you have to do, it’s not just I hit my target, OK I’m at my goal, that’s it, throw it all away and forget it, it’s like you start it today, it’s with you forever and that’s something you have to keep in the back of your mind and I don’t believe a lot of people do and that’s why they regain the weight and more.

Facilitator: Mm-hmm, mm-hmm, yeah, lots of challenges with that, I know. Anything to add on certain things that you’ve done to eat healthier or tried to change your diet, anything to add, R4 or R3, to this? So, we’ve mentioned panning our meals accordingly, like staying in a budget, journaling.

R3: No, not really, I think it’s just trying to keep it in mind in addition to what was mentioned before.

Facilitator: Has anyone specifically on the call used apps? So, R2 probably not you or do you know, yeah, so you don’t have personal experience with that. R4 have –?

R2: I mentioned I did not, but I know my husband has used an app on his phone and he would show me like this, like if you eat this bread, this is how much calories you’re eating, if it’s one slice, two slice, peanut butter. And it’s everything listed out, if it’s organic, if it’s blah, blah, blah and he showed me and I was like, wow, oh my goodness, this –

Facilitator: [Unintelligible 00:12:44] tired –

R2: Yeah, I was like, wow, it exits and people do track and people are using this app and, as I mentioned before, it has worked for him. Now, that he knows what he is eating, he’s not using the app as often as he was when he first started using it, because he already has an idea. But if, there is apps out there and it does exist and it works.

Facilitator: Has anyone else on the call used an app to try to change their diet? No?

R3: No, necessarily to change my diet, but I have used some apps for exercising and, well I’ve tried a few and, in some cases, there were also the nutrition part of it. Pretty much the same logic here, trying to follow what you’re eating, the whole week, but I’d say, from what I’ve seen so far, it was more of an annoyance and it required too much time to do that. And it was not necessarily giving the best advice, it was mostly about calories and things like that, so I guess I could’ve eaten gummy bears all day, but still respect, still respecting the number of calories, so that doesn’t really serve the purpose of having like a good diet.

Facilitator: Yeah, really good points. R4 have, are you familiar with applications to track your diet?

R4: Yes, I am familiar, but I use more traditional way to do it, like paper, like having that on my fridge on what to do.

Facilitator: Great, paper to pen. Just so you know, it’s hard to hear you sometimes, but I got that, but yeah. And R1, anything to add?

R1: There we go, sorry –

Facilitator: It’s good.

R1: – not really, nothing really to add to that, it does sound to me like paper and pen was the way to go. I had, like R3 had said, the exercise apps definitely did have like a nutrition part to it. I have used those, have seen exactly what R2 said, with the choices of the different types of foods that are available on the app, but it was pretty limited exposure to those types of apps.

Facilitator: And again, the same sentiment, that it’s just sort of annoying, requires too much time and effort to complete those apps. So, I mean, I, have any of you happened to have seen an app that mirrors Canada’s Food Guide? No, OK?

R3: No.

Facilitator: No, headshakes all around.

R3: Yeah.

Facilitator: Any questions, yeah, so maybe, yeah, any questions go for it –

R1: Sure.

Facilitator: – we’ll start by that?

R1: I did have a question about that. It looks like in the mock-up and when it comes to the real application, are we going to be like selecting specific food items to put on this plate or is it just going to be general, like you know, grain, veggies, whatever or just made up of –?

Facilitator: Yes. That sort of goes with how does the application work. No, so that’s what’s going to be so unique about this, is that whole point that R3 made about, you know, fat, full-fat versus low-fat or type of granola bars and nut based or chocolate chip, will be not included. … So, when we think about, like the example that you gave, R3, in equally R2, about eating out and about or granola bars, how do you, and do you envision this kind of interface working?

R3: I’m not sure I under, I’m not sure I understand, I understood correctly. You see, you said that we’re not able to change if it’s just veggies or we can –

Facilitator: Yes, so sorry.

R3: – we can, I don’t know, we can select. OK, I’m sorry, I misunderstood.

Facilitator: No, so let’s say you ate an apple –

R3: Yeah.

Facilitator: – or a kind of salad, that’s like a good example. Like –

R3: Mm-hmm.

Facilitator: – just like, you went out and just had like a massive salad –

R3: OK.

Facilitator: – then your whole plate would technically be with no protein on it, your whole plate would just be vegetables, your whole circle would be green.

R3: OK. So, there would be no difference between a garden salad and a Caesar salad in that situation, right?

Facilitator: As long as there’s no chicken on it.

R3: Yeah. I can see that as an issue.

Facilitator: OK, talk more on why, give it to me [laughs].

R3: Well, maybe not since the pandemic, but they’re selling salads at McDonalds and I can assure you that you’re probably better with the burger then with the salad. So, I feel that –

Facilitator: You’re right.

R3: – that this could be an issue here, since not all salads are created equals. are created equal I would say. So, I don’t know exactly, if it’s possible to select something precise that would give me a better idea of, I’m eating right according to the guide. But just dividing my plate, I mean even then, even if I try to not go too far with this idea, I think there would be a huge difference between half of my plate is spinach as opposed to potatoes. If I do that all, every single day, but technically it fits the same category, so I’m not sure that it’s, that’s a good idea to had to have a one size fits all.

R1: I would actually disagree with that, and I’m sorry for jumping in.

Facilitator: No, do it, that’s what this is.

R1: The reason I would disagree with that is because it does seem to be that the food guide is trying to simplify things as much as possible, so I can sort of see why there is no specific size to what you’re filling up on your plate. You, you know, you get, like half the plate’s going to be vegetables, whatever, you know, the proportions that you set up on it, it really sort of gives you, it makes it a lot easier or simplifies the process of sitting down to a meal. The problem that I would have about it, is recording all of this stuff inside the app … I don’t know, it looks like it’s going to be like, you know, like as I sit down to a meal, I have to like pull up the app and start like, you know, saying the proportions about how much I filled out. That’s going to be the only issue I see with it.

Facilitator: Totally fair. Any first thoughts, R2, about that or building off of anything?

R2: Well, I actually agree with R3, because to be honest it’s selling a false pretense that you’re eating vegetables and you’re eating a healthy meal. And for sure, if I buy [Wendy’s 00:20:24] salad I know for sure that calories, oh my goodness, as he mentioned, I’d rather, I should’ve just eat a burger, because I probably had more calories eating that salad. So, for me I don’t agree the way, how this app is incorporating things, I still believe there should be something built in, like calories, portion size, serving size, you know, weights.

Because again, when you weigh certain food, it tells you the calorie intakes and all these things, it’s, that’s why there is so many variations of apps and it works for some people, it doesn’t work for others. Yeah, I mean if we can get one app that does it all in one place, then it would make life easy for everybody. I mean, I could see there is some good parts in this app, but to me, I think others might say, yeah, let me eat the salad from Wendy’s every night and they may gain 20 pounds and be like, oh my God, Canada Food Guide told me to eat salad [laughs]. But I’m just saying, that’s just –

Facilitator: You’re right.

R2: – my opinion [laughs].

Facilitator: Yeah. R4, do you think that you could, you would use the application to record your meals or any further thoughts on that?

R4: I have tried in the past, but it’s really a challenge, you missed a day and then you missed a day that have been not efficient, so that’s why I do it in a traditional way.

Facilitator: So, when we think about that plate and how it looks like, would you, so sort of building off of what you’re saying R4, what would a successful day look like? So obviously, you mentioned missing –

R4: Mm-hmm.

Facilitator: – tracking, so just tracking would be a successful day? but do you think hitting the targets of Canada’s Food Guide would fit your lifestyle?

R4: Mm-hmm, definitely. It’s shape of the, what you receiving daily it balance it, a lot better than the focusing on, of only one portion, having the variety, it’s important.

Facilitator: We spoke a lot about Wendy’s and adding stuff to salads that would make it worse than burgers. So, with that spirit in mind, what, we’re sort of speaking about other foods, right, let’s just say it, the elephant in the room, is there is so many other foods that we consume that don’t fall on this Canada’s Food Guide. So, how, any thoughts on how you would envision tracking that in this kind of interface? … So, for instance the –

R1: I would –

Facilitator: Yeah, go ahead R1, do it, yeah, go, go.

R1: OK. Looking at the interface right now, with the way that it seems to be very general, green, red, brown and [unintelligible 00:23:58], I would say that it, you know, to track the other foods that are not listed there. Maybe have like, it would have to be very simple, because like I don’t want to like, you know, spend like ten minutes marking down what, you know, like extra food that I’ve had. Like maybe like little icons or something that would sit up on the corner or something, showing that I’ve had different food items or something that day or that, on that plate. I don’t know –

Facilitator: And would you just sort of like check a box or would you be interested in writing them?

R1: I could see it more like little icons, like let’s say, I had an apple at 10AM, is that something that I’m going to record in the app, if it is, then I would like to have like a, you know, like a little icon or, you know. Like somewhere, you know, on that plate or whatever, you know, and then like bring it over into just like the other food on there or and maybe I’m missing –

Facilitator: OK, so a visual picture, I didn’t appreciate what you were saying. So, if I understand correctly, with the apple example, you would have literally a picture of an apple that you would or something that would sort of, you’d bring onto the plate?

R1: That would be kind of cool, yeah, you know, something that’s, I find that visual seems to be where we’re all sort of placing our focus right now. And I know that – and to go, get off, a little bit off topic here, I know that science is predicting that in, you know, another 100 years or so our eyes are going to be like bulging out of our head just from evolution, right. Because we are very visual people, like we use our eyes a lot for everything, and so that’s one thing that, well I would like to see. But then again, I guess you go into accessibility issues and concerns as well, but.

Facilitator: What about the info, I’m like so hung-up on the salad dressing thing, because it’s true – oh my, it went out of focus. – how would you track salad dressings on something like this? … Or like R3 for your case and point with like the Caesar versus garden, like do you think that you would have, how would differentiate when you’re eating a salad that’s not so healthy versus one that’s super healthy?

R3: Well, I assume that since most companies are submitting the nutritional value of what they’re selling in Canada to I guess Health Canada or something like that. Maybe a kind of database could be used, so we could select precisely what we’re eating and that would make it easier for us to see the difference between the types of dressing, but even between brands. I don’t know if this information is available, I understand that if it’s not, that would be horrible to really build this database. But if it’s already there, that would be extremely helpful to be able to select precisely, like you’re eating Kraft Caesar salad dressing, so you select this and there’s already the nutritional value of that specific brand or what’s declared to the, to Health Canada or something like that. So, that would be quite useful I guess at that point.

Facilitator: So, yeah, go R1, yeah?

R1: OK. Just to add to that, my take on that question is a little bit different. In terms of salad dressing, I would, I think that would, wouldn’t that like fall under like the protein group or something like that, like a, so it’s just fat?

Facilitator: Yeah, it’s fat, sort of like cream-cheese, people think it’s cheese, it’s actually fat. That’s another really good example.

R1: And cheese sort of fits into that protein portion though?

Facilitator: No.

R1: Oh, doesn’t it?

Facilitator: From a dietetic standpoint, if you came to me for advice, I’d say that’s fat, you can’t count that as a protein.

R1: OK. So, my take on that is, how would you record that in this interface. With the dressing, I would say that that would definitely have to be somehow, you know, you’re going to have to like change the ratio of, well I guess it doesn’t fit in there, like it sort of, it’s tough to say. Sorry, if I –

Facilitator: So, from what I understand, you’re thinking of another category that you could put on the plate?

R1: Not necessarily another category, but to add it to one of the three that are there, but then it doesn’t actually fit into any of those three, so I guess it’s like again, maybe another thing that you would have on the side or whatever of what you’ve, you know, then like an icon and it would be a little Kraft bottle and you’d put it up on a screen there and.

Facilitator: What about things like baked good, like cake, cookies? I mean, technically those are grains, but they’re not healthy, how would you envision tracking that, assuming they’re unhealthy, because some are actually relay healthy [laughs]?

R2: That’s the thing, how do you distinguish, right, I mean you might eat a wholegrain muffin or a bran muffin, and some bran muffins are not as healthy as you think they are. So, I mean –

Facilitator: Right, yeah.

R2: – that’s why for me, I don’t see myself gravitating towards this app, because I don’t, I think it’s got, it’s so much, it sells pretense, like I’m saying. I will still use that word, because it’s not giving me the true accurate information that I am looking for, like calories of what I’m consuming in a daily basis to keep my weight. Like let’s say I what to stay within 135 from now till whenever, if I am counting my calories with the app that I am using, I am putting in everything that I’m consuming on a daily basis.

If I use a vinaigrette dressing versus, you know, creamy dressing, it tells me how much calories I have added to my salad and so and so forth. Where by with the Canada Food Guide, it’s just telling me divide my plate up and use, eat this amount of greens, this amount of fruits and vegetables. You know, who am I to say that I’m actually eating, say I supposed to eat 1 000 calorie in a day, how am I knowing I’m eating 1 000 calorie there, I mean I don’t know.

Facilitator: Yeah, that’s a good point and that speaks to, if you take supplements and you want not to and eat your foods instead –

R2: Correct.

Facilitator: – which you can’t –

R2: If you drank a Gatorade or a Red Bull.

Facilitator: Yeah, or alcohol, yeah, because that –

R2: Right.

Facilitator: – that adds up quickly, right, small changes.

R2: Exactly, yes.

Facilitator: – amount to a lot and –

R2: That’s right.

Facilitator: – additions. What about mixed dishes or cultural foods that might speak to you in your households? How would you track those knowing that you had to, if you had a lasagne or a mixed rice dish, how would you put that into the app? Any further thoughts on that, do you think that that’s a challenge that speaks to you?

R1: I think that would definitely be a challenge, especially with the way that the interface is setup right now. Again, how would you figure that out, you know, based upon the wholegrains or using wholewheat pasta or whatever. How are you going to fill that out on the app and say like, this is how many grains I’ve had of that pasta, plus the protein that came with the meat that was in the lasagne and then, you know, with the vegetables that were in there. I’m not quite sure, that would be a [unintelligible 00:31:22], that base point of the current interface.

Facilitator: Anything to add on specific examples of other foods that you consume often that you think would be hard to track on the app? … No, OK? Let’s just talk about drinks for a minute. So, there’s different kinds of drinks, there’s water, there’s juice, tea, coffee, alcohol, sugar-sweetened beverages; which is in the juice category or your Gatorade, as R2 had mentioned. How do you think that, do you think they should be included in other food or do you think that beverages or drinks should have their own separate category? Let’s start there, what do you think, should they be included or separate?

R1: I think it should be separate. The reasons I would say that, is because beverages can actually contain quite a few calories in them and I know that as a fact with my diabetes, to raise my sugars, you know, have a cup of orange juice and that means that there’s calories in there. And I don’t, and when it comes to the plate, you know, it’s like one thing, but then if your looking at, if the focus is to diet, then my thoughts would be that you’re going to want to record all of these additional food and beverages that actually might add to your weight. And so, it would definitely be helpful if it was separate as opposed to, you know, other on the plate.

Facilitator: One of the big things is milk. Where, what do, if we talk about milk, even if you don’t drink it, how would you record milk on the app? Chocolate milk, strawberry milk, white milk.

R1: I’d put it as a protein, but you know, it’s like –

Facilitator: OK. Anyone else want to chime in on milk? … Any milk drinkers, anyone? … Yeah, that one’s a challenge. Is there any other elements that you – we’ll just move on right away – that you think should be on the app? Like would you guys envision some goal setting on the app or something that would monitor your progress as you go through the day to know that you’re hitting, you know, the end of the day or some sort of reminder or notification?

R1: It would be nice if there was like a, you know, a way to connect with other family members or even just, you know, people that you want to sort of have like this diet, you know, thing going on with. So, that would be pretty helpful.

Facilitator: Yeah, that’s neat, like eating as a group or, yeah, something like accountability, checking in on each other. Anything to add R3, like any sort of features that, like even with your other apps that you have used that help you stick to coarse?

R3: Well, since it’s not necessarily eating three meals a day, it can be not necessarily dealing with a plate, but it can be also your, just the option to add your beverage on the side and see afterwards if it fits with a, a part of a good diet or if it’s just extra calories. Just the option to add more than just your regular plates, since not everything is technically a meal or … pretty much.

Facilitator: Yeah, that’s great. What about tracking your mood or like how you feel which sort of speaks to that second part of the food guide? Would that speak to anyone or not really, not interested?

R1: Not really myself, but it might be helpful to see if there was like some sort of trend going on and feeling one day, so like had that extra-large pizza, you know, like I don’t know.

Facilitator: Anything to add? Do you guys, if we were to create this app and refine it with all this incredible feedback, do you think that something like tutorials would help you, would be useful in the application itself to help sort of explain the app? Any like user feedback on like, you did great today or tomorrow eat this or suggestions and tips, like anything that you think would be helpful to be built in the app? R4, I saw you nod your head; I’m sorry, you did, when I mentioned the tutorials. Can you add to that, like is that, that you’re like, yes, that we need that, we need to have assistance on that?

R4: [Unintelligible 00:36:52] we have, probably to have the tutorials so that they [unintelligible 00:36:59] or if something that they don’t [unintelligible 00:37:01], so we have that, the to go section, it will be helpful.

Facilitator: So, you’re saying the tutorial – again your audio is hard, but I’ll repeat it – that your tutorial, as you were mentioning, would be helpful? … Did, what about –

R4: That’s correct.

Facilitator: OK, perfect, thank you. – a list of foods that would help guide you onto what, where food might end up in the guide, would that be helpful? R2 is nodding her head, yes, yes it would.

R2: Yeah, I think that would be beneficial, because again, we come from all different backgrounds and we eat different things in our diet, so I mean for myself, let’s say if I’m making an Indian cuisine, I might not know like which category best describes or fits it, right. Because it may have cheese in it, it may have some milk and like so many different things are incorporated into this one meal, so it might make it difficult, I might just say, yeah, it’s just a cheese dish, but it’s not a cheese dish. So, that’s where it makes it difficult to just, you know, say it’s not this, it’s others and a whole bunch of other things [laughs]. It’s meat in there, it’s cheese, it’s rice, it’s whatever, whatever, yeah.

Facilitator: Any features that you think should be built in the app to help you stay to course and keep you tracking? So, I mean some of the examples or some challenges, I think that sort of speaks to what you were talking about, R1, if we create some sort of community, like your, yeah, in it together and you get your badges or rewards or whatever that might be; competition. people are shaking their head, yes, yes –

R1: [Unintelligible 00:39:14] much so helpful, having that feedback it always sort of helps, keeps you on track, so. At least I find it so, so yeah.

Facilitator: And something like the peer chat?

R1: Definitely, yeah. Not just because of the [diatracting 00:39:31], but also maybe something as small as, like preparing groceries or whoever got the best grocery shopping. So, if you have like two, like if you live in a, like a two-person home or whatever and there’s going to be like, you know, it would be nice to sort of have that, you know, chatting and be able to like, you know, put together like a grocery list with some [unintelligible 00:39:50] or, you know, something like that.

Facilitator: Yeah, that’s such a great example, hmm. Are there any features in the app that you think could help increase your confidence, so feeling good to tracking your diet?

R2: I guess, like some form of like graph or trending from start date of when you started using the app to current and it’s just showing you your progress, have you, are you achieving your goals, maybe showing your weak spots. You know, those are helpful, its great tips, it helps to motivate you to push on and at least show to yourself that where your weaknesses are if you’re unable to notice it yourself. So, for me, I think that will help some.

Facilitator: Great. Any other feedback on user’s confidence, so again, it’s like feeling good and that you’re able to meet the goals of targeting or tracking what you’re eating? Anything in that app that you can think of?

R2: I think maybe like positive quotes at the end of the day to keep you motivated, like let’s say, even if you may not have meet the goal for the day, but it’s just still a reminder that ‘to keep on keeping ‘on kind of thing, ‘you’re still on course, you’re still on track, you can still get there’, you know, like a self-motivator kind of thing, right. I mean, we’re all looking for that, we all, we’re all thinking I want healthy mindset, right. So, if we can get that in the app, like some prompts, some little quotes, some motivational little, you know, inspirational message that is geared towards, you know, yourself or universal and per se for everyone who is using the app, right. And yeah, I mean I thin it helps, I mean for me, I know when I read something that’s inspirational and it hits home, I really, you know, I keep it at the back of my head or I pass it on to someone else and make their day better, so.

Facilitator: Yeah, that’s great, I never would’ve thought of that. Anything to add, we’re, we have actually only two more questions, so we’re really at the end, we’re doing really well?

R1: I would add one more thing. Maybe something that something that does like, you know, points or something like that, so like if you do meet your goals, it just, like you know, just a number that keeps accumulating or whatever, and maybe there’s like an in-app game or something that, you know, it sort of keeps it interactive and keeps you sort of focused on staying on top of the diet. By that I mean, it would be something, you know like, if I’m using it everyday and I get into the habit of using it, if it’s something that’s fun to use, then it’s going to keep me on there.

Facilitator: OK. Yeah, excellent.

R3: Maybe to add a little something about this last idea. Points for points is nice to know that you’re doing good, but technically there’s not really a goal to have a lot of points, but if we can exchange these points for something that we could use in the future. By example, you get 20 000 points then you would get a $20 gift certificate at a grocery store or something related to eating well, so not like $20 at McDonalds by example. But yeah, and at that point you don’t need to be paranoid if you’re really doing the effort, it’s probably that, probably that the person will make the right choices when using this, but it’s just a little more incentives as opposed to just saying, I made a lot points.

Facilitator: Yeah, incentive, fantastic idea.

R2: Yeah, I think with R3, the point system, I think that would keep the users on your app as opposed to motivate them to want to make the points, so if you want to keep your user, you have to have some kind of incentive to keep them. Like for example, I’m not sure if any of you guys know, like CARROT app, they have like, it’s like a tracking and they do like questionnaires. And it’s a physical app and it does challenges and they, you know, every day they may have a topic on let’s say yoga or, you know, anything and everything, right, or on young moms and then you read stuff and then it’ll give you points. So, that keeps their user engaged and in the app, because you gain points and you’re learning as well.

Facilitator: Interesting, great.

Facilitator 2: Sorry, just to like chime in, but that was the CARROT Rewards App –

R2: Yes, yes.

Facilitator 2: Yeah, OK, yeah, thank you.

R2: So, it, yeah, and it’s linked to like you can cash in your points for [Petra 00:45:18] points or you can cash in to maybe a ballot to win some gift card or whatever. So, I like it, because I am learning from it as well and I am getting points, so.

Facilitator 2: Thank you.

R2: You welcome.

Facilitator: Yeah, that’s great, wow, we just learned something [laughs].

R2: There you go, you can use it as your own [laughs] –

Facilitator: We’re learning a lot – and not just that –

R2: – or incorporate it somewhere.

Facilitator: Yeah.

R2: Yeah.

Facilitator: In terms of accessibility, so for people perhaps with impairment, if it’s audio or language, what are some things that you think could help the users, is it font size? Like I’m just throwing out some ideas and if it speaks to you, chime in.

R1: I would definitely say, font size, contrast, audio would be nice, like OK Google, I have like a 30,30, 30 plate here, just, you know, like something like that.

Facilitator: OK, so you speak in and say, exactly as you said, and it would do it for you and you save?

R1: Yeah.

Facilitator: Got it, OK … Anything to add to that? Maybe not, OK great. Finally, is there anything that you want to add that you think in terms of the application, any other features that you think would help you eat according to the food guide … in the app that we’re proposing? Go ahead, yeah.

R2: Yeah, for myself, as I mentioned before, in order to, for myself to be a user of this app, I would need more from this app, not just visually what’s seen on my plate is good enough for me. I would want to know calories, I would want to know portions, weights, because I think in the sense, that is useful for me. Also, give, having, like motivating me to keep using the app, so the incentives, have like, as I mentioned, the motivational side of it, keeping on top of trends, what’s happening in like, in terms of new diets or new exercising fabs and so on and so forth.

I mean, we all want to keep up-to-date and know what’s happening, I know for myself, I do, I’m not sure if everyone is like that, but I like, not that I try them all, but it’s good to know what exists and what is working for others. So, in that sense, you have to keep on top of it, because you fall behind as an app and then people are like, OK this app is dead, its stagnant. So, for me, if you want to keep your users you have to be ahead of the game and you have to always keep reinventing and be current to stand out.

R1: I think it would also be nice if inside the app you’re able to connect directly with a dietician, whether that was like a paid for use service or something, but having that access to an expert would definitely be helpful to.

R2: Yes, agreed.

Facilitator 2: Yeah, like connected to [Vera 00:48:59] right away [laughs].

Facilitator: I’d be busy. R3 anything you add, like if you had a wish list, like in a diet app that sort of looks like this, what would be like your ultimate?

R3: I would say probably the possibility to search for a whole variety of food items and recommendations with it, if it’s something that is overall recommended, how should, how often you should eat it or things like that. I’ll give you an example, people who want to replace milk with soy mil or oat milk or things like that, so is that good, if you take it should you take it with something else, is it part of a good diet, is there, are there issues with that; things like that.

Facilitator: Thank you

R3: You welcome.

Facilitator: That’s great. And R4, anything you want to add in, hopefully we can, we’ll hear you clearly?

R4: I don’t really have anything to add, but I agree with the comments that made by the other participants.

Facilitator: Great, thanks so much. So, we’ve reached the end, we did it in record time –

Facilitator 2: Yay.

Facilitator: – right. No, that’s fantastic, it’s so great. So, we’ve talked about lots of things, yeah, we’ve got some – [Patricia 00:50:37] there – techniques to tracking your diet and you’ve explained, yeah, it was just so great. Traditional paper to pen is always fantastic and different applications, the pros and cons of eating according to the app and how fast-foods, granolas, Gatorades do not fit. Differences, opinion on what are those different, you know, incentives that can be used and features that can be included in the app. Anything, this is your chance now, anything else you want to add, you know, add in general, like your final two-cents type thing? And you don’t have to, but if there’s something that you just want to get off your chest, this is totally your time. I’m giving it a free-for-all on anything … No, I see nothing. Go for it R1.

R1: I don’t know, I was just like, you know, toying with the idea in my head, like is this app going to be something that’s going to be sold, like is it like a, or a freebie app?

Facilitator: Yeah, it’ll be –

R1: Is this –

Facilitator: – free for the general public, yeah, yeah, yeah.

R1: OK, cool. So, what I was thinking was, you know, like just like the final thing would be to just, the visuals, it’ll definitely need to be very captivating on the visuals now. I know back in the day that you can get away with just a thing like checkboxes or entR1g in text, but I think that right now everybody is still, is pretty much focused on what they see versus how it operates in the backend.

Facilitator: A great suggestion and comment. Anything else, I know R2 you said, no, but we’re good. R3, yeah, R3 go for it.

R3: Wow, yeah, well I was thinking that, you want to eat right just to stay healthy and a good weight, but I assume that people who will download this app, well some of them will want to gain weight, most of them and many of them will want to lose weight. Maybe to integrate a functionality helping people doing that like, how to eat well, but if your goal is to lose some weight, how to do it in a reasonable manner instead of trying the crash diets and all of these fads and things like that. Just according to the guide, but also portions or calories or whatever, I don’t know, how you would do it exactly. But I would feel that this would be a more, a trustworthy source as opposed to a private corporation trying to sell me diet fads and crap like that. –

Facilitator: Exactly.

R1: To add what, to what R3 had said, I would definitely think about having some sort of a – and I lost my train of thought again [laughs]. Yeah, but – OK, so based on that it would be useful to have like graphs, again going back to what R2 had said as well. So, something that keeps a history of what you’ve done and then sort of like uses some sort of like smart, I don’t know, AI or machine learning thing that would sort of – Like if you can put in like, today or well my goal for this next six months would be to lose 30 pounds or something like that. –

Facilitator 2: Right, yeah.

R1: Based upon the history of using the app, maybe it could like sort of, you know, fill out, you know, like instead of you putting in the proportions, it would automatically fill out the proportions for you and then you just sort of follow what, you know, is on that plate.

Facilitator: So, you’re suggesting go in reverse, where the app dictates it to you?

R1: Yeah, yeah.

Facilitator: Given your profile on what your goal is.

R1: Exactly.

Facilitator: OK.

R2: I actually like that point too, what R1 just mentioned, because that makes a lot of sense too. I was also going to mention that, because we have vegan, we have vegetarians, we have meat eaters, we have all these different eaters, right. So, maybe there’s a category you can choose that you are this, right, and then your plate tells you what you eat, so it helps you in that sense. Because a vegan person is, doesn’t eat the same as myself who eats meat and so on and so forth, so therefore their plate will look completely opposite than mine.

So, if that’s in there, that will definitely help them to make sure they’re eating proper and also maybe build in, because a vegetarian and a vegan have to incorporate vitamins and supplements in their diet to offset not eating meat. So, therefore this app should have that for them to make sure it has a reminder, did you take your vitamin or supplements and so on. Or to prompt them if they are unaware, because maybe they’re new to this vegan, vegetarian lifestyle and unaware that they’re missing out and it may affect their health down the road. That’s just my take on it.

Facilitator: Yeah, it’s sort of like having, to R1, building off of R1 and yours, like different plates, as if you were vegan, this is what your plate should look like and this is why –

R2: Right.

Facilitator: – it should be included in it and these are your multivites that we would recommend you taking?

R2: Correct, correct.

Facilitator: OK, we have a lot of work to do [laughs].

R2: Sorry [laughs].

Facilitator: No, this is what I, I mean this is my business, right. –

R2: We’ll zip it, we’ll leave you, if you may have 20 groups more [laughs].

Facilitator: Yeah, and taking over from, yeah, I just think it’s awesome, I mean totally fantastic. I am so thankful for your time, this has just been such a pleasure to meet you virtually over Zoom, I’ve very much enjoyed myself, I hope it’s the same for you. And you know, we’re done, but we’re, you can always get to me or the team through the email, feel free to reach out, we’re conducting these focus groups quite a few of them, all throughout Canada with individuals like yourself. And we are doing it with the experts, which is dieticians, so we have at least 40 to 50 dieticians lined up to speak to us. Given that they were also consulted in created the food guide, so it’s only fit that they should be consulted in helping us create this application.

Facilitator 2: Yes.

Facilitator: So, the next steps are us, is we take all this data, we throw it to our software developer who will help us create the app and then we’re going to come back and pilot it, so it’s going to be super fun. And hopefully we can, you know, we’ll let you know when that happens with your permission, and lots more to come with this application. But again, the thrust of it is just to help Canadians eat healthy, whatever that means to you, exactly to your point R2, and meet the goals that speak to you. So yeah, so again, like any, I think we can stop the recording, we’re –

[End of recorded material]
