# Supplementary material for: Perceptions of self-monitoring dietary intake according to a plate-based approach: A qualitative study
Source: PLoS One. 2023 Nov 28;18(11):e0294652. doi: 10.1371/journal.pone.0294652 (PMC10683993; doi:10.1371/journal.pone.0294652)
Supplement: S4 Appendix — (ZIP) [file pone.0294652.s004.zip › Anonymized GP Transcripts/Icanplate-gp-focus-group-3.docx]

**Icanplate-gp-focus-group-3**

[Start of recorded material]

Facilitator: OK so I can play focus group with members of the general public on July 28^th^ at 5pm EST, ADT actually. And this first part we’re going to ask you some questions about the Canada’s food guide that I just showed to you. So if you would, if you want to you know I’m just wondering how would you see the Canada food guide to follow? What makes it easy or hard for you to follow the Canada’s food guide and eat in accordance to the guide?

Participant 1: I’m not sure I understand the question?

Facilitator: So you know basically the guide, the food guide says that there should be a plate, that half of the plate would be for some vegetables, a quarter for whole grains and another quarter for protein for that. And the details are, yeah and I mentioned some details a bit earlier so I’m wondering what would make it hard for you, or what would make it easy even for you to eat in accordance to the guide?

Participant 1: It was easier with the older guide because if you remember it was all the pie shapes. And I actually still have trouble looking at it the way it’s now configured.

Facilitator: So what makes it hard?

Participant 1: It just seemed easier, the old guide. The pyramids right?

Participant 2: I was going to say it was the pyramids, that’s right.

Participant 1: Yeah.

Facilitator: So what exactly doesn’t make sense to you? The way that you can’t visualize it into your plate?

Participant 3: I guess like, I guess for me like I’m not always necessarily like eating out of a sectioned plate. Like that could be like in other forms or like when you, for example, get a burrito bowl, you get it in one mixed bowl and like you might not be able to separate what are the, like how much of your bowl is from vegetables and how much come from proteins and other stuff.

Participant 1: It doesn’t really reflect the portion sizes. I mean we all know palm, thumb, we all sort of grew up on that, but I’d be hard pressed to know. I mean in the old days it was like was it six almonds or something you know, that was always designated. Was it three ounces of fish, three ounces of meat? Where’s the cheese? You see I find this more difficult to comprehend.

Facilitator: Yeah there some kinds, many kinds of cheese and cream cheese definitely doesn’t go into the guide, but yeah the usual maybe cottage cheese could be included.

Participant 4: Yeah is there a photo of cheese in there anywhere?

Participant 2: Yeah it’s interesting. There’s no products really. It was mostly the old guide that had a lot more, milk had a stronger prominence.

Facilitator: Yes there are many foods that are not included in the guide and we are going to talk about the after I’ve asked you some questions about it.

Participant 1: Well thank goodness we see Axel’s toast there.

Participant 4: [Unintelligible 00:04:14] bread is there. Yeah, no pasta.

Participant 1: They’re only thinking rice pasta.

Participant 3: No chicken wings.

Facilitator: Yeah so as Marlene just mentioned, we don’t have any portion sizes, we just have proportion and you could just see that the proportions, you should follow the, if you want to follow the guide you should follow the proportions.

Participant 1: So one way is a good way because some people don’t like the strictness of the portions. However, other people prefer that. I know weight watchers changed. They used to have portions, but now it’s a different set up. But yeah is that supposed to be on your plate? You should have half fruits and veg and then a quarter protein and a quarter whole grains? Is that supposed to be for every meal or is that in a day?

Facilitator: It is supposed to be for every meal.

Participant 1: Yeah and not many people eat like that combination anymore.

Facilitator: Yeah, but I don’t think it would have [unintelligible 00:05:28] to every kind of meal that we are going to have.

Participant 5: Plus it doesn’t say like how much of each. I mean as you referenced Weight Watchers, I was familiar with it many years ago, I think it was like five fruits and now I think it’s a freestyle, you can eat as many fruits as you want, some are probably more restrictive than others, but you know there has to be some semblance of how many fruits a day or how many vegetables in your portions. I mean look at the bottom, I think it’s tofu and potatoes. Well there’s your carbs.

Facilitator: I think protein is actually the protein.

Participant 3: In the protein. It’s in the top right. I think those are –

Participant 5: What is that?

Participant 3: That’s sweet potato I think.

Facilitator: Yeah. I think the small white bowl is yoghurt. Any other ideas? Yes, Lana?

Participant 1: I find it a little bit [unintelligible 00:06:47] the way it’s laid out because it implies that you’re going to eat all of those things together on one plate and it doesn’t suggest the size of the plate. It could be a small plate or a big plate so I wouldn’t have the fruit on a meal of vegetables and protein so once you remove that it becomes one third of each, but then it would have to be enlarged so I think everybody pretty well agrees that this is not ideal.

Participant 5: It’s not realistic.

Participant 2: For me I actually don’t mind it that much because it is a communication channel. You have one poster and I find, I think people understand, you know people are smart enough to know that it’s eat well each day so we’re not saying this is your plate every day, we’re just saying in the course of your day or even in the course of the week there’s going to be a day, if you have a bowl of pasta, you’re just going to have pasta, it’s going to be mostly in the bottom right corner that you’re in although you might have a bit of sauce and so on, but maybe later in that day you might have more salad and fruit and so on.

But I think you know, I mean I don’t know, unless people are really following a strict diet, you know people just sort of eat, they’re making choices on the flyer with, you know you’re going to the cafeteria at work or you’re going to a restaurant or you’re making your own food at home. It’s not like you’re necessarily weighing so you have to have sort of an intuitive sense of OK I need to eat more of this stuff and less of this or I’ve already had a ton of pasta today.

I’m sorry, Miriam, but you know but you know maybe I need to have more salad just to get, to me it’s a, yeah it’s a bit confusing because there’s a lot going on on this plate. We’re all trying to figure out, now we have to look and figure out what is everything? But overall I think the messages you know, I think we all get it OK like all the fruits and vegetables we need to eat more and then there’s, and I find it really interesting how, in the previous food guide, milk had such a strong presence and now we’re seeing a little bowl of yoghurt. In fact I thought it was, I didn’t realize it was yoghurt, I thought it was dip and I was wondering [unintelligible 00:09:17].

Facilitator: No, we don’t have any sauces or seasoning here.

Participant 2: No I was just going to say when you’re having a bowl of pasta a lot of your nutrients are coming from the sauce.

Participant 3: I think maybe if they took all the ingredients in this plate and then maybe divided it into five plates, five like different plates that would maybe make more sense. So for example one plate with, because no one is going to like eat pasta with toast and with steak and with chicken. But for example, if they have like one plate that had some steak, some mashed potatoes and some vegetables so that would be one plate and then the other plate would be some of the other ingredients here. And they could still fit it on a one page poster so they could have smaller plates, but five of them so that could serve as your diet for the five days of the week maybe and then you would be able to mix and match for the rest of the days as well.

Participant 5: Yeah. But it’s interesting when you look at it what we eat that is not on this plate every day or on the weekends, our glass of wine, excuse me. Or our granola bar after workout. I mean there’s so many foods we all have every day that wouldn’t make this plate.

Facilitator: Totally.

Facilitator 2: Yes. Please keep those in mind for the later questions because we’ll definitely get into all of those.

Participant 4: I find it [unintelligible 00:11:14] I do like the plate and I like the messaging afterwards and yeah, like Axel said it’s you know, a one shot deal. There’s one image, what are we going to put there and I think everything [unintelligible 00:11:28] is there that I need.

Facilitator: Great. Totally, yeah I agree with all of your ideas. And next question, how would you monitor yourself if you want to make any changes in your diet? Like what techniques would you use? Maybe you would use setting your own goals or journaling? What technique would you use when you’re going to make any changes in your diets?

Participant 1: Well what I’m very mindful about are portions. So I have the best scale in the world and I use it, I’m anal about my scale because I’m very generous otherwise. So unless I, you know, put my piece of fish on the scale, I mean nine ounces, you know I could go for a nine ounce piece of fish very easily. And some days I have to say I do do nine ounces because everything else in my salad I would subtract from that.

So I mean that’s my go to is always a scale or measuring cups. I mean again, because my meals are pretty regimented, I measure my cereal every day. I know my cup of cereal, my quarter cup of milk. I do it because I’m just that conscientious about it. I do not measure my wine Friday night. That is not part of the deal.

Facilitator: Yeah that’s fair. So planning you kind of – So you mean something like planning or setting goals? Do you set goals when you want to measure you food? Set any goals, yeah?

Participant 1: Are you asking me?

Facilitator: Yes.

Participant 1: It’s weight management because I know every day when I work out, and I work out seven days a week, how many calories I’ve expended and I’m also, you know I’m also part of the fitness pal and Fitbit and when I run or whatever I do I know how many calories I’ve expended. So I’m very mindful if I can afford that extra whatever, half a cup. And I say you know, cake C.A.K.E., I mean I do allow myself a little piece of cake if I’ve worked out or we’ve done a huge cycle on a Sunday. Why wouldn’t I have a piece of cake?

Facilitator: Yeah, sounds fair. Any other techniques? Any other ideas that come to your mind when making any changes to your diet?

Participant 4: For myself I don’t use anything, I just use common sense and I kind of stick to the yeah common sense for myself anyways. Yeah, I think it’s working. I don’t cook more than I have to because I know if it’s in front of me I’ll eat it and I don’t care for leftovers so I’ll just have what I cook for the day.

Facilitator: Any other ideas. Yes, Axel, go ahead.

Participant 2: Go ahead, Lana.

Participant 5: I just want to say the sort of change I made recently was to not eat between meals. I make sure that what I eat at my meal is satisfying. I think I used to always not get quite enough protein and much more inclined to load up on carbs. And I never was a big egg eater, but I’m sort of realizing that it’s probably better to just not get the munchies after and to just stick to three meals a day and maybe a piece of cheese with half an apple or something as a snack occasionally. But I mean that’s the way to go, for me anyway.

Facilitator 2: So Lana your technique is in your head? You’re not writing this down?

Participant 5: Yeah it’s absolutely in my head. Get out of the kitchen. You know otherwise too many temptations.

Facilitator: Axel you wanted to add something?

Participant 2: Yeah. Some of us live like Tour de France riders, they’re measuring everything they weigh and calorie intake and calorie outtake. I’m like wow, that’s intense. But I’m more on the Lana side of things. You know I sort of, although I mean we’re a family of five, I have three little girls and so [unintelligible 00:16:34] so we have a take no prisoners policy when it comes to cooking. So I do use the scale to make sure I have no leftovers because otherwise we you know there’s too much stuff.

But for me, it’s also, there’s two things. One is habits when we go shopping right? So my wife and I, we’re trying to reduce our milk intake. Partly because of the kids they’re drinking milk and we’re drinking milk and we’re trying to you know, so now I’m buying different types of milks and they’re really good these different alternative dairy [unintelligible 00:17:11]. I call them dairies, but they’re really like oat milk and almond milk and those kind of things.

And the second thing is for me is to try to go to bed earlier. I find that if I’m up until 11 I’m hungry and then I can’t get to sleep and so if I just go to bed like just an hour earlier, I don’t intake those calories. So I’m trying really hard to get that done. It was hard when I was deep into the MBA stuff, but now I’m almost at the end so I have a bit more time. But I found that I put on a couple of pounds doing that because you’re working late and then you get hungry and then you start eating and then you go to bed. And anyway, so it’s not scientific at all just personal observation.

Facilitator: So [unintelligible 00:18:08] do you have anything to add?

Participant 3: Not particularly. I’m kind of like Lana where it’s all in my head, but I typically just look at the information facts behind the boxes when I buy stuff. And I don’t have like a, I don’t know like a hard or fast rule for it, but I typically just look for anything that has any like too much sugar or too much fat especially like trans fats. So I typically try to stay away from those like really a high fatty or a high sugar [unintelligible 00:18:51] foods. That’s mostly it.

Participant 1: Yeah I’m definitely into label reading definitely.

Participant 2: Yeah me too.

Facilitator: So I knew that a few of you mentioned that you’ve used some applications before, can you tell us what was easy or hard with those applications when you were tracking your diet?

Participant 5: I’ve never used an app.

Facilitator: I know that Marlene said I think Lana.

Participant 5: Yeah I used to have sort of you know like one of the earlier fit bits. It was called [unintelligible 00:19:44] And it came with, that was part of the app on the phone was you could check off what you’ve eaten that day. And I just found it a little bit tedious to have to do that. You know to sort of look for your particular food in the list. I didn’t find it very helpful.

Facilitator: Yeah it took a lot of time right?

Participant 5: I can’t say a lot, but more than I was willing to give it. It was just tedious.

Facilitator: Sounds fair. Yeah Marlene how about you?

Participant 4: Well as part of my lifestyle, I mean it might sound boring, but I do find that I’m pleased with the results so you know it’s not tedious. I mean from the days when I was on Weight Watchers you have to write everything down, record it. So it’s just part of my make up, my whole psyche is that, as I said, I’m a creature of habit so two minutes when I come in from my run or cycle or whatever, I just put it on my phone, it’s recorded, I know exactly what I’m having for lunch, dinner, you know. And it just works for me, it just you know? It’s my benchmark and perhaps it just keeps me focused on what I know I can eat and as I said it’s a lifestyle, it’s like brushing you teeth. You don’t think about it everyday. You just do it.

Facilitator: Yeah so there’s nothing [too hard? 00:21:23] about it for you?

Participant 1: I did tell my sister about this focus group because I was trying to get her on and she says “A diet app? I’m sick of inputting information. No. Pass.”

Facilitator: Totally understandable.

Participant 3: Valuable feedback.

Participant 2: Maybe I could, I’ve actually never worked with any kind of nutritional app, but I think I would be sort of like Lana and say you know after a while it’d be tiresome to have to fill out everything I’ve eaten every day and I have worked with dieticians before and they do have these food journals that I’ve found really intense just to see because in a past life I actually worked with Tamara a long time ago and I see what they’re doing and it’s pretty intense what they ask.

But I’m a little bit like Marlie as well, I like to run and ride my bike and so on. And I’ve discovered a couple of years ago this app, Strava. I don’t know if anybody else connects?

Participant 1: Oh we’re on Strava.

Participant 2: You guys are on Strava? OK we’ll hook up on Strava. But Strava is great.

Participant 3: I have my own channel.

Participant 2: What’s that?

Participant 3: I have my own channel on Strava.

Participant 2: You have your own channel? OK yeah. Yeah and so it’s good because the data is automatically entered so I just my watch on, whatever watch it is it doesn’t matter. It tracks your heart rate, it’s got a GPS and then it goes right on to the Strava application. And what’s cool is that then you’ve got friends, like you know friends from all over the place and they’ll like it or whatever. Sometimes we comment on some of the course we’ve done or how well you’ve run or whatever. You know there’s a bit of banter.

And so I was thinking you know what would be really interesting now, you know we have a, there’s a lot of imaging technology that could be part of this new app where all you have to do is with your phone you just take a picture right? And then the picture can analyze what’s on your plate or on your bowl or whatever. And then maybe it’d be less tedious and it’d just be like just like what Marlie was saying, oh it’s a habit. Oh I just take a picture.

And the cool thing is maybe you’ve made something really nice, you know you cooked something for your family and you say look what I just made, I just made this awesome lasagne, whatever it is. You take a picture and then you know, your other friends who are in the same boat can comment, oh how did you – Whatever. Where’d you get that cool gluten free pasta? Or there could be a bit of back and forth in the community that could start to –

And so then you start to get more into it as opposed to just saying oh I’m entering data for myself or for a study or something. You know there’s something that you get back out of it right? Anyway I find the Strava model works, it works well for me because I’m sort of like Lana, I just kind of do stuff.

Facilitator: Yeah. That was great. OK so do you know any [unintelligible 00:24:54] monitoring applications that resemble Canada’s food guide? Like having the plate method? The plate method, the plate concept?

Participant 3: I don’t think so, no.

Facilitator: Having this information I’m wondering how would you envision this app working when you think about your eating throughout the day?

Participant 2: And maybe an idea, you know one thing maybe that could help the app is if you could turn on the camera option and so not necessarily to, you could take a picture maybe, but just to say well here, you want me to figure out what the proportions are, maybe I could just visualize it on the phone and then overlay your disk right? And then you could say OK well with my finger I can sort of adjust, based on what I’m seeing in the image I can adjust the portions because it’s not an exact science, you’re just sort of saying oh about roughly this.

Facilitator: Something like using AI you mean right?

Participant 2: No. No AI, it’s very simple, just saying it’s just an overlay where you’re saying, I mean AI would be cool, but in the first step just to say OK you have the overlay like you just showed us, but I could see my plate at the same time.

Facilitator: Oh so kind of like tracing it?

Participant 2: Yeah you can trace it.

Participant 3: Kind of like AR.

Participant 2: Right kind of AR. Exactly right so you can just like I could sort of trace on top, just as if you take a calc paper or a transparent paper and you’re drawing over, well here you’re drawing over your plate. Oh yeah, OK. I mean obviously if it’s a plate of pasta, then it’s just going to be 100 percent I guess. Or you’ll have to guess because it’s all mixed or a salad or whatever. But in a plate where it’s set up in a way where I could just do it that way that’d be kind of neat. But without going into AI because AI is a whole different, that’s a whole different level of complexity.

Participant 1: And where do the snacks go? Like the orange that I had at 3 o’clock or the boiled egg that I had at 10? I don’t eat plates at a time. I’m just –

Participant 2: Eating. Yeah.

Participant 1: I mean I do once a day, but the rest is just yeah, not so rigid.

Participant 2: That’s true. People don’t necessarily eat three meals a day.

Participant 4: Yeah. I don’t have a family to sit down with and eat a meal in the evening. It’s whenever.

Participant 3: And unless like you have maybe OCD or something you don’t necessarily separate all your vegetables from your proteins, from your wholegrains. So for example you might have like one piece of steak is here, the other piece is here so you can’t really like say you know, you wouldn’t necessarily take them out.

Facilitator: Yeah it would be hard or mixed dishes I think, yeah.

Participant 5: Yeah I think I’d be very hard pressed to try and dissect everything in my salad. It’s one toss and there it is.

Facilitator: Right. Something a bit more like visualizing everything according to the plate method. Yeah?

Participant 2: Yeah I think for the app to work that would be one view, but you might have other views that you would need where you know, like Vanessa saying oh I just ate an apple or an orange I should toss it in there.

Participant 4: An imagine would be good though to take for memory because then you might want to go back to it later or revise and I don’t know, but yeah. Photograph would always be good.

Participant 1: And is the purpose of this to track what you’re eating?

Facilitator: Yes.

Participant 1: OK. Over the course of the eight hours or 12 hours of your day?

Facilitator: 24 hours.

Participant 1: Really? 24? OK.

Facilitator: Yes like almost everything that you eat throughout the day.

Participant 1: OK.

Participant 2: Right. So my midnight snack has to be covered in there too right?

Participant 3: That’s why you go to bed early.

Participant 2: Yeah so I don’t have to enter it into the app.

Facilitator: Yeah and how are you going to enter that into the app? The snack. Yeah.

Participant 1: Exactly.

Facilitator: OK so how would you represent the different, how do you think should different portions be presented in this application? The meals portion.

Participant 4: Can we see the app again or is that what we’re just going to see that one time?

Facilitator: This is just a [mock up? 00:30:30] Yeah we totally can show it again to you.

Participant 4: We didn’t talk about the imagines at the top did we?

Participant 1: The fork and knife.

Participant 4: And the plus is?

Facilitator: Other foods. Yeah anything that you want to add? And the pink one is for the protein products, the green color is for fruits and vegetables and the orange color is for the grain.

Participant 2: Oh OK so if, for instance, when Vanessa eats an orange she’d hit the plus button and then you’d go I guess veggie, 100 percent or?

Participant 1: No an orange is a fruit.

Participant 2: Right but I don’t have the fruit option here.

Facilitator: The green represents for all fruits and vegetables.

Participant 4: Why wouldn’t it say fruits and veggie? It should say fruits and veggie?

Facilitator: Yeah it’s just a mock up so forgive us for that.

Participant 4: And the drop is for liquids meaning any kind of beverage?

Facilitator: We are going to talk about it later.

Facilitator 2: I think at the moment the drop means water.

Facilitator: Yeah at the moment it means water.

Participant 2: OK so wine doesn’t count?

Facilitator 2: Yeah in this mock up, unfortunately wine does not count.

Participant 4: So your question is how would you represent the different meals portion on the plate?

Facilitator: Portion. Right.

Participant 4: I don’t know, you tell me.

Facilitator: For example, portion sizes for example, do you think there should be some standard references or should be some measuring tools that would be standard? For example if it suggests –

Participant 3: So do you mean like the portion of the plate so the side of the plate?

Facilitator: The portion of food that you’re going to consume. For example, how would you measure them? How do you think you could measure your food and track them in the application?

Participant 1: Well as some people said I mean just they can visualize it. So when you buy your piece of fish or meat or whatever, they know it’s for one meal or it’s for two meals or whatever right? So it’s just visually or in some people’s cases they actually take out a scale. But the question is again, how do you take that food product and put it on the app? Are we, you know is there, do you take a picture of it? I mean I don’t know.

Participant 5: Yeah so if it’s an apple I would click on the plus I guess or the fork and knife. I’m not really sure. Put one next to the image of a fruit or vegetable and then that image will populate in the green and eventually all the green will be filled up when you’ve had all your fruits and veg.

Participant 3: I think maybe the plus would come into play like when you’re eating something that’s not in any of the three categories. For example, I don’t know, cheese or like snacks or like a chocolate bar, something like that.

Facilitator: You know, so basically can I just, I just want to prepare some more information. So basically when you’re going to track what you’re going to, what you’re eating, I mean the portions that you’re using, how would you represent those portions? For example, if you’re having some rice, how would you think is the best way to track, to know about the portion and how to track it? For example, can you measure it by the spoons, table spoons or something like the hand, hand references? How would you think representation -?

Participant 3: Cups maybe.

Facilitator: Yeah cups even.

Participant 1: Or one serving spoon, like a big one. You know that’s it. You don’t start picking again. What’s on your plate you stick to it.

Participant 2: In the UK they have a system that I think Marlie would really identify with where they show you how long you have to run if you eat this. Or how long you have to walk. It’s actually controversial because it depends on your height, weight and things, but it’s kind of like OK if I eat this I have to go walk for an hour.

Participant 1: Oh my God, well that’s definitely pressure.

Participant 2: I was just going to say it’s a tricky question. I mean the pie chart I think is kind of a universal, I think people, I think universally will sort of understand the pie chart concept so I think this is pretty good. It kind of depends, too what level of accuracy you require because we already saw earlier, we weren’t exactly sure whether tofu is a protein or is it a veggie? What is it?

Sometimes there’s some things where you make, or even what is vegetarian right? So you may have like errs that are going to come into your data set when you’re, given that you’ve got these very broad groups, but I understand why you want to do that because you know again, like some of us were saying well you know entering data is kind of tedious so we want to make sure that people do it. So I don’t know, I think this could work, but –

Participant 3: I had an idea, but I don’t know would it work or not. Like it depends on your budget for creating this app and like how you would be creating merch so I’m thinking like maybe if you could come up with summarized plates or just like different plates or bowls. And then for example like those would be your standard bowls for the app.

For example whenever someone registers an account they would like get one of, like one set up like these plates and then like since your application already knows the portion sizes in all these plates and they’re standardized in your application when someone, for example has some rice in this section, the app already knows how much rice this and how many calories is are in there.

Participant 2: Yeah that’d be cool.

Participant 3: Like theoretically, but like if it could be like expensive to be time consuming, but it’s an idea.

Participant 1: I mean you almost want the app to be preloaded with every conceivable variation on a theme of food. So if we’re recording it or however we’re getting that information to the app, it’s already in the app.

Participant 3: Yeah I was thinking about when you are, like put everything in the app and you could just like bring your phone over it, take a picture and then it would know that like OK so this section is for the veggies.

Participant 1: You’d think that would be.

Participant 5: But also the question is how do you track the amount? If you’re supposed to have half of your 24 hours being fruits or veg, you do have to write an amount. So the total, 100 percent in the end so if you’re saying like should, and rice is really difficult and I guess most of us are familiar with recipes and recipes we use cups and table spoons and all that. So I guess stick with that form of measure. If you had a cup of rice you’d put a cup of rice.

Participant 1: But yeah, I think the app should do the work for us.

Participant 5: But how does it know it’s half a cup? By picture.

Participant 3: It’s the app’s problem.

Participant 1: I think whatever we enter the app should be able to calculate it. So if we have the rice and that’s part of a carbohydrate –

Participant 5: But you have to enter an amount is what I’m saying. You can’t just take a photo of rice.

Participant 1: No I agree, but I think the app should be able to recognize it.

Participant 3: Yeah that’s what I’m saying because even if you like you know that you’ve made two cups of rice, you may not eat the whole thing yourself in one sitting. For example, you might share it with someone else and you might have some leftovers, like you shouldn’t go through the trouble of like [unintelligible 00:39:59]. So I made two cups of rice and I had 40 percent of it and like that would make it, you know like that’s too time consuming for users.

Participant 1: You’re only showing the app what you’re consuming, not what you have like on the table remember?

Participant 2: Yeah and are we allowed to have seconds of rice?

Participant 1: You don’t have to tell the app that.

Participant 3: No one needs to know.

Facilitator: So as you all mentioned, there are many other foods like the granola bar that Marlie has after her work out and some other foods like sausage, kidneys, everything that are not placed in the guide. So how do you suggest tracking down in this application? And what other foods can you even think of that was not already in the guide?

Participant 4: Yeah so like granola bars, would you have to break it down into what? Like oat [unintelligible 00:41:26] are in there?

Participant 5: Exactly like the grains. The grains, the sugars, the nuts.

Participant 4: I think miscellaneous would be, yeah. And you can choose from the miscellaneous. What about herbs, like spices, herbs?

Facilitator: What about sugar?

Participant 1: Yeah. Or all the condiments. I mean you know the chutneys that we do and the sauces. I mean when we put marinades on thing. I mean how far do you go here?

Participant 4: Scrap the app. Call it Scrap.

Participant 1: Yeah, life is too short.

Facilitator 2: We sure have a lot of work ahead for this app. There’s a lot more muscle work.

Participant 1: [Unintelligible 00:42:15] going to upload all the different categories of foods. Then that means you’re going to be spending time scrolling, looking for your particular granola bar or whatever. Is there no way like hey Google, is that considered AI? Like just tell him or her what you’re reading and approximately how much and let them figure it out.

Facilitator 2: Oh like hey plate. What is on my plate?

Facilitator: Yeah interesting.

Participant 1: But how is it when I use my Fitness Pal and I’m adding my food, sometimes what I can do if it’s not on the Fitness Pal, I put my phone to the bar code. Automatically it comes up so it tells me exactly what I’m eating.

Participant 4: There you go. Perfect.

Facilitator: We can use it for foods for that that have some labels and bar codes or even [unintelligible 00:43:24] in some databases that Lana mentioned. What about some other foods that are not packaged or does not have any barcodes like some mixed dishes or some international food. Or how many cakes. Yeah, what about them?

Participant 4: Yeah I mean scan the recipe that I made the cake from and there’s all the ingredients.

Facilitator 2: Oh wow. I didn’t think of that. OK I’ve got to write that down right now.

Participant 3: Ask the cook.

Facilitator: Do you think there should be already some recipes down there or just followers would share with other users?

Participant 4: Yeah I don’t see why not, you can have a recipe section, sure. I think that would be a good idea. I don’t know if it would be, like who would be choosing those recipes or would all users be sharing because then -?

Participant 1: Yeah and make it to like privacy, not privacy, but are you allowed to share somebody’s, like Jamie Oliver? Are you allowed to share that? I don’t know. Like we don’t want law suits here.

Participant 4: Or we should be sticking to heath recipes and if that recipe isn’t healthy and you know, how much sugar is -?

Participant 2: Well yeah it should somehow stay anonymous right? It makes a big difference in terms of your honesty level into how people enter. A long time ago we did a study on concussions on football players in the CFL and it’s amazing how we had to, in the first study they put their name and all of a sudden, it was weird because there was no concussions in the CFL whereas in university football there’s tons of concussions. Like what’s going on?

And then we made it anonymous and realized OK so it’s a totally different result. So I’m just making a parallel here that you want to make sure that if you want, you know people, yeah I mean some people eat cake sometimes and it’s OK. But we still have to be great if we could get that information right? So they have to be confident that it’s not going to be on Instagram, oh my God, Axel just had cake last night.

Participant 3: Yeah and then you open Instagram and just like [unintelligible 00:46:08], like how do you know?

Participant 2: Right, exactly. And my colleagues at work like hey what’s going on?

Facilitator: So about beverages. How do you think we should try beverages on this application? Anything like sugar sweetened beverages or wine, coffee, tea, Friday afternoons wine? Anything.

Participant 2: I really honestly think that the bar code idea is great. I think that’s key, but also the image recognition that I think will get you the most accurate. You know if people could just sort of, oh I’m eating a mango and I can, you know it’ll recognize it. Oh I’m having tea. You won’t know what kind of tea it is, but at least you’ll have an idea that OK this is kind of a water based beverage and maybe the person can add a comment, you know I threw in some honey in my team so now you know OK that’s sugar.

Participant 5: It’s water, apple cider vinegar, lemon juice, cinnamon, ginger.

Facilitator: How you going to track it?

Participant 5: Yeah exactly.

Facilitator: Show me the recipe.

Participant 2: I’m going to Vanessa’s place. I think we’re neighbors anyway. Sounds pretty good.

Participant 5: No it is. It’s actually for acid reflux which I get so I’m supposed to drink a bunch of this every day and it’s working actually. It’s been a month.

Facilitator: That’s great.

Participant 5: But that’s I guess, easy to upload because it is a strict measurements, but to take a photo of it then, no there’s no barcode for that.

Participant 2: But you’d have the option to put some comments like oh this is something you know this is a concoction I prepared and I’ve been having for a month now.

Participant 3: And I guess the variety of like different drinks that people drink on a weekly basis isn’t that high. So for example, like you will make this special drink and like drink it for a month. And it doesn’t change so once you put it in the app you can just say like oh like add in the drink that I had the other day. You don’t need to add it every single day so that will make it easier. So yeah, you don’t drink like hundreds of types of drinks, like custom made drinks every single day. So I think that’s kind of, yeah.

Participant 2: That’s interesting. I’ve never thought about it, but yeah.

Participant 5: Like what about kids, younger people. They’re drinking anything that’s available to them when they get [unintelligible 00:49:03] and then they go and spent it on bubble tea.

Participant 3: Yeah it’s tough at Starbucks, but I think like the variety of drinks that you consume on like a weekly basis, I think that wouldn’t be that high.

Participant 5: And would people not tell the app that they’re drinking the Monster drinks and the energy drinks?

Participant 1: Good point.

Participant 5: So honesty is there too.

Facilitator: But those have bar codes too. Yeah. So do you think they, even we need to separate beverages from meals?

Participant 1: Well some people include it as a meal.

Participant 2: Yeah I do. I would.

Participant 1: Like a shake. Yeah I would.

Participant 4: Smoothies.

Participant 2: And I think beverages can have a huge impact on your calorie intake.

Participant 1: Absolutely.

Participant 4: This isn’t a calorie counter right? This is not a calorie counter I don’t think.

Participant 2: No but it’s just like, I mean Miriam mentioned sugar. If you look at the amount of sugar that’s in a soda or a pop can, it’s enormous. It’s hidden by the carbonate right. So that makes a huge difference.

Participant 3: It will contribute to like your sugar intake. Yeah.

Facilitator: So what other elements, you saw that at the backside of the guide or there are were some other elements that the guide advised us to do those suggestions. And do you think we need to include some of those in the app? Like mindful eating? Or like yeah tracking some moods, tracking feelings? Do you think they can be helpful if they are in the app?

Participant 1: That’s a good question.

Participant 4: As reminders I guess like limit the high sodium sugars and saturated fat. I mean, enjoy your food, yeah obviously. Maybe some images would help you to convey, enjoy your food or use food labels. You don’t have to use the text, but some images.

Facilitator: Like daily reminders or daily images right?

Participant 1: Yeah, it wouldn’t hurt. It definitely wouldn’t hurt.

Facilitator: So someone in our previous focus group suggests that people could read those suggestions and get some points. Do you think that can be helpful?

Participant 4: Points to do what?

Participant 2: Like a reward program?

Facilitator: Yeah for example to read more kind of advices.

Participant 3: I have one like suggestion here, but like it could be kind of like a hit and miss. For example, one of them is marketing can influence your food choice. So I realized that some of the applications like Uber Eats and [unintelligible 00:52:25] and stuff like that, like McDonalds, when you have their app on your phone, typically around lunch time or dinner time like you’ll get your notifications like do you get hungry? Here’s $2 off from so and so. So you can actually beat them by half an hour so like 11:30 you could just say “Beware of the McDonalds”.

Participant 2: That’s awesome.

Participant 4: Time to turn off your notifications.

Participant 3: Go for a walk and don’t look at your phone.

Participant 4: And if there is some notifications on this app, of course you’ll have the option to opt out or notifications once a week instead of every day. Or different choices.

Facilitator: How about tracking moods? Do you think it can be helpful?

Participant 4: Tracking?

Facilitator: Moods. Feelings.

Participant 1: I mean I think you’re opening up a whole different kettle of fish here.

Participant 4: I don’t know about tracking moods.

Participant 3: It could, but I’m not that person, at least not at first I think, maybe because if an app wants to do everything at once especially in it’s earlier stages, it might kind of go south.

Participant 5: Yeah you’ve got to figure out who’s your target market here? Who are we going for?

Facilitator: Everyone. Almost every adult.

Participant 5: So a few different choices would be good for the person that wants more obviously and then the person that wants less, like me. I don’t feel this [unintelligible 00:54:23].

Participant 2: Yeah I think it’s important. I mean all those questions are great. I think it’s key that the person that’s going to use the app gets something back somehow. And this is, I mean I’ve noticed this in the workplace too, if you’re going to ask people to enter information and data they have to see a benefit for them like in their work or in their lifestyle or their choices. So it could be, I mean yeah you could have sort of a reward program, but I don’t know if that, I mean you’d have to look at research on that, but I don’t know how motivating that is unless there are like airline points or something, I don’t know. But I think people are motivated, they come from a different place of motivation when you’re talking about nutrition.

Facilitator 2: Or maybe grocery gift cards?

Participant 2: Sure.

Participant 5: Is it going to be linked or promoted by grocery stores? Like who else is involved?

Facilitator 2: Oh that’s true.

Participant 2: Yeah see I’d be careful to do that because as soon as you put a commercial component to your personal motivations it just, you –

Participant 3: People think you’re after the money.

Participant 2: Well yeah and it’s demotivating.

Facilitator: Totally. So what do you think could help people to tracking, engage them more using the application?

Participant 2: I think the social aspect is probably one of the key motivators and if a lot of people are using it, you know the biggest motivator for most people is well if others are doing it, I’ll do it too. Right? And it’s hard to get there because it means you’ve got to go through these early adopters and eventually get to, but if there’s kind of somehow an exchange like yeah you’re putting up information that you know a research group can use, but that’s great, but I can also connect with my friends or my community in a way and exchange on something that’s very important and during the day [unintelligible 00:56:58].

Facilitator: Yeah so you’re meaning an in app peer social media set, something like that?

Participant 2: Yeah like I was saying earlier, with the Strava, I mean all those, you know Instagram, Facebook all these figured out that oh you know people actually want to connect with each other. They don’t necessarily want to have a big conversation, they just want to say you know, hey look, check out the salad I just had yesterday and I’m really proud of it. Or check out my new tea concoction. I’m feeling better and maybe you want to try it too.

Facilitator: Yeah. Great. So any other ideas for people tracking and using the application?

Participant 3: I just wanted to, like about, like tracking feelings and moods. I noticed that it says be mindful of your eating habits on the kindness food guide. I think even without tracking feelings, just by like entering the meals that they were eating and for example, and so what they’re eating at what time and like what’s the frequency of their meals? Like what is the time between their meals?

Even that they are not doing anything extra, you can like have so many, like extra so many data just from those data. Like so for example you know that this person eats every 30 minutes or every eight hours. And maybe like after a month or two you can like kind of remind them that like this is your eating habit like in case you didn’t recognise it. Or like you ate every four hours on week days, but on weekends you just go haywire, just like go everything inside. Yeah and that basically could be making people mindful of their eating habits.

Facilitator: Lana did you want to add something?

Participant 5: I just wanted to say, you had mentioned earlier, attributing extra points for something and I didn’t understand what was the point of the points. What do you do do with those points?

Facilitator 2: Yeah I don’t see any point.

Facilitator: We think maybe people, maybe they could, I don’t have any idea about it yeah.

Participant 3: You get to have cake. 200 points worth of cake.

Participant 4: But if it was some monetary value or a gift card or something from someone that would work I think. For myself yeah, I sign up to things because of what I get in the end. My $25 gift card. However I am interested in this, I am. But yes I do like points.

Facilitator: But you didn’t know about it, the points.

Participant 4: Yeah some of them are just going to give me a smiley face or something. No, no, no. I want something [unintelligible 01:00:14].

Facilitator: Yeah I know that.

Participant 4: I was going to mention a lot of people are using the Good Food Boxes, you know how they’re [unintelligible 01:00:21] so would they just get the box and then, I don’t know if they were scanning, I don’t know if there’s a bar code on that or how it works, I’ve never ordered one, but at least things are separated out and maybe it’s easier for them to track [unintelligible 01:00:38] a Good Food Box because that is getting really popular. I don’t now if those are the people that would be using this app.

Participant 2: Yeah. And I just want to build on what [Reza? 01:00:52] was saying. I think the sort of a control center, not a control center, but like a dashboard is the word I’m looking for. Like a type of dashboard where you can get information about your stuff, I think also it would be good. Like you’re entering all this information and at the end of the month you get this dashboard and maybe that’s where those messages comes in. Hey look, we can tell you’re making an effort based on what we’ve seen in the previous month.

Or hey, it looks like you’re eating a lot out at the restaurant, maybe it’s time for some home cooked meals. Or sort of very sort of action oriented messages as opposed to saying you know people should eat more or should cook more meals at home. You know just hey, based on what you’ve entered in the last three weeks you know, you haven’t been home. Maybe it’s time to you know –

Participant 5: That’s bagging on people being honest.

Participant 2: Yeah but it’s, I mean it’s based on, that’s why they have their own dashboard. It’s about themselves right? I mean yeah you can lie to yourself and that’s your prerogative, but if you’re going to do it right then –

Participant 1: But you know sometimes when you finish a workout I mean the little symbol comes off and it says oh yeah you’ve reached your goal or [unintelligible 01:02:22].

Participant 2: Yeah, yeah. Little things like that right?

Participant 1: So it just means like it’s funny, it’s cute, but it’s validating. All you really want is to just validate that you’ve done it and move on. I mean I don’t think I, like I’m not interested in a reward or cashing in or whatever. You know what, I’ve done it for the day, Bob’s your uncle, let’s move on till tomorrow. However, at the end of the week if it shows that I haven’t consumed water, I’ve really gone overboard on something else then that’s a good appreciation like with the, not the happy face, but the down face. Or you know something visual to say to you know what, you’d better sort of up the ante or something. Just something simple, I mean it’s for our own selves really.

Participant 5: I’ve got this meditation app and you know you get stars each time you do consecutive days so yeah if I miss a day and I forget that day you can go back and pretend you did it. And I do that. So then I [unintelligible 01:03:26] the stars. So you know. I don’t have to start from the beginning again, zero stars. Well no it’s just because I forget that one day out of 200.

Facilitator: So at the end of the day you get some monetary reward?

Participant 5: No. People see that you’ve done consecutive days.

Participant 2: You’re being good. Yeah that’s a good case in point of your pressure.

Facilitator: So what do you think could help people about this application could improve user’s confidence when they track their diet?

Participant 1: Would it improve their confidence?

Facilitator: Yeah. What would help them that they would be confident enough to keep tracking?

Participant 1: Well again as I said, if there’s some sort of validation just to show the happy faces or at the end of the week, like on some of my apps, it calculates and it tells me like you know, how many steps, how many calories or how many work outs. I mean you know you’re not getting into heart rate and all that business, but just something to show that you’ve made a good effort. I mean you don’t want to complicate people’s lives here, but you want them to show that they’ve done their best and to show that you know that’s a great effort, to acknowledge it.

Participant 3: Yeah I’m big on acknowledging it. I guess so you see like iPhones do this every like week or something, like they show you your screen time per week. So yeah so you kind of have something like this for example. Oh so this week, if you want to really focus on Canada’s food guide you can. So this week you followed Canada’s food guide like 10 percent more and research shows that if you follow this it would have this many benefits for you, something like that.

Participant 1: That’s great, yeah I like it.

Participant 5: A weekly profile that you can compare where you’ve gone over and where you’re under to help you keep the balance.

Participant 1: Yeah you can graph it if you want, put it on a graph. There’s lots of different applications out there.

Participant 4: That’s it, it has to stand out. They should have had it already, we could have been using it for the Olympics.

Facilitator: So yeah for the interest of time I’m just going to ask one more question and the question is that yeah, in terms of accessibility so that the app would be accessible for all users for every kind of people with impairments, yeah. What do you suggest us to consider when developing the app? For example language or the audio or anything?

Participant 2: Well first of all I think you’ll have to do at least on the main mobile applications like IOS and Android and so on, but you also need to have it as a website for some because some people don’t just have a computer or have a computer and not necessarily a – And so it has to work on those different platforms. Language wise, well we saw with the governor general, make sure it’s in French too.

Participant 4: But just for visually impaired, I don’t know what [unintelligible 01:07:53] do you have audio? I’m not sure.

Participant 2: Yeah that’s a whole different app almost. That’d be an audio [unintelligible 01:08:08] app right.

Participant 5: I just have one thing I wanted to say, like when you’re traveling, whether it’s business or pleasure, there should be some way to let it know that you’re not cooking because you’re not [unintelligible 01:08:26] very different types of [unintelligible 01:08:32].

Participant 3: Great opinions. So for example I have this app to do list. It’s kind of like as it’s name implies, it’s a to do list and so you can have streaks so for example you will say my goal is to do five tasks every day. And then when you get your five you get streaks like for one day, two days. And then it has like a vacation mode.

So for example when you’re going on vacation you don’t necessarily want to do tasks and then you say this like I’m on vacation, don’t bother me like for the next week. And then like the next week you come back and you still have not lost your streaks and then you can like go on. So yeah that’s kind of based on what Lana was saying.

Facilitator: Yeah that was a great point, Lana. Thank you. And you were really thinking really hard about everything too. You were like oh my gosh, that’s a pickle.

Participant 4: And we didn’t really talk about promotion, do we care about promotion where it’s going to be advertised and that?

Facilitator: Oh that’s an interesting point. Wow, we never even thought about that either.

Participant 4: Dietician or nutritionist. Yeah.

Participant 5: You should be targeting students because they tend to get off the –

Participant 2: Yeah that’s true. What are you going to do with the data or is there going to be a central repository or what’s going to happen in the background? Like is this for research or is this going to be like just for the participants or like if you’re using the data, like -?

Facilitator: Yeah for now it’s just for research. And after we’ve tested the application and it was good to go out then we will, yeah people could use it for free and it’s for all general public, for everyone. And it’s not just specific for Canada. We hope to yeah it’s for all over the globe.

Participant 2: Sorry, but what I’m not clear on is OK so if I use, so once it’s accessible, if I start entering information, who has the information? Does it just stay on my phone or does it go to like a to you guys or how does it -?

Facilitator: For now definitely only on your side I think. Yeah otherwise, for example if Health Canada wants that data they would definitely let you know about it.

Participant 1: Are you going to be publishing this?

Facilitator: Yes.

Participant 1: And this is part of your PhD so you’re presenting this?

Facilitator: Yeah we’re going to test it in another setting. When we develop it, based on your opinions, and we have 40 experts, [unintelligible 01:11:51] registered dieticians that we have the same focus groups with them and we will be developing the app based on your and the registered dieticians perspective and once the app is developed we will test it and pilot it in the general population.

Facilitator 2: But once it’s piloted there’s no, I don’t think there is like a confirmed idea if it’s going to be used for research after that first pilot study. I think it’ll just be used for people to just track their diet based on the Canadian food guide.

Participant 3: I think like after like the pilot period has done like they’d have to come with a really strict ethics plan to be able to even publish it because I have some friends that when they want to publish their apps, especially on the app store for the iPhones like you have to really have a strong ethics behind your app. So like how are you going to store your data? Who has access to it? And everything else.

Participant 1: Exactly, yeah. Yeah that’s huge.

Facilitator: Yeah there is a lot for us to think about.

Participant 4: And just one more thing, to sign up for, perhaps you know we all signed up as individuals, but if you have a group sign up like a family of five that because they’re eating the same meals so don’t you have to constantly – Yeah and maybe that [13? 01:13:28] year old doesn’t want to use the app so the parent will or the sister or brother, you know.

Participant 3: Yeah good idea.

[End of recorded material]
