# Supplementary material for: Perceptions of self-monitoring dietary intake according to a plate-based approach: A qualitative study
Source: PLoS One. 2023 Nov 28;18(11):e0294652. doi: 10.1371/journal.pone.0294652 (PMC10683993; doi:10.1371/journal.pone.0294652)
Supplement: S4 Appendix — (ZIP) [file pone.0294652.s004.zip › Anonymized GP Transcripts/ICANPlate-GP-Focus-Group-2.docx]

**iCANPlate GP Focus Group**

[Start of recorded material]

Facilitator: This is iCANPlate Focus Group with members of the general public on July 27^th^ at 4:00 PM. \

This first part of the focus group will be about some behavioural [safeties 00:00:20] that we’ll be discussing about it further.

So first of all, let us know what makes it easy or hard for you to eat in accordance with the food guide? Just feel free to jump in and make a discussion.

Male 1: I don’t like vegetables, generally, so that’s my main problem.

Female 4: I like sugar and I like meat.

Female 2: I would say time constraints in terms of the recommendation to cook at home, that makes it hard when you don’t have much time.

Female 3: Yeah, I feel you have a lot of stuff that you have to eat to be healthy, and often I'm just going to prepare some protein; I'm just going to have a salmon and then some potatoes next to it. But I don’t have time to eat nuts or have really the full plate.

Male 2: For me, there’s sometimes difficulty if you want to have plenty of vegetables and fruits, depending on how you store them. Well, generally, in the store they're not ripe, so you don’t have the nutritional value, and then after they seem to ripen overnight, and you can't consume them throughout the week. So, I mean, if you want to fulfil this, either you get access to already ripe fruits, or you have to do your groceries pretty much every day, which is itself time-consuming, depending on where you live or how accessible the stores are – I mean, good food stores.

You may not need just to go to do it.

Facilitator: Okay, any other thoughts?

Female 4: I would say, I mean, I’m lucky enough that this doesn’t really apply to me anymore, but healthier foods are more expensive. And there are times when I look at my food purchase expenses at the end of the month, and it’s a little bit shocking. Not everybody can afford it; it’s a lot cheaper to go and buy a box of mac and cheese than it is to buy vegetables and fruits every day of the week.

Female 3: And also, some people can have allergies, they cannot eat gluten as well, so they also have some limitation on that side. Also, people who are vegan or vegetarian, maybe if they're just starting vegan or vegetarian, maybe they're not educated enough to know what other sources of protein they have.

And – yeah, that’s it.

Male 3: Well, I have a phobia that I’ve had since childhood; I cannot eat food from water – fish. My whole system reacts when I'm presented with that kind of stuff.

The other food I dislike intensely is beans, like, Lima beans, stuff like that. And I'm sure that dislike came from the poor presentations I was given growing up with the beans. But that’s another matter.

However, I don’t mind saying I'm a carnivore, so I get lots of protein through meat, and that seems to satisfy that name. The other thing I struggle with is the huge quantity of misinformation out there concerning what is good and what isn’t good for you anymore.

And the other component of that item is that I have the old Canada Food Nutrition book from 1951, and I keep hearing that the food that was measured and tested back then is not the same food we have today. That the contents that we’re getting out of the fields and out of the animals are considerably different, because people have been manipulating them in the past seventy-odd years to improve growth, to improve quantities, to improve profitability and things like that.

So that concerns me, because that’s taking nutrients out of our food supply that we need.

Facilitator: Oh, it sounds great. So, the next question would be, if you want to make changes in your diet, what techniques would you use to monitor yourself that you're sticking to those changes? Any techniques that you could think of?

Female 4: I use a food log. I use MyFitnessPal specifically. I find that it has – I’ve only tried that one and Noom, and I found that MyFitnessPal has a significantly more thorough, more accurate database to choose from. I can put in the name of the product, and if it’s a shelf product, it has the exact nutrients already in the system.

And it really makes you think twice before you choose to eat something, because you have to go through the trouble of logging it. It’s definitely more strenuous when you're making recipes for yourself, but it also has the function to add in your own recipe.

So, for me, as long as there’s the ease of entering things, a food log is the best way. But if it gets too strenuous, a food log is – I wouldn’t want to do this on paper, let’s say like that.

Male 2: Is it MyFitness – sorry, that’s a question for [Shai] – is it MyFitnessPal where you scan the barcode, and it tells everything in there?

Female 4: That’s one of the functions it has, yeah.

Male 3: Really?

Male 2: Yeah, so I’ve used that in the past. I feel, like, making lists is maybe something more efficient, both for the budget and for – I usually go – you know what I'm doing good, which is horrible, because now I want to buy the whole store – which also leads to bad choices, like, too many veggies and so on.

So, making lists – and my mum used to do this, actually making a list of plates and meals, because she had to plan the family, and that would be a good one also. What’s helpful is probably, make changes would be to [unintelligible 00:07:15], like, I can afford that – a day to cook and then freeze the meals, because then I would just [unintelligible words 00:07:23] the time today.

Facilitator: Any other techniques? Yeah, like, go for it.

Male 3: I have ADD and logging is not my favourite subject. [Laughs]

Facilitator: So how would you try to stick to the changes that you want to make in your diet? Would you, for example, set your own goals, or yeah, what other techniques, if you do anything else?

Male 3: I try to construct menus of foods that I like and give myself enough choices of different meals so that I have a couple of choices at each meal, and generally stick rigorously to that sort of set of menus, if you want to call them that.

Facilitator: That’s a really interesting idea.

Male 1: I make lists, like the other people said, and I also make a point of not going shopping when I'm hungry, because if I do that, I’ll just [unintelligible 00:08:33].

Male 3: [Laughs]

Facilitator: That’s a good idea. Okay, so Shai already mentioned that she’s using MyFitnessPal, and what other applications have you used, you yourself, personally used, and what was hard or easy for you to work with those applications?

Male 3: You know, twenty-five years ago, I got hold of one of the early versions of the Food Database from the government of Canada. I can't remember if it was from Health Canada or from Food and Agriculture, but I put it all into an Access database and tried to do a front-end where I could log my food.

And after a few weeks it got rathe complicated, because you also have quantities, and you have quantities in different systems of measurement, and it just became a terrific burden to try and get this thing to work easily, that someone would use it as a logging tool.

But the advantage, if I ever had it working, was that it would give you all your nutrients for every meal or every day, as you wish. And you could see where your shortcomings were and what you needed to make up.

But I think anybody that decides they want to build this kind of product, wow, good luck to them.

Facilitator: Yeah. Anybody else, have you used any other applications? Yeah, go for it.

Male 2: I use Yuca 00:10:29] as a healthy – I don’t know if yuca – there is a [Carrot 00:10:35], it’s an app on [unintelligible words 00:10:36], mainly developing your app, but after, it moved recently into Canada. It’s basically, you scan a barcode, and it tells you which kind of additives you have, like atrazine pesticides and so on. And then you can make supposedly healthy habits, which is somehow good, because they also have the nutritional value.

And also, it starts to be scary, because then after you have – it’s like a [dirty 00:11:02] partner, and you scan everything that people – the guys can just scan everything, and they decided that sort of freedom is early taken away with the app.

Female 3: I’ve also used – it’s called Quinoa, where you also scan the food. It’s way easier when you can scan and they actually have it in their database, compared to when you have to write it yourself, how much do you eat. Because often, personally, I don’t know. Like, just a handful of almonds, or something like that. But I don’t know in grams how much it is.

And yeah, so I think that was the only one, but I only used it for four days for another study. Initially it was fine, I could keep track pretty easily and I wouldn’t mind. But then towards the end, it took more motivation to actually keep track of those, because it was, like, I was just starting to become lazy to do it.

And also, I feel like it helped me a lot to keep track of my diet, so I can lose weight. So, because I was too lazy to put the food in the app, so therefore I was, like, “Well, I'm just not going to eat that chocolate, because I don’t want to enter it.”

So, in that specific situation, it helped me, but I don’t think I would be able to do it for too long.

Facilitator: That’s a good idea.

Male 1: I haven’t used any apps, but I did another study a few months ago where they were doing paper-based logging. But instead of detailed logs and everything, you ate – it was just, like, a circle, like, a plate, and you divided the app into slices of how much grains, how much protein, how much vegetables. If found that simple enough that I could keep doing it.

Facilitator: So, it was basically like the Canada Food Guide that I just showed to you, right?

Male 1: Yeah.

Female 2: Sorry, just curious, just wanted to ask, was that the study with Celeste Bouchard?

Male 1: Yes, I think it was.

Facilitator 2: Oh yes, okay, we’re familiar with that. Good to know. Thank you so much for letting us know.

Female 2: I’ve used Recovery Record. That one was recommended, because it more intuitive, it doesn’t really have anything to do with calories or anything like that. And it focused more on how mindful you are when you eat, who you're eating with, you know, how you're feeling after the meal. Are you feeling overly full, or just fine?

You can also take a picture, which I like, of your meal. Yeah, so it just kind of has other contextual factors to consider in that app, other than just what did you eat and what were the calories.

Facilitator: Oh, it sounds really interesting. So almost everyone has tried, is quite familiar with these applications, that it’s really great for us. It’s super-helpful.

So, in this part, we’re going to show you – so before we go further, do you know of any diet tracking applications that currently mirror the Canada Food Guide that I just showed you, like, having five fruits and vegetables. Okay, I can see heads shaking –

Several: No.

Facilitator: Okay, great. In this part, we’re going to – so first of all let me know how would view working with the app, working to record all the meals throughout a day?

Female 3: I think it’s going to be easy to use, but you might lack a little bit of – like, the exact quantity that you're eating out of each food. And also, often you have, for example, pasta, you have carbs, you have veggies, you have cheese, so it’s all mixed up together. I'm not exactly sure how I’ll be able to know the exact quantities.

I guess it also depends on what’s the goal of the app. Like, do you want to diet, do you want to count your calories, do you want to just make sure that you're having a bit of everything, or does it have to be really precise or not, in my opinion.

Male 2: I kind of agree with you, yeah, because if you have to [unintelligible 00:15:56] guesstimate the proportions, and if I'm using the example of pasta and veggies, so would an app count the grams of pasta and the grams of veggies? And then you just select?

It might be – I don’t see from that little demo how you're going to guesstimate what you're about to guesstimate. Is it the calories or is it the weight that you're entering, or estimating in the app?

Facilitator: It’s basically just how you would think that your plate or your meal you're having, what proportion for each category of the food that would have. Like, my plate has a quarter vegetables, and you just put a quarter – just, like, a quarter of the green colour for the vegetables and fruit.

And yeah, it is not important, the quantity and the weight of the food that you're consuming, or the colour even.

Female 2: Yeah, just to add on, I'm pretty sure this Canadian Food Guide doesn’t focus on the amount of calories you eat and what amount of food those calories are made of.

So, it pretty much just focuses on what proportion of what you specifically eat in that meal or that day, is actually how much of what you eat personally is vegetables, what you eat is protein and what you eat is grains.

Male 2: I'm sorry, I’m still confused. You know, what proportion? And then there is a unit [unintelligible 00:17:44]. Is it, like, silly example, counting pastas: I have two hundred pasta and four carrots. So that would be two percent carrots and the rest is pasta, if that’s [unintelligible 00:18:00].

I understand the calories, that’s not where we want to go, because that may be overwhelming. So, it’s a guesstimate according to the weight. I don’t have to measure it, but then it’s an indication if you say a quarter [unintelligible 00:18:15]. Is it visual, is it – that’s what I'm confused about.

Facilitator: Yes, I think it’s mostly visual. Yeah, you should just visualise what you're eating, and yeah, put it in the app.

Male 1: Yeah, I did this for a week on paper, like, I said, and it works really well for meals where you have a bunch of different things on the plate. But, like somebody else said, if you're eating a stew or a bowl of pasta or something, it’s harder to figure out, because it’s all in one pot.

Female 3: And I guess it also depends what type of food you eat. For example, I'm Romanian and we eat a lot of soups with a bunch of stuff in it, and at least fifty percent of the soup is water or broth. So, I'm not sure how I’ll be able to say how much of my soup contains what type of food.

Facilitator: Yeah, any mixed dishes, yeah, it’s really hard to track them. Yeah, any other ideas? Audrey, do you want to add anything?

Female 2: I would say I agree with what everyone said about, it’s hard to kind of visually track something. Like, if you're having a burger or something like that, or a stew or something, it’s a bit harder to input that. But I think generally, the concept is nice, because it’s more about patterns, I think, over the long-term, and just kind of getting close to that, you know, what’s on your plate, those proportions, versus not having to be super-exact with it every single time you log it.

I think that’s kind of nice, because then it gets easy to get very sucked into numbers. Yeah.

Male 3: I still think you need the weight.

Facilitator 2: Yeah, I guess a lot of people are used to thinking about weights, because it’s easier to log it, so you actually know how much of your food was actually vegetables or protein, which makes sense, for sure. We’ll keep that in mind. Thank you.

Male 3: Well, if you have the weight, you can also do a much better analysis of the different nutrients they might be getting, based on the proportions of the three different food types.

It’s a starter, but I don’t think it’s an answer.

Female 2: I think it will depend on what the goal of it is, because I think for the general population, you kind of have to be careful about what – I don’t know, if the goal was intuitive eating, let’s say, and you're just trying to kind of approach this from a gentle nutrition perspective, then you don’t want people to rely on external cues, such as numbers, weights, calories and stuff. You focus more on kind of, what do I want to add into my diet, versus, what do I want to take out, if that makes sense?

Male 3: Yeah.

Female 2: So, having that visual plate, I don’t know, personally I'm a fan of it. But I do think it needs to be tweaked a little bit.

Male 2: Yeah, a cultural difference; I'm from a European background, like Julia, and when we do prepare a recipe, everything is in gram. When you go in North America, it’s in oz, spoons, teaspoons, coffee – which is a nightmare when you try to cook and follow a recipe.

And that’s fun, that’s part of, you know, [turning 00:22:07] this side of the ocean. I really like the fact, like Audrey had said, it’s not to be over-complicated, that’s for sure. What we evaluate, maybe examples or videos could be useful, like your typical plate that you guesstimate a quarter veggies and so on, would really help people to see from ten different plates, what does it mean a half of veggies, what does it mean a quarter of grains?

In terms of a pudding, they both can feel familiar on how to handle that.

Facilitator: Wow, those are really great opinions for us, really great. So how would you represent the different meal portions on the plate? How do you envision tracking the portions?

Female 4: I use a scale and I use grams, just because it’s – sorry, I know we’re trying to move away from that, [laughs] but it’s the most accurate. If we’re going back to the comment about – Frederick’s comment about the recipes, I always look for recipes that use grams as opposed to recipes that use cups, because they’ll always tell you when you're baking, you're better off using grams, because you're not going to get a precise measurement with a measuring spoon.

And I can tell you that I have two different measuring tablespoons, and they're not the same size. And these things are supposed to be uniform. So, my confidence in saying, “Oh, this is a cup of [unintelligible 00:24:00] or whatever food, what are we using as a cup?” I can tell you that my mother refuses to use measuring cups, and to her a cup is whatever cup she’s using. So, it’s very subjective.

Male 1: My mother used a palm-full, a pinch, you know, a handful, you know, those old – the grams she got from her grandmother.

Male 2: And that’s the best recipes, right. I think the [unintelligible 00:24:36] represent the food on the plates, maybe I could think, you know, if we have the pasta or paella or soup, then maybe I could see as a two-step, because the one very simple presentation could be uniform.

But if you said, like, I have a salad, like, a pasta salad or a rice salad, you know, having those proportions and you can adjust them, and maybe the amount of veggies could be bigger than the rice, you know, to feel a bit – to give a visual clue to people about how it would look.

Like, several steps; you know, you could have three or four images when you add veggies of a salad with little amount of veggies, medium amount of veggies, high amount of veggies [unintelligible words 00:25:28] input, that could help.

Male 3: Maybe you should include water as one of the segments.

Facilitator: Yeah, we’re going to talk about water as well. Yeah, they're even – it is really important to think of water, and we’re definitely going to ask about it later.

Male 3: Well, I'm thinking of the soup recipe, you see, because then you can –

Facilitator: Oh, I see.

Male 2: Yeah, if you have a soup, that’s a great idea from [Phillipe]. If you have a soup then the app, instead of asking three, it could ask four, what’s the amount of water you added?

Male 3: Mm-hmm.

Male 2: And one litre, or three ounces, or twelve ounces of water, which is your preference. But that could be interesting to see, okay, if you have that amount of veggies and two litres of soup, the nutrients will be – and if you take a cup of it, it gives you less as – you know, if you have those videos and [unintelligible 00:26:36].

Facilitator: Any other –

Female 3: Sorry, if I have to use this app for whatever reason, I think I’ll just think ahead and plan my meals so that they will be most simple meals that I can have. For example, I’ll have three potatoes and – okay, maybe not three potatoes, but just for an example – and a part of salmon, for example, and then that’s it.

And I will maybe not make a sauce that has a lot of veggies in it and a lot of cream, so I know, because I want to be able to track that, and I will not feel comfortable to track it in the app. So basically, I would just think more about what I eat.

Male 3: That’s always positive.

Facilitator: So just – Julia mentioned that there would be some sauce on the salad. So how do you think those foods – those foods that are not included in the guide, could be shown on the app? Like, sauces or – yeah, cream cheese. Yeah, there are many such things that are not included in this guide.

How could we show it on the application?

Male 2: Could you have something like options, where you select your plate, whether it’s salad, and if you have a sauce, you could have homemade vinegar or seasoning, sorry. Or then you could have light purchased seasoning or regular seasoning, maybe something around those policies.

Like, you ordered – ate part of a pizza, so there is all pizza, and then after the options, extra onions, whatever. And then you could have those extra options, so you don’t feel that you have to evaluate how many you used.

But then it could raise the question on what you're thinking, I’m purchasing my salad sauce or my ketchup or mustard, and you know, mustard has generally less high calorie count or impact than some industrial salad seasonings. That could be a way to think about it then.

Female 3: Having a database with all the common products that you have in Canada, like, all the sauces, yeah, all the [really good 00:29:35] products are – what’s it called again? The stuff that you buy that, it’s ready-prepared – I forget the exact word.

Male 2: The processed food?

Female 3: Yeah, exactly. So, if you have a database of all that stuff, then you can just count it.

But another thing that I just thought about right now, which is a bit unrelated to this topic, but I just wanted to mention, what if people don’t know what product is, for example, a protein or carbohydrates, or any type of – like, how can you know that people are actually educated enough to know which thing goes in which place?

Facilitator: Well, I think what Frederick was mentioning, was that we first write it – for example, we could just search the meal that we’re having, and then the app would show us some choices, right. Am I right, Frederick? You could jump in here if I’m wrong?

Male 2: Yeah, that could be – that’s an idea to get people get to [unintelligible 00:30:48].

Facilitator: And it would be like a reversed plate, right. Yeah, we would just tell the app, and the app would say how many [unintelligible 00:31:00] portions that we’re consuming.

Female 3: Okay.

Facilitator: That’s a good idea.

Female 4: I agree with Julia, there’s a potential issue here in terms of people’s education level, and I think this is often a problem that we see with study groups and focus groups, because they tend to be kind of skewed towards people with higher education.

I mean, I could tell you that I received this through the fact that I'm a Concordia alumni, so that already biases me towards somebody who has a background in a more science-y field than – I'm very comfortable with the terminology when you were saying, oh, yeah, foods, vegetables, carbs, proteins, whatever, I don’t think about it twice. That’s not necessarily the case for everyone.

So even if you give sample plates and they show you, well, okay, chicken is a protein, that doesn’t necessarily mean that people are going to understand, oh, a lamb chop is also protein. Because to some people, well, chicken and lamb are not the same thing.

Facilitator: Yeah, so how about the food, the [unintelligible 00:32:10] food in the guide? Do you have any idea? I’d like some other food, like, you know, some cake or muffins that can be counted as grains, that are not really healthful, and they cannot be counted as grains in the plate?

So how would you think that they could be tracked?

Male 3: I can't imagine a food that’s not in the guide. Blubber? Whale blubber?

Facilitator: Yeah, right.

Female 4: Things you love to eat section.

Male 3: Oh yeah, all right. [laughs]

Facilitator: A good example would be even vitamin supplements, or even added sweeteners, like maple syrup or honey. How would you classify those types of foods, which are not exactly in a protein or a grain food group?

Male 2: But isn’t the purpose of the app to track this – I'm not sure, [unintelligible 00:33:19] we’re just going to – so those are kind of supplements. The same way, if you have honey on bread, but then I put only my seasoning, then how am I going to track it?

Facilitator: Yeah, you could maybe tell us how.

Male 2: No, I would track it by a spoon this way, how many spoons I would use. Again, I don’t think, when I just look at the same time I have, you know, the Canada Food Guide main page, and it’s really, really simple, because there are simple ingredients. So, I'm not sure on how deep we want to track good and what we [unintelligible words 00:34:06].

Because there’s the balance between it’s precise, but then it takes a lot of time to coordinate it, and it’s too simple, and then there’s a lot of information you may want to have, and you don’t know how to find it.

The same thing, kimchi. This is excellent; spicy, most like veggies, but there are so many different kimchis. So fermented food, you know, there is kimchi in Eastern Europe [unintelligible 00:34:39] we made, so are the [unintelligible 00:34:41] through decades and since [unintelligible 00:34:44].

So maybe your statement doesn’t go over – it’s not a calorie checking out, like, Shai mentioned before.

Male 1: For me personally, I'm not interested in that kind of detail. I'm interested in trying to force myself to eat more vegetables. And if I can see a plate of what I ate and see there’s half vegetables, then that makes me feel good.

Male 2: Yeah, it’s a very good point. If your app is overall [unintelligible 00:35:19], but I think, let’s say I eat a third of veggies and wholegrains, but then often missing the protein and then I can say, like, “It seems that you might benefit from this.”

So, you can have your trends and maybe the veggies should be in the green, commonly referred as a good thing. But if you don’t like veggies, because they're green, you know, the colours –

Facilitator: Yeah, that’s a good point. [Laughs]

Male 4: I think it’s also important to be careful – I think it’s a mistake that a lot of people make where, okay, let’s say I have my plate and I see that I ate perfectly, I ate half veggies, I ate a quarter grains and I ate a quarter protein. But then also, I had a lot of this unclassified stuff. I had half a chocolate cake.

So visually it looks to me like I did very well, but I think that there should be this other category that’s inserted into the plate to show you that actually, if you then ate half a chocolate cake, your fruit and veggies don’t count for as much, because they're not actually half of your plate, they're only a quarter of your plate, because the plate has to accommodate for this other category.

And it could just be a giant miscellaneous category, where you really throw anything that you're not sure about into. You err on the side of not designating something as a fruit and veg if you're not sure that it is. But I think that might give a better, a more accurate perception of the percentages, the proportions that you're eating, the conditions of the Canadian Food Guide.

Male 1: Yeah, you could show a second, smaller plate, like, a dessert plate, for putting that stuff on.

Male 3: All sweet stuff and carbohydrates that you shouldn’t eat, like honey, sugar, chocolate bars.

Male 2: Shai has a very good comment here. The Canada Food Guide’s cover only mentions what looks like a main dish, and what about if you have [ekgha 00:37:40] – you know, cod ekgha just before having veggies, how much saturated fat will you intake? That’s awesome, that’s good, you know.

Or arancini, which are very simple truffles, but they're fried. It’s grain, but then it’s fried, so they show a main course, and what about what you eat around it? You have veggies, but you get your two wraps oof processed food, and then a muffin that you had, which is then your whole food [unintelligible 00:38:18].

Facilitator: Yeah, interesting. Audrey, do you have any other ideas how to – do you think, is it necessary to track all these other foods on this plate method?

Male 3: Oh yeah, oh yeah, that’s where the carbs come in, the real bad stuff. The chocolate cake, the honey, the maple syrup. That would be a fruit and vegetable, wouldn’t it, maple syrup?

And what about milk?

Facilitator: Yeah, we’re going to talk about it, Eric, yeah, after –

Male 3: Because the Food Guide says you shouldn’t have it at all.

Female 4: Yeah, it’s a strong movement away from when we were kids and the whole Got Milk? campaign was everywhere.

Male 3: My wife took one look at that line and said, “Well, there goes that guide.”

Facilitator: [Laughs] So let’s go first, talk about beverages other than milk, like, water, sugar-sweetened beverages, juice, coffee, tea, anything. How do you think that beverages could be included in this plate?

Female 4: I’s hard to say, because you also have to understand the nutritional value of whatever you're consuming. Because water is neutral, we know that water is neutral. If you are drinking a sweet drink, whether it’s a fresh orange juice, which is better for you, which would technically count as a fruit and vegetable, or if you're having a Coke, well, that needs to go in that other category that we discussed, because that’s basically pure sugar and chemicals.

Then you also have the question of artificially sweetened beverages, and I mean, until recently I was a mass consumer of Coke Zero. I mean, I just really like it. And then I read a study that came out of Yale a little while back that said that when you mix an artificially sweetened beverage with a calorie-laden meal, you actually confuse your brain and your body doesn’t properly process the calories of what you ate, because the artificial stuff from the drink made it think that it’s not getting calories.

So that just throws everything completely awry, because then it doesn’t matter what you put in your plate, because your brain and your body chemicals are all missed up and your insulin’s going crazy.

So, I think that putting them under just beverages is over-simplifying what they are. You need to really go into, is it a sugar, is it a fruit or veggie drink, is it water, is it dairy, etcetera.

Male 1: Is it alcohol?

Male 3: You should have a segment for manufactured chemicals that we eat. I remember the very first, they called it [mardrant 00:41:40].

Facilitator: Yeah, any other ideas how to show the beverages?

Male 2: You know, I don’t see how you envision the app, but let’s add a meal and then it asks about food, and then it slides – do you drink something? And then you select those categories where it’s water, milk, alcohol, soft drink and soft drink with artificial sweeteners. And then it helps a little bit.

And then a guesstimate of the quantity would be nice.

Female 3: But if you have an orange juice, is that going to count as your extra fruit portion, or should it count as a drink? That’s also something that you need to ask yourself, I guess.

Female 4: And not all orange juices are made equal; you’ve got the ones from concentrate, you’ve got the pure ones.

Male 2: Is that also the orange juice you remove all the fibres, because you’ve got the juice, the rest – the good thing – stayed on the skin. But then it’s not equivalent.

And I think the good thing is, if you ask about what you're drinking and that’s fresh fruit juice, versus – I don’t know how to call it, because made from concentrate is not the same. Or fruit juice, no sugar added, and other fruit juice, maybe, would be a [unintelligible 00:43:27].

Facilitator: Another question: how do you think we could show the beverages, you know, like, how they would track them? Should there be another plate to track the beverages, or should it be, for example, a standard glass, or yeah, how do you think it should work?

Male 2: So, like, the simpler, the better. A glass, yeah, and guesstimate of maybe a bracket of how many mils you would take. And then maybe the app can let you select how you guesstimate the beverages. Because I go with millilitres, but if you want to go with other units that are more familiar, everyone is at ease.

And then I don’t think we have to go over, is it fat-free, is it three point twenty-five, is it the high [unintelligible 00:44:28]? That would not be – I don’t think that is the tracking, just a simple volume reading.

Female 2: I think if you're going for something visual, it could be good to have illustrations of cups, but different categories. You can have fruit juices, soft drinks, whatever, all of them, and you can kind of maybe drag them into a section where – or select and then that shows up in a beverage section for the day. You can visually see how many cups of whatever beverages you actually consumed.

I don’t know if that makes sense. I'm just picturing it in my mind.

Male 2: Also, a recap of the idea [unintelligible words 00:45:14], hey.

Female 2: It would be, like, either at the end of the day, or you could log it throughout, but at the end of the day you’d have, let’s say, a little visual cup of coffee, and then right next to it, if you had three cups of water, you’d have that many. And each cup would look different, based on what – or maybe would be labelled, based on what you actually had to drink.

Male 2: Maybe with some key part, [unintelligible 00:45:45] part, you could select the tea, the caffeine, the latte, because it’s the different –. And you know, a glass on the [unintelligible 00:45:54] and juice. The visual is a lot simpler, so you can get it and it can be very helpful, visually it’s going to engage –[unintelligible words 00:46:06].

Facilitator: Okay, interesting. Any other elements of the Canada Food Guide that you remember, that should be included in the app?

Male 2: Would the app have a direct link to the recipes? Because the guide has access to some recipes. But if you have, you know, your food guide, maybe you [name 00:46:37] some [unintelligible 00:46:39], some – I mean, you select what you want to add, like, add a meal, add a drink, get access to recipes, tip of the day. It would be interesting to have that, yeah, because the recipe helps you, definitely.

Facilitator: Yeah, and do you think any specific recipes should be there? How about international foods or –?

Female 4: That’s hard, because as you can tell probably from my name, I'm also not originally from Canada, and there are certain things that I eat that are probably not going to be found in the Canada Food Guide, because it’s just not food that’s eaten this side of the world.

But that doesn’t make it any less nutritionally valuable. It’s just a that if we’re going by easy-to-use categories, it’s less likely to be found in there. To say, “Oh, can we offer recipes that really are applicable to everybody,” trying to please everyone, I think, is a little ambitious.

What I was saying before about MyFitnessPal, what’s so great about it is that users can add to it, which is what allows it to grow and be so applicable. So, if there’s that function, where somebody who is from the Middle East and eats tabouli every day, can put that in and then that’s accessible to everybody else.

Oh, what is tabouli? Oh, somebody took the time to figure out that it’s this much grains, this much vegetables, and you kind of have that information.

Male 2: It’s like you shared your recipe, kind of stuff.

Facilitator: Right, it’s really an interesting idea. How about other elements on the backside of the guide, like the mindful eating? Do you think we should put it in the app as well?

I know that Audrey is using an intuitive application. Do you think we should put it in there?

Male 3: Sorry, what is that?

Female 2: I think so.

Facilitator: What other elements, like, mindful eating, like intuitive eating, or being mindful of what you are eating, these are some other elements that the guide has suggested.

Female 4: Or even eating with other people as well, like, you know, having as a social thing, like, when you go out to eat with friends at a restaurant.

Facilitator: Yeah.

Female 2: I think it would be great as an optional thing for people if they want to – depending on what their goal is. Maybe they want to focus on, I don’t know, the link between their mood and what they're eating throughout the day.

And you can have one question just asking how they're feeling before the meal, let’s say, and then after the meal, you know, are they feeling full, satisfied? Maybe not satisfied, I don’t know, just different options for that, I think, would be helpful.

Male 2: I like the idea of the notifications really a lot, because they're – so I see seven categories on the back of the food guide. If I had a recommendation, it would be to give a lot, maybe five to ten per item, so you don’t have the feeling to every three days see the same one. Because then after, if you see the same one, you know, if you can see a pattern, then you just turn off notifications, then you lose that ability for good tips.

Facilitator: So, you’d suggest that tracking moods and feeling could be shown up by a notification?

Male 2: Mm-hmm.

Facilitator: Great.

Female 3: I think that would be good if the goal is to do some research on it to see how people feel before, after and such, like, in what context they eat. However, when I used the Quinoa app, which had those types of questions, it didn’t really affect my behaviour when I eat. Like, they would ask you what’s the reason why you're eating; is it because you're hungry, is it because you're bored?

Well, even if I was bored, it didn’t affect me, it didn’t stop me from eating that food, for example. So again, I guess it depends on what’s your goal in having those types of questions.

Facilitator: I think you tried the Quinoa app just for a short time. Do you think if it was a longer time you were trying the app, and you could find a trend about your moods when you were eating, do you think that could affect how much you're going to eat, or which foods you're going to eat?

Female 3: That’s a good question. I think I would be able to find a trend, but I don’t think I’d be motivated enough to stop eating fast food when I'm bored, let’s say, or when I go out with my sister, or so.

I don’t know if it’s going to help me, but maybe it’s going to help me to kind of realise, okay, here’s the data, here’s the proof that I'm eating when I'm bored, let’s say. So maybe that’s the first step to help me stop, let’s say.

Facilitator: Good point. Getting back to when you were talking about the recipes. Do you think – which other instructions or support should be provided in the app when the users are tracking their diet?

Female 3: You can have food groups to see what type of food you eat, where does it go, is it healthy, is it not healthy? So it can better help you to fill out the proportions. Maybe that’s going to help.

Female 4: I think it would be important to have resources just directing people, if this kind of triggers anything in them or makes them struggle with food. I know that as soon as you try to monitor food, if you have any sort of eating disorder or obsessive behaviour, this can really trigger some things.

I know that, for example, it’s very common now in television shows and all that stuff, if an episode deals with an eating disorder, at the end they have a contact number, saying, “If you're struggling, please call this number and you can get some help.”

I think having those kind of professional resources, just having those links out there, I need some help, I'm struggling with how I feel about how I'm eating, I think that could be really helpful.

Facilitator: Oh, that’s a really good point. I’ve never thought about it. Yeah, that’s great.

Male 1: Some kind of trend assessment would be good too, like, to have the app tell me or you, “You ate too much meat last week, maybe you should eat more vegetables this week,” so that kind of thing.

Facilitator: Like, notifications? It should be an in-app notification, or –?

Male 1: Yep, like, “You're eating too much ice cream. Cut back on that,” or something.

Male 2: I think Audrey or Julia mentioned the recap, some stats on your profile as well. Maybe that [unintelligible words 00:55:01] mark the – [unintelligible words 00:55:03]. Then just entering without being able to see it – maybe export. I don’t know what kind of a – if there is an online, like, from apps you can go online and you have those graphs, maybe more manageable than within an app, I don’t know. But the tracking would be interesting.

Facilitator: Yeah, so do you think – what features can you think about that could help people to speak to – you know, just to keep tracking, to speak to using the application? Like, maybe some competitions in the app. Yeah, what would engage them?

Male 1: I have a Google Assistant and I use it quite a lot, so it would be nice to just be able to say, “Hey, Google, I'm having a cup of tea,” and have that recorded without having to go into the app and fill stuff out.

Male 2: There’s a widget. A widget, you know, if you slide sometimes on the phones, they could [unintelligible words 00:56:28]. If you slide on the other side, you have shortcuts. It happens that this is WhatsApp and they have four.

But you could, say, just slide, add a beverage to a food and meal, then you don’t necessarily have to open your phone and the app, load the meal, like, premature [unintelligible words 00:56:48]. It would be interesting.

And also, your regular food, you know, when you add a beverage. I drink a lot of latte, which is bad, because it’s too much coffee and too much milk, but if the app learns that I add a latte, then it’s just like, boom, you have your favourite shortcuts, being [unintelligible words 00:57:12].

Female 3: I guess – and I think that doesn’t take too long to fill in. And also, maybe you can keep track of every food category that you eat. And let’s say, for the day or for the week, if you are ready, when beyond the limit of carbs that you're supposed to have for the day, maybe have a notification to say, “Well, maybe you should slow down, or maybe you should eat more fruits, because you had one portion for the week”, or something like that.

Facilitator: What other features – yeah, yes?

Male 2: Would the app be able to give advice on when to eat what? Because usually, you know, it happens that when I'm working, I just take short meals at lunch. There’s no time; I'm eating in front of the computer.

But then after, at the end of the day when I'm back, I feel I need to treat myself, so sometimes my biggest meal is in the evening, which is, again, awful, because then you just sleep a couple of hours after and then you store all that instead of using actively the carbs or whatever source of energy, throughout the day.

Facilitator: Oh, so setting reminders on when to eat your meals like this then?

Male 2: Yeah, like, there’s an app when to drink, and it pops up, like, it’s time for your water. But back in the day in the lab, I was starting at nine and sometimes an experiment would run until 3:00 PM, so at twelve I was hungry, but now it’s 3:00 PM, no, not hungry anymore. Then I would just take a coffee in the morning, and my biggest lunch in the evening, which is probably where my bad habits started.

Facilitator: Yes, how about – do you think if there would be competitions or games or peer support in the application, it could help people keep tracking?

Male 2: I think it would be good if you link it also to a group or a Facebook group or a [unintelligible 00:59:40] group. Because then sometimes it’s good, like, achievement or – at the end of the day that would be nice to be able to connect with friends, because maybe, you know, we’re starting to know each other here in this group, and that’s nice. But if I have random, names, you know, if I would see – let’s begin with the first one – Patricia has invited you to a challenge, you know, it’s like, well, I don’t know Patricia.

That said, if my neighbour is, like, “Hey,” and he said, “hey, go for it,” Interesting to challenge your own friend.

Facilitator: Do you think – so you're basically mentioning that an in-app support group would be more helpful than linking the app with other social medias?

Male 2: I think it would be hard to manage if it’s inside the app, unless it’s another added storyline. Many other apps have added that add-to-your-story. But if you can share maybe on those main social media, or if you can – there are some apps you can – and maybe some running apps you can have a small community that is booking in and saying, “Get a challenge to your friend.”

It will be both, like, you can invite them to use the app, and you can challenge them as well. The challenge should be inside, then the whole community supports and makes changes, it could be difficult to have this inside the app.

Female 4: It is always the concern also, as soon as you integrate anything into the app, you take responsibility for it. And I think you're the one who commented before, “Oh, would it be moderated?” Because you know, you leave people on the internet and the worst comes out.

Male 2: Exactly, yeah. I said to moderate and share your recipe, like, that’s basically [unintelligible 01:01:41].

Female 4: Yeah. [Laughs]

Facilitator: Right. So, what features could you think of that could improve users’ confidence when they're using the app? Yeah, when [unintelligible words 01:02:07], they would be more confident?

Male 2: A clear statement that they have the option to not have – I don’t know if it’s Apple Health or Google Activity, or you know, a clear thing, like, they're in control of what they share with anyone. And they're [unintelligible 01:02:29] of privacy, and that’s, I think – and that would be open, like, you can choose if you want to share. If you want to have anonymised data that the team could use to evaluate the app at the beta testing.

But then, if you want to completely have a standalone, that would be nice. Also not having the option to create an account, would be nice as well, because then, if you create an account, you feel like, if I'm using my Gmail on this app, and then I'm using Gmail on MyFitnessPal, then Gmail can make the connection on both of them. And then I feel like I lose control of my –.

I don’t know, that’s a little bit – to facilitate that, because you mentioned confidence.

Facilitator: Yeah, anyone want to add to how to improve confidence?

Male 1: I agree that being able to use the app without creating an account is important for people – people who are worried about their privacy.

Female 4: I agree with that, except that then some of the other features we discussed would not be available if you don’t – well, again, I don’t think, because for example, sending you notifications or keeping track of all the meals that you repeatedly put in, I think that would be very difficult, on a practical level, to do if you don’t have an account.

Male 3: Yeah, if you're going to have an account then you should make it available through products like Windows and Edge, as well as apps on Android and iPhone. And maybe even a Windows app, do they still exist on mobile? [Laughs]

Male 2: There’s Windows still, right.

Male 3: I had to dump a Windows phone, because there were no apps left. [Laughs]

Facilitator: Okay, great. In terms of accessibility, how do you think we could make it accessible for every adult, for everyone? Like, people with impairments?

Female 4: I think there are plugins that you can get, like, companies – this is the whole thing they do, where it creates a – and that’s to do with how you code your app, that it can then read it out to people. And actually, it would be extremely important, because this is, to a certain extent, covered by legislation in Canada, especially in Ontario. Ontario has a very strict disabilities thing.

Sorry, I'm a lawyer, so it’s [unintelligible 01:05:28] obvious, really. These are the things I think about, privacy issues and disability and discrimination, and there are companies out there who will take your product and make it legally compliant.

There was a ridiculous situation, I remember, a couple of years ago, where a guy was suing Porn Hub, because there were no subtitles on the porn video that he was watching, and it therefore did not meet the Americans with Disabilities Act. [Laughs]

A lot of great stars. Anyway, I don’t think it was ever – I think it’s still in court, I don’t think it was resolved yet. But everything legally needs to be accessible. So, whether it’s having your own coding that allows it to be read out, or integrating a different thing, I definitely – my unsolicited advice is, get that, because otherwise it’s – as soon as you don’t have reach to everybody and people saying, “Oh, hey, this thing was put out, but can’t use it if you're visually impaired.”

Or what about people we were discussing before, who don’t have the education level required? Maybe there could be a section, something like a quick introduction to nutrients: This is what a carb is, this is what a protein is, this is what a grain is, this is what they do, this is why it’s important to eat them.

And I'm sure some of this exists in the guide, but if you’ve downloaded the app, to have it right there in your hand, and a quick button that says, “Oh yeah, reminder, this is what this is.” I think that people would really appreciate that, because it’s hard to correctly determine exactly what everybody’s level of understanding is.

So, then you've got the whole French-English thing, and it’s [unintelligible 01:07:21].

Male 1: Yeah, there should be a high contrast mode for people who are colour-blind, screen reader for people who have trouble seeing at all. There should probably be speech input for people who have motor control issues.

Male 2: To add on what was said by Shai, the Canadian Food Guide is available in about [unintelligible 01:07:54] languages, so maybe in a [third vignette 01:07:59], and sort of slides. Maybe if people are ؘ– and there might be a priority on how to get this done. You know, there is a – I'm not sure, Canada government had to make the – it is [unintelligible words 01:08:17].

So, from what Mark said about high contrast, it may be seen of a dark mode. Now, there is a white background by default, or a black background by default. It’s a game-changer.

And when you start one, you don’t really want to go back to the other one.

Facilitator: Yeah, those are really great points. For the interest of time, so I'm just going to wrap up the conversation. Just tell us your two cents on this application that could be really helpful, the features that could be included that we have not mentioned during the discussion.

Male 3: What a challenge ahead of you. I wish you luck.

Facilitator: [Laughs] Yes.

Male 3: Can we field test it?

Facilitator 2: Actually, yeah, we’re actually having a pilot study after the app has actually been developed, and we can definitely contact you guys again if you guys are still interested.

Male 3: Oh, I would be.

Male 2: Yeah, I would be too, yeah.

Facilitator: Sorry, I just wanted to mention that this application would be developed based on your general perspectives and ideas, and we’ll have another focus groups round for the experts in the field, like the registered dieticians. We have forty dieticians that have their own perspectives on this application, and we have your perspectives on it, and we will develop it based on this whole idea. And we will pilot it after.

Male 3: Great.

Male 2: That’s a great challenge. I really like the simplicity to evaluate how many edges and realise that – at least if it just helps realising how many – well, the [unintelligible 01:10:35] or, what’s the proportion of your eating, if it just raises awareness, that’s very good.

There is a Duolingo – I don’t know if anyone uses Duolingo to learn languages – but you have this day streak, where you know, if you practice one lesson a day, you have a streak, and more you get, the higher, and so on. You know, it feels like a reward.

If you had something around this where, you know, just – [unintelligible 01:11:05] a day, so people can get engaged, you know, where you submit one meal a day and then you get your day streak, your sort of day streak, and then that could be a way to bring in – to engage people and to, you know –.

Male 1: Yes, I have a two hundred and eighty [unintelligible 01:11:25] day streaks, and I still can't speak French, so –.

Male 2: You didn’t choose the best language.

Facilitator: Well, feel free to jump out if you have any other things to do, and you could just email us your other thoughts on the application, and we’d appreciate all of them.

So, any other ideas?

Male 2: The Canada Food Guide online, I have the page up on the right, and there is an HTML version, so I think you shared with us a PDF version. But if you have in the app the possibility to just mimic those sections, because it seems pretty well done and pretty accessible, and the categories make sense, you know what I mean?

If you have this in the app, it would be interesting, because I think the website is responsive, so even open an in-app browser, you know.

Facilitator: Yeah, great.

Female 3: Just to save some time – I don’t know if you guys can do that, but I would probably just take another app that is the most popular, and that people are the most comfortable with, and I would just do the same app, but improve it with all the things that you think are important, based on the discussion and so on. Obviously being legally covered, so you don’t look like you copied them.

But yeah, I think that would be the easiest to do, because people are already comfortable with different types of apps, and the people that are interested in tracking their food, they already have an app, so –.

[End of recorded material]
