# Supplementary material for: Perceptions of self-monitoring dietary intake according to a plate-based approach: A qualitative study
Source: PLoS One. 2023 Nov 28;18(11):e0294652. doi: 10.1371/journal.pone.0294652 (PMC10683993; doi:10.1371/journal.pone.0294652)
Supplement: S4 Appendix — (ZIP) [file pone.0294652.s004.zip › Anonymized GP Transcripts/iCANNPlate-GP-Focus-Group-6.docx]

**iCANNPlate-GP-Focus-Group-6**

[Start of recorded material]

Moderator: So this is the iCANN Plate focus group with the members of the general public on August 1st at 3 p.m. EDT, Eastern Daylight time. So first of all, let us know what would make it easy or hard for you to eat in accordance to the food guide, to Canada’s food guide that we just showed to you. What is good about it? What can you find it hard to follow? Let’s see the guide here. Yes, please, just start – yeah, you don’t need to raise your hand, just jump in and – yeah, it’s like a discussion. Go ahead.

Participant 1: OK, I was going to say the sort of normalized American portions. Like not only portion sized but like what is an American dinner if we think about it? It’s like mashed potatoes and a huge steak and that doesn’t necessarily map over to this. Especially like our understanding of what’s a good dinner doesn’t match with what’s – what our bodies really need or what’s healthy and it’s like that was enforced decade after decade.

Moderator: So do you think everything about the steak and mashed potato can be here on the guide?

Participant 1: Sorry, what do you mean?

Moderator: For example, you know that the mashed potato contains maybe sometimes – sometimes people maybe add something else to the mashed potatoes that are not necessarily in this guide. Like maybe creams or –

Participant 1: Oh, yeah.

Moderator: – cream, yeah.

Participant 1: Which like, you know, cream isn’t bad, like I love heavy cream. I make huge smoothies with like a ton of heavy cream, which if doctors dietitians saw that they would say, “You’re going to die of cholesterol,” but I do not – I’m OK, I haven’t died yet. But I think the dose makes the poison, I guess. Like steak alone isn’t that bad of an item perhaps; it is almost purely protein, which is good. But, you know, when somebody has that day in, day out, which like North American wealth allows that to happen. You know, that can cause so many diseases. Like historically, gout was known as the king’s disease because only kings could afford to eat meat every day. And now we’re it’s an economic situation where most people probably can afford that one way or another.

Moderator: Yeah, so in general, do you see this guide easy to follow? Is it applicable for you?

Participant 1: I think so, yeah. I feel like there are a lot of things it leaves out. So it’s like, “Oh, what am I supposed to do with that then?” For example, as we were talking, like heavy cream and drinks, things like milk, how does that play into this necessarily? And also sweets and sugars, there’s no dessert on this. It’s like sure one can probably from a dietitian, like nutrient standpoint, one can probably live without cake. But like [Participant 6 00:03:59] and I, I know, like we can’t live without cake so how does that play into this is a question as well.

Moderator: Right. What does everybody think about it? I agree with [Participant 5 00:04:13] but I think this picture is a very good picture; it shows exactly what we are supposed to eat for a healthy lifestyle. But I think they could have incorporated the calories or the portion that you’re supposed to eat and also during, like for breakfast, lunch, and dinner or snacks in-between, because portion control is very important. Yeah, and also as Participant 5 said, they could also include the liquids that we have, like specific if you can have milk or juices, fresh or store bought or something. I mean, yeah.

Participant 1: And also, perhaps, even alcohol which doesn’t have much nutrition, but obviously it’s something that goes into our body. I was also going to add, perhaps in terms of like healthy living and how that relates to eating, like I don’t read these studies myself but my mom does every now and then and tells me, like, “Oh, don’t eat past 8 p.m. and don’t eat right after waking up,” and whatnot. But I know that it’s a habit that a lot of people have or a lack of consideration or stuff like that. It’s like, “Oh, I’m hungry and it’s midnight. I’ll eat and then go to bed right away.” Personally, when I do that I feel really bad the day after. That’s something.

Moderator: Yeah, great idea. Yeah, I think [Participant 2 00:05:55] want to add something?

Participant 2: Oh, yeah, I think I wanted to touch a little bit more on what Participant 5 was saying maybe about kind of like traditions. Like maybe we grew up traditionally understanding food a certain way. I guess from my personal experience I used to live alone when I was in college and I was kind of in charge of preparing all my own meals for myself, so I could always choose what I wanted. And I recently – I graduated, came back home and live with my family now and my grandma lives with us as well. And she’s very picky and particular about her food and the result of the family kind of like molds to what she would like even though maybe it’s not the healthiest thing but it’s something that she’s familiar and wants to eat often.

Just looking at this picture, I think it’s an attractive picture, but for her, so my grandma’s from Malaysia, I don’t recognize a lot like the foods that she would eat, for example, maybe like a curry. Like a curry doesn’t look – like you can’t pick out the ingredients in it, it’s just like a big bowl of stuff that you eat. And like this picture here I feel like if I was trying to eat in accordance to this guide and I’m going to match this picture, like half my plates are veggies and a quarter is protein, a quarter is grain. If I was my grandma looking at my curry I would not be able to pick out how much of what I am eating. Maybe I also wouldn’t care because she’s a little picky, but that is something I wanted to throw in.

And yeah, so I guess there’s a bit of a cultural aspect to this picture as well. Like these ingredients remind me more of Western food than it reminds me of Malaysian food.

Moderator: Great idea. Yeah.

Participant 1: Yeah, I think I can share a bit of a cultural experience with that as well. I grew up in Turkey before I came to Canada for university and I’m not a big bread person and I never was, especially as a kid, but bread is a huge part of Turkish eating culture. And I used to always be told like, “Oh, if you don’t eat bread you’re not going to be a big man.” So it was always, “Bread, bread, bread.”

Moderator: Yeah.

Participant 1: Which really helps shapes one’s notion of what one should be eating or not. And I know my grandparents, for example, it’s like, OK, food, and then there’s probably an equal amount of bread that goes with that.

Moderator: So I think the quarter of the plate that belongs to bread is not enough, right, for people who are living in Turkey –

Participant 1: Probably not. So it’s like, yeah, it’s just another example of culture I think as Participant 2 was mentioning.

Moderator: Yeah. Right. So [Participant 3 00:08:56] or [Participant 4 00:08:56] did you want to add anything?

Participant 3: I mean, I agree with Participant 5 and Participant 2 how like the cultural is like a bit of a barrier to how we would eat, because especially from my family we always have rice at least once a day. It’s kind of like a staple, like my diet, and it’s just kind of like when I say only about a quarter of rice, I can understand I should be eating that, but it’s like rice is so good that I’m like I really enjoy it. I don’t know, even just having rice with just like a sauce is still enjoyable as well. So trying to cut down on like carbs, especially is a little difficult, but I can understand, like this could be kind of like a step in the right direction to what I should be trying to change for my diet, I guess.

Moderator: Yeah, that makes sense. So how about you, Participant 4? What you think?

Participant 4: Yeah, I’d also agree with the others and for myself it is somehow maybe difficult to follow this guide, and especially I think it is because of my habits and my tradition. Because we huge amount of rice and rice is somehow has the biggest part of our nutrition. And when I see this plate it is somehow very different from my eating habits. And I think if I want to follow this one I should change a lot and put a lot of different things in my habits. So it is very attractive and I know it is very healthy but it is somehow a change, a big change for me. Especially when I came to Vancouver and I am somehow changing my habits and I see that it is really difficult for me now to get to use this. But, of course, it is really healthier than my tradition nutrition habits.

Moderator: Great. So it seems like we have a lot [unintelligible 00:11:04] today.

Participant 4: Yeah.

Moderator: Yeah, great, so – yeah, go ahead.

Participant 4: Nothing. Just I wanted to agree with you.

Moderator: Yeah, totally agree with all of your thoughts, yeah. So if you want to make some changes to your diet or eating more healthily, how would you speak to those changes? How would you follow the changes that you’ve made? Would you self-monitor, monitor yourself, yourself to be in the right direction to make those changes in your diet? What techniques would you do? Like any goal settings or like support group? Journalizing? What would you do?

Participant 3: I think for me I would probably make, maybe like a set thing I would eat, maybe like three times a day or at minimum like twice a day depending if I’m busy or not. Like if in the morning I would probably want to eat more fruits and maybe more – maybe go for more veggie stuff and stuff like that, maybe in the morning. And maybe more later in the day it’s maybe more meat, maybe a little bit, like some carbs and all that stuff. And then lunch maybe – maybe a good mixture of both like veggies and even some of that and have that set – like every day or something like that. And I’ll try to illuminate like snack in-between as much as possible. I think that’s probably what I would do and maybe try to keep to that routine as much as possible.

Moderator: So that would be just in your mind, you don’t follow any like writing them down or following them in an application? You don’t any of them, right?

Participant 3: Yeah, just probably note to self and stuff like that. But maybe a journal would also help like ease it, like, the whole healthier eating and all that stuff.

Participant 6: I had – oh, I’m sorry.

Participant 1: Go ahead, Participant 6.

Participant 6: OK. I think a few months ago I had started using this application called HealthifyMe, so where you could actually put in whatever you ate and the amount of, like, the portion size or you can calculate by a cup or the weight, and just keep on adding up and then it will show how much, like, how many calories you’ve taken in a day. So I did that for few days, not even a week I think, but it became very difficult for me to log in and then put everything on there and then see if I have achieved the goal or something.

Because I specifically remember because it keeps on sending you notifications, you know, “Have water” and that’s really good, hydration is really good, but then I felt that I don’t even enjoy my diet because I started having salads and incorporating a lot of healthy food, which I was not a fan of in the long run. So, I mean, it was motivating but I wasn’t that motivated to continue in like after a week because I was really bored. And also, as I working it became really difficult for me to monitor my food and sometimes I work late hours, so it was really not possible for me to get that in a restaurant or a takeaway or something. So that’s what I did.

Moderator: Yes, Participant 5?

Participant 1: I was going to say, I don’t know if this is what they call “cold turkey” or not but when I’m trying to reduce the amount of deserts I eat or like cut out something specific and whatnot, I know that I am too lazy to get up and go to the grocery store when I want something. So if I don’t have it at home there’s a good chance that’s going to be that way. So it’s like, “Oh, if I want to eat more salads in the upcoming week or two weeks,” it’s like, OK, then I’m going to get my tomatoes and cucumbers and like load up the house with the good stuff and if there is no bad stuff then I just can’t have that like physically.

And one thing I think people should keep in mind when they try that is to not go grocery shopping while hungry because then you end up getting, you know, the chocolate and everything.

Moderator: Yeah, totally.

Participant 2: Sorry, the question was how do we track what we’re eating, right? Is this –

Moderator: No, what techniques would you use when you want to make any changes like that?

Participant 2: What techniques? OK.

Moderator: Any techniques.

Participant 2: Oh, any techniques, OK. I think I tried using an app before, it was called like My Fitness Tracker, but I don’t know, my friends told me really good things about it but I personally didn’t like using it. I think this is more of like a user issue with the app. Sometimes I couldn’t find the serving size or portion that I was eating on the app and then it just took long for me to figure it out. Like I was in-between a class or maybe my lunch break was ending, I have to go back to work and it’s not worth my time to spend time trying to figure it out, like how to use this app.

So I think this app wasn’t user friendly, so I guess it didn’t really – or maybe I thought – I might have thought that I was making changes to my diet, but I guess I can’t really say for sure if I am not tracking what’s happening and I’m just kind of trying to think back to what I ate because I don’t think memories are reliable in that way.

I was considering starting to create a journal, but I also think – I haven’t started yet, but I think there’s flaws to that. Like if I was ever eating out I would need to bring my journal with me. And also another thing about eating out is when you order something you don’t always know what is inside the food. Maybe they put a lot of butter or salt that you would need to record but you don’t know about so you don’t. So I think it’s actually quite hard to track and if you want to make changes to your diets to see if those changes are being made because sometimes you might not know what you are eating.

Moderator: OK. Yeah, great. So you already mentioned – did anybody else want to add anything?

Participant 3: Oh, like Participant 2’s point of – the idea of eating out, a lot of people – like I lived in my university residences for three years, so there we had our cafeteria. So it was the same idea of like eating out but like on the daily when you’re not the one making the meals not necessarily knowing what is going into that. And like that may or may not even happen at home if your parents are cooking. There’s a good chance that you’re involved in that a little bit or at least you know what’s in the fridge and everything, but when you’re not the one on top of your diet, basically.

Participant 6: Also can I say something?

Moderator: Of course.

Participant 4: Yeah, thank you. One that that would really help me is that if I decide something to follow with my other friends that’s really helpful for me. When I decide to make these changes on my own it is somehow harder for me to follow. For example, here a group of my friends and I are trying to work every day and we are very careful about our steps, we count it with our application and it is really helpful for us because we are a group and we are all very, I don’t know, it is very important for all of us to follow that.

But here I decided some time to make changes to my diet but it wasn’t very helpful for me because I’m alone and the other friends do not care about it and when I see them that they are easy going, they do not care too much, they do not – all the time consider about how much calory they are using, and that makes me somehow, you know, it makes me disappointed and so I do not follow that anymore. So I think some thing that would help us be in a group so that we can – in a group that all of us have this challenge it would be really helpful for us. I don’t know, it would work for me a lot.

Moderator: Yeah. Totally. Interesting idea. Anything else to add? OK, so I know that some of you have tried some applications to tracking your diet. Participant 2 mentioned My Fitness Tracker, that it was really hard for measuring the portion sizes or yeah and for tracking them. And I think Participant 5 was using one and [unintelligible 00:21:02]. Yeah, so let us know more about them. What specifically about those applications made it hard to follow or made it really easy for you to follow? Say, both points. Yes?

Participant 1: Would intermittent fasting play into this idea?

Moderator: Have you used an application for that?

Participant 1: Yeah.

Moderator: Yeah, go, talk about it. Sure.

Participant 1: Yeah, especially when I was at college, university, during finals seasons with like no classes I would also take time off work for that. So like no classes, no work, no meetings, no nothing, and then suddenly because it’s a very clear schedule and with the added stress levels my eating schedule would like all over the place. Sometimes I would binge eat in the morning and then fall asleep after that, wake up for 8:00 p.m., eat a little bit more, study until 3:00 a.m., go to bed, wake up at 12:00, like it was all over the place.

So I started doing intermittent fasting during finals period and I used an app called Fastic, C, Fastic. And that one the more hours of fasting you do, you sort of level up, so they gamify it a little bit. It also had those reminders of like, “drink some water” and then it would also ring you, like, “it’s 6:00 p.m., go eat something.” It would also show you what process you are in your fasting journey. Like first hour, OK, your insulin levels are dropping, then your liver kicks in, then your other hormones are kicking in, and then autophagy happens and what not.

So it’s like, “Right now this is happening in your body.” I don’t know, I think that app was doing a really good job. You could also add friends and if they were fasting with you, you could see how many hours they’ve been doing. So I don’t know, it was helpful.

Moderator: So the reminders were really interesting for you, right?

Participant 1: They were helpful, yeah, because I would lose track of time and then “bing, 6:00 p.m., you’re done,” yeah. And it was one button you would hit to start your fast and to finish your fast.

Moderator: Great. OK. Do you want to talk about the apps that you were using?

Participant 2: I think Participant 4 tried to unmute earlier but I’m not entirely sure because she unmuted again.

Participant 4: Oh, no, that’s OK. I will add whenever I have something. Thank you.

Participant 6: OK, I shall talk about the app, what I was using. So I was using this app called HealthifyMe, so it’s very easy to use and the way they’ve designed the app is really user friendly, it’s just I don’t have the time to, you know, put in the data every now and then. And so, the good part about that app is it has a pro version and in that pro version you have a personal coach who will get to – I mean, I didn’t try the pro version but it was free for me for like seven days so the coach tries to get to know you and, you know, your lifestyle and according to your lifestyle, your work, and where do you stay. So he or she, they try to give you suggestions, like what you can eat and what time you can eat and what are the exercises or something you can start with or continue or engage in. Some fitness activities, so that was a good part which I really liked.

But I actually felt sometimes when you go out or you try some new food you really don’t find that there. And I mean, I really can’t weigh my plate in a restaurant, right? Like how much it weighs or how many calories it has, they don’t really mentioned it everywhere. So that was something which I couldn’t enter. And sometimes I feel the portion side in the restaurants, like sometimes they are too big and I really can’t say that I ate half of this or whatever, it’s a portion size I ate. So that was what I found a little difficult to add on there.

But otherwise, it was really good because it helps you track the number of calories and it also tried to maintain, so there’s a goal every week or every month, I suppose. So you are supposed to review so many kilograms and for that, suppose you set up a goal, so you have to like eat so many calories and work out so much or so little to gain that goal, I mean, to at least reach there and they keep on motivating you, you know, sending you all those inspirational notifications that, yeah, “It’s time to hydrate. Yeah, let’s add some proteins,” or you know, “this meal looks a little high on fatty calories so let’s add a little protein or some healthy calories.” So that was really good, which I liked.

Moderator: Interesting. How about the My Fitness Tracker, Participant 2?

Participant 2: I think the things I didn’t like about it was they didn’t really have the – I used it a long time ago, I kind of forgot how it is used. But I think it is – the meals that I would eat, I would have maybe like a bowl and I would have like quinoa and I would have a bunch of ingredients. It would be very time consuming for me after lunch. First, I would have to have my phone on me, I would have to remember to use this app, and then I have to go into this app and find every single ingredient that was in my bowl. And I have to – another thing for me was I don’t really understand what 100 grams, 300 grams, I don’t know what this looks like. To me it would be more helpful if it was like, “The chicken breast was the size of my fist,” or something, but I could put something – that would make more sense to me than like 100, 200 grams. It’s the same as like Google maps when it’s like, “In 300 metres,” like I don’t know when that is.

But I think it was difficult for me to use the app because I had to always have my phone on me and I had to always remember to go onto this app to log every single ingredient I had and it was very tedious to look in ingredients and then also try and convert in my mind, like, “Is 300 grams my fist or is that 100 gram?” So it was just a little too time consuming and it took up too much of my mental space for me to want to use this app.

Yeah, and I think it’s just – I wish there was something easier. Maybe it’s like you can take a picture of your meal and the app can identify the things that are in it and maybe how much it is. I don’t know if that’s very techy. I’m sure we could do it. Or maybe if there’s a way like there’s a meal you eat all the time, like I eat a lot of noodle soup all the time. If I could save my meal into my app and then instead of going through and finding all the ingredients I could just say, “I had my usual noodle soup again.” But I didn’t find that on that app. Maybe it existed but I didn’t like it enough to find it, so, mm-hmm.

Moderator: Great idea. Yeah. It’s really hard to measure everything or can visual everything and change them to grams, yeah, it is really hard. So do anybody here know of any self-monitoring application that kind of resembles or mirrors the Canada’s food guide that we just showed? Like the plate method? Yeah, no one? OK. Is there anybody want to add anything? Participant 3? Participant 4? Did you guys want to add anything here? Saw our first mock up, let us know how you view this app working when you think about your eating throughout the day? How do you see it working? Yes, Participant 5?

Participant 1: Again, to start it off I feel like to visualize for one plate, perhaps really usable, perhaps really good. I’m thinking it like relative to how I tend to eat which is on multiple plates. Sometimes if there’s a soup and I don’t know, something else solid, like one is in a bowl, the other is on a plate, how do you quantify soup? I don’t know, or yeah, I would say most people probably have dessert on a separate plate to begin with, so there’s that. Some things aren’t on the plates at all, like if you’re having some bread on the side. Sometimes things are on top of each other the way you plate them.

Participant 2: I kind of agree with Participant 5. That reminds me of when I go eat maybe like dim sum or something where it’s like there’s a big – there’s all the dishes in the middle and you just kind of like take one thing and your plate is this small. And you eat it and then you go and take more. So I would be remembering like thinking about it in a giant plate and how many dumplings did I have and this and that, because you’re just enjoying yourself.

Participant 1: Yeah. Or again, me and my friends all you can eat sushi is our thing, and like for that to think, “Oh, this is the sushi and there’s a little bit of meat in there, so is that like 10 percent of the sushi?” That’s not really feasible. So it’s like, OK, 70 percent rice, I guess. Some like cucumber, avocado, whatever, vegetables in there, but yeah.

Moderator: Yeah. What everybody else think about it?

Participant 3: I agree with Participant 5 and Participant 2, it’s just like understanding the proportions that we would eat on a daily basis is just kind of difficult to visualize on just like a single plate, right? Because sometimes in Filipino culture we have like dishes that are more saucy, so it will be like kind of hard to determine if it’s like how much meat and vegetables because it’s all mixed in with like the sauce. It’s like the sauce itself, it’s like, how much of that have I actually eaten as well? So it’s just going to be a little difficult to kind of place that on the screen, basically, and just kind of visualize that. And then I kind of tell the app like, “This is how much I’ve eaten,” I guess.

Participant 2: I have some other thoughts about the app.

Moderator: Yeah.

Participant 2: If no one else is ready to share yet. OK. When I first saw it, I was actually very attracted to it. I thought it was very user friendly. I guess my issues with the My Fitness App was that I had to find each little ingredient and then put it in; it was very tedious. And it seems like this app has taken away all of that, I don’t know, annoyance, because of finding each little vegetable I’m eating I’m just saying I ate this much veggie. But I guess a thought that came to my mind is I used to be very anemic, so I wanted to keep track of my iron and I’m guessing maybe this app is – I don’t know if it would be detailed, but maybe it’s a little too simple, where I can’t track – like if I’m saying eating veggies maybe I’m eating lettuce that doesn’t have a lot of iron. Or maybe I’m eating spinach which has a lot more iron.

So I don’t know how I would be able to track like if I wanted to track my iron specifically, how would I do that with this app? So I guess it might fit someone else’s goals but maybe for like my goal, and I’m sure other people are – maybe I want to eat more, I don’t know what someone else wants to eat, but it might not suit their needs.

Moderator: So you mean the app could be like customized for everyone based on their goals or their needs and you – for example, some reminders or some guides based on their instructions based on their goals could show up, right? In the app.

Participant 2: Sorry, was that a question or more of a –

Moderator: Yeah, I was just following your – yeah, I was just probing more on what you were talking about. So from my understanding you meant some instructions or some reminders or that are customized for everyone would be helpful in the app, right?

Participant 2: I was thinking more of the app looks very simple and user friendly but I don’t know if it would be a little too simple. If I was just recording vegetables but really I want to know how much iron am I getting from my vegetables, the data that I would collect from the app might not tell me how much iron I’m getting from my vegetables which is what I want. So yeah, but on a separate note about reminders, and also when I was talking about reminders with his app, I found that really interesting and if I didn’t have like a set eating schedule I would like reminders. But that’s more separate from what I was talking about before.

Moderator: Yeah, we’ll get to that as well. Yes?

Participant 1: I was going to add, perhaps, in a similar way with the apps user interface, like that is very simple, it’s really nice, but I hope that there’s a side to it, like behind the input, where it averages out what you’ve eaten that day, that week, that month, and then sort of returns to you, either some sort of advice, some sort of result, being like, “Hey, you have been really carb heavy, be careful. You’re going to get a heart attack.” I don’t know.

Moderator: Yeah. So yes, Participant 6, you wanted to add something? Yes, Participant 5, do you want to continue? Sorry.

Participant 1: Oh, the idea that followed that up in my head was, again, how that might need to be like customized to persons. So like for example, a young, skinny guy who was trying to put on body mass, you know, like he was going to the gym, working out and whatnot, he probably would eat a lot of proteins and a lot of carbs just to be able to get all those calories in whereas someone trying to lose weight would most likely be cutting down on the carbs but still keeping high protein, so that would also need to be told to the app.

Moderator: Great idea.

Participant 6: I agree with Participant 5 and Participant 2. So it can maybe have more details where you would actually look for maybe carbs or protein or iron or something like that. And I think it’s too simple. Maybe it’s simple for being – for people who want a simple app, but I think for me it could use a little bit of details, like here and there. And also like me, you know, kind of see what I’ve eaten through the month or how am I doing and everything.

Moderator: Any other ideas?

Participant 2: I’m just adding on to the details like what Participant 6 was saying and what I mentioned before. Maybe it would be interesting if like we had our plate and then part of it is green, like the vegetables, if we could tap on that and then list out all the vegetables that you had in that and then – or for protein you can tap on it and say, “Oh, I had chicken, but I also had tofu,” et cetera, so that for each meal has some details. And then maybe somewhere in the background processing of this app it can calculate – because I’m sure that like protein from chicken I’m pretty sure is quite different protein from tofu. So it would be able to see like what you’re getting, whatever you want, so ...

Moderator: Interesting idea. Yeah, great. Yeah, anything else? OK, so let’s talk about the portions that I know that Participant 2 has some – was struggling with the previous app she was using. So I’m wondering how do you suggest to represent different meals, portions when you want to try your food in this application? You know, like how would you measure – like for example, what references would you use to measure your portions? Yeah, do you prefer like fist and palms or like weight or what do you prefer? What do you suggest? Yes?

Participant 1: I’d say generally relatable objects like a matchbox or like a mug or a tea cup.

Moderator: A tennis ball?

Participant 1: A tennis ball, a ping pong ball, like things that most people can visualize in their head. One would really need to think about it perhaps, but yeah.

Moderator: Any other ideas?

Participant 6: I think I would go for, like Participant 5 said, any objects which I know will have a standard size and shape and will not change, you know, according to [unintelligible 00:40:26] or something. But I think I would also go for that and I would maybe use a cup which I know which will hold so much food or whatever I’m taking and I will always try to measure in that bowl or that place and go forward with that. So that will be easy for me but that will only help if I’m eating at home, so I cannot do that every time if I’m eating out or at work, so yeah.

Moderator: How about some mixed dishes, you know, mixed dishes have so many different ingredients in them, how do you want to portion – how do you want to measure their portion? Does any like ping pong balls work for them, for their mixed dishes, or for their sushi dish that you were talking, the little avocadoes in them, what do you think about them?

Participant 1: If the person’s involved in the preparation stage then like standard measurements for like cup, half a cup, a quarter of a cup, those can be done because generally when you’re making rice you go, OK, I’ll use this mug – I don’t know, I use the same mug when I’m making rice every time. So if I measure that once with an actual standard cup, now I know, when I make rice, it’s actually one-and-a-half cups or something. For mixed dishes I feel like that’s somewhat reasonable if you’re seeing the amount of things going into it.

Participant 2: Yeah, I think I agree with Participant 5. I think that if I was cooking at home, I was making like a Dal or something, I would – as I’m cooking I would be using cups, et cetera, so I would know how much is going in. but I guess one thing to consider is you might be making like a big pot of Dal but you’re not eating it all. So if you put in one cup of lentils or whatever, you’re probably not going to eat the whole pot so you’re not going to eat the whole one cup.

Participant 1: Yeah.

Participant 2: Yeah, and I think going back to your question, [Marion 00:42:44] about like the mixed dishes, like the sushi and whatnot, honestly, I would just guestimate. It’s kind of hard to measure in your mind as you’re eating it, you know, and then you have to remember every piece of sushi you had and what was in it. It’s a little much for every meal you have.

Moderator: Yeah.

Participant 1: I know that built into Google is actually a pretty good service about like calories and nutrition if you search, I don’t know, “How many calories in one eggplant” it will give you a couple options, one of which probably first your case. It will be like one long eggplant or one small eggplant, or one circular one, or half an eggplant, or a quarter – like it will give you many options, one of which applies to you. And for most food items it knows that about like what people would be using. So those are some nice resources.

Moderator: Interesting.

Participant 1: Like with Participant 2’s app previously, the one that would ask for grams, I feel like I used something like that in the past where I’m like, “I don’t know how many grams this is,” so then you Google that with, “How many grams in one cup of rice?” or something and it generally comes to, you know, a [guestimatable 00:44:17] good answer.

Moderator: And – yeah?

Participant 2: Or sorry, you go ahead.

Moderator: Sorry, I think Participant 5 – do you think people would do that when they’re using the app? Do you think they would Google everything ...

Participant 1: Well, from my user experience or like as a design standpoint they shouldn’t be, like an app shouldn’t require you to go on Google so that you can standardize the input that you’re putting into your app. Yeah.

Participant 2: I agree, I wouldn’t Google it. And in the past, I did not because it was too much time. It’s not worth your time like –

Participant 1: Imagine you’re signing up for something and it asks for your country but it asks for the three letter code of your country. And sure, for Canada, you know it’s CAN but if you’re from Bangladesh is it BAN or is it BND or is it BND? Like then you’re, OK, I’m going to Google this, find the three letter code and then put it into the app. That’s just bad user experience, bad user design.

Participant 2: Yeah, I agree with that. And building on that, I think it would be nice if the app had some sort of kind of like reference guide, for example you could click on and it would show you something where it was usually one chicken breast, I don’t know, one average, oh, it’s kind of hard, chicken breast could be large or small. But maybe like the size – you could say like a softball equals 300 grams or something like that where like so now the user is also learning what does 300 grams, 300 millimetres, like whatever, how much that looks like in comparison to objects that probably don’t change.

Like for example, I like using my fist because I can just look at it but everyone has a different size fist, so maybe that’s not something the app should be using. Or if I have a special cup at home that I always use but no one else owns in the world and we’re trying to make this app for everyone in Canada then that would not be a good object either.

Moderator: Oh my God, I’m just looking at the time now and it makes me – you guys have really interesting ideas for us and I don’t want to – I just wanted to keep going, yeah, but I don’t want to bother you and keep you here more than the time that we have told you before. But let’s go to the next question, that is I’ll ask about something that you just mentioned about some instructions. What other instructions do you think should be in the app, like some [scores? 00:46:52] or instructions could be helpful when you’re using the APP? Like the – yeah, you just mentioned some instructions about portion.

Participant 6: I feel if – I mean, the app should calculate on it’s on it would be really helpful for me as a user to suppose if I wanted – if the app was asking me in the size – in the measure of cups, so if I don’t know how many cups it was I just add grams. So the app should or could just calculate it for me, so that would be easy for me rather, you know, to search in the reference or the guide or anywhere else in Google, you know, how much would that be.

But also I know it’s – I mean, I don’t know if it’s possible but I did see an ad when I was browsing through the internet, like if you just click a photo of your dish – it will just calculate in the back end saying how many calories and what is the size and portion. But I did try that app only for a day to see that it doesn’t work with everything because it uses the presentation of the food. Suppose if we just make your food, the app doesn’t recognise or doesn’t make it out as to what it is, so it’s difficult to use it. But sometimes it just – it figures out eggs and everything but I think if you go for calories or any rice, you know, preparation, so it wouldn’t know what exactly is on there, like on your plate, so I think I feel that ...

Participant 1: Yeah, I think for AI to be able to understand what food is that’s going to be a huge project that like Amazon or Google can handle themselves. But for a simpler, user-friendly app that can get created quite easily, that can get supported, developed quite easily, I mean, I agree with Participant 2 and Participant 6 in terms of having some documentation and guidance built into the app. So as to make it easy to input whatever it is that you want to input but to also learn a little bit at the same time. Like, “Oh, and I guess this about half a cup,” or like half a cup is about 50 grams, something like that. Yeah.

Participant 2: Does Participant 3 have anything to say before I say something? I feel like I’m doing the talking.

Participant 3: Oh, yes, I’m just kind of thinking. But for instructions, I don’t know, maybe the app, maybe have little videos to maybe give like a visual, a representation of like tutorials or anything like that or portion sizes or something like that to give – yeah, just like a visual representation of how to do things and all that stuff or what you should be doing, something like that.

Moderator: Great idea. Yes, Participant 5?

Participant 1: I was going to say building onto the idea of the app sort of in the background, like analyzing the data that has been put into it and then returning some meaningful results, I was thinking what about some recipes that can get built into the app and those recipes are going to have their meal profile, like how much vegetable, how much carb and what not. And the app can suggest one of these recipes to sort of out your meal profile like “Oh, you’ve been too heavy on carbs. Here’s a mostly vegetable dish,” or something.

Moderator: Interesting. Yeah, I love it.

Participant 4: Yeah, so I think if I want to use an application to change my diet I would prefer some that I can follow my health condition as well. I don’t know it may be very difficult or tricky, but I would prefer some that at the very beginning it has an optional section to ask for – or for example the lab results for example, because all of us do some regular checkup blood test. So I think it would be somehow beneficial to get that results and after a while it gives us some feedback about what should we do with – by considering these, for example, condition for example the blood sugar or the cholesterol or something like that. Yeah, I think it would be really helpful because I, myself, care a lot about my health condition and if an app has this option I would definitely use that, yeah.

Moderator: So do you think just reminders would help or some tutorials and instructions and yeah something else could help as well?

Participant 4: Yeah, I think reminders would be really helpful because it would help us a lot to speak to the application and also the tutorials would work best [beeping] – sorry for that, but if I have an option between these two I would choose the notifications most. Yeah.

Moderator: Yeah, it’s like a burden for the users to like –

Participant 4: Yeah.

Moderator: – see the video tutorials or read them, yeah.

Participant 4: Yeah, you know, because we can have access to these tutorials through other platforms. We can just search and find a lot of tutorials, so if – yeah.

Moderator: Yeah, so how about some – the other foods that we earlier mentioned about like the foods that could not be in the guide, like the cream in mashed potato, or like cake that [unintelligible 00:53:07]. How do you suggest tracking those for ...

Participant 6: I basically have no idea how to track those because it – I mean, sometimes you just – like when I bake, I kind of substitute something if I don’t have like the – if the recipe says it should be 2 percent or 3 percent milk, sometimes when I don’t have it at home I generally substitute or I can substitute with some almond milk or some other alternatives or – I don’t know how to track that. And also the portion of cake I am having, I don’t know how to calculate like how much to cut or – I mean, I don’t want to weigh that every time I have the cake, right? Or the dessert or sometimes it’s just a homemade recipe that you follow so I cannot track the calorie intake when I prepare that. So it’s difficult for me, so I just stick to the principle that you’re supposed to have less desserts and have more healthy food and have like those tiny portions so that you know you are healthy and it’s good for you.

Moderator: Yes, please, Participant 5?

Participant 1: I’m going to say perhaps a button that can be used to basically grab the attention of the real human behind the scenes, like a developer, and to all of them, like, hey, mashed potatoes for example, and the user can write, “I’m not sure if you guys are assuming mashed potatoes have cream or not.” And then for every instance of this like reporting and inconsistency surveys can also be launched to users at it – like one question surveys every now and then when you go into the app, it’s like on average do you put cream into your mashed potatoes? And if most people are saying yes then perhaps the code background is going to be changed to assume, OK, like most mashed potatoes have cream in it so it’s values are slightly different and whatnot. But yeah. [Unintelligible 00:55:49] –

Moderator: But do you think there should be like different categories for different foods? Like there should be a different category for seasoning or a different category for like international foods and the users would add something, their recipes into those categories, and maybe the app could calculate those – not calories exactly but maybe the proportion based on the plate? Or some salty snacks? Yeah, there should be different categories I think, yeah.

Participant 1: Perhaps it can be categories of food items like meals, like cuisines and dishes, but not necessarily – because that’s something I used to have a lot of difficulty with, like the idea of your plates should be 35 percent fats, 35 percent carbs, 35 percent proteins, or like 30 percent carbs I guess. Like what is a fat? Because nobody is like – you’re not drinking olive oil down the drain, so like what is fat? What is protein? What is a carb? And in the case of the mashed potatoes with cream, well, cream is also a good amount of fats, cream is also a good amount of proteins and that’s going into the carbs. It’s like, what really are the percentages here? Like I feel like that question can be left to the food scientists who establish generally like appropriate value for each food item and then people will choose. People like even if, I don’t know, Participant 6 likes her mashed potatoes extra creamy it’s not going to be way too off the charts.

Moderator: Yeah. Yeah. Participant 2, I saw you were nodding, did you want to add?

Participant 2: I don’t know, this one’s a hard one. I’m trying to think like if I was eating out in a restaurant and say I had these mashed potatoes and I didn’t know if there was cream in it or not, I guess my question is how am I supposed to track something that I don’t know is in my food? If I don’t know it exists I can’t track it. And then I was thinking in my head like is this a me problem? Like am I supposed to try and figure out how much this restaurant is putting in, how much cream they’re putting into my potatoes so that I can track it or should it be something – like this is going to be a huge project if it ever happens, but if restaurants submit their recipes to this app or whatnot so the app you can say, “Oh, I ate this at this restaurant,” the app would know the recipe and know the nutritional value of that and then you as a user can just select that dish.

That’s too much work for me as a user to go and like try and pick out what is in my food, what is not. I think it –

Participant 5: I feel like –

Participant 2: Yeah, it’s not for me to do and I wouldn’t do it, so ...

Participant 5: I feel like that plays into the idea of the far future dystopian internet of things like everything is connected and you eat at a restaurant and your phone knows what you ate.

Moderator: Wow, that would be really awesome.

Participant 2: Yeah, I mean, I don’t want it to be like sinister or anything like that. But yeah, I guess my kind of idea was if it gets to a point where it’s no longer – like it’s kind of out of my control and it’s not – to me it’s not my responsibility to go digging for things that I don’t know about, you know? I think that’s not – to me, that’s not what food is about.

Participant 5: But perhaps one should also consider that like healthy living is a habit and it’s a strategy but it’s not – you don’t have to stick to it like every meal of every day. So if you’re eating out once a week, sure, like go for it and you don’t need to be really, really accurate about what you’re eating out. I feel like from a healthy living standpoint if somebody is eating out very often or if they’re ordering in, you know, Uber Eats, SkipTheDishes, all the time, that’s unhealthy to begin with. Because even if you’re ordering a salad from Mandy’s there’s like a million things in that salad; that’s probably your healthiest option. Sorry, I don’t know if everybody knows what Mandy’s is, it’s in Montreal. It’s a fancy salad place. It’s like for most things, most restaurant food is not necessarily healthy; it’s full of butter, full of sugar, full of salt, and all sorts of stuff.

Moderator: Yeah, totally.

Participant 5: Just like go enjoy life. Eat what you want every now and then but one shouldn’t be doing that. Like neither for their body nor for their wallet.

Moderator: Yeah, totally. Yes. Anything else to add? How to track the other meals that are not in the guide? Yeah, Participant 3, do you have any other ideas?

Participant 3: If I was thinking about like maybe desserts, specifically, if like you can choose – sorry, if you can choose like the shape of the cake and a way to like format – maybe there could be like a gage or something to show how much you’ve eaten of the cake just for that. Or like if it’s like cookies or other smaller biscuits or something like that you can input into like a dropdown box –

Moderator: I think AR – it could help, like AR putting your phone in front of the cake that you’re eating and it would visualize for you like what AR do I think – yeah, artificial –

Participant 3: Yeah, something like that or something close to that.

Moderator: Hmm.

Participant 2: I think it would be nice if, for example, I ate like a specific curry all the time, I think in general just like a thought I’ve had listening about this app, it would be nice if it was a little bit more personalized. Like how does – I hope I’m saying your name right, sorry if I’m not.

Participant 4: Yeah, that’s true.

Participant 2: OK. Thank you. I like when you put in like certain medical conditions you might be wanting to watch – like if I could – like that would be a nice way that I could personalize the app for me, maybe I could set a goal. Like I guess going back to my iron thing if I want X amount of – I want my iron numbers to increase by X number then I could put that in as a goal. I can put in my blood test, et cetera, and then these foods where it’s not in Canada’s Food Guide but I still want to track them, it would be nice if I could save a recipe or save – I think Participant 5 was talking about this a little bit, like save a meal profile so that instead of putting in all these little ingredients all the time I could just like select my meal and it would have all the nutrition saved there. So it would be, I don’t know, a better experience for me as a user using this app and it would be more personalized.

Moderator: Yeah. Right.

Participant 3: Kind of adding on to what Participant 2 said about like more personalized –

Moderator: Sure.

Participant 3: For me, since I’m like actually allergic to fish, I wish I could tell the app that like I’m allergic to fish so that they don’t give me recipes or anything that may contain fish or fish oil or anything closely related to fish that I can avoid that as well. And maybe I could also help the people who have more allergies, like some people are allergic to peanuts or nuts or like which other ones – like other allergy, like food relates allergies to help those type of people because sometimes they might be left out of that because of their own allergies and they might not be able to fully experience all that.

Moderator: Yeah. Great. So just Patricia added something on chat, I think our focus group will be a little over time. So yeah, just please feel free to leave the meeting today at 4:30 p.m. or 1:30 EST. But we would appreciate it if you could stay a little longer so that we go over all of the questions and it would be awesome. But feel free to leave any time, yeah. So the next question would be about how do you suggest dairy to be tracked, you know? Especially milk was not included in the guide we just had. Yogurt, there was not anything specific about milk. How do you think it should be tracked? Like chocolate milk, white milk, or even strawberry milk? How do you suggest tracking that?

Participant 2: I think personally for me, I wouldn’t want to like go to another place in the app like click a different button and then put in my dairy. Ideally, like personally, like my plate, I would want it to be representative of everything I was eating. If I was eating something like with a yogurt sauce I would want to put it on my plate. So maybe having more options than the three like greens, veggies, proteins. But I don’t know if that would help your goal though of following Canada’s food guides, which is not very – but as a user, that’s what I would want. I would want my plate to represent what I’m eating.

Moderator: Yeah, or do you suggest to try the meal [unintelligible 01:06:26] in your beverages? We’re going to talk about beverages as well. Do you think there should be another category for beverages or track meals over there, or just in plate, or even maybe you could even try meal as a protein for that? No? Yes? No?

Participant 5: I was about to say this idea might be used for other food groups as well, but using a visual cup and a slider like maybe like a really big cup and perhaps something most people can relate to, maybe like those big Starbucks cups and what not. And then as you slide the slider then they’ll double those up. And like visually might have a couple other things around it to like gage approximately how much milk that we’re actually inputting to the thing. And I was going to say as a similar idea, especially for eating out, say you ate, I don’t know, a cake outside, these like questions or sliders on the scale for like, “How sweet was your cake? Was it 1, 2, 3 4, 5?” If it’s 5 out of 5 then the app knows there was a lot of sugar in it and if it wasn’t as sweet there was less sugar in it. Like help the proportion.

Moderator: Yeah, I think there is a question about, yeah, how to improve the confidence when you’re tagging. Let’s get back to the eating out later and just focus on the preferences, how do you track beverages?

Participant 5: For most beverages, like Coke, Sprite, they come in either cans or glass bottles which are standard. Someone should be able to just add one or two or one.

Moderator: Yeah, sorry, Participant 2, do you think they should be in – they should be placed on this plate?

Participant 2: Oh, I think, sorry, if it’s a drink, I was thinking more of like a yogurt or a sauce that you’re putting maybe on your chicken. That is what I would want on my plate. If I was drinking like a late or something I think it would be nice to have a cup beside my plate that I could click on and I could, I don’t know, say what I’m drinking, I’m drinking a late, et cetera. Any other ideas?

Participant 6: Also I have seen when you try to order any hot or cold beverage, like in some apps they show how many calories are on there like right on the page it just shows how many calories are in your final drink but that doesn’t usually happen every time. So also especially if I want to – like I make smoothies in the morning, like I add my greens, and bananas, and everything, so I would want to measure how much I am adding if there is a simplified user-friendly way in the app just by clicking on the portion, like how Participant 5 said, in a class if I just add a tiny portion of dates and, you know, a big amount of Spinach or something, that would be helpful.

Moderator: Great. So what are – yes, you remember I think that there was some other elements in the food guide, like eating out, like eating with family or friends, like eating more mindful, be mindful of your eating, or be aware of marketing. Do you think – what other elements of the food guide should be placed in this app? Do you think it’s necessary, like to track some moods or feeling? Yeah.

Participant 5: I think if those – oh, sorry, go ahead, please.

Participant 2: You go ahead. No, you go first.

Participant 5: OK. OK. Sure. I feel like it falls under the idea of being mindful like what you’re eating because just because something is a vegetable or fruit doesn’t necessarily mean, “OK, I’m done with this.” The idea of balancing, for example, protein, sorry, vitamins especially, like when we’re grouping fruits and vegetables into one category if you’re eating just broccoli all day every day then you’re probably missing vitamin C. So the idea of diversity in what you’re eating and also probiotics, like things – for things to play into that.

Participant 2: Yeah, when I look at these – actually, I kind of have interesting feelings when I saw this page with Canada’s food guide. I think that – I don’t know, I like these prompts but to me some of them are just like – I don’t know what this means to me. For example, like be aware of food marketing. Like what am I trying to be aware of? Like is someone trying to scam me or is someone, I don’t know, someone says their food is natural but it’s not organic, or I don’t know. To me, some of these prompts I don’t know how – what does it want me to do like specifically? And it doesn’t tell me how am I going to track, for example, if I’m – for one of them, maybe like being mindful of my eating habits, like what does mindful mean and then how do I track if I’m mindful? Should I be like – I don’t know how to track that. Or even like, “Enjoy your food” should I be having like a little thing like every time I enjoy my food I give myself a tick and then at the end of the month it’s like I enjoyed half my meals? Like I don’t know what that means to me.

Participant 5: Wow, these are very good points.

Participant 2: Yeah, I think these are good prompts to have. I think this is maybe more to do with your mindset but in terms of how is that going to transfer to me using this app, like am I going to get a reminder saying like did you enjoy your food today? Like I don’t know what that means and I click yes but then also I guess it goes back to your goals. Like if I’m using this app to like track my iron to increase it, maybe like – I don’t know how to say, like enjoying my food, maybe that’s not my main goal for that time. I don’t need these reminders of asking me do I enjoy my foo and do I need to track that information?

Moderator: Yeah.

Participant 2: Yeah.

Moderator: So like for example, if you –

Participant 4: That’s a good point.

Moderator: Yeah, that’s a really good point. Also for example tracking your mood, like every time that you want to eat something, some emojis would pop up and you could choose your feeling in mind of emojis, something like this, do you think it could even be helpful being mindful about your feelings when you’re eating?

Participant 2: I feel like –

Participant 5: Yeah.

Participant 2: Oh, sorry. I guess my question was how do you know that your feelings are coming from your food? Maybe you were at work, you just had a meeting, it went really badly and you went to go eat lunch and you’re not feeling very good. Is it because you don’t like your food or did you just have a bad morning? And yeah, I don’t know, to me it’s kind of like it’s hard to pick out, like is this food making me feel this way or is it something else going on in my life that is making me feel this way? Like if someone really close to me passed away, maybe everything I eat for the next month will taste bad even though I really like that food it’s really good for me.

Participant 5: Yeah, and also like even for choices the idea of willpower, especially if you’re home like if you had a really bad day are you going to make a salad or are you going to go and fry some potatoes and like I don’t know – this is from a personal experience but like the other day work went really bad and then it’s just like, “Uh, I know I was going to make salad, that’s why I bought the ingredients and such, it’s like I don’t want a salad right now.” So the idea of willpower and mood. And also I was going to say eat meals with others, I don’t really understand what the health benefit of that really entails.

Participant 2: Yeah, when I look at this I think this is more of like a mindset lifestyle that they want you to have about food. But I guess each of these prompts will mean something different to another person. So me reading it, I have to understand that I need to make this personalized to me in some way and then I need to come up with my own systems to choose – to see whether I am meeting these prompts in the ways that I want to. So it requires a little more work on my end.

Moderator: Good idea. How about you guys? What do you think, do you think there should be these elements in the application as well, Participant 3 or Participant 4?

Participant 3: Yeah, I think it’s a good idea for this document to be accessible through the app, like inside of the app.

Participant 4: Yeah, me too –

Participant 3: Like more info sort of section. Sorry.

Participant 4: No, sorry. No, I was thinking and I was thinking how it can be helpful and it can be wrong in the application but I think it is really necessary to put the mood part in the application because for myself whenever I’m stressful or whenever I’m worried about something I eat lots of sweet foods and it somehow concerns me and I think if it has some option to ask me how I feel – but I’m not sure how it can help me but because my diet totally changes when I’m stressful, for example, I need something to measure this but I’m not sure how it can be helpful. Yeah.

Participant 2: Yeah, actually, I really like what she said, Participant 4, about – I guess I was thinking more is the food making me feel a certain way as opposed to I’m experiencing an emotion it’s going to influence my eating. But then my question would be like if I’m tracking that, so if I’m feeling bad an I always eat cookies whenever I feel sad, me knowing that information, is that going to change my habits? Every time that I feel sad and I going to be like, “Oh, but I also can’t eat cookies.” Like I think this is just tracking this information and seeing it is probably just going to make me feel bad but it’s not going to change the way I eat. I think would come from something else; that would not come from an app.

Moderator: Yeah, you’re right.

Participant 2: That’s [unintelligible 01:18:06] on it.

Moderator: Right. So what features in an app do you think – should be in the app that would help people to be more engaged when they keep tracking, be more engaged in using the app, what features do you think can be helpful? Like peer chat or support group or competitions or gamification? Yeah, what do you think can be helpful?

Participant 2: I have a question. I’m sorry, I feel like I’m throwing out a lot of questions more than comments.

Moderator: Fine.

Participant 2: For example, sticking to the app or like having these games or these – for example, games and competitions; I’ll just focus on these two. If I have a certain health condition and my friend has a different health condition, we are not going to be working towards the same goal. So a game or a competition to me wouldn’t make sense because we’re not going to the same – we’re not working towards the same place. I mean, I guess this is more personal, I’m not very competitive in anything that I do so I feel like if you’re competing in your diet then it’s – the focus is more, “I want to beat this other person,” as opposed to, “I want to focus on my health,” and I think this app should be more focused about an individual.

Moderator: Great. How about internal groups, internal groups in the app?

Moderator 2: Like for people, just to elaborate on it, like groups as in for people who have diabetes, they can have like a little community to I guess reach out to when they have any problems. So they have people who also live with diabetes to talk to you and stuff.

Participant 2: I think that would be really useful to have that support and community.

Moderator: Any other ideas? How to keep people tracking?

Participant 5: I don’t know.

Moderator: Yeah, like sharing photos of food, do you think it can be helpful?

Participant 5: I think for some people.

Participant 2: I think photos are just easy –

Participant 5: Oh, sorry.

Participant 2: Oh, sorry, you go ahead, Participant 5. I was interrupting you. I’m sorry. Please go.

Participant 5: The internet is a beautiful place but it’s also a really dark place full of bullying and not everybody has the same skills in terms of both cooking and plating and photography. So I don’t know, I can sort of see it becoming not as good of an experience for certain people. Like some people, probably like for myself, I would probably have a lot of fun with it. It’s like, “Look at what I made” whereas someone else is going to see that and be like, “My food doesn’t look like that. Oh I’m just bad. I’m stupid.” I don’t know, it can have a dark side I feel.

Moderator: Yeah.

Participant 5: It’s similar to how generally like our generation how we’re impacted by social media at large. Like do we want to introduce that to something that aims to keep people healthy?

Moderator: Oh, yeah. That’s true. So yeah, anything else to add?

Participant 3: I guess that like when I’m thinking about sharing photos of food, I’m thinking more like you would share it among your friends, right, and you can tell your – like a share a photo to your friends like, “Hey, look at this. I made this food and I think it’s really good, maybe you should try it some time. Try this recipe.” That’s what I’m thinking of like on the lines of sharing photos of food, I wouldn’t think of it more as like a very broad, like you can share it to literally everyone; I’m more thinking of more personal and you can share it to your closer friends and family and show that like maybe you found a nice combination or portions of food together and it would like suit someone’s diet or yourself and it works for you and has some – give some support to like other ones to see and try it out as well. That’s just my opinion on just like the sharing of photos.

Participant 2: I think it would be nice if – I’m just looking at this question to kind of stay away from photos because it says other things too, what features do you think could help with sticking with using the application. I think – so some sort of encouragement, like if I’m doing – if I put my goal as, I don’t know, I want X and I – throughout the month and I’m working towards there and I’m reaching half-way, it would be nice if the app told me something like, “Oh, you’re halfway there. Keep up the good work.” Or if I’m not sticking to it the app still encourages me and says like, I don’t know, just like, “You can do it. Add some vegetables to whatever, you can still achieve your goal.” I think it would be nice if the app was kind of cheering me on towards my goal. And then, you know, instead of people cheering me on because I guess some people can’t be nice, the app will always be nice to you.

Participant 5: Oh, that makes me think of – I don’t know if anyone used dual lingo but when you don’t use it they start sending you emails of their logo, which is the owl, and it’s crying. It’s like, “You made Duo sad.”

Moderator: Oh, yeah, I saw that.

Participant 2: [Laughs]

Moderator: OK, so how do you think what would help people to, you know, improve what features in the app could improve users confidence when they follow – when they are tracking their foods. I think the one that Participant 5 was mentioning before about eating out like it has something about it could – it could offer something about improving people’s confidence. Yeah, what do you think about it? What features could be used in the app?

Participant 5: Focusing on progress for the most part and accepting, welcoming approach to failures along the process as well. Being like, “Oh, you didn’t meet your goal this week, it’s OK.”

Moderator: Sorry –

Participant 5: Oh, sorry.

Moderator: No, no, you continue Participant 5. This is your – sorry.

Participant 5: Sure. The last thing to come into my mind was maybe also – I mean, this might be a little bit more complicated for AI logic but the idea if somebody is failing at certain tasks, maybe it’s not about them, it’s about maybe they shouldn’t be doing this, maybe taking a different route could be better. So like, you know, if they’re having difficulty lowering their intake of a certain food group and the app is pushing some recipes towards that person, like “Hey, I know this is what happened, maybe try this recipe this week,” or they’re not trying that recipe or they keep on failing that. It’s like having other approaches which are probably like best created by an expert or a scientist of sorts.

Moderator: Or maybe even if I add onto that I just thought of maybe just suggesting like smaller goals that would go toward their bigger goal in the end I guess, maybe, like yeah.

Participant 5: Perhaps. Trying to find like different ways towards the same process.

Participant 2: I think what would also be nice is I guess right now we’re thinking about it as the person who is using this app, they are also fully in control of their diet which means that everything that they put into their app and whatever they’re tracking is kind of like the individual’s responsibility when sometimes that’s not the case. Maybe someone doesn’t have the finances to go buy health food, or maybe someone is, you know, their parents always prepared their food and they’re not allowed to or at they don’t. So I think when it comes to confidence it’s – I don’t know how an app would do this but it’s just understanding that sometimes it’s not always the individual’s fault that they might not be eating the most – like not eating the Canadian food guide’s way or they’re not eating healthy for some reason. Because I think that if I was, for example, like if I couldn’t afford healthy foods and I’m using this app that I’m tracking it and the app is – I don’t know, I guess even if the app was saying, “Oh, you can do this and that,” like the app doesn’t know that I don’t have enough money to go buy vegetables and that would – you know, that would make me feel good and then I would stop using the app.

So I don’t know what – maybe the app can suggest like resources, like, “Oh, can you,” I don’t know, say you’re a UBC student and, I mean, I was never a UBC student so I don’t know who had access to what in UBC but if you’re a student and you have access to go see a dietitian or something if it could refer you, like, “Here’s the office address. Here’s the number,” so that you can – because I can app can only do so much. There’s a lot that goes into someone’s food in their diet. If they can refer them to a place where they could get the support that they need for their situation that would be better.

Moderator: Yeah, totally makes sense. Yeah, that’s great. So any other ideas? What could help improve users confidence? So in terms of accessibility, what do you think are required to ensure accessibility for all users in this application? Like, you know, you like fonts or colour or, you know, language even?

Participant 5: It’s going to have some general accessibility features that most apps should have for most people. You know, high contrast colours, bigger fonts, perhaps compatibility with their apps that read what’s on your screen for you, so like being compatible with that. Like contrast –

Moderator: How about for those who are in the minor majority, like people who are living with impairment? How about them? How to make it accessible for them? Or older adults? Or yeah.

Participant 5: Like bigger logos and not – like bigger things to touch on and such. But I feel like for the content of the app itself there is another layer of accessibility which is everything related to food, such as like eating disorders, allergies, and other things that people need to necessarily focus on specifically. Like people who do not consume meat and dairy products and whatnot, like vegetarians and vegans are more of a risk for having B12 – vitamin B12 deficiency. So being able to focus on those in the app, maybe iron, allergies, intolerances, dietary restrictions. And also abilities, like fish isn’t necessarily cheap so a lot of people, you know, you can’t go around having salmon all the time.

Moderator: Yeah, like cheaper recipes.

Participant 2: Yeah.

Moderator: What else?

Participant 5: Voice control for impaired individuals.

Participant 2: Or maybe having patterns for different – like the protein patches that are colours if someone is colour blind.

Moderator: Yeah. Interesting.

Participant 5: Yeah. I don’t know, there are experts out there whose whole job it is to think about this stuff. I know a lot of blind people use smart phones, which sounds very counter intuitive but your phone can voice out whatever you press on your screen. So it might be made compatible with that, the app. Yeah.

Moderator: What else? What other features do you think?

Participant 2: What about Participant 3 or Participant 4?

Moderator: Sorry, Participant 2, I didn’t hear you. What –

Participant 2: Does Participant 3 or Participant 4 have anything to add?

Moderator: Yeah, do you guys have anything?

Participant 3: I mean, yeah, like continuing off of what Participant 5 said about accessibility to people who have like allergies or like food allergies specifically and then how Participant 2 wanted to be more the iron and all that stuff to kind of make it usable to people who have a certain type of goal or just allergy details that the app knows that they’re not going to give them things that might cause complications in their health or anything like that.

Participant 5: I’m thinking, I don’t know how reasonable this would be, but I’m thinking a simple mode that might be better for people with certain mental impairments in which case like you want to add what you ate, you don’t get 20 different options about the specifications of what you ate. You get this one or two options and it’s perhaps that approach is that it’s better than nothing, it’s still tracking it. It’s using a very ballpark, very like guest estimate like very approximate value but it’s still tracking something. That could be an approach.

Moderator: Yeah, interesting.

Participant 5: So it’s not like, OK, eggs, and then you’re going to select is it poached? Is it sunny side up? Is it, I don’t know, hard boiled, soft boiled, this egg, that egg, is it a qual egg or duck egg or an ostrich egg? It’s like, no, what did you have? Eggs, that’s it.

Moderator: My God, I think you have a lot to do with this application, oh my God. I never thought about that. Yeah, oh, great ideas.

Participant 5: I also work in the tech industry.

Moderator: Oh, I see.

Moderator 2: That’s really helpful, so thank you for participating.

Moderator: Yeah, totally, they’re super helpful for us. We really appreciate your time over, yeah. So it would be – those were all of our questions. Oh my God, I can’t believe we’re at the end. So yeah, anything else that you see, that you can think of and we didn’t talk about? Any other features that you would add to the app? Yes, Participant 5?

Participant 5: Integration with some of the like leading very millennial food companies, things like good food and there are a few other ones. The ones that like send you a box of food that you can use for very specific recipe during the week, you know what I’m talking about? Yeah, they tend to have a lot of –

Moderator: Hello Fresh, right.

Participant 5: Exactly, Hello Fresh is one of them. They have lots of discounts and such and it’s everything you need for that mealtimes six in one box. So an integration with that would mean if you ordered a certain recipe from them than the app knows – and it can probably even pull up even right away like, “Is this what you had tonight?” So like yes and done it’s all in, you’re not selecting anything else. So an integration of that could make it very user friendly.

Moderator 2: That’s a good idea. Wow.

Moderator: Yeah, they are great.

Moderator 2: I just – sorry to cut in-between, just in the interest of time because I know we’ve almost been like 30 minutes over the time limit, I just wanted to let people know that you guys can leave whenever you want but if you had something that you wanted to say still please feel free to stay or even email us if something comes up later. And just to let you guys know that since you guys participated you will get your compensation – the compensatory gift cards within a week just to let you guys know, yeah.

Participant 5: Yay.

Participant 2: I do want to say when I first saw the app I actually was very attracted to it. I thought that – I know that we kind of picked on the simplicity of it but I think if someone is just trying to be more mindful of their – I’m going with that prompt inside to kind of – if you just want to be more mindful about what you’re eating I think that that would be a nice set up that is simple. Of course there are limitations to it with the mixed [input? 01:38:18], I just want to say that it is probably the most simple app that I’ve seen and I was attracted to it because I didn’t have to do a lot of thinking, so ...

Participant 5: Mm-hmm.

Participant 3: Yeah, first seeing the app I did like the simplicity of it and maybe just like – it’s not too simplistic but simple that like everyone can use. But maybe a little detailing would just like help just narrow it down so it becomes a little more personalized for everyone for themselves and all that stuff but that’s just probably like maybe a later problem in developing the app itself. But I do like the simplistic look of it and how it’s just fairly simple to use at first but then the detailing will for the next part will come later I guess in the app.

Moderator: Yeah.

Participant 2: And I think the [divisionalization? 01:39:08] is really a good idea. It was a creative idea. It was a creative idea.

Moderator: Great to hear that. Yeah, anything else? Yeah, your last words; your two cents on it.

Participant 5: One last thing for accessibility, haptic feedback, which is when you touch very minor vibration on the phone that lets you know that you actually touched something.

Moderator: Yeah.

Participant 5: I don’t know, it just came to my mind.

Moderator 2: Great. Yeah, love it. Also, if you guys were interested when we finally do develop the app if you guys were interested in actually being a part of the pilot study to actually download it and try it out please let us know or we will also send out emails to you guys to see if you guys would be interested. But that will be fine if you just ...

[End of recorded material]
