# Supplementary material for: Perceptions of self-monitoring dietary intake according to a plate-based approach: A qualitative study
Source: PLoS One. 2023 Nov 28;18(11):e0294652. doi: 10.1371/journal.pone.0294652 (PMC10683993; doi:10.1371/journal.pone.0294652)
Supplement: S4 Appendix — (ZIP) [file pone.0294652.s004.zip › Anonymized GP Transcripts/iCANPlate-GP-focus-Group-5.docx]

**iCANPlate-GP-focus-Group-5**

[Start of recorded material]

Facilitator: So this is the iCANPlate Focus Group with members of the general public on July 30^th^ at 4PM Eastern Time. So before I get into the first question of this focus group I just want to explain the concept of diet self-monitoring, since we’ll be focusing on that during this discussion group. So diet self-monitoring typically involves recording all food and drink items that are consumed in a 24-hour period. Those food journals are either paper-based or even on a phone application, and are returned to researchers or dieticians to analyze that recorded dietary intake of the day. And they are analyzed for a specific nutrient content or a technology base that include websites and smartphone applications, allowing individuals to track their food intake throughout the day. So does anyone have any questions about diet self-monitoring before I keep going?

All right, perfect. So the first question I have for everyone is pertaining to the Canadian Food Guide. So do you guys think it would be easy or hard to follow the plate method which is shown in the Canadian Food Guide? And what do you guys think makes it easier or hard to follow in accordance to it?

Respondent 1: I think it makes it easy. I started doing this kind of monitoring of my own food a while ago, and I think it’s quite clear on your plate if you do it like that, rather than – I have done diets and counting points, actually, not calories. I did Weight Watchers and it’s less restrictive, actually. And it’s common sense, and it’s easy to teach to children, without teaching fractions.

Facilitator: Mm-hmm, it’s very visual for the kids. That makes sense. Less restrictive.

Respondent 2: Can you share the picture again?

Facilitator: Yes, the plate. Yeah, for sure.

Respondent 2: I guess one of my problems with this is plates can be different sizes.

Facilitator: Mm-hmm, yeah, for sure. They don’t have like a specific size for the plate in the Food Guide.

Respondent 2: It’s easy to give a ratio, which is helpful. But a quantity as a gross number is going to be hard to judge here.

Facilitator: And do you guys find it difficult without have the specific portion sizes, like there was on the 2007 Food Guide?

Respondent 3: Actually I think – Oh, sorry.

Respondent 1: No, go ahead.

Respondent 3: Actually I think it would be different for different kinds of people, because, well, some people exercise more than others and develop their needs. I think more proteins. And, for example, some days that I, myself, exercise, I really rather to consume more protein than the other days. And so I think maybe it’s kind of hard to follow just this picture.

Facilitator: Yeah. So do you feel it’s very generalized then?

Respondent 3: Yeah, I think it’s so general, and it’s not specific for everyone.

Facilitator: Yes, [Respondent 4]?

Respondent 4: So sort of in the same line as that, I have some really rare and weird health conditions so I have lots of dietary restrictions, hence loving the lentils. I have to love the lentils. So something like this I think is good for a really wide population, but for someone who is being asked to make very, very specific choices or have allergies and that kind of thing, this can sort of maybe not be – I mean it’s helpful to give sort of a baseline but it’s not really helpful in: how do I build the diet that I need based on this, and then all the restrictions that I have?

Facilitator: I see. It’s very, like I said before, very generalized –

Respondent 4: It’s broad, yeah.

Facilitator: Yeah. And that’s not specific for people who need to have a certain type of diet, for sure, yeah.

Respondent 4: Well, for instance, a doctor will say you need to sort of avoid – well, an easy one, you need to avoid lots of carbs. I don’t know where that is in this picture. I know where they are because I know what carbs are, but those instructions you get from health practitioners, they’re not using sort of – well this is very simplistic. So yeah, they’re sort of missing a level of detail here if you’re being asked to do specific things with your diet.

Facilitator: That’s true. Because over here on the fruits, like the apple could be a carb. And over here on the grains, the bread and the noodles could be a carb. So carbs are pretty much all over the place.

Respondent 4: Yeah, but sugars is tricky too. You’d think there’s not a lot of sugar, you know, I just avoid fruits but that’s not [a sure bet? 00:05:49] either and that’s not a good thing to do. Just some really sort of big health things a lot of people have, sort of you don’t get any tips in the guide here for those big things, like cardiovascular, diabetes. The big ones that tons of Canadians deal with, they’re not getting any cues here.

Facilitator: That’s interesting to note. Thank you so much for that. Does anyone else have something to add on to that? [Respondent 5], do you have anything to say?

Respondent 5: Well I agreed with [Respondent 3] when she said that – you know, I feel like the diet – like everyone’s diet is going to be different. Because, yes, I too workout, and personally I need to have way more protein than what it says here with my goals. So I guess your diet will also differ with a person’s goals and needs too, right. And, yeah, it’s just really broad; I wouldn’t know how many grams of protein a person should have per day. Like, is this for children? Is this for adults? Is it for everyone? I don’t know. It doesn’t really tell you, right?

Facilitator: Yeah, for sure. The one poster is very, very vague, I guess, you can say for – That’s what I’m trying to take away from everyone.

Respondent 5: And this is – Sorry, this is what they released as the Food Guide, the new one?

Facilitator: Yeah, in 2019. Yeah, they release it every about like 10 years I think.

Respondent 5: Well, I can’t believe this is literally it.

Facilitator: Yeah, that’s pretty much it. I think there is a website version and there is way more detail put into it, to the website. But yeah, this is the official one, just like the two-sided poster, yeah.

Respondent 4: Oh, this is not the cover of a book that has lots of info inside? This is it?

Facilitator: Yeah, it’s a one-page –

Respondent 4: Oh, wow. [Laughs]

Facilitator: But if you go to the website there is a lot more, kind of like – it would be similar to the amount of information you found in the giant booklet we had back in 2007. But yeah, so building upon that, since you guys were talking about your different diets, if you were to make changes to your diet do you guys have any techniques that would help you?

Respondent 5: I have one. So every day I need to have a certain amount of calories, and in order to do that – since I don’t have an appetite really – I started to wake up earlier to eat a meal in the morning so that I would have the calories I need per day. So if your goal is to try and gain weight or muscle I think a really good strategy is starting to wake up if you struggle with eating every day, like eating enough food. Because once you start eating in the morning you can snack throughout the day, like a healthy snack, and you know, just try to get in the calories.

Facilitator: That’s so interesting. There was another person in our focus group before and they wanted to do the opposite to avoid eating more, they slept earlier in the day so they don’t have to eat at night.

Respondent 5: Oh.

Facilitator: But yeah, is there anything else that you guys wanted to share with us, besides waking up earlier to eat more? Yeah, Respondent 4?

Respondent 4: Certainly making your own food, which is just a big lifestyle, but making that change really gives you tons of control as to what you’re eating. So the move towards preparing your own food gives you tons and tons of control over what you eat. I would definitely recommend that to anybody who needs to look at what they eat, and carefully. I mean it’s labour-intensive, but it gives you the most granular control over things.

Facilitator: Yeah. Especially, like you said, that you had to change your diet for medical reasons, so that gives you a sense of security in knowing what you’re eating.

Respondent 2: Yeah, and I am kind of the opposite of Respondent 5. I do intermittent fasting, so I won’t eat generally until like 12, one o’clock in the afternoon, and then just have two big meals in the day. And that’s easier for me to partition my calories and macronutrients.

Respondent 1: I just started doing that on the advice of my doctor, because I’m on borderline diabetes and I have been for four years. And my diet is squeaky clean, I make everything, I hardly ever eat out, I buy nothing prepared, and he – As a last resort just two weeks ago he said, “I don’t know a lot about it but I’m going to refer you to a nutritionist, but in the meantime read about it.” And so I did, because I thought I don’t want to take medication, I already have a cholesterol issue. Anyway, so I read a lot about it. And I’m sure you know the guide there, [unintelligible 00:10:45], it’s a wonderful resource.

Yeah, and anyway I just started doing it. Oh, I think I just started last week, at the end of last week, so it’s been almost a week. And it’s so easy, and it’s so easy for exactly what you just said, [Respondent 2], because I am so fine with not having breakfast. I am active in the morning, I take exercise. I never thought I could do it. And I eat my first meal at 11:15 and my second meal – my husband is really willing, is very supportive, so normally we eat a bit later. So I’m eating my second meal at 6:15, I can’t believe it.

I have a question about the Food Guide. Where is dairy? Like where is the Greek yoghurt that I have always been told is so great for me?

Facilitator: There’s actually yoghurt in the protein section, but there is no dairy food group in this food guide. But yeah, thank you so much for sharing all of your perspectives on how to change your diet. So I was just wondering, since a lot of you have been trying to monitor your diets based on your lifestyles, were there any applications that you guys have used to track your diets before?

Respondent 4: I have been using my MyFitnessPal for a long time and it’s pretty reliable. You have to make sure that the items that you’re using are verified, or that you’ve checked them, because there’s all sorts of stuff there. But once you have your inventory there it’s been super, super helpful. You don’t have to do all the math in your head, it’s just all there. And stuff like iron which is hard to track, it’s just there. So, yeah.

Facilitator: So you find it easier to track your iron, for sure?

Respondent 4: Oh, that was just an example; I don’t have to track that. But just stuff that tricky to know what the levels are. How much of this, how much of that in different foods, the app just takes care of adding it all for you.

Facilitator: Do you find it easier to use, rather than just writing it all down?

Respondent 4: Well, writing it all down doesn’t give me sort of: Did I get enough vitamin A today? Did I get enough protein today? I’d hate to have to do that manually, so yeah, the app is – it’s everything for me.

Facilitator: Has anyone else used any phone apps to track their diets?

Respondent 2: [With joy? 00:13:42] I use MyFitnessPal, along with a food scale, and I just kind of weigh everything out. It’s a breeze just to – it does it all for me.

Facilitator: Perfect. So I was just wondering if [Respondent 1] or Respondent 5 or Respondent 3, have ever used an app?

Respondent 1: Yeah, I used the Weight Watchers one and it was good for a time. It was helpful because you didn’t count calories, it was a point system, and fruits and vegetable were free, and eggs, and tons of things. So as I said, I am astounded how easy I find doing this, because I am retired and so I have a pretty predictable schedule also. And I don’t tend to eat late in the evening because I don’t like to, but I cut out snacks in the evening and I am just so surprised at how – And it doesn’t cost anything, so for that too it’s quite remarkable.

I don’t know how it’ll go at Christmas, you can call me and I’ll tell [Laughs]. But this is my last ditch effort to stay off medication. And it’s in my family, the whole – it’s not a –

Facilitator: A genetic thing.

Respondent 1: Yeah. So that’s the only one I have tried.

Facilitator: Yeah. Thank you for sharing.

Respondent 3: Actually I haven’t used an app for my diet, but I have used different kinds of apps for different kinds of purposes, and so I think maybe I have to start using an app for my diet as well.

Respondent 5: Yeah, I also don’t use an app. But I think I would like to start using it just so I can keep track of my macros better.

Facilitator: Perfect, nice to know. Thank you guys. So the [unintelligible 00:15:20] on like phone applications, specifically have you guys like seen or know of any diet self-monitoring tools that look like the Canadian Food Guide, like specifically the plate? Have you guys ever seen any self-monitoring tools that look like that?

Respondent 2: Not really.

Respondent 5: No.

Facilitator: All right. Yeah, I figured, because most people say that. But just in case people actually did know it would be good to be notified by that. But thank you. So now that you guys have seen the little prototype of the application, we were wondering how you would view the application working so that you could record all your meals throughout the day. So considering your meals like breakfast, lunch, dinner, and even snacks, how do you think you would imagine seeing the application when you record your meals?

Respondent 2: I think it would be awful, because what if you didn’t have grains and everything? I mean, it’s reducing food to categories you may not fit into. Like what [is bacon for? 00:16:34], fat and protein, but if you just want protein a chicken breast gives you much different nutrition density than that. I don’t think it accounts for all the other things.

Facilitator: Yes, Respondent 4?

Respondent 4: Yeah, certainly if you’re following fitness goals, or if you’re following health goals, the three sections there are – I don’t want to sound mean but they’re really not helpful [Laughs]. If you’re somebody who has a very poor diet and the main goal is: We need you to eat a lot more fruits and vegetables. Like some people are just eating meats and breads and that. So if the goal is simple like that, to just get you to eat more fruits and vegetables, then this could be good because it’s making sure you fill that half-plate with your day. But if your dietary goals are any more sophisticated than that I don’t think this is going to be helpful.

I also don’t really know, is that like – the round, is that for your day or is that for your meal? And do the pies fill up as you add foods? I totally don’t know how that works.

Facilitator: It’s per meal. So if you just had, I don’t know, mostly pasta, your plate would mostly look like a grain; so it would be like an orangey colour instead of –

Respondent 4: You’d enter sort of “I had pasta” and then the app knows to sort of fill that part of the pie, is that how it works?

Facilitator: Yeah. So shown in kind of like the video, the walkthrough, you could actually adjust the size of the different colours. So you would see – If you had more grains you could kind of swipe up on the yellow or the orange for the grains, and then there would be a lot more grains in the plate.

Respondent 4: OK, so the user has to know what they’re eating and know which button to add, and know how much content – what they just ate. Yeah, that’s super complicated, isn’t it?

Facilitator: Yeah, pretty much. Yeah.

Respondent 2: I think it also doesn’t take into account the height of what you’re eating too. I mean you could just grab a scoop of rice, super thin, or you could have a big clump of it.

Facilitator: That’s so true. I just realized that now.

Respondent 2: Like how [do you? 00:18:47] you take care of that?

Respondent 4: Someone who weighs their food would know that really well, hey, Respondent 2?

Respondent 2: Yeah.

Respondent 1: But I agree with what Respondent 4 said, and I agree with what Respondent 2 said. But for someone who is not eating well it’s a good start to say you have to have to have – like the way when you read it’s important to have colour on your plate, have a variety. So just knowing that, it really guides you. I mean the people who are sitting here are probably all pretty health-conscious too. But I mean I can think of tons of people who [just eat? 00:19:30] meat and potatoes, that’s not unlike how I grew up probably.

Facilitator: For sure. Do you guys have anything else to add about how you guys would view it, or how you guys would not want to view it, I guess?

Respondent 4: I guess I could share – certainly the environment in which I grew up was not at all food-conscious, so when I started taking care of my health a lot more, and having to do so, and having to track my food, and learning about food, everybody checked out on that or were like: “Wow, that’s complicated. You’re a big nerd. You like reading so it’s OK.” But what I’m hearing basically from them is that is way too much work, it’s way too much information for them to learn.

But also, like every time you sit down and have a meal you need to get into that ritual. So if you really have to be or like to be healthy-conscious then that’s fine, but I got to say there’s not a lot of people in my life that are like: “Yeah, I totally want to sit down at the end of every meal and write stuff down”, like nobody wants to do that. And nobody also wants – And if it was just: pick a food, like see it on a list, I had some apples, I had three slices, tap, tap, tap, that’s one thing. But right now you have to know what you ate, you have to know what category it’s in, you have to know how much you eat. There’s no way, I could never get that gang of people which is my family into this project, not at all, not right now, not with that setup.

Facilitator: Yeah, so I guess [unintelligible 00:21:02] people have to have a certain amount of nutrition literacy to be able to fill in the meal, the plate, on the app.

Respondent 4: Maybe designing it sort of for a ten-year-old might be actually a good lead to make it easier for everyone to use it. To make it very visual and to make – How many slices did you have? And show the slice, rather than how many grams of meat did you just eat? I mean people [unintelligible 00:21:45] know that.

Facilitator: Yeah, for sure. How about Respondent 3 and Elise? Do you guys have anything that you wanted to say about how you would view the application?

Respondent 3: I actually do. And I think it’s more useful for children because it really makes it easier to explain to them that you, for example, have to eat more proteins and your colours should be more pink, or something like that. But it’s not really going to work for adults I think, and especially for those who are really concerned about their diet.

Facilitator: Yeah, for sure.

Respondent 5: Yeah, I also agree with Respondent 4, because I feel like it’s a little bit generalized. And yeah, it would be helpful for children to learn from that kind of pie graph thing.

Facilitator: For sure. Thank you. So when you guys want to represent the different meal portions on your plate, how do you guys think you’ll go about doing that? So what types of references, I guess, would you think of when you want to fill in that plate? I guess maybe an example that I could think of is measuring, by defining measuring, like standard measures. So like a standard plate, or a standard bowl. Maybe even using your finger or your hand as a reference for when you want to represent the meals.

Respondent 1: My understanding is that your meat shouldn’t be more than the palm of your hand. That’s what I’ve just understood. But when we eat it’s like – That’s why I like that graphic, because for us it really is – not even 25% is protein, meat. It’s really, like more than 50% is salad and vegetables. But like 75% is lots of water. So no bread, we don’t have bread with meals, which is quite a dramatic difference from even 10 years ago. Again, you’re speaking to the choir, preaching to the choir, so I don’t know.

Respondent 3: Actually when I was younger I would do this hand thing, but now really I’m more comfortable with counting calories, because I think I can have more control in what I am eating. And so I really am more comfortable with that.

Facilitator: Sounds good. Thank you. Oh, sorry. Respondent 2, go ahead.

Respondent 2: Yeah, for me, I just measure everything in grams. Anything I eat, I put it [on the stove? 00:24:54] and just start putting things on. And I had a rough idea in my head about what was [unintelligible 00:24:58] will be for me, so I kind of just do it that way.

Facilitator: Yeah, and it’s easier to know how much of what you ate in relation to each other, because you have it all in the same measurement.

Respondent 2: Yeah, and it’s always accurate too, so how do I perceive the size of my hand [unintelligible 00:25:13]? Or whatever. Here is my number.

Facilitator: Sounds good. How about for Respondent 4 and Elise?

Respondent 4: I’m not extra clear on what you’re going for with the question. I’m sorry.

Facilitator: No, that’s fine. It’s a very confusing question. So when you want to represent the different portions of your meals what would you kind of use as a reference to show like how much of it that you ate?

Respondent 1: I can add something else to the question to be more clear. So the application has not been developed yet. It’s just like a mock-up of the application and we definitely need much more work to do on it. So we are asking, when it’s developed we are going to measure your portions to, you know, measure those proportions and the plate method. So what do you think is the best way to measure those proportions? Like maybe weighing – the weight of the food that you’re having? Or like using your palm as a reference, or using cups or a glass, or a tablespoon? What references can you think of that could be used for measuring that portion?

Respondent 3: I think weighting it, because it’s more consistent that way.

Respondent 2: Yeah, like a cup of spinach could be very different for different people [unintelligible 00:27:14] [how packed it is? 00:27:15], right?

Facilitator: That’s true, yeah.

Respondent 2: So I don’t really see it as a very effective way to measure it constantly over time.

Respondent 4: I think there’d be a big advantage to using photos, visuals. So if it’s a cup of spinach, but have something like a pencil next to it so that people can really gauge what the sizes are. But I’m thinking of parents with many kids, there’s no way they’re going to be weighing foods; you know, that’s not how life works. I think the general population’s not going to do that either. Like if we’re trying for this app to be really, really used, like widely, especially for people who are [adult? 00:27:49].

And Respondent 1’s point was right, we’re all I think a little group of really health-conscious people that are tracking, already do this, so I don’t think we represent at all the general population. So if the app had photos of different items that people can just tap, and tap it three times if you had three times that much. Like three slices of apples and tap that three times if you had a full apple, for instance. That makes it a lot more user-friendly.

We can ask people to do weighing and that. I mean there could be maybe two levels to the app. But for the general population, I don’t think it’s realistic to be asking them to weigh stuff, to know what [weighs stuff? 00:28:31]. And even the palm method is great, but if you’re getting proteins like me, not from meat, that reference doesn’t work anymore. It doesn’t work with eggs either. And if you’re watching your cholesterol, doing it for eggs doesn’t work anymore.

So those are a big old-school, those methods, and I don’t think they’re universal enough to be used as like – My best bet is photos at this point for an app like this.

Facilitator: Makes sense. Thank you so much for sharing. And thank you, Respondent 1, for elaborating on the question, it was a little bit difficult for me to word it properly. So there are many other foods that are not shown in the Canadian Food Guide, which I think some of you probably would have noticed. So which foods can you think of that you would find difficult to represent on the plate?

Respondent 2: Any meal, really.

Respondent 4: Can we see the photo again?

Facilitator: Yeah, for sure. And how do you think they would be tracked on the app? Like foods that are not included in the Canadian Food Guide. And how do you think they would be classified within the application?

Respondent 4: Yeah, take something easy like spaghetti sauce, like where are you going with that in this? Or a stew. Or, I made a chicken soup. You know, stuff that you would regularly eat. You have to know what went into it. It’s fine if you’re the one cooking it, but if you’re not that’s a big task to sort of undo what was – what went into that stew. And a cream of vegetable soup, then you have no idea what was in there other than vegetables.

Yeah, I’m going to keep strumming that string of the photos there I’m afraid, throughout this discussion.

Facilitator: Yes, for sure. Besides milk or any dairy products can you guys think of any foods that you would find difficult classifying in this plate method?

Respondent 3: I think peanut butter is one of them, because I know that it has lots of protein, but it also contains [unintelligible 00:31:04] kind of things. And I think maybe it can be a little bit hard to classify it.

Facilitator: For sure. So I guess on that topic of those other foods then, there is also chocolates, candies. And like Respondent 3 said, there is butter. There is a whole bunch of different foods that are not part of the Canadian Food Guide. And we were wondering how you guys would think it should be, I guess, tracked in this phone application. Since we plan to have the app go kind of beyond the Canadian Food Guide but still be kind of like the basic [concepts? 00:31:51] based on the Canadian Food Guide.

Respondent 4: So do you want the app to be tell them or –? It depends if we want the app to be telling them: these are good things that you’ve eaten. Like track the good stuff that you’ve been eating, and to hit your goal. Or versus, we also need to – Like if you’ve been eating sugar all day, do we want the app to flag that at some point and be like, OK, you’re way too far off one end.

So it depends what we want to be doing; like positive reinforcement or negative reinforcement with the app?

Facilitator: Yeah, we haven’t considered reinforcement from the app yet. We were just thinking it was a good tool to use for people to just track everything they’ve eaten in a day, not necessarily for I guess reinforcement.

Respondent 2: I mean, where would something like pizza go? That’s kind of like half protein and then half whole grains.

Facilitator: That’s true, yeah. For sure.

Respondent 2: There’s not really a thought for fats on here either. Does that [unintelligible 00:32:49] with protein?

Respondent 1: Yeah, it’s depending on what kind of pizza too, it can be quite healthy. There’s gluten-free. A thin crust or a thick crust. There’s a huge difference in that. But also, I would think that treats, like pies or cake, maybe it could go in a – like not too many of this section, or like a treat section. You know? I mean if that’s something that’s important. It’s important too, like birthday cake and ice cream. You can’t say “don’t have any”, but I guess in moderation. And that’s a tricky one, sort of to say, OK, have you had four treats today or one, or two? And also what’s your size?

Respondent 4: The size of the treat, yeah.

Respondent 1: Yeah, so a triple scoop or a frozen yoghurt? There are so many variables.

Respondent 4: I have certainly found it useful in other exercises, not with the app that I use now, that to identifying foods that were not giving you really anything nutritionally-speaking. So it’d be interesting for an app to be able to tell you: This food or this meal that you had, you had tons of calories that really did nothing for you but just give you calories.

It might be interesting to learn which foods take up a lot of your plate, but are actually a low nutritional payback.

Respondent 1: Yes, and maybe at the end of the week you could get a report card, or some kind of tallying up. It’s a tricky one. Good luck with that.

Facilitator: Yeah, for sure. Do we have anything else that we’re missing from this conversation, Respondent 3 or Elise? Anything that’s different, that you wanted to add? Or you pretty much just agree with what has been discussed?

Respondent 5: Yeah, I don’t really have anything new to say. I do agree with what everyone’s been saying.

Respondent 2: I feel that some of those mixed foods would be asked to kind of deconstruct them even.

Respondent 5: Yeah.

Respondent 2: Like a cheeseburger, it could be a huge bun. Or [unintelligible 00:35:19] or whatever, like it doesn’t fit into one category unless you break it down.

Facilitator: Yeah, especially like maybe even stews too, that would be very –

Respondent 2: Yeah, soups and that.

Facilitator: Very complicated.

Respondent 1: Yeah, and spaghetti sauce if it’s made with lots of vegetables, or let’s say lamb meat as opposed to lean, lean, lean, or turkey. You know? When it’s turkey. And there is also the importance of water, but I think you put it on there.

Facilitator: Yes, so to make things even more complicated, how do you guys suggest tracking beverages in the applications? Because there’s more than just water, for sure.

Respondent 4: I’m glad you brought that up. So when the Canadian Guide came out last year, two years ago, 2019, was it?

Facilitator: Yes, 2019.

Respondent 4: OK. So I come from not a very healthy family, and there’s lots and lots of alcoholism on both sides of my family, so I was so disappointed when this Canadian Food Guide says: Yeah, you can have one alcoholic beverage a day. And I was like, oh, why are we putting that in there? Because I mean that’s the tune that my family’s – Well, at suppertime we’re going to have wine. Yeah, yeah. So somewhere, somehow they’re getting this message: Well it’s in the Food Guide. It’s not: We’ll tolerate a bit of alcohol during the week. It’s: You can have one a day. And that’s a downhill slope.

So I’m always very surprised at that that keeps staying inside the Food Guide. So if we are tracking beverages, tracking alcoholic ones are probably a really good idea. Alcoholism is a pretty prevalent problem, [I think? 00:37:20] one in ten Canadians have an alcohol issue. So that might not be a bad place if we’re going to ask people to track their beverages, to send a little flag up. [We’re like? 00:37:30], OK, enough with the booze this week. When you’ve hit your limit that probably wouldn’t be a bad idea. There’s lots of calories in alcohol as well that are totally –

Facilitator: Yeah. Yeah, so like I guess keeping that in mind, do you guys think that beverages in general should all be classified with water, or do you think it should be classified as like another food? Because there are those beverages that have more nutrients than water, or different types of ingredients than water, like juices or shakes and stuff.

Respondent 3: I think there should be a section for sugars, because there’s so many beverages that have sugars. So I think maybe they should be classified as beverages with sugar, or beverages with alcohol, or a different kinds of other things.

Facilitator: Yes, for sure. Can anyone else think of any other beverages that you think should be, I guess, talked about when talking about beverages and classifying them into a different category? Maybe coffee, because coffee’s a big thing now, or even energy drinks. Gatorade, that type of drink.

Respondent 4: I think all of it should get tracked. I mean some of them – Cumulatively some of them are not good. Lots and lots of coffee with lots of sugar and cream, not good. Lots of juice, not good. Lots of energy drinks, not good. Everything is good in moderation, but we were raised in a house where juice is fine, it’s fruit, go ahead, drink. Like have as many fruits and vegetables as you want. That’s actually not true at all for juice; that can be a lot of sugar throughout the day.

Facilitator: Also something that was home-made, like juices, like without – like as like extra sugar. But yeah.

Respondent 4: I think beverages should be tracked just like every other food, I think they all pack a punch, good or bad.

Facilitator: Yeah, so no one has anything else to add? I will go on to something more specific about beverages, and go into the topic of dairy, because it’s been mentioned a few times before in this focus group. So how do you guys suggest that dairy – specifically like milk – should be tracked on this application? Do you think it should be in its own category? Should it be within proteins? Do you think it should be under beverages? What do you guys think?

Respondent 1: It’s pretty complex. Different kinds of cheese. Different kinds of cheese. Is chocolate milk dairy? Is high-fat yoghurt? You can get really high-fat yoghurt, or zero percent. It’s just so, so complicated. And ice cream, where does ice cream –? My beloved ice cream; where does that fit? It’s like my favourite thing in the world.

Respondent 4: I miss it too, Respondent 1. I miss it too.

Respondent 1: It’s the cruellest. I like sherbet, and they have these other ones now that are –

Facilitator: [Gelato? 00:41:12]?

Respondent 1: Yeah. Don’t waste your money on that. But there is one that – I can’t remember it – Chapman’s. And I was so excited because it said sugarless and dairy-free, but you look at it and it’s full of calories. And it’s very tasty.

Facilitator: It is. It’s very tasty.

Respondent 1: Put that in your shopping cart and you think, “This is perfect”, and you get home and you think, “My goodness.” Not to mention all the problems with all those sugar substitutes.

Facilitator: Yeah, for sure. There’s a lot of sugar –

Respondent 1: I’m getting hungry here; it’s almost suppertime in Montréal. I’ll be quiet.

Respondent 4: Might I ask, my impression is if it’s not on that plate you shouldn’t be eating it. Is that the right interpretation to make about –? Certainly the cheese lovers that I know are like, “What do you mean it’s off the plate? What does that mean?” Should we not drink it anymore, or should we not have dairy anymore? Actually I had never looked into that and wondered what the – That’s a big pretty big turn for the Food Guide, to point those off the map, right?

Facilitator: Yeah, so they’re not necessarily saying to completely remove dairy out of your diet, especially since in 2007 I remember dairy being like its whole own food group, right. I think they were –

Respondent 4: Oh yeah, that’s right.

Facilitator: Yeah, I remember that as a kid. But I think I’m not entirely sure why they completely removed it; I think it’s just a thing to – I think because people thought that I think the dairy – B.C. Dairy, or like the dairy farms were associated with the Food Guide. I’m not entirely sure, I don’t know how to disclose that because I don’t want to disclose any [mal-information? 00:42:54] to you guys. I cannot answer that question at the moment, but definitely email us about that and we will definitely look into it and answer your question about the removal of dairy about the Canadian Food Guide.

Yeah, does Respondent 2 or Elise or Respondent 3 have anything to say about how you guys would want to track dairy products or milk in the plate?

Respondent 5: I personally would just – Sorry.

Respondent 2: Go ahead.

Respondent 5: I personally would just add it to protein, just because I don’t really prioritize my dairy, I just think of it as cheese to my meal, to just add a little bit of calcium.

Facilitator: Yeah, for sure.

Respondent 2: Yeah, I rarely ever have dairy anymore, other than cheese. I have replaced cow’s milk with almond milk for everything, pretty much. But yeah, I guess protein would be the closest thing to it.

Facilitator: Definitely, milk alternatives have been a really big thing as well. So as I showed you guys the Canadian Food Guide earlier I talked about the back side, which actually had a lot of qualitative elements about food, not necessarily pertaining to what you should eat, but stuff that’s associated with eating. And we were wondering if that kind of stuff should be added to the application. So you know, how to talk about or track mindful eating, or eating with your friends or your family, and if you have any goals with your diet that you want to talk about or track? Or even like mood tracking as well, like before or after you eat.

Yeah, it’s a very difficult question to answer.

Respondent 2: Yeah, it’s hard for me to say because I – just generally one of my big meals is just a smoothie full of all kinds of healthy stuff in there, and I don’t really get any pleasure from that. I don’t like cooking, I don’t like any of that stuff, I just – nutrients can get in there, done.

Facilitator: Yeah, [unintelligible 00:45:25].

Respondent 2: I know, but a lot of people love the family sit-downs, especially like a big home-cooked meal with good feelings and everything. It’s hard to track.

Facilitator: For sure, yeah.

Respondent 4: The back of the guide also says stuff to avoid, like saturated fats and all that. Is that [correct? 00:45:49]?

Facilitator: To minimize it. Yeah, to minimize it.

Respondent 4: I’d be more tempted to make sure that the app is capturing and flagging people when – you know, high in sodium, sugars, like if the app could flag those, I think that’s where a lot of health problems arise, there. And if the point of the Food Guide is to help people steer clear of long-term health problems I think the idea to be like: “eat more fruits and vegetables”, like that general push is good. But the app being able to flag and say, OK, you’re like, this day was full of sodium and probably a good idea.

Facilitator: Yeah, [unintelligible 00:46:48] –

Respondent 4: And maybe, you know, get little points for: Yes, I cooked a meal today, or yes, I had a meal with [unintelligible 00:46:54]. You know, add up little points that you get at the end of the week, that’s probably a nice motivator.

Respondent 1: Yeah, that’s good. And also if they had an easy link, too. You know, there cookbooks, like one-pot meals. There are really easy things to make, I know because I cook all the time and some days it days me a lot longer than other days. And there are so many easy things to make your plate interesting, you know, adding things like sprouts, or just colourful meals. And if they had something – Every so often they’ll do that in the newspaper, they’ll have recipes to make with your kids. I used to make my kids’ lunch with them because I couldn’t stand making them, so we made it into a game.

So if they had a link to easy recipes for people who like chicken, let’s say, or people who liked whatever. You have to kind of give people the tools. I don’t think everybody – I think people are really confused. Now at the grocery store so much of it is already premade meals; if it’s a huge section you don’t have to make anything. And things that are easy, like shepherd's pie and lasagne, these are not complicated things to make, and they’re very affordable. Like you look at the price and you think, "I can’t make it for that."

Facilitator: Yeah. For sure.

Respondent 4: It might be interesting to run a couple of your sessions with parents, explicitly with parents with kids under 15, I think they’ll have very pointed perspectives about what’s feasible, what’s realistic. And you can do another slice with people who are low-income, and the challenges they face: I want to eat well but, you know, like I work, you know, 50 hours a week, and I work night shift, and I have three kids. These are the challenges I think that get in the way of people wanting to eat well but not having the tools, not having the time, not having the resources.

So all of this kind of structure for them is frustrating because they can’t engage with it. It’s not made for them, it’s made for us that are like, “Yes, I know exactly what’s in my food, and I watch my food, and I’m conscious of –” But like I said, I don’t think we’re representative at all, of the majority of the Canadian population.

Facilitator: Thank you so much for keeping that in mind and always bringing that perspective in.

Respondent 2: Yeah, good point.

Facilitator: Mm-hmm, very good point. But yeah, are there any other elements of the back side that you guys think should be shown or reflected on the application?

Respondent 1: Well, it would be great if they had something like Respondent 4 was just saying, a link to community kitchens where you could go, you could go and cook a meal. They have that sometimes in lower-income areas where you can actually go, you learn how to make something, and you leave with something made. That would be such a great thing, if they would do that.

I mean I know it’s done, I know it is done, but if it was more sort of not even necessarily because you don’t have the money. I mean if you were well-off you could make a donation to it. But that would be nice if the –

Respondent 4: That would be brilliant.

Respondent 1: Yeah, if the Canada Food Guide would sort of put their food – What am I going to say? Put their money where – You know what I –? [Laughs] I could make a joke if I had a few minutes. But I think that would be so nice. And I mean people like me, like I have a lot of experience in the kitchen, I had four kids and we were – I was a stay-at-home mom, I learned how to cook because that was what I did, and I liked it, and I’m – But [there’s not? 00:50:52] everybody like us. Some people just don’t like it, you know?

Facilitator: Yeah.

Respondent 1: I can’t be bothered. Like if I make a dessert and people say, “Did you make that?” And I think, “Yeah, and it really wasn’t hard, you know, it took me –” It’d take you 30 minutes to make something that people think is just –

Facilitator: Like it’s a science.

Respondent 1: Complicated, yeah.

Facilitator: A complicated science.

Respondent 1: Really complicated. So I mean, I would be happy if there was a call for people to go and help, you know. As a retired person I would love to do that.

Facilitator: That would be amazing, yeah.

Respondent 1: You get on that, you get a grant written and I’ll come out to B.C. [Laughs]

Facilitator: Yes, please, come join us.

Respondent 4: But for people sort of like Respondent 2 who says “I don’t want to cook and just give me the nutrition I need, I have other things to do”, that’s another way to get your food in and to eat well, is to not make it a sit-down. And people live alone are like, “You know, you should always be eating with somebody”, and I was like, “But I live alone, what do you want me to do? Call people on Zoom and have –?”

So to have other ways to eat healthy, like here’s some shakes you can do all week and you’ll have everything you need, and you’re not going to be cooking, and you’re getting – Like that would be – I’m sure Respondent 2 would love that.

Respondent 2: Exactly.

Respondent 4: And people like that who are not interested in cooking, are really interested in getting nutrition, then they just have to buy a blender and they know what to get. Other ways to sort of eat, there’s really not only one way to eat well.

Facilitator: For sure, yeah. Thank you so much.

Respondent 2: The [back of it? 00:52:31] actually had like [unintelligible 00:52:33] like a lifestyle. It looks like eat with people, and do this, and do this, and cook all the time. I disagree with all that; for me personally anyways.

Facilitator: For sure, yeah.

Respondent 4: It’d be nice to have little points in the app if people want to track that. But I’d also want to be like, don’t calculate that in my – like I can’t go out and see people every time I have a meal, like just –

Facilitator: [Laughs]

Respondent 2: Yeah.

Facilitator 2: But do you think if there would be some basic questions, for example when you want to sign up and do the application there would be some questions about your lifestyle and –? Yeah, so – And the app would give you advice they send, or those lifestyle choices that you have made, do you think it could be useful for other people?

Facilitator: Yeah, like make it customizable I guess for each person. Do you think that little questionnaire in the beginning when you first start using it will help you in the long run and make it easier for using the app?

Respondent 4: I do work with behavioural medicine people so I do have an understanding of behaviour change, and every app will fail if it’s not – if you don’t feel like it’s speaking to you directly. And so I think what everybody saw when they said that on the – when we saw the plate for the first time, are like: “Yeah, but.” And that was hard to engage with, so definitely if the app is not ready to understand – you know, I have mobility issues, don’t ask me to walk around, like some people can’t walk around, they’re in a wheelchair. So all these things have to be – I think for the app to really be successful the app needs to be able to adjust.

Facilitator: What [side? 00:54:29] do you follow? Or what type of lifestyle do you live? Or what do you have to consider when eating? So yeah, that’s perfect to know. Thank you. And yeah, just throughout this conversation we realized how complicated it would be to use this app, so I was wondering if there should be – like what type of instructions or support should be provided to help guide people to use the application, if we were to add all these different features that we were talking about?

Respondent 3: I think basically the app should be really easy to use, because otherwise people won’t use it. But for the instructions, I think that it can have different kinds of instructional videos like every other app. And I think videos maybe is the best way that people can find about how to use the app.

Facilitator: Thank you. And then I think we mentioned before, sometimes the general public won’t know how to classify different foods, so do you guys think there should be some sort of page to explain what type of foods lie under this food group? Do you guys think that type of resource would be helpful?

Respondent 4: Essential. I don’t think the app works without that. When people don’t have that information they can’t use the app, I don’t think.

Facilitator: Perfect.

Respondent 2: Yeah, so maybe if you could input what you ate and it would sort it out for you.

Facilitator: Mm-hmm. Sort it out for you. Yeah.

Respondent 1: This requires a certain degree of literacy too, because I mean it’s maybe not obvious to everybody but not everybody would know how to spell. You know? Like you put something in that you spelt wrong. I don’t know, it should have some sensitivity I guess, to that, or maybe you could say it.

Respondent 4: That’s why the photos is a no-brainer there.

Respondent 1: Yeah.

Facilitator: Yeah, photos. OK, perfect. And then for I guess features of the app, what type of features do you think could help with having people stick to using the application, like continue using the application?

Respondent 5: Maybe you could do a point system, like checking-in every day to get points. But then I haven’t thought about a reward for checking-in every day. Because, I don’t know, I had that idea from another workout thing, checking in every day for your goals, so it could be something like that. I’m not that great at coming up with ideas.

Facilitator: No, that’s perfect. Thank you.

Respondent 4: If we wanted to go high-high-tech you just take a photo of your food and app works most of it out for you. You know, in a not-so-distant future that’s actually probably possible. But all the apps, if it’s at all complicated or people feel dumb using something, they’re gone, they’re gone, they’re not going to use it.

Facilitator: Yeah, for sure. Simplicity is best.

Respondent 4: But also, I am using for a week but all I keep seeing is bad scores, people also drop out of that as well. So it’s that tricky balance between, you know, we want to encourage you as you go but keep you within the lines. Behaviour change is so difficult, and food is such a fundamental lifestyle element. This is why I wanted to contribute to the focus group, this is such a big mountain for most people, for everyone probably. So every little bit we can try and do is good.

But rewarding people not for the results but for the effort I think might be where we want to hit there.

Facilitator: Oh, that’s such a good point to add. Thank you. Because most people feel like they’re rewarded after they’ve accomplished something.

Respondent 4: Yeah, but if you don’t get that accomplished then do you keep trying? Generally human nature is like: Well I’m no good at this so forget it.

Facilitator: Yeah, that makes so much sense.

Respondent 4: So to try and stop that [automatic? 00:59:11] would be really hot.

Facilitator: I’m definitely going to like underline that because that is something that sticks out to me a lot. But yeah, both food and behaviour change is very complex. But yeah, does Respondent 2 or Anita have anything to add?

Respondent 2: I mean do we want this app to be just about food tracking, or is it about lifestyle, like health changes, health goals, anything like that?

Facilitator: It’s actually going to be focused on just food tracking.

Respondent 2: OK. So the app doesn’t care if you gain weight, lose weight, or anything like that?

Facilitator: I don’t think we would ask about your weight. Yeah.

Respondent 2: OK.

Respondent 3: I think maybe adding a chat room for people who are using the app, it can be a kind of good idea because people can share their progress for each other, and maybe it can motivate them to use the app more and more.

Facilitator: For sure.

Respondent 2: Yeah, some kind of sense of community, sharing recipes. Maybe, hey, this – if you’re looking to hit these certain portions of your plate I have found this is a great way to do that.

Facilitator: Yeah, totally. Thank you for that. And it brings us to the next question which asks about which features do you think in an app would help improve a user’s confidence when tracking their food.

Respondent 1: I guess to make it as easy as possible, and I guess taking a photo of your food is probably – and I don’t think it’s that far in the future, I think it’s pretty – It’s probably there, you know, just like a QR code. So yeah I think probably taking a photo, it’s kind of fun to do that. And, maybe by my suppertime saying – like a warning: You haven’t had any vegetables yet, and it’s six o’clock.

Respondent 4: Oh, brilliant. Brilliant. Yeah, that’s good.

Respondent 1: Yeah, or a water thing is really important too. And it’s unfortunate that the exercise isn’t mentioned, because somebody eating well, that doesn’t do any kind of exercise at all, and somebody who’s – and then as you said there is a whole handicap issue. Yeah, it’s quite a big job. Yeah, glad I don’t have to do it.

Respondent 4: If the app at the end of the day can give you a little report card, and maybe people can choose: I want to look how bad I was today, or flip, I want to look at what I got right today. So someone like me will want to look at what did I screw up today, because you know, that’s where I’m at, fine-tuning. But somebody who’s just starting out might just want to be like, hey, you got X number of vegetable points today. Like just a positive reinforcement. OK, that was good today. Those were good choices today. I’ll repeat those tomorrow. Et cetera.

Those might be good to keep people in sort of – You get points every day if you use it, period, whether or not you did well with your food. You just like going back to the app because it’s telling you, “You did good today, you did good today”, regardless of how good you did that

Facilitator: And I think just a question I have for Respondent 2, because I think you used MyFitnessPal, I was wondering if there were any parts of the app that made you want to keep going back to it or using it for your lifestyle?

Respondent 2: Just for me it tracks everything I need it to. I just need calories, and then my protein, carbs and fat breakdown. It’s super easy because I can just scan a barcode whenever I’m eating, too. Just ease-of-use really. I know Layne Norton also has one called a Carbon diet app which has coaching as well. It’s on flexible dieting. But that’s much more intense.

Facilitator: Yeah, for sure. Thank you. I think, if I remember correctly, Respondent 3 and Elise have not used any sort of diet-tracking apps. So I was wondering, if you guys were to start one, do you guys have anything that comes to mind that you think would help you stick to it?

Respondent 3: I think having reminders in apps is kind of really important for me, because I have so many apps and I think that there should be a reminder to remind me to, for example, use the app, or – I don’t know – track my food, or something like that.

Facilitator: For sure. Thank you.

Elise: Yeah, if you add a push notification feature, something like even just reminding you to drink water, that would be like a good idea.

Facilitator: Oh, yeah. Just for water, it doesn’t have to be necessarily like a certain food. Because water is essential, essential. So I think, yeah, using water would be very good. Yeah, thank you for that. And I think Respondent 1 and Respondent 4 have actually mentioned accessibility issues before, so we wanted to also get into that and ask: What do you guys think the app will need so that it’s accessible to all people, people who have disabilities, like mobile disabilities, or live with blindness or deafness? How do you guys think that would be helpful? Or what are some ways to make the app accessible to people who live with disabilities and accessibility issues?

Respondent 1: You should probably do a focus group with those people.

Facilitator: Yeah, for sure.

Respondent 1: Yeah.

Respondent 4: And there are app developers that specialize in making an app accessible for colour blind, for blindness, for deaf – I mean there are app experts that will take your app and then go and say, “OK, this is what you’re going to need to do to make it accessible for X audience.” So you don’t need to invent the wheel there, there’s already experts out there specifically for that. But anyone with learning, reading, or any kind of physical disabilities, yeah, a special focus group specifically with them is I think your only way into that, into those worlds, to figure out how to serve them.

Facilitator: Yeah, for sure. Yeah, so it’s a very complicated –

Facilitator 2: So how about parents? I know that Respondent 4 was mentioning parents.

Facilitator: Oh, yeah.

Facilitator 2: Or even older adults. How do you think this app could be made more accessible for them?

Respondent 4: I’m not a parent, I can’t tell you, but I do know that they’re going to have strong opinions about it and you should speak with them directly.

Facilitator: Yeah, unfortunately I’m not a parent as well, so I can’t answer it as well.

Respondent 4: But also, I mean if you wanted to do another run at this kind of thing with people who live with diabetes Type 1 and Type 2, or totally different people who live with cardiovascular disease, people who are still smoking. You know, you could just redo focus groups like this and pull different stuff. But also maybe probably do some with health practitioners who serve those populations, who already know what they need and what they should be getting through an app.

Facilitator: Definitely. We have a separate focus group specifically for registered dieticians. Yeah, since we probably don’t have any other ideas about accessibility I’ll just go into the last question. We were wondering if there are any other features about the diet self-monitoring tool that could be helpful in [marrying? 01:08:06] the new Canadian Food Guide that we haven’t addressed yet. Do you guys have any ideas from the top of your head that we haven’t spoken about?

Respondent 4: Not me.

Respondent 3: I think maybe the app should have a section for different kinds of malnutrition, or even diseases associated with nutrition and foods, for example, anorexia or bulimia. And I mean, that’s maybe a source of – these diseases can be being so concerned about what you are eating, and about calories, or about everything else. So maybe there should be a kind of information about these kinds of diseases, or about different kinds of things like this. So some people who are just so concerned with their diet or with what they eat, maybe it can kind of be an alarm for them and so they can get more about all these diseases.

Facilitator: Perfect, thank you so much.

Facilitator 2: Sorry, Respondent 3, I was just wondering, do you think it should be just some notifications and reminders or instructions for them, or it could be just different categories in the app for those people who are dealing with anorexia? I know it is kind of difficult for them to track their diet, but yeah, what do you think?

Respondent 3: Actually, because I had anorexia I think that maybe at some point you are not aware of your disease; I mean you are not aware that you have anorexia. And so it shouldn’t be a different category because, well you won’t go in there, I mean you are not aware of that. And so maybe I think it’s better to have some reminders about it, and just notifying people that just be careful. Be aware that there are some diseases like this, and just check your diet, check your symptoms, and just be aware of all of these things.

Facilitator: Oh yeah, for Respondent 4?

Respondent 4: Yeah, I’m going to need to go soon so I just wanted to give you my last idea. Just between four or five of us here today, we like sort of went [really how? 01:11:09] far in scope and how complex some of these issues are. So maybe my best piece of advice would be to choose really specifically what the population you’re trying to reach, rather than all Canadians, I think that you’re doomed to fail with that kind of an approach because everybody’s got – There are so many different slices of the population that have different requirements, needs, health problems or goals.

So to be really, really specific about who you’re targeting so that people don’t take the app up and get disappointed and say this is not – Like be really upfront about: this is the profile of the person that will get the most out of this app and serve them well, rather than trying to serve everyone not so well, is I think what I would say to you guys.

Facilitator: All right. Thank you so much.

[End of recorded material 01:12:18]
