# Supplementary material for: Perceptions of self-monitoring dietary intake according to a plate-based approach: A qualitative study
Source: PLoS One. 2023 Nov 28;18(11):e0294652. doi: 10.1371/journal.pone.0294652 (PMC10683993; doi:10.1371/journal.pone.0294652)
Supplement: S4 Appendix — (ZIP) [file pone.0294652.s004.zip › Anonymized GP Transcripts/iCANPlate-GP-focus-Group-9.docx]

**iCANPlate-GP-focus-Group-9**

[Start of recorded material]

Moderator: So this is the, iCANPlate focus group, the 9^th^ focus group. With their numbers are general public on July 15^th^ at 5pm eastern time. So the first question that I'm going to ask is that what you think would make it easy or hard for you to eat in accordance the food guide, to the guide that I just showed to you? Would you like to follow it, or what makes it hard, what make it easy for you to follow any thoughts or ideas?

Participant 1: This is more –

Moderator: Sorry? Yeah the, here you can see the guide, yeah the guide is the plate. Half of the plate is filled with fruits and veggies and a quarter is filled with the whole grains and another quarter with protein products. Do you eat or do you like to eat in accordance to the guide or yeah? Yes Participant 2.

Participant 2: For me a couple of things, one I would say bad eating habits from when I was younger. My stomach is obviously stretched out, hear me, so it takes more to fill me that’s from bad eating habits when I was younger, eating buffets, eating contests and things like that. So that’s a little hard, the other thing is I am diabetic so when it comes to diabetes I mean I can look at the grains and I can say yeah and that turns into sugar, I mean the carbohydrates. Vegetables are good, fruits yeah I mean a lot of sugar, natural sugars there. I have a bad problem with my diabetes, proteins are good but I mean I've been watching a lot of documentaries, I'm a person I can’t stand soap operas or sit coms or stuff like that I love documentaries.

And lately I've been watching ones on nutrition and food and what happens and really what the cause of diabetes is and stuff like that. And a lot of that has to do with red meat which I am a huge fan of, so I mean it's just saying I have to, because of the diabetes I have to even be selective of what they say is healthy to eat. And when you get down to knowing certain food you even go to the diabetes website, look at the meals and you’re like, “no I shouldn’t eat that come on why are you telling me that that makes diabetes worse.” And it's just ridiculous. But a lot of that too is as your one, your previous slide there showed there was a thing of watch food marketing.

Because it may not be the healthiest thing for you but they will market it as the healthiest thing but when you look at the studies done, well yeah the study for milk was done by the dairy not by an independent. So I mean it's kind of one sided and stuff, I mean you can twist statistics any way you want, its all how you present them. And you can present them to look good even though they are actually bad. But yeah that’s my thing, like is say I have a stretched stomach and the diabetes and knowing that a lot of the stuff that I should eat will affect the diabetes.

Moderator: Great, any other ideas? Just feel free to jump in. So the potato even right Participant 2? The potato part in the half, half of the plate yeah they say it is no good for diabetes.

Participant 2: Yeah and I love potatoes, I love mashed potatoes, garlic mashed potatoes I mean I could eat them all day. But once that gets into you that is a slow sugar, it slowly converts to sugar so it raises your sugar levels over a long period of time. As opposed to something that is a fast sugar, I mean like you get into you that reacts right quick and peaks your thing so I take my insulin to pull it down but I mean insulin’s only good for so many hours and so if you’re putting things like potatoes in or spaghetti you don’t –

Moderator: But there is, yeah there is no fast sugar here right.

Participant 2: Yeah.

Moderator: There is no like – so does it work for you, the guide?

Participant 2: It does but it's also the same because of how I live on a very low fixed income for me and my son. It's Ontario disability because I fractured my back on a job, I was out working, I enjoyed working and stuff but then I fractured my back. So now I'm stuck on disability and they just don’t give you enough to eat healthy. They give you enough to cover junk food, yeah I mean the stuff that’s bad for you, the cheap stuff. So you’re kind of stuck, the one part of the government says here eat healthy the other part says yeah but we’re only going to give you enough to buy crafting or processed foods and everything, not enough to cover good veggies and fruits and stuff.

Moderator: Right, right exactly budgeting. Yeah it is real important when it comes to follow the guide, yeah Participant 1 do you want to add anything, I saw you moving your -

Participant 1: Honestly I, stick the question that it's made for me. I mean this sorting that works for me, for everyone to choose a non-piece or -?

Moderator: No just like if you want to eat throughout a day, for example you’re having lunch, would you like that your plate be like this one, like half of your plate be filled with fruits and vegetables and the other proportion? Do you think it works for you?

Participant 1: I like the mixed bowl, I don’t like to sort like this I think.

Moderator: Yeah I think -

Participant 1: Mostly because of flavours, you mix some ingredients together, you create new flavours that you cannot experience them by sorting out or trying separately. If that’s the question, no I don’t like this division.

Moderator: Yeah I think as you mentioned that you love Iranian foods, I think the food, the most of the, I know that most of the Iranian’s plate should be filled, usually should be filled with grain like rice or bread and this app does not mirror that right?

Participant 1: Yeah.

Participant 3: Can I add something?

Moderator: Yeah sure.

Participant 3: Well actually my main problem with this, actually proportion and ratio of this food is with my daughter because she loves specific kinds of foods, she just love rice, pasta, potatoes and something like this and I can hardly add something which has vegetable in her food. She actually separated those vegetables, put them aside and eat the rice off the plate. The first problem for me is with the children, with my daughter actually and the second is about the recipe. I'm not sure if there is a recipe, recommend that I know it may be very different among different people with different taste but it shows in a book of recipe’s which actually contains the same ratio of this ingredients in each plate, to have a healthy plate or not?

Moderator: I don’t think they, one fits your recipes right now but yeah we are going to make this application, maybe they could add it, yeah.

Participant 3: Yeah it's a good idea.

Moderator: Yeah we’ll get to that as well. So any other thoughts?

Participant 4: I’ll say it right off, I'm a bit biased because I look at it every day, it's on my fridge. And I used to look at the old one, which was the one that had the portions and sort of the pyramid on it. This one is much better than the old one because if I had eaten the number of portions per day that the old one told you to eat I would be very, very fat right now. I just found it impossible to eat that much, I was, it was just crazy. So there’s no notion of portions or quantity in there.

Moderator: So this is much -

Participant 4: Yeah this one is better from that perspective, the controversial part of course is this one is that there’s ton of veg which is half of it. I personally like veg but it's not, it doesn’t suit everyone’s preferences or cultural background. I was actually born in Morocco so yeah I would see mostly grains, couscous and what not on there as my main diet and I guess fine bread. That wouldn’t do it for me, but I do tend to eat what is there and I'm also biased because we have a diabetic son so we have to watch what he eats so that joins back with Participant 2’s remarks.

Moderator: Great, yes Participant 2.

Participant 2: Just an add-on following up with, I think its Participant 3 said. My son I don’t know if you can see him walking around in the blurry background, he’s autistic and autistic children have, they’re very sensitive to certain things. And one of he’s is his sensory of the textures of food. So for me to try and follow the guide, I’d love to but with him it's basically it's, cheese, hotdogs, craft dinner and chicken rom and noodles, that’s about the only thing he’ll eat. There’s maybe four or five other things, I mean certain oatmeal cookies, there’s only one brand he’ll touch. Cookies that I make but of course they’re sugar cookies so like that’s just great for he’s dental but that’s what he eats.

Other than that he loves his Kinder eggs but he only wants the surprise, we throw out the chocolate he won’t touch chocolate. Freezie’s he says yeah I’ll take one, he just kind of rubs it a little bit on his lips and then it's like no, don’t want no more and that’s it. He will not eat strawberries, blueberries, carrots. He eats corn, but of course we all know corn has no nutritional value other than the little piece of protein in the middle and that, but he’ll devour corn like it's going out of style. He used to eat my chicken wings, he won’t eat chicken anymore. Some chicken nuggets, some chicken fingers but I mean we’re talking deep frying or breaded stuff that I mean just adds calories like crazy.

But that is a big thing that you get to follow with your children and as Participant 4 said with a diabetic son, I have the diabetes but also I have an autistic son so I've got two things just interfering with trying to follow the guide and eat healthy.

Moderator: Great. Any other ideas to add? So when it comes to the other food that are not in this guide, like the cookies or the fries that Patty says that are, would love to have. What other foods can you think of that are not included in these guides? Can you see it again, see the guide please Participant 3? Yeah we already that there is no ice cream there right. No cakes, no ice cream, no even the fat sources like the oil that it add to the fry. Yeah we can see the examples over here. So how do you suggest tracking these foods in the application, in you know?

Participant 2: Well if I may, if you look at it you can say well chicken’s not there, or peanut butter’s not there but we see the egg which eventually should turn into a chicken, yeah I mean if you leave it alone. And peanuts and stuff, OK so you’ve got raw peanuts but you don’t process and turn them into peanut butter. Jams for sandwiches and so well that comes from strawberries and the fruit that you see there, but you’re now getting into processing food and stuff and adding other ingredients and chemical preservatives and stuff. I mean, so I think the stuff is there but a lot of dairy is missing from there.

But yeah, that’s covered with that little thing of yoghurt but I mean you’ve got dairy, you’ve got cheeses you have all that other stuff made from dairy that isn’t shown there, but it kind of is but it's processed you know what I mean. So I mean I think the foods are there it's just you have to go away from the natural unprocessed food to into the cheap processed foods now. Which doesn’t make sense because they’re adding a whole bunch of stuff to it which should raise the price and the cost but it doesn’t, it lowers it.

Moderator: So let’s see the Apps first marker first and then I will probe this question a bit more. Can we see the Apps – first of all let me ask another question. So keep these foods in your mind for our next question that I will ask you after showing the first mock-up of the application. So if you want to make some changes into your diet what techniques would you use or what would help you to keep tracking, to be stick to those changes. Would you like to set goals or journalizing, journaling or maybe some support group competitions? What would work for you when you want to make changes in your diets? Yes Participant 2.

Participant 2: I know I talk a lot, I'm sorry.

Moderator: No worries that’s totally fine, that’s what we want.

Participant 2: What I do, I don’t know if you can see it, I’ll get it – I have a Fitbit, not to promote it then over an Apple watch or anything. Yes my band just broke so I've got to get a new band but with Fitbit they have the same here where they can track the calories burned from the exercise you do. You can go in, you can log your water, you can log your food all that kind of stuff. I also have an app I use for my diabetes that I, it ties to my blood meter, it's not the app that came with the thing it's an outside app in which just blows away every other diabetic app I've seen. But it ties my meter, I get the reading, it calculates how much insulin I need based on what my sugars are at and what my insulin sensitivity is, it does all the calculation.

I can also with that track my food in the way of it's got a barcode section, so I just pick up a can of soup scan it and it's already got a list from, there’s like four or five data bases it uses, it will get all the nutritional values and it will say, well if you’re going to have a serving of that soup you need to add two more units of insulin or three more units of insulin. Sometimes it will say I have to bowlish it 20 minutes before eating or immediately and then wait or something in case my sugar’s already up high and it says, OK let’s bring them down first before you start eating. So I use Apps, I like the Fitbit because it can track everything. My big issue is that double entry, triple entry I mean by the time I've entered it all into my Fitbit app and then I enter it all into my diabetes App.

And then I enter it into this will work here and stuff I'm not hungry anymore, I want to go to bed because I'm tired of double entering everything. I mean or it's like, well let’s see it says wait 20 minutes. Well I guess I've got time to enter it in to all the other Apps before I eat, or it's like I mean I've got to prepare the food and it says wait 20 minutes. Well great my foods now going to get cold before I can eat it. It's, there’s some Apps out there but it just, they all don’t work together I mean, it's not like Microsoft office suite where you can transfer data between all the Apps and stuff like that. That kind of I guess connectivity between things is, would be great but we need, like I say double enter like too much time.

Moderator: Exactly yeah. Any other ideas how, what techniques would you use when it comes to making changes to your diet?

Participant 4: The one thing could, the main thing is awareness. So most people don’t realize what they actually eat and I've participated in a couple of studies, the main beef I have with all the applications is you enter stuff to Participant 2’s point until you’re blue in the face but they don’t give you any results. Right it doesn’t tell you after a week or after two weeks or after a month, hey you did well or you did poorly or advice. Obviously you need a human to help you out with that. But I'm sure that applications can be managed a lot better to provide you with that insight because at the end of the day you’re just a prisoner of what you entered and you spend way too much time for I would say very disappointing results.

Moderator: Yeah be mindful, yeah that’s a really great technique. Be mindful of your eating. So what else, what other techniques?

Participant 3: Well I do agree, first counting every calorie that you take every day is really not such a good thing for everyone and you may miss that and you may not actually like to do that. Well, I think that must be something that do not need any entry data more as I think Participant 2 said, it should be, has a connection between the devices that you already have between maybe or a smart watch or a computer or the other applications that we have. They might be connected in a way that they alarm you if you, if your dish was health today or not and but you should be, considerate for the other days.

Moderator: So how do you monitor our diet, do you use the, be mindful of your eating?

Participant 3: I try to be but I should say that I am, it doesn’t actually happen every day. Sometimes we are out of home sometimes I cannot have enough time to put my sums with it and it's something I have to get right is something from what I already have in that situation. Which might not be very health I know.

Moderator: Yeah. And others feel free to share your ideas in the chat box or if you could just jump in and tell us now about your, you let us know about your ideas. Any other thought, what techniques would you use when it comes to making changes to your diets? Yes Participant 1.

Participant 1: I can respond but I don’t know my response is completely related to your message or not.

Moderator: Yeah not it -

Participant 1: Other than I want to change in my eating habits, I for example I like to use lots of fruits and lots of daily district, I fill my refrigerator with those things. And I have to eat them because I paid them, I don’t want to throw them away. As they come -

Moderator: Yeah kind of like fining yeah.

Participant 1: Yeah but if I want to use an App, I prefer, because I value taste of food a lot, it's the most important thing for me that what I eat tastes good. And for example most people don’t like to eat raw vegetables. If I want to use an app I prefer to use suggestions of recipes that make for example those vegetables taste good, taste better. So I can use them in another way. And Moderator maybe you can help with this, I don’t know the English word, the English equal for Carras, it's a vegetable – I think you don’t know it too.

Moderator: That’s a good question, I don’t know it either.

Participant 1: It's a vegetable that makes you lose lots of coloury, it's very helpful in losing weight. But I hate to eat that vegetable raw.

Moderator: Yeah celery is the -

Participant 1: Or the recipes to find, I don’t know to kind of bake it or mix it with other vegetables, other ingredients and that makes it taste better, yeah.

Participant 2: You have my interest there on that one, I’d love to know what it is I will find a way to eat it.

Participant 1: Yeah I think that’s the problem for so many people, we know that’s healthy, we know that our body needs it but we hate it so when you’re looking for a way to, I love it, I don’t know it changes -

Participant 2: It's like Brussel sprouts, nobody likes them but they’re good for you.

Moderator: Yeah exactly. So what, and I know that some of you have already used some of the applications that are, that help people to track their diets, like Fitbit or the ones that Participant 2 was using for diabetes or those studies, that Participant 4 had used when participating in those studies. What other applications can you think of, do you know that you have used to track your diet? Have you ever used one of those, probably self I don’t know if I had? OK so what has made it easy or hard for you when you were tracking your diet in those applications? I know that you have already brought up some ideas but let’s explain about it a little more.

Participant 4: So I’ll start with, I’ll talk about Quinoa because that the one I used. And I should also mention that in my past I was also responsible for UX design in a software company so I know a little bit about designing interfaces and that interface drove me crazy. And it drove me crazy because it's not very, it doesn’t follow the normal habits of what happens when you sit down and eat just like Participant 2 was mentioning before. If you have to sit down and think about it and take 20 minutes to enter something you’re not going to do it end of story? With Quinoa it was extremely frustrating because you would take a picture, that’s how you start right. It would then try to guess what was on your plate, did a miserable job of it so you ended up having to enter everything manually and then it would require you to do that in fairly precise quantities.

So I happen to be a nerd so I have a scale next to me in my kitchen so I could give any, weigh any grams that was being inputted because my son uses it every day. No normal human being is going to measure how many grams of celery or how many grams of meat is going on their plate that simply doesn’t work. The other frustrating thing about Quinoa is it's great for prepared and processed foods. So you can scan the label of the food and it will say, hey you’re eating whatever and put all the values in. My problem is 99% of what I eat I cook myself or my wife cooks it or my son kicks it or my daughter cooks it. So as soon as you have a homemade plate, now you have to deconstruct the whole thing.

So I'm French I like [foreign language 00:26:58], there’s 30 ingredients in there. So you have to type in 30 ingredients, try to figure out what the proportions are. I was born in Morocco as I mentioned before, I love couscous, there’s 50 ingredients in couscous. How the heck do you enter that without spending half an hour getting the information in there and you guys from Iran, I mean there’s tons of complex foods out there that I love, but again it would be very, very difficult even to find the ingredients in the data base that some of them require. Right I couldn’t even enter all the ones that were in couscous from Morocco and they’re not that exotic.

So I could imagine what it would be if you’re Chinese or Persian or something else and you won’t find them because you have to be precise because otherwise to your point you’re not going to find them like cake.

Moderator: Right, right exactly. So any other ideas, Participant 2 do you want to explain a little bit more about the Fitbit or the other app that you were using for your diabetes?

Participant 2: Well they’re fairly intuitive to EED, the nice thing with the Fitbit one is you can do it on their website and enter it in it will immediately strike sync down to your watch and stuff through the internet which is great and vice versa, that you can actually on the watch actually go in there and say OK well I just drank an eight ounce bottle of water, oh I need to add this many calories. You can’t do it as good as you can on the website because on the website you can actually get more information in but the watch you can do basic information which like I say synchronizes and stuff. That’s great, the other app I use, like I said, the one from the diabetes it's, you’ve got the USDA data base in there and there’s about four to five hundred data bases that you can search.

And if you do find something you stand a UPC I mean there it is somewhere there and it's not in the system you can actually then go and look at the I mean at least here in Canada we’ve got the nutritional thing there, so I can go in there add a new item put in a manufacturer da, da, da, da, the barcode and enter all that information and then it gets added into that global data base. So it's like everybody builds the extra, the other data base. Not the USDA one and stuff like that but they have one that they have their own where all these other ones get added in. So it's like everybody helps build it and like I say, the barcode thing is great because you just scan and there it is, I've got all the information.

I can do my whole meal, my breakfast, I can scan two eggs, this, this, this, this or even just do a quick search. Boiled eggs, there entered two of them and it just adds it all up, save them as a meal so that I can just go breakfast, and it puts all that there for me. It's a great design, it's done by a company in – God, let me think now, somewhere around what was Czechoslovakia, done there by a medical team and stuff. Great features and it's like four bucks a month for the extra ability for more profiles and stuff. But the add on if I can plug it, is you can also track your pet’s diabetes, you can set up a thing on their profile and go it's for a pet. And if you got a pet that is diabetic, you can do it as well in there and stuff.

Moderator: Great. Is sounds like great features. Interesting. So any other ideas? Any other thoughts to add to what would make it easy or hard for you to follow those applications?

Participant 1: If I want to give a suggestion about these applications related to food, as I said before, I don't like that dividing food by either notice, this is dairy, this is fruit, this is I don't know, vegetables, you need to eat this. I prefer to go with calories. Maybe there are some favourite foods, regular food that people eat everyday. For example, you mentioned pasta. I mostly like for example, in pasta, I see the ingredients there. The ingredients of pasta and how much calorie the past has. And I can choose among the food. For example, I want to know a regular human body, how much calorie needs during the day. And I can choose among those foods, because I can see how much calorie they have.

And I don't, know for example, between pasta, kebab, and I don't know, chicken beans, I can choose which one better suits me if I want to lose weight. The key point is that I prefer to go with the calories rather than dividing food.

Moderator: OK, great. So do anyone know some any self monitoring tool or application that currently resembles Canada's Food Guide? Like where's the Canada's Food Guide? Anyone knows any application? No, I guess not. So when you think of eating throughout the day, how do you view the app working? Considering having your breakfast, lunch, snacks, dinner? How do you see it working?

Participant 4: Again, I’ll start, if I look at quinoa. Quinoa you started by taking a picture. Half the time I would be hungry and I would eat most of my plate before I actually got to the picture. And then you go oops. Because obviously you have no picture to show. And then it becomes much more difficult to enter the information in there. So this approach is actually very close to the paper based one I did, where you simply put the proportions in. So I can see that you can do this in about 20 to 30 seconds.

So that would be fun. I don't snack, but I would imagine that it would be even faster for a snack. So that's very positive, because it has one merit. It's very, very simple. You're not asking for details about food, you're not asking for weight, you're not asking for very specific things. You're just asking for proportions.

Moderator: OK. Any other ideas?

Participant 3: Moderator, I have a question. And I don't know if it's possible or not, or if it's something benefit, it has some benefit or not. Is it possible that we scan and the plate and it distinguishes the ingredients and the ratio, based on the Food Guide, Canadian Food Guide or not?

Moderator: So like the one that Participant 4 was telling us about the quinoa that you just try the food and it would automatically fill in these guides, right.

Participant 3: I haven't seen that application, but I don't know if it has already do the same. You scan your plate and distinguish the ingredients and the portion?

Moderator: Yeah the other way to do that, but it doesn't fit in plates. It doesn't just fit into the guide.

Participant 4: To your point, Participant 3, it works very badly. Because it’s taking a picture and it's trying to guess what's on the plate. So every time I would take a picture, let's say have a curry. It would get something horrible. Because it has no clue, because it's looking - for it uses artificial intelligence. It's trying to guess, but it's not good enough. It's more frustrating actually, than helpful.

Moderator: If they are using some machine learning algorithms, I think they would be better as we progress during this –

Participant 4: That takes big money.

Moderator: Yeah, exactly. And developers might have no money.

Participant 4: I don’t mind if you give me the contract, but no, it takes big money.

Participant 3: The machine learning would work for this?

Participant 4: Yes, it would work very much work, because it would recognise things. I do - I'm specialised in the aviation field, use a lot of AI in there. It is phenomenal what it can do. But we are talking probably, two, three, four, $5 million worth of budget before you get into recognition of specific foods, right. So I'm sure somebody will figure it out.

Moderator: Yeah, hopefully future grants would support us, I hope.

Participant 4: Good idea, too soon.

Moderator: Yeah, hopefully, fingers crossed. So yeah. Any other ideas? But how do you see this application working?

Participant 4: Can you show us the next steps, for instance, for going to water, and so on, and so forth?

Moderator: Yeah, it hasn't been developed yet. The only thing that we have so far is this simple plate. So we're going to add some more features to it.

Participant 3: Is the plate the same for all people, for seniors, for children and adults? Is it the same?

Moderator: It’s supposed to be for general public, like adults, people who are older than 18 years old.

Participant 3: OK. Because seniors might have different diets.

Moderator: It should be working for seniors as well, but the idea could be working or for an adult, not for children or teenagers. Any other ideas? So, if you wanted to represent different meal portions on the plate, how would you suggest those? What references would you suggest when you want to feel the plate proportions?

Participant 2: With it right now, like when you do the dividing lines, once that's done, can you then grab the line and slowly bring it up to reduce and expand the next one and stuff? Because it's kind of an - if not then or an add on would be, just above each one of those, the buttons down below is, put a little pull down or some as a percentage. You know what I mean? Just above the green, you could just go in there and go 50. Boom 50%. I mean, and be able to calculate what's remaining. It can be shared between the others and stuff like that. Yeah, rather than trying to grab a line and say, OK, I want exactly 51% but I can't get it. Easier to just type it in a box. I mean, 51 bang and there it is, and it draws it.

I'm one of those that goes back to the DOS days of computers when it was all tech space. No fancy graphics, pictures are nice. Yeah, I mean, I like my little icons on my screen, but I could not count how many times, I still wish I could just type it in like Linux and just type the commands or something like that or just text based and get it done, forget to pretty pictures, uses resources in an application. I like to get away from that. I know it makes it look good. But I mean, fresh coat of paint on a car doesn't make it dry faster. It's what's in the engine inside.

Participant 4: Also very good points and good UX design. UX stands for user experience, basically says that you should have two, three or even four if possible, different ways of entering the same data. Some people are graphic, some people are numbers driven. Some people are proportions driven. So if you give them minimally for good UX design, three different ways of inputting the same information in, that's a good design. Any system that only has one, you're going to appeal to the guy who likes numbers, like Participant 2, but not to the person who likes drawings, right.

And if you're trying to use it on a mobile device, which is clearly the intent here, then the user interface is quite different from what you would see on a big screen, on a computer, on a PC. So the advice there is to have at least three ways of entering the information. That way you're sure to capture everyone's preferences. Because everyone has a different style.

Participant 2: Yeah, that's what I do. I'm a software developer, as well Participant 4, and doing interfaces and stuff. And like you said, I have a preference towards the number side. So the apps I develop, I kind of stray towards that. But as you say, there are others, they like the pretty pictures and little graphics. OK, go for it, but multiple choice for ways to do it is absolutely the best way to go.

Moderator: Yeah. Great idea. So when you want to track your food based on portion, which references would you suggest? Would you suggest hands and palms or face, or like cups, spoon, table? What references would you think could be suggested for the general public?

Participant 4: Allow them to choose. That's always the best, right? You personalise the application, you own it.

Participant 2: Yeah. I mean when you're entering and people may want to do proportion, but they also may want to say, well, I want to enter in based on calories. I want so many calories coming from this, so many calories coming from this. I know I've got 2000 for a healthy day, so divide that by three and I mean, give them that option. And like the other app I use, when you put in the proportions and stuff, it gives you a pulldown, one cup, teaspoon. One serving, which it knows is from the data that's been entered about the nutritional value, you also put in the serving size. So you can go serving size, have things like one slice, two slice when you bring up bread, it does that.

I mean a large, a small, a medium, all different options. Yeah, I mean, for those that want to get precise, and they can do it. And they got their choice how they want to enter it. I mean, I want two cups of fruit. Well, how many calories is that? How big of a portion is that? They don't know. So I just want two cups.

Moderator: Interesting. Yeah, I like the idea of giving people options.

Participant 2: We don't get to choose much nowadays, it's chosen for us. So the more you can make people happy and let them choose something, you're looking at a happy user.

Moderator: Yeah, the those options could be customised. For example, when they want to sign up on the application. They could customise the app based on their preferences.

Participant 2: Yeah, weather apps. My weather app, I'm glad I can customise it because I'm old school. I like Fahrenheit and I like feet and miles, you know what I mean? But somebody may want Celsius and kilometres and stuff. I mean, it's –

Moderator: Great idea. So any other thoughts or -?

Participant 1: I have one question about - does this app considers the people's own situation? For example, how much active they are during the week or it’s the same for everyone?

Moderator: Yeah. We definitely should have to think about it.

Participant 1: Yeah. Because I'm thinking for at least three levels, one for athletic person who does lots of exercise size, one for regular person. And the other one for lazy people like me or don’t do lots of exercises. And yeah, I think having those three levels at least would be better for plate suggestions.

Participant 2: To add onto that, you can have the same where it's somebody who is more active, may want their total calories per day, and their proportions done a certain way to match a highly active person. Yet someone who's not so active like me that gets exercise from picking up chicken wings, changing around so you want less carbohydrates, more proteins. I mean, based on that, as [Participant 1] was saying.

Participant 3: Yeah. Definitely, I as a user want to liking the application for the first time, I should enter some of my data, such as my age, my weight, my height, and maybe IBM, something like that. And then it will be customised for the user, yes, for the user who is going to login.

Moderator: Yeah, that could be a good idea too. Yeah, just this basic mock-up is all that we have for now. But we definitely have to put those –

Participant 3: Even the data there. Yeah.

Participant 4: I think part of the discussion you're having right now is we've discussed the inputting. But the other side of it is, what you get out of it. Because people are about effort and reward. What is the reward of the user that they get out of it? If I listened to Participant 1 correctly, you'd want to get some kind of calorie count and some kind of result out of that, let you know, well9, I'm a couch potato, so I'm doing okay. Or oh God, I'm eating way too much and I better be careful, and maybe change my behaviour. Right? Sorry, no offence to you Participant 1.

Participant 1: It's OK.

Participant 4: But you know what I mean? For every effort I make in life, what do I get out of it? And I don't want to sound egotistical, but most people behave that way. And they stop doing things if there is no reward at the end. So what have you guys looked into as to what you could get out of the other end? Other than a great dietary study, which, of course, is part of your studies?

Participant 2: Well, you Christoph, you look like you're around the same generation age bracket as me back then. I mean, people didn't worry too much about rewards and stuff, they did things just to do them.

Participant 4: That hasn't changed, Participant 2. People have already based on that.

Participant 2: Well, the newer generation, it's more what's in it for me?

Participant 4: Oh no, I don't pretend that we were any more generous than the new guys. So what's in it for me, Moderator?

Moderator: Yeah, it's a surprise as well, I know. So yeah. So let's get back to the question about the other meals that we were talking about earlier. Oh, I see that [Participant 5 00:48:50] could join us. Yay, it's great. So yeah, let's talk about the other meals, like the cakes or the fries or the - those other foods, that ice cream that was not on the guide. How do you suggest them to be tracking this application in this interface? We can see the question in the chat box, yeah.

Participant 4: Well, by separating them, you automatically give them a value. Because if you're entering information into any system, if it is part of the standard information that you provide, you're not giving them any value, negative or positive. You're just saying, OK, well, this is the information that I need, it's just data. The fact that you separate them out means that they're either good or bad, because you have to make an extra effort to go and get them. The intent when they did the new Canada Food Guide was very much to exclude them, that was done on purpose. And if you look at the food guides from the rest of the world, I have the French one from France originally. They're still on portions and stuff like that.

So they don't separate that out, and they don't discourage people from eating anything. He would just basically say, don't eat too much of something, but still eat it. Here the message is kind of don't have cake, don't have ice cream. It's subliminally perceived as being negative. Don't have a kebab, right. Because I don't see any kebab on there. Don't have [ceftin], and I love [ceftin]. So it's like you're sending a subliminal message by trying to do it separately. And that's the difficulty, because I know it's not integrated into the food guide, and it makes the entry more difficult. OK, so it's not an answer. It's just -

Moderator: Yeah, an idea. What everybody else think about it? Yes, [Participant 5]. Oh, we can't hear you I'm sorry, [Participant 5] you're on mute.

Participant 5: Yeah, OK. Can you hear me?

Moderator: Yeah.

Participant 5: I think dropping the dessert is not a good idea. Because we cannot eat any dessert in long term. It can work for a short time, but it's not a good idea for a length. I think maybe it's better to include them in the plate. But we have some limit for it, for example, a quantity per week, for every week or in mounts. But dropping them, I think it's not a good idea.

Moderator: I agree, I need the cookies too. What do other people think? Should they be here? Let me share the examples. You could see them. What do you think about them?

Participant 3: I still haven't get my answer of my question, why cheese and dairies are not included in the plate?

Moderator: That is some recommendations when you go to the website of the Food Guide, Canada's Food Guide. We could see some recommendations, but I'm not sure why I think it - I don't have the - I can answer your question here. So there are some recommendations to have dairies there for that, but there is not any specific portions or any specific place for dairies in the guide. I'm not sure, I'm not –

Participant 3: You mean that there isn't any specific reason for that?

Moderator: You could see just a bowl of yoghurt in the guide, but there is not any cheese or milk.

Participant 3: Yeah, for breakfast. Yes. And in some countries like Switzerland, it's a main part of their dish.

Moderator: Yeah exactly. So how do you suggest them to be tracking this application if we wanted to include them? Because they're important. So how do you suggest them to be tracked? Do you think they should be - we should put them in the protein product, when people are tracking their food? Or they should be a separate, maybe even category?

Participant 2: Yeah, why not a separate category of other? I mean, you've got those, you've got the plate from the Canada Food Guide died divided a certain way. Put those buttons here, but then in other, and under that, yeah, I mean, you could sub that out into dairy products, baked goods, fried goods, stuff like that. I mean, I don't know. It's more that thing of more options. But you got to be careful, because if you get too many, then people can get flooded with too much. It's like going through a voicemail or a digital thing, an IVR. Like press one for this, press two for this. By the time you're done, you've entered in an international phone number, and you've got nowhere to speak to somebody, don’t have any options.

Participant 3: Actually, I think some scientists believes that there is not necessary for our body. Maybe the reason of dropping them in the plate is that they think that it's not necessary for our body. But I think we can add, there is which a very low portion of fat into proteins like Greek yoghurt and 0% fat of milk, we can put all of them in the protein part. But about the fatty, there is butter or cream cheese or cream, we can have a separate part. But I think it's better to have some - how to say? Maybe the app should have the capability to separate the quantity of each meal. For example, when we eat cream cheese, it should have separate the protein part into the protein section, and put the fat part in the separate section. As you said, in other sections, for example, yeah.

Moderator: So you mean that you just enter the name of the food that you're having, and automatically would be will be -?

Participant 3: Of course. It will be separated into the sections.

Moderator: Yeah, like scanning barcodes or -?

Participant 3: It’s not necessary to have the dairy part, I think it's not necessary.

Participant 2: Like the app I use for my diabetes, when I go there to enter food, it's just a one spot, I just start typing C-R-E-A-M and it will start pulling in like with, I'm guessing something similar to Ajax programming, it'll just update as I type along. And it will then give me a choice when I get enough of like cream cheese. Then I can go down and it'll say, maybe cheesecake or something else, anything to do with it. And you just kind of scroll through the list, find the one you want. If it's not there, you hit the plus sign and you add in, based on all the ingredients. But other than that, it's just a simple entry box and you start typing what you have. Chicken or whatever, and then give you all the different options and stuff.

Moderator: So how about beverages? What do you think? Do you think there should be another category for beverages, like sugar sweetened beverages or coffee, tea, alcohol, or even water? Or they shouldn't be in the plate at all? Yeah, in the app.

Participant 5: I think it's necessary to have the water section, because it's very important. But I think we should have the limitation for eating coffee, tea. But I think it should be as a limit, not a necessary section.

Participant 2: Yeah, you can you can start breaking down things a lot. You know what I mean? You've got the grains proteins and I forget what the other one was there, a fruit. But yeah, I mean, do we add in another button for dairy, another button for beverages, another button for this? And eventually, you're going to have a whole screen of buttons. You've just kind of broken it down, I would think too much, or you could end up potentially breaking it down too much.

Moderator: Yep. What do you think Participant 4? Do you think there shouldn't - we shouldn't put values on these on beverages as well? There shouldn't be on the app?

Participant 4: Oh, the question with beverages is it's extremely variable and it depends on what you want to do as a nutritionist with it, right? If I were to drink three litres of water a day, I would be spending my day on the toilet. So some people need a lot of water, some people need a lot less water. It also depends on, well is it 30 degrees outside or is it minus 10? Am I exercising, or am I doing something else? So the problem with liquids is that your intake is going to be very different, based on what your level of activity is, the temperature outside and so on, so forth. Quinoa was notoriously bad at tracking that, actually didn't track it at all. And I just gave up, because it was just terrible. Liquids are also very culturally driven. So I will ask some of my French from France friends, oh they don't consider wine and liquid, it's just there. Very different for other cultures, you consider tea a liquid or coffee a liquid? The answer is well it's based on water, so of course it is, and it's part of your intake. So if you actually measure how much water you do drink in a day. I drink a lot more than just what's in here. But that's not counted. So if you're asking for all the details, getting back to Participant 2's point, and you start drilling down and down and down, then we get to the problem we had before, which is, you're asking for so much information, you're just going to discourage people from entering it in the first place. And what I like about the interface that you have is it's actually very simple. Maybe just have a button in one corner and just say, OK, well, how many liquids have you drunk, in litres, or whatever it is? And it doesn't matter what it is, because at the end of the day, a liquid is a liquid, is a liquid.

Participant 2: That's what the Fitbit app, when you do the Fitbit thing, they essentially have two categories, main categories. It's your water intake, or enter food. And it's that.

Participant 4: Keep it simple.

Participant 2: If you're not wanting to be very picky, as she said, water, tea, comfort, there's water in tea, there's water in this there's water in a can of pop. I mean, you just enter it that's a liquid. You drink a 355 millilitre tank of pop, you drank 355 millilitres of water, but then you have the other nutritional things you need to consider. I mean, the sugar that's in it and stuff. But still, as you said, water is water. Doesn't matter what shape or colour its in.

Participant 4: That being said, if you drink 355 millilitres of juice, which is notoriously sugar heavy, then you should account for that somewhere. A lot of people think that drinking juice is healthy. And we know that it's not. Excess fruit drinks, lead to the same problems, right. So drinking a coke or drinking a litre of orange juice, not a very good idea. And it has to be accounted somewhere.

Moderator: Any other ideas? What do you guys think - do you think that it should be on the app, tracking beverages should be in the app or not necessarily?

Participant 1: I suggest that I think you should put all of them in the part of other less options. Yeah, I personally, I don't like crowded apps with lots of things to enter. So yeah, I think you should be [unintelligible 01:02:31]. And I don't know four or five more than that.

Participant 3: I think it should be completely a different section. It shouldn't be mandatory to fill the parts in. Because some people might be curious about how much they take during the day. And some others might not be interested to know. So I actually not agree with other part with a completely different parts, I think is much better.

Moderator: OK, yeah. How about milk? What do you think about milk? Should it be tracked? We are, we're talking about dairy. You, some of you told us that there should be another category on the maybe others meals for dairy. How, how about, specifically about milk?

Participant 2: My feeling is, I remember a while ago, you had your four main food groups. One was dairy. They've removed that. Why? Who knows? Whatever. But to me, that's a main group. Dairy, because it encompasses so many other things. I mean, cheese butter. Oh, God, you know what I mean? Like creams, whipped cream, all that kind of stuff. It's all dairy why they removed it, I don't know. But to me, that's one of your main groups should be there as a main group.

Moderator: Yeah, everybody. And what do you think? Yeah, agree, not?

Participant 1: I definitely agree with Participant 2 about having dairy. Because it's important and I don't know, I kind of track it for myself every day to at least drink two cups of milk every day. I know that you're doing it for Canadian people, this app. But for example, I've seen lots of people in Iran, especially women have problem with their bone density after they turn 45 – 50, years old. So if you like it to have a healthy life after you turn 50? Yeah, I think that's important to intake dairy. Especially -

Moderator: Yeah, the app is not only supposed to be for Canadians. The idea is to be like, for everyone.

Participant 2: One thing to think about, like what Participant 1 was saying, was the thing about bone density, osteoporosis, I'm dealing with that. Because of the back fracture I have. It's not really necessary to have dairy to get your vitamin D and stuff. You need vitamin D and a pill as a supplement. I've watched the show I don't know who else has Netflix. But there's a great show on there called what the health and it goes over the things of meats and like red meat, how it affects your body cells by loading the fat into the cell, which blocks the insulin from opening up the cell to bring into sugar. It goes over all these - and it actually goes over to say about dairy that really, I mean, humans are the only ones that do, supplement their diet with another mammal’s milk.

Yeah, I mean, other mammals don't do that. We're the only ones who do it. And when you get right down to the nitty gritty of what dairy does for you, you really don't need it, you can get it elsewhere. And then you're not subjected to the hormones and stuff like that, that are in the dairy from the cow and stuff. It's a very interesting show. Like if you got Netflix, I suggest watching if you're really interested in your foods and stuff.

Moderator: Yeah interesting. Yeah, I'm going to ask it is the name of the documentary after? Yeah. So any other ideas?

Participant 4: How do you deal with snack Moderator?

Moderator: Oh, yeah, that's a good question.

Participant 2: Pick them up and eat them?

Participant 4: Well, the problem is it's an impulse, right?

Participant 2: Yeah, that's your impulse eating.

Participant 4: So how do you deal with impulse eating? I can't say I have experience with it. Because I don't. I don't eat snacks periods. I just had three meals a day. And that's it. I'm very boring. But I know a lot of people especially my wife loves to snack. So the question is, how do you deal with that? And how do you make it easy enough for somebody to enter that information? As they think of it, because when you are impulsive, clearly, you don't want to be slowed down by app. Yeah, I have to pick up my phone and I have to start entering stuff in it. No, I really want an ice cream or peanuts or whatever, it is that I'm going to snack on me.

Participant 2: I'll be honest, I use those apps, I will track my meals. But when it comes to the snack, it's like me. Yeah, no, I really don't want to know so –

Participant 4: There you go. Guilty as charged.

Participant 2: Yep, grab it and go forget about it. And then of course, that throws off all the measurements and stuff of what calories I'm bringing in and stuff like that. But as you said, you don't want to have to grab your phone before grabbing your first potato chip and dip.

Moderator: So yeah, speaking about impulsive eating, like having snacks. When -there were some other elements on the backside of the guide. Like being mindful of your eating. Do you think those elements should be tracked as well? Yeah, they should be included in the in this application?

Participant 4: If you do a lot of snacking, obviously you do, because it's – Participant 2’s point, it's going to have a big effect. Because usually snack foods are not the right ones.

Participant 2: Exactly. chips and dip. How do you know how many you're going to sit down and eat? Like how much do you know how much dip you put on each chip to total up to? It's just, you just don't.

Participant 4: Yeah let's look at Participant 1 smile there.

Participant 2: Everybody knows what I'm talking about. I mean, ice cream. How much is in a scoop? How big is that scoop?

Moderator: Yeah, exactly. So what are other elements, do you think about? Can you see the backside of the guide so that people could have more ideas on it?

Participant 5: Excuse me, about a snack idea. I think it's unnecessary to have a snack part in the in our plate. It's good to the app capable to automatically separate the snack into the - if it has any of three portion of the plate it put them in the plate and for about the other ingredients like fat, sugar or salt or anything like that, it can have a potion for them and have the limitation just the limitation for the day. And just suggest or remind people to or over the limit or in under the limit. And I think that's enough, it's not necessary to have the portion because they are not including our plate every day. They are not the necessary part. And also a more athletic the app is more convenient, and people enjoy using them.

Because in our busy days, people cannot put and we have a full time to insert all of them. The app, they should be able to automatically separate them in the three section three main section and other limitations.

Moderator: So let me double check if I'm getting it right, you mean that the app should recognise the macronutrients in the foods that you're consuming? And it will automatically guess which proportion it would fit into right? Like it should guess about how much protein does it have? How much carbohydrates does it have?

Participant 3: Yeah, for example, when it’s chips and dip. It has some carbohydrates part. It goes to the grain, I think of the whole grains, it goes to that part. And maybe I think it won't have any protein, maybe dip some, have some protein it goes to that part. And others will go in fat, salt. And we’ve, for the fat and salt part, we should have just a limitation. For example, it after you eating chips and dip, it mentioned you that you are consuming 80% of your –

Moderator: Like a notification, right?

Participant 3: Of course. Yeah.

Moderator: OK, yeah.

Participant 2: You could integrate like the daily values, that they suggest how much salt you should have per day, how much sugar per day, all those different things. You can get that information on most of the nutritional stamps on stuff where it will tell you. Under on the label like that in so much salt, and it's 2% of your daily value. You can be tracking that as well. And like Participant 3 mentioned here was the eating as a group goals progress mood. That is in one of my apps where it has where you can put your mood so that then you can look and see that well on the days where I'm feeling a little depressed. I'm eating more, but more of the snack foods stuff instead of the good stuff. When I'm feeling good, I eat more veggies than that. Yeah, I mean, so you can see the correlation between them.

Moderator: And how it would help you? How would you - when you see the correlation, does it help?

Participant 2: I would think because if you go to put in, say you're having a bad day, and you're just like me, I've had a rotten day today. You might go into the saying in go to put that in depression and you pop up a saying, warning you I mean, you are about to go over this because you're in a depressed why not suggest other things? I mean, other things that can help and stuff. Do this instead, eat this instead? Not Oh, there it is. Yep. Like it says here, like eat meal with others like that can really help. That's one thing that's covered in that documentary was the thing of when you eat with others rather than alone, you tend to eat differently. I mean, and the thing of cook more often, which means eat more often.

When it comes to a diabetic, you really don't want just the three meals because then your sugar spike, they come down and they spike again. And then they come down. You eat and then you snack and it's the goal is to keep the sugar at the same level. So that's something to be mindful of.

Moderator: Yeah. So how about other, some other support or instructions that should be in the application? Yeah, thank you Participant 3. Thanks for being here. Bye.

Participant 3: I'm so sorry. I have to work.

Moderator: No worries. I totally understand it. Yeah, please feel free to leave if you had any other plans.

Participant 3: Yeah, yes. Yeah bye all.

Participant 2' Bye, bye.

Moderator: So yeah, we were talking about the instruction and support. What other instructions or support do you think should be in the app? Some like, food, like personal side guides or like user’s feedback or tutorials?

Participant 2: Well, yeah, like you can do that when you're entering in a meal or something, you can have it where when they're entering, you can see on the other side, how it's going to affect the rest of the day. That happens with one app I have where you put it in, you can see well, I've only got this many calories left for dinner. Well, then you could ever think of knowing, looking at what I usually eat, try to do like a meal suggestion with how many calories I have left for that day, or what how many macronutrients I have left. Look at what I ate in the past, try and come up with something. I mean, like a meal suggestion thing, based on what you have remaining for that day.

Moderator: Great. Good idea. Yeah. Any other thoughts?

Participant 4: I would just stick to like just a simple tutorial at the beginning. That's –

Moderator: About the app how to use the app or -?

Participant 4: Yeah just the app. Because Keynote, for instance, didn't have that. You had to figure it out yourself. Which is fine. But it's wasted time. Something very simple to implement. More sophisticated stuff is, you said Participant 2 earlier that you know you're doing this for the beauty of it. Well you're getting a lot of it now, out of the application, if it starts suggesting meals to you, and how many calories you've got left, or whatever measurement you choose. So again, that's fine. But what you guys need to do is to think about what the purpose of the application is for the people who are going to use it. If it's simply a monitoring tool to raise awareness, then that's fine. Just provide basic feedback. If it's going to be a modification of behaviour modification tool, then that's another story.

Because you want to build a process in it so that the user experience goes from OK, I entered data. Now I get something in return. Whether it's an instant gratification, which is OK, well, I've got 200 calories left, or it's a, here's a recap of the day. And the system doesn't judge it just says look, in today you have consumed whatever. And perhaps show it against what a nominal or standardised value would be. And then let the person decide for themselves. Is this good? Is this bad? Does it make them angry or indifferent? Or does it make them mindful of the fact that well, maybe they had too many chips and too many pop? Ultimately, question is, what is it for?

Participant 2: That's what I was just going to say like, I know, I came in late. So I don't know if that was said, what is the goal of this application? Is it just to help people eat better and just track stuff? Is it to reward people help them lose weight? Is it like for weight loss? Is it - as Participant 4 said, what is the ultimate goal of the app?

Moderator: OK, so when you are talking about some competitions, or rewarding, you know, people like to be rewarded. So how do you think what features can you can help people to keep tracking this application? Could help them be safe to using this application? Like any games or competition?

Participant 4: Well that's more of a generational question, right? Yeah, it also depends on the on the medium that you're using. So very clearly, if you're asking Participant 2 or myself, you're asking the wrong people, because we were not born with hand phones or cell phones in our hands. And we're just trying to get used to the to the idea that it should rule our lives. Some other people just don't care.

Participant 2: I have to step away for a second, my kid needs help.

Participant 4: Yeah go for it. So the question is what - prizes don't have to be something material, information is often just enough. Information is just awareness that you know what, I probably ate too much today. And the app tells mean, yes, we did eat too much today for whatever it is. That's probably enough. That's probably 90% of where you're at.

Moderator: Yeah, right. So how about the peer chat or peer support or communities in the application? Do you think that could work, could help?

Participant 4: For sorry?

Moderator: The support groups or some communities in the application?

Participant 4: Well, now you're now you're specialising it, right?

Moderator: Yeah.

Participant 4: Because it will - these communities will have specific needs, right. So again, when you're designing software, you try to think of what is the ultimate goal? What is the person using it wanting to obtain from it?

Moderator: What if we specialise them? What if they would be customised for specific issues?

Participant 4: So that would be a good price, quote, unquote, a good reward, because they are looking for a specific goal, and they want to meet it. And that makes it a lot more pertinent and attractive to that specific subgroup to actually use that information. The question then is if the application is also for general use for you guys, as nutritionists to follow and track the general population, then that's going to skew the data. And whenever you go into data analysis, and you go into a very specific group, you know, you're going to have that skewing of the data issue, which then means you cannot generalise from that. And that would defeat your specific purpose as researchers.

Because you don't want to be there unless you happen to be studying the eating habits of military submariners, who spent four months under the sea and a nuclear submarine. That might be a really relevant subject, and really useful to the guys and gals down there. But I don't think it'll have an application to the public at large in Canada. Right, because I personally don't want to live in a nuclear submarine for four months under the sea.

Moderator: Yeah, totally. So how about sharing the photos? Like, yeah, you can see the examples over the chat box. We can see - do you think these features should be included in this application to help people keep tracking?

Participant 4: Yeah, photos, a nice additional piece of information. But again, from personal use, I forgot to take it half the time when I was eating right.

Moderator: No sharing like with others with your peers?

Participant 4: No. OK. So that's a different issue. Now you're moving into social media. So if you're turning the application into a social media application, where you might have a comparison of you know, hey, I this and here's sharing of a pledge or stuff like that, then that's fine. But it will become a completely different target. And you're going to skew people's behaviour. I travelled to Asia a lot, people will change what they eat at the restaurant based on its Instagram value. So they will order and especially in China, they will order the prettiest dish, not the one they want. The prettiest dish, because it really looks nice on Instagram, and they can send it. Or the most expensive dish and send it on.

And I'm looking at these guys don't really OK, yeah, well, that I had never thought of that. But that's a social media type of behaviour that's going to skew the data. And they don't necessarily enjoy eating that food. Because I asked them why the heck did you choose that you like that. Is it not really, but it looks really pretty. Well different motivation. And so you have to be a little bit careful about that. Because what you want is obviously, data that is not skewed. And that reflects what reality is on a day to day basis. Which is yeah, I ate too many ships or I drank too much beer or I drank too much wine or whatever the case might be and actually have people honestly give that feedback.

Moderator: Great. So any other ideas about those features that we are sharing in the chat box?

Participant 5: I think it's a good idea to, the app can record their photos rates or persons of fat, blood fat, and also something else, because they can see their change and their progress. And maybe it's encouraged them to continue with the app,

Participant 2: One thing to consider is if you're going to have the thing of pictures and sharing, you've got to remember that each picture is going to take up so much room on that phone. And so if it's a lot if you get someone hooked, and there's like, yeah, picture this picture, this picture this, that's more and more storage room they're going to take and not everybody has a phone with 128 gig. Mine was 384 gigs. They have an eight gig phone or something. And they're going to fill up quickly. Like why, what happened? Where'd all my space go type thing? So to avoid that yet, when you're looking at they take the picture and sends it to the cloud?

Well, now who is going to pay for that cloud storage? Developer has to pay for it. But like, then how did they get compensated for it? Now it's a cost driven app. There's cost to it or a subscription? I mean, things to consider there when it comes to that. Because I know there are apps that do chew up a tonne of room because of features like that.

Moderator: Great idea. So the last question is about how do you think the app. What features in the app could improve user’s confidence while they're tracking their diet? So like, I think the simplicity of the application is an important aspect for making people feel - so that people could feel confident enough to keep tracking.

Participant 4: Yeah, that goes back to the, what's in it for me question, right. So I think because [unintelligible 01:27:14] was talking about just that, which is it sort of gives me a limit or gives me a hint as to what I should be doing, right? Is that what you had in mind?

Moderator: Yeah, kind of right. So I didn't quite hear you well.

Participant 2: Me. Oh, I'm, I'm kind of stuck for words. It's kind of like Participant 4 said, it's back to the what's in it for me. Let me - do you go to an extreme thing? Well, if you in the app you're tracking, if the person loses 100 pounds, they get a $5 gift card or something like that. But then who's going to fund it? I mean, there's something in there for them. But just not everybody will respond to a, as Participant 4 said, a badge or something like that. That happens like in the Fitbit thing. You walk so many steps, you get a badge called the subway runner or something like that. And you get these badges. Yeah, it looks nice, but at the same time, it's like is it really worth all that trouble just for a little thing?

It's like I tell my kids that played Roblox and stuff. They want the money for all those Roblox so they can have a hat that they put on their character. I'm like, why that's just digitally goes away. If the cloud dies, why not take it and save up and go buy a new set of headphones or something like that something tangible?

Moderator: Yeah. So if there is no - any other ideas, any last ideas? We have reached to the end of our question? So can you think of any other features or any other ideas that we didn't ask about. Feel free to share them with us now or even you could email them to us or -?

Participant 4: I would just suggest maybe putting in a group feature. Going back to the sort of the social aspect. And, you may want to go into this with some friends. Not necessarily the entire universe, which is what social media do. Not Facebook, but maybe you want to do it with your brother or sister or parents or whomever and keep that into a small group and say, OK, well, this is what we want to get out of it. Whether it's awareness, or reducing calorie count, or whatever that that is. And then be able to share it within that small group that you designate yourself as supposed to social media, which then becomes this big monster, where you don't know where your data is going. You don't know what information is being shared and how it's working?

Participant 2: Yeah, and anyone that uses the Fitbit app will see that they've got that same.

Participant 4: Yeah, exactly. It's similar to that. Because you want positive reinforcement back. You don't want negative reinforcement.

Participant 2: Yep. You can connect your friends and you can share with them, or they have groups like I'm in a couple that one that is Canada, one that is diabetes. I mean, you can go and talk to people like what do you eat, to help with your diabetes and what meals and you can do that in that group, and you're only sharing with those people.

Participant 5: I think we can have some goals. And it's, again a specialise for the body of every person. And when the person makes their goals, they feel confidence to continue being in their diet. And also, I think it's good to the app to be able to define some food. And for example, one time we define food and insert the, for example, percentage of protein, fat, carbohydrates and everything. And after that, it will be familiar with this food. For example, I can cook a special spaghetti for example, or special Iranian food. It will know that food for the rest of life and I can use it very simple. It's not necessary to insert all the ingredients every time. I can – it will know that special foods, for example, special dessert or special foods.

Moderator: Yeah, you can even share the recipe of your food, of the food with other people.

Participant 5: Of course.

Participant 4: That's a very good point. Because that was my beef with quinoa, was with home cooking, which is 99% of my meals. It's impossible to enter the information and that was the biggest frustration. Because you have 20 ingredients that you have to enter. Half of which are not in the database.

Moderator: Yeah, enter once for life –

Participant 4: Very good point there.

Moderator: Yeah. So any other any last points? Any –

Participant 4: Oh, you're making me hungry.

Moderator: Oh.

[End of recorded material]
