# Supplementary material for: Perceptions of self-monitoring dietary intake according to a plate-based approach: A qualitative study
Source: PLoS One. 2023 Nov 28;18(11):e0294652. doi: 10.1371/journal.pone.0294652 (PMC10683993; doi:10.1371/journal.pone.0294652)
Supplement: S4 Appendix — (ZIP) [file pone.0294652.s004.zip › Anonymized GP Transcripts/Icanplate-gp-focus-group-8.docx]

**Icanplate-gp-focus-group-8**

[Start of recorded material]

Facilitator: So this is I Can Plate study focus group with the members of the general public on August 4^th^ at 7pm eastern time. So please let me know what do you think would make it easy or hard for you to eat in accordance to the food guide that we just showed to you? What would make it easy or hard? Would you do that? Would you eat in accordance to the guide or you don’t?

Participant 1: Well you can eat healthy like that, but not everybody has the time to do that, I think that’s a strong factor in what people put in their body so they don’t have the time for it in their busy day or a long week and they’re too tired to make a whole good meal to even think about what they’re putting in then it’s just not going to happen.

Facilitator: Right

Participant 2: Yeah just a follow up, yes and I know you know you see your doctor probably at least once a year and he more or less kind of says the same thing, but I mean everyone still you know every now and then you’ve got to go and have that Five Guys burger, but whether it’s healthy or not you know there’s a craving.

Participant 3: I think personally because of my situation I would be able to eat this way, but I don’t think that’s the case for everyone because not every place has access to healthy foods especially fresh produce. For example, in the US, there’s a lot of places where there’s food deserts and people just don’t have access to healthy foods or healthy foods are very expensive. So I don’t think it’s necessarily feasible for everyone and then another aspect is the taste. Sometimes, as others have mentioned, you just crave something else that’s not necessarily whole grain for example so that would definitely also be affected.

Participant 1: Yeah I know there is a lot of marketing with easily attainable items for people, there are food apps like Hello Fresh or other delivery companies.

Participant 2: And like with the food deserts Patricia, I think, was saying you know it’s the same sort of thing like oh you know it’s too far for me to go to a grocery store and get the healthy vegetables and fruit, but I can go to 7-11 I can get a couple of greasy Tekitos and you know that’ll take care of me and it’s right there.

Participant 1: Hey don’t get me wrong, 7-11 has good salads too. In the deli section, they all have one and it’s all [unintelligible 00:03:09] at checkout. Some other countries 7-11’s, they are chocked to the rim with like five whole walls of like deli stuff, all pretty healthy. Japan, for instance.

Participant 4: I feel there’s a lot unsaid in this poster. I don’t know if it’s a leaflet or poster. Everybody knows by now that these are healthy food and you need to be eating these. Nobody needs to be told that OK these are the healthy foods. I think what’s required as more I mean in depth thing on what not to eat and stuff rather than what to eat. For example, you have Tropicana juices. People think that OK great, Tropicana is a great juice and you will drink it, but then it has a lot of added sugar in it. But then that’s not as far off you know? It says on the back of the leaflet that food marketing, but then it doesn’t go into specifics on what you really need to watch out. I think that’s what I would expect from Health Canada’s you know, marketing or material and not just a thing say OK these are the healthy foods so that’s what I make of this.

Facilitator: Right. Yeah the aim of this poster is a guide, the things that it’s focusing on is more than, like eating in those proportions. Like having half of your plate filled with fruit and vegetables and a quarter of the protein for the [unintelligible 00:04:50], another quarter with whole grain foods. Yeah.

Participant 1: I think it lacks a little intensity with just the amount of diversity that Canada itself has for the kind of cultures that are within it. And I’m not just talking about the cultures that are natively here, but also of people who have different cultures who may be used to eating different things. Like instead of where the bread is you could have half of that slice of bread and maybe half a naan, same shape, but kind of put together so it looks more heart warming for people of different cultures. You know just stuff like that, but inclusive to the very many different aspects of culture that are within Canada. I mean times changes and you know, sure Canada has all these native stuff, but it looks like that’s native, grain’s a native, proteins and vegetables, but there’s a lot more than just that that’s available in Canada thanks to those efforts of those people.

Facilitator: So I’m saying that [Amine? 00:05:56] is sharing something about fast foods. Amine do you want to share it here and say to us about –?

Participant 5: Yeah. I just didn’t want to interrupt people, but the thing is that usually healthy food for the same amount of calories consider way more expensive price rather than junk food. For example you can always go for a McDonalds, I don’t know, ten to twelve, thirteen dollars for a meal and get like 800, 900 calories, but for the same amount of calories of healthy food you should probably get a salad which is probably going to cost you like $20 at least. That is one thing.

The other thing is that like there are way more fast food places rather than healthy food place which makes like the diversity of the available fast foods way more which results in like being appealing to more people eventually because of having more diversity of tastes.

Facilitator: Yeah you’re right. So any other thoughts on the guide?

Participant 6: Actually this question is very forward for me. At a personal level I used to eat this healthy, nutritious, balanced diet, but so far as a student it’s really so harsh to find the time and because we have so many ends to meet so it’s really too expensive to find healthy or organic fruits and vegetables and all of these things. So I’m with, as Amine said, with all of these restaurants and then like the fast food chain over the world it’s really harsh for us to keep this balanced diet.

And for other countries food becomes like it is a crisis since 50 years and it becomes worse and worse now with the pandemic and everything. So like some countries they don’t have the ability to have like, I don’t know, something displayed. So it’s like it depends on you, the time, the financial situation you have, the environment you’re living in, the country you’re living in. It’s like a lot of things. Like food is more than just like what we eat or what we like what we crave. It’s way more than that.

Facilitator: Yeah so I think John was going to say something, yeah.

Participant 7: Yeah. I just have one problem with the food guide. The bottom right hand corner is choose whole grain foods, but I for instance, love wholegrain, wholewheat bread, but that’s also a good portion of protein and it’s not mentioned so when I’m trying to figure out what portion of protein am I actually taking in each day. I love wholewheat pasta, I love wholewheat grains, I’m already getting a good portion of my protein which doesn’t show under protein in this plate mix.

Participant 8: Well your body processes proteins, basically just turns them into sugars anyway. I don’t think it matters that much.

Participant 7: It’s still trying to figure out what number of, how many proteins, or what percentage of protein I’m taking in each day. If I’m supposed to be getting in a certain number of grams of protein then understanding exactly where I’m getting them helps.

Participant 8: OK so you’re into that right? So you’re someone who would like record all that. I think that’s good for you to be part of this because this is, it can help address some of that.

Participant 7: I wouldn’t actually record it. I wouldn’t record it, I’m not –

Participant 8: Would you be interested in knowing it though?

Participant 7: Yes.

Participant 8: OK. Perfect that you’re here.

Facilitator: So I see you nodding, Howard. Do you want to add anything?

Participant 9: To some extent it’s almost, to my mind, incorporates what’s being said. It’s rather, well we’ve identified barriers, barriers in terms of time, cost in this sort of thing and it sort of puts it out at you and to some extent it’s a challenge and if you don’t meet it then you’re almost shamed into going an alternate route. So it’s sort of almost too simplistic. I would like to know the time, it was discussed about the time to go buy vegetables etc. that can be measured and also the cost that can be measured. And so it would be very easy to have plenty of vegetables and fruits, but very quickly here’s the added time or cost to it and then really it’s up to the individual to decide if she or he wants to make that choice. The other choice of course is calling it fast foods, but it would be helpful I think just to measure the amount of effort it would be to follow this guide.

Facilitator: Yeah totally. Time and cost are really important factors. So what’s, if you want to make some changes to your diet and it’s more healthy, what techniques would you use to stick to those changes? What techniques would help you? Like would you set your own goals? Would you do support groups? What techniques?

Participant 6: Actually I have a question which I’m not sure if you can answer it, but what exactly is considered healthy foods and I guess how much do we know in terms of nutrition and diets about what different foods do to the body? I’m not really up to date and I’m wondering if is the science definitive? Do we know a lot or is it still something in progress?

Facilitator: Well that’s a really good question. So I think, yeah we should not talk about any healthy or unhealthy foods here. So the main goal is how would you make some changes into your diet? For example someone decides to make any changes, any changes that they want. I want just to know how would you stick to those changes? How would you monitor yourself to seeing that way?

Participant 1: So a classic example would be a type 2 diabetic that has an elevated sugar or haemoglobin A1C, the two to three month moving average. So the Canadian Diabetes Association has very good resources as does other ones as well. Education, give people the knowledge, most people have a good sense if they’re eating too many sweets or fast foods or that sort of thing, but it’s really rewarding to see people use the knowledge and then get excellent results. So to me it’s very much education and then personal choice.

Participant 2: I think it’s the money for me. And having the time to go out and remember exactly how much I need of this and how much I need of the other thing. That’s the biggest thing for me, if I can plan out that I’ve used this many vegetables and not so many meats or whatever or like mushrooms and that I can go to the store and do my daily business catching up on what I need, but at the same time it’s having the thing about the cost of those materials.

And you know, if you’re going to do that twice a week which is how I usually get groceries which is the average for most people that’s, like they’re not going to have the time to do that and check their fridge, what they have, they’re going to try remember and that’s what everyone does. They try to remember, but you just miss things now and then.

So it’d be nice to have something to say OK you’re up on this or you’re low on that and make sure you grab a few of these or this item will compliment this stuff that you’re getting, why don’t you try some of that. Or you’ve been eating a lot of this stuff with your meals, how about you supplement something else for it instead and then just to change the pace a little bit. Like suggestions for the better, but not necessarily a forced instruction to do something.

Participant 4: I’m not sure which question you’re answering, the barrier or the technique? The barrier right?

Facilitator: The techniques, yeah.

Participant 4: Oh the techniques. So a common misnomer that I’ve seen most people here address is that the need for changing to a healthy diet costs more. I can personally say that it didn’t. I have recorded results over the last two, one and a half years. So I wasn’t [unintelligible 00:15:28]. During the pandemic, when it started, I decided to experiment going vegan because that was the trend.

I used to see that everywhere, everybody’s going vegan and that was the in thing. So I decided to try it and then I decided to do it in a well phased out way. So I removed certain eggs slowly from my diet. I replaced it with chickpeas, chickpeas powder. It looked like egg, but it’s not egg. And then the meat with soy. Various different changes.

I did it over a period of six months and then I went completely vegan and then what I realized is the common thing that people say, oh it’s very costly buying vegan food or even vegetarian food for that matter. It is not because I calculated my bills from what I was eating earlier to what I was eating right now and then I found that it’s actually a little cheaper now than before when I was eating unhealthy food because I replaced nearly everything with healthy options.

I’m not saying that I don’t eat unhealthy at all, there are unhealthy at all, there are unhealthy foods even and vegetarian and vegan foods. All these patties that they market, the beyond meat ones, everything, all of them are unhealthy in its own way because it has way more sodium content than what is relative, it says three person and then –

Participant 2: But you’re making this stuff yourself right? Like the vegetarian meals you’re making yourself, you’re not buying the pre-made stuff?

Participant 4: No I am buying pre-made stuff and I’m cooking as well. So I’m doing both. So I did buy these patties and stuff initially just to try it out and then I realized that it’s just a marketing gimmick, the entire thing. It’s much easier to get other alternatives.

Facilitator: So how did you calculate those, like how did you keep yourself being in that way? Being a vegan. What techniques did you use? Did you use like [tanning 00:17:38], did you write them down? What did you do to [unintelligible 00:17:41]?

Participant 4: I was following a lot of Instagram accounts and I was influenced by it I would say and it was an experiment to be honest. I didn’t imagine that I would take it through all the way and follow it. I just thought OK let me just try it, let’s see what this entire fad is about. And then when I started this journey, I knew that it’s very difficult. So I think in the first week I tried cutting it down completely, then I realized no that’s not going to work so I decided to cut down each thing little by little. That was an experiment that I did to myself.

And then even milk. Milk was the hardest part I think. And then I experimented with different kinds of milk which gives me the taste of the real milk and finally I arrived at oat milk. So a lot of research went into it, but then I liked it as well. So what are you going to do? You need to be completely dedicated to that. You should want it. Unless you want it you will not get it. If you just want to do it for the sake of doing it, that’ll never happen.

Facilitator: Great. Exactly. So you just used the like professional support or group support?

Participant 4: Instagram was my greatest supporter.

Facilitator: Yeah. OK thank you for sharing that with us. So John, do you want to say something?

Participant 7: Yeah. Three things worked for me or still work for me. I measure, but I only measure the things that I’m worried about so I know that certain things are higher calories. I love walnuts, I love almonds. I love pumpkin seeds so I know that I have to be careful. I can’t have three handfuls of them and they’re easy enough to measure whether I’m eating them along with cottage cheese which I also measure and cheese. But that’s pretty much it.

I also secondly, when I can, cook in big batches. So last weekend I made mushroom and lentil soup that I really like and it gave me eight two cup portions, six of which are sitting in the freezer. So frozen food really helps me when it comes to fast food. I can keep salmon fillets in the freezer and they take two minutes in the microwave. Same if I’m going with salmon. I love sardines, all of my choices are the foods that I like so it’s so easy for me to pop open a can of sardines, get lots of great nutrition and mix it with a bowl of frozen brown rice.

And then the third thing that I do, not as often, is rely on the website Nutrition Data to be able to look up you know, if I’m looking at making a choice between a recipe which is going to be better for me, walnuts or almonds or pumpkin seeds etc. It gives me at least a quick overview even though I’m not a nutritionist and I don’t get the full import for what it means.

Facilitator: Great. So I see that Jeff is sharing that writing down the goals would help. Do you want to tell us more about it Jeff?

Participant 2: Well you can write down your goals, daily goals, weekly, yearly. If you write them down, maybe say them out loud to yourself, share them with you know a group of friends or whatever, like I want to eat healthy. I’m not going to come and have beers and tacos next Friday because that’s not good for me. I’ll still come, but I’ll have something healthy. As long as they know and you know having some way of accountability as well.

You know, at the end of the day this is what I ate for the whole day and you have an idea mapping that out. And kind of like I said, I know a friend, you know she does a weekly meal prep you know so like Wednesday guess what? It’s mushroom and lentil soup for lunch and guess what is next Wednesday? Mushroom and lentil soup. No apologies, John, that’s really good. I mean she does it, but like everything is just, you know you open up her fridge and she’s got all these containers and that’s what I’m having.

Facilitator: Great, yeah. So Armine was trying setting goals and writing them down as well, but it seems as though they were not successful. So how about setting rewards? Are they working for you Armine, right now?

Participant 5: They’re not that good. I had the same probably as Jeff mentioned. Like I set a plan and just repeatedly ate something for like Wednesday, Thursday and it got boring and I was not following it anymore. I was thinking OK, how can I do something to follow it?

Participant 1: I think having a goal really works well with that. Like if you want to get to a certain weight. Say you’re 130, you want to be 140, you know you’re going to have to eat more. If you’re only planning your meal preps around like say if you do meal preps around the weight you have, what you normally eat, you’re only going to end up at 130 at the end of the month even because that’s what you’re prepping for, you’re not prepping for something more.

You have to say OK maybe I need 130. Let’s look at the percentages of what I’m using and maybe the energy intake for making all of it put together and maybe have to make that amount for it. Like I have to make more for instead if I want to end up as 140. But in a reasonable way, like not overloading your body and getting an overstuffed stomach and not getting the nutrients in kind of just to go the other side. The goal is really important.

Facilitator: Yeah exactly. Yeah.

Participant 4: Another thing is like when I was trying to deal meal prep, like I usually did them for four days, five days. Get the first day, the second day was alright, but it got stale after some point. Not really stale because it was in the fridge, it was not bad honestly, but it was not as good as it was on the first day. So that’s another problem I have with the meal prep plans. And I have not figured out any solution for that yet.

Participant 2: I find there’s always food in my fridge that goes stale too because I’m not using it because I’m always eating other stuff. Like I’ll start a bag of something like a bag of bread and then I’ll just eat the bread. And then when there’s other stuff and healthy in the fridge which I can maybe incorporate with it.

And I just forget to incorporate the other great stuff. Like I have veggies from my mother, that’s like radishes and beans and this and that. But I haven’t even touched into it because I’ve had cans of beans and regular potatoes and you know, frozen veggies that I’m just used to. So the reminder isn’t there for me. If there was someone that I could say OK I have this stuff in the fridge or I could scan the receipt that says, pulls everything up automatically and say I want to have a preference of one thing over another. Like say if I want to eat the bread or eating something right now I’d like to be able to select that in an app and have it say back OK well there’s this other stuff. You can make this and that and this with it or this can be made for that or if you want something for a longer period, try making this larger cooking meal. Or if you like to cook how about this other more intricate cooking meal?

Facilitator: Exactly. Yeah so setting goals would definitely be helpful for some people right? So any other techniques that you can think of?

Participant 3: Yes actually I brainstormed and there’s a couple of things that made me adapt how I ate. The first one was health reasons. When I switched from living at home to living as a student away from home, due to personal circumstances I got anaemic and so I had to adapt my intake to get more nutrients to recover. So that definitely made me more aware of what I ate and influenced my decisions and made me stick to them because if I didn’t I wouldn’t feel good.

The second thing that helped me was having a role model in the sense that I am surrounded by my sister who cooks really well, really tasty foods and eats very well. So having someone, and also my parents always cook very healthy foods when I grew up so being surrounded by people that can teach you how to do these things is very helpful.

And the last thing, it’s kind of tied together, but being on a budget and also wanting to eat tasty foods definitely encouraged me to learn how to cook different recipes. And yeah, that’s pretty much it.

Facilitator: Yeah. Great techniques. Any other thoughts? OK. So have you ever tried journaling? Like using an application to write down what you’re eating to record your food intake? Have any of you tried an application? Yes John

Participant 7: I have once for a very short period and it was actually for a study that your lab was doing. And the journaling methods, there were two different methods, one was to draw a plate and to place on that plate where the different foods that you had during the day so whether it was going to go into which of those four quarters. And then the second method was straight forward, a complete journal, line by line of exactly what you had eaten, who makes it, what additions whether it was mustard or whatever. And I think that lasted for about two weeks and I couldn’t do that regularly. I’m just not that dedicated.

Facilitator: So what do you think made it hard for you to so that you don’t want to continue that?

Participant 7: I found myself thinking about the food and how to describe it too much as opposed to actually preparing it and enjoying. I know I have to think about it before I prepare and portion sizes are hell especially if it’s pasta because it looks healthy before you cook it, but by the time you get it into the plate it’s three times the amount I thought it was going to be. And I was brought up as a kid to eat everything on your plate so that’s something I try and correct, but the journaling for me just doesn’t go with my personality.

Facilitator: So both methods that you tried were paper based right? You did not use a smart phone application or a website?

Participant 7: No.

Facilitator: OK how about everybody else?

Participant 6: Actually I tried one time for a couple of months or three months an application. It’s not journaling, but you have to put what you eat in the three meals or the snacks. So in order to calculate the calories you have on daily basis. It was useful for that time, but I just stopped because like as John said it’s like you have to stop and think OK I ate this at breakfast, if you couldn’t just like put it directly or before the breakfast. I just start writing what I’m having in front of me. And then like it’s like I don’t know. It helps, but it’s a mess, kind of a mess. I don’t know. I just stopped it. I just like –

Facilitator: So what was good about it at first, at the first place?

Participant 6: Just to see the colors that I’m having. And I got shock because most of the times like I didn’t reach the 1500 and then I was like OK why am I gaining weight. You know? This thing like OK I’m not eating that much and then I really got shocked. I put every single thing I ate, even the snacks and then I didn’t exceed 1500 calories and I got really shocked because I’m gaining weight.

I know it’s like [unintelligible 00:30:55] because now everything is online as students and then everything, the stress. But this thing really shocked me. And then you have to upgrade so again you have to pay for the application if you want more information, more helpful things and if you want to take a picture for the plate so not to write down all this stuff.

Facilitator: What was the application that you were using?

Participant 6: Fit Pal?

Facilitator: My Fitness Pal?

Participant 6: Yeah that’s it. That’s the one.

Participant 8: Touching on that, I think people need to know exactly how much [energy? 00:31:35] they’re getting at, what they have for intake and as opposed to what is going to be excreted. You know like for say drinking juice, you’re going to let go so much liquid for even – Or food you’re going to let go so much of physical waste. You know that’s going to affect your weight. I mean of course if you have some of that you know there’s many different things people do from also expunging frontwards. It’s not a really healthy way, but you know if people had more info about how much to take in, maybe they wouldn’t feel that they need to go those other extra routes and it would just be a healthier environment for all.

Facilitator: So have you ever tried one of them?

Participant 8: Tried an extra method? I mean like I’ve tried supplement build.

Facilitator: I mean an application.

Participant 8: Oh an application for that sort of thing? No, I’ve thought about it, like doing like weights [unintelligible 00:32:40], but it’s just not really for me to try that. I just can’t handle that.

Facilitator: OK so anybody else who has tried one of those applications?

Participant 4: I didn’t try any application, but I was sharing the things that I was eating with my sister through Google [unintelligible 00:33:00] and then she also has to share so I don’t know if that counts, but then yeah. I tried recording the total number of water and the glass of water recorded there, all that. It’s just too much effort for when you think about like why and it’s like nothing. So you just give up. It’s just extra for doing all this.

Facilitator: Yeah. OK any other thoughts?

Participant 6: Also I used the same one as Navine, My Fitness Pal, for not a long time, maybe two or three weeks, but I didn’t like it because for a couple of reasons. For of all, looking back on it I think the reasons why I started using it were already out of self-consciousness so that’s not a good place to start. And then I think it kind of changed my relationship with food and I think that’s definitely not something I should have done because I started doing food as something to calculate and I started focusing more on what the application was saying and what I thought as opposed to what my body was telling me and what I was craving or eating and so I was not hungry anymore or not eating when I wasn’t hungry.

Facilitator: Yeah, right. Great. So do anyone a diet self-monitoring tool that currently resembles the Canada’s food guide? Do you know any application? Any tool? Heads shaking. OK so it’s a no. How do you view the app [unintelligible 00:34:56] when you think about your eating throughout the day?

Participant 2: Well I think that it could be vastly improved by adding like a selection after you have the initial pie diagram set out. Like have a slider that you know like on slot reels? Something that goes back and forth and you select left and right and it just keeps rotating around? Like have common varieties of whatever that pie is depicting and be able to let them slide with their finger so they can just rotate it around and quickly get to what they want so that they can make it more specific so they can get a more detailed look.

Like Howard wants more detail in his life right? This would be a good option for him and it would be simple because once he already has the selection laid out he has automatically basically the amount on the plate that he has. So he has the dinner plate, an average dinner plate and it’s pretty average for most areas of any country except for America is going to be slightly larger there which is actually studies are just slightly larger. But that would help to ascertain a better look at their dietary intake.

Facilitator: Yeah exactly. So any other thoughts? When you think about tracking your diet in this application throughout the day considering breakfast, lunch, snacks, dinner?

Participant 4: I’ll just tell you a pain point that usually people have. When people try to record, that is, when you’re eating a dish, say you’re buying it from outside, are you going to analyze how much of grains this has, how much of this thing has? You’ve just got to eat it hot right? You’re not going to analyze oh OK this has this, this has that. No, you’d rather not record and once you missed that first recording that’s the end of the application.

It’s going to get [unintelligible 00:37:10] soon. So it’s all about that and to have the consistency either you watch, you put extra effort each time to record and do everything or else you eventually give it up. There is no middle ground in these applications, either you do it or you give up. I mean this is from personal experience. It could be different for each person so that’s just a pain point of an [unintelligible 00:37:39] so that I’m telling you.

Participant 7: I was also wondering, how do you figure out for instance for breakfast, if I have a slice of wholewheat grain toast and a boiled egg, is that 50/50 and how did I come to that conclusion? Was it the calories in each? That’s the question that I still have.

Participant 1: Good point. I think the slider would address that because then you could pick specifically say you had a selection for breakfast, like a tab at the top of your screen for breakfast where it’s like the home and the water drop. Instead you each could pick breakfast, lunch or dinner and have the common items there and be able to quickly pick them. And that would suit that need. And so you wouldn’t need to know exactly how much you’re getting because you’re trying to find out how much you’re getting, you don’t know. So a pie graph isn’t really the best thing for that unless you’re looking at how much you’re seeing on the plate and like just what it is on the plate and not [unintelligible 00:38:38] values. There should just be a different way to select them.

Participant 7: M’s suggestion actually does come from I mean. I think his suggestions would make it far easier for me to use.

Participant 1: I mean this is just very popular things I see in every thing that’s happening that’s gaining popularity from discord which has nothing to do with dietary nutrition, it’s just a chat app. But it popped off, like it took off with popularity because you had so many ease of access and usability features for the end user. I mean that’s what people want and if they see that and they have their accessibility, I don’t even mean for like disabilities, but then they don’t really have a reason to complain or to say hey, this is not really working for me. You know if they have immediately what they need in front of them or what they’re asking for in front of them and like a small picture or something that’s easily visible, that’s really what they want.

Facilitator: Right, exactly.

Participant 7: And just one last point, if it just says grain, what does that tell me if I’ve had the worst white bread that sort of you give it to the rats they’re going to die versus a very nutritious whole grain bed? Should that somehow be represented?

Facilitator: That’s a really good point. We’re going to ask about other food that –

Participant 2: I’d say that’s regular [unintelligible 00:40:12]. Yeah.

Facilitator: Yeah exactly.

Participant 3: Actually I had one suggestion sort of a thing. So in the flesh, if you have an option where you choose the bread, like you have different kind of stuff like say wholegrain bread or something like that you choose that and it automatically fills up the nutrient content of that one slice. And you add how many slices? Then it’s much easier for the end user right? Instead of knowing oh OK this looks like protein or this looks like something else. This looks like bad instead of discerning all that, it’s much easier if there are pre-made options that you can choose from. For example, rice. It has multiple nutrients so then you just have to select that and then went on with it right?

Facilitator: Yeah the app [unintelligible 00:41:02] proportion [unintelligible 00:41:03].

Participant 1: Yeah should you do a live update like if they’re seeing on the front face exactly what they picked and say if they want more or less maybe let them zoom in and out with their finger and have the size of the item increased or have individual stacks increase. Like say you have one piece of toast, say you zoom in on the toast. Then it’s not zooming in on the plate, like the plate is set, but because you’re zooming in the item it’s putting it from one toast to toast to three toast or whatever.

Like five grams a piece to ten grams a piece or more and it’s visually updating it in front of them on the plate so they not only can adjust for seeing different nutrient contents, but they can, like if they’re selecting an item they can manipulate it on the plate to make it the exact size visually on the plate.

So they don’t have to count every piece, they can get a quick view of the plate, they can get a quick view of the circle and they don’t have to get a giant tablet that’s the size of the plate, but they can see in rough comparison what exactly is there. And that’s easy for people, that’s something they teach in grade one, kindergarten some places. Like OK you got this hole, that thing fits in it. OK make this circle, make that circle, copy this shape, gain some dexterity.

Everyone has that you know, it exists with everybody and I think that’s a great, future proof option because if you get a new item in all you’ve got to do is take a giant picture of an item and then slowly size it down on the edges to where it’s like one P or two P and then you can increase the image and then have it be a larger image. You don’t have to Zoom in on the image, but the image itself widens.

Facilitator: Yeah, great idea. Thanks for bringing that up.

Participant 1: I mean there’s other options like going 3D which is just over the top. I mean you’re just using too much energy at that point and it’s too much electricity usage and CPU usage. People’s phones are going to shut down and you’re going to get complaints. But like a 2D image, like people can relate to that and that’s easy for your phones, easy for your servers and you can have mor people use it, you can upgrade equipment better because more people will be using it and it’ll be more accessible.

Facilitator: Great. So any other thoughts? I mean do you have any ideas how to track the single, like for example if you’re having an apple, how to track that? You’re seeing that this plate won’t work with those kind of foods right?

[Unintelligible 00:43:48]

Participant 5: Yeah but a selection will help, but like for other meals that you’re eating during the day, for example I’m eating some chicken breast with some veggies, how shall I calculate the ratio and insert in this app?

Participant 1: Maybe have something that says “Pick the meats” right? And then they have one chicken breast and then you add it to the plate from the menu, not from the plate itself.

Participant 5: How you cook the chicken is very effective.

Facilitator: Yeah. It is really important.

Participant 5: And like what kind of veggies are you taking? Like how much water was included in them when you were [unintelligible 00:44:24] and there are a lot of factors.

Facilitator: That’s true. I know in another app they have other option sections on every food item that you put in and it’s like all these different things. Like if it’s cooked too burnt or if it’s cooked well done or if it’s medium rare. Like there are different protein values and I saw that and I was like OK well that’s, yeah interesting because it kind of went down in protein as more got cooked, but also lost moisture and they did calculations for that. And they basically went and said OK you might need to drink more juice in your day because you’re eating dried out fruits. And that was very cool for me.

Participant 5: It’s very cool to have that option of like how much have you cooked a specific kind of meat, but like everything is organic.

Participant 2: Yeah. [Unintelligible 00:45:11] or if it’s like grain fed or grass fed foods.

Participant 5: The other thing is that like shall we weigh everything that we eat before eating them? Like if I’m eating some veggies should I weigh them?

Facilitator: Weigh before tracking?

Participant 5: Yes because how else would I be able to?

Participant 2: I think you could. I mean you can weigh them I suppose, but I mean if you’re looking at an average portion size it’s going to have an average weight.

Participant 5: Oh yeah an average weight.

Participant 2: Yeah so I mean if you put a size of something on a plate that says some width and diameter right? It’s going to have an average height, average width, average length and you’re going to get that approximately at the right size with the visual comparison then it’s probably going to have the right weight.

Participant 1: Yeah like they always say it should be the size, your chicken breast should be the size of your fist or something like that. And all of that. It should fit in your palm, that’s what they said. It should fit in your palm.

Participant 2: Oh that too. Yeah there’s all different ways of saying it.

Facilitator: Actually our next question was exactly about how to measure those portions on the plate. Yeah you just jumped in to our next question, it was really great. So any other ideas about the portions other than weighing or palms or fists? Anything else?

Participant 2: I think weight isn’t accessible for people. Like not everyone’s fist or palm is the same size, but the plate itself is always going to be the same. Like that’s always going to be something that’s not going to change, it’s not going to grow over time or shrink over time. You’ve got to imagine like your app working for thirty years.

Like people are going to change from kids and you know they’re going to be small, teenagers and a lot of different changes and then you know they’re going to grow, they’re going to get bigger then start to shrink again. So thinks might now work all the time. Right? I mean if you have the option [unintelligible 00:47:14] adding in their weight and what their goals are etc. and maybe the age range and how they can supplement various other things in their diet for themselves which would also help. Then you can get really in depth and really start to help people to get nutrients for their body.

Participant 7: With a lot of items for me measuring cups work. I have a set of nesting one cup, half cup, third cup, quarter cup and I sort of get used to. I know that the veggies that I want are probably going to be half a cup and I have a basic idea of thanks to again using frozen vegetables, I have a good idea from the back of the package what the nutritional value is going to be for each of those portions and I don’t have to go back and try and eye it or figure it out. And I do that with probably 60 percent of the things I eat. The others I do [unintelligible 00:49:19].

Participant 1: That’s probably a better thing than that what I do because I usually just look at a plate in the exact pie method that she’s kind of doing. But sometimes stuff is going to get piled too high and I realize that I don’t really measure it out when putting it in the pot or whatever. But like stuff just goes back in the fridge and I’m like oh man, I’m not eating fresh food, it’s like not great for me, I’m eating like left overs.

But that’s what happens for me you know, so if there have a way to measure how high something is instead of just how wide it is I mean I might be able to control my usage of food that I’m cooking. If I can lay frozen stuff out on a plate before I cook it I can lay out it perfect for when I’m eating it and that’s helpful for me because I can do that and put the rest back in the fridge and then I’m saving food and I have something fresher later.

Participant 3: So another way of recording this, so for example, again this I used [unintelligible 00:49:23] so if I buy say 100 grams of tomatoes, if the app has an option where I’m only going to cook half portion of it. So automatically it says there OK 50 grams of, no, no tomatoes are going into this and calculates [unintelligible 00:49:43] accordingly right?

So for example Roma tomatoes has a different thing from say the chunky one, what is it beef stick tomatoes. So if there are options, the premier options wherein you can already select based on what you buy because these are not things that you need to weigh each time because you know what I already bought. And if there are things remaining, for example this can also be the shopping list [unintelligible 00:50:09]. You’ve bought, for that week, you’ve bought say 1kg chicken, say half a kilo of fish and say 1 kilo of broccoli. I’m just giving you an example.

And then you just cook one portion of it, say one cup of each thing. It’ll calculate whatever is remaining and what is already there cooked. So a lot of [unintelligible 00:50:34] portion it out and then give you the plate instead of you know you inputting each time because from an [unintelligible 00:50:40] perspective you want less work right? Because time is what it takes, very app, every thing is trying to catch your attention and you don’t have enough, there’s only so much time right?

You’re going to spend more than five minutes doing this each day or each meal, you’re going to give it up so I think from a product manager standpoint, who’s going to create this, it’s less time, more premier options and still you know, you have to see the benefits right away. Otherwise the user [unintelligible 00:51:14] won’t be there for the application.

Facilitator: Great. So in the interest of time I was just motioning that maybe our session will be a bit longer than what we were expecting. So if anybody wanted to leave in I think it would be 8:30 easter time, please feel free to leave the meeting, but we’d appreciate it if you could stay longer to go to the other questions and make sure that the environment would be like so that everyone could share their ideas. So yeah, any other thoughts? Does anybody want to share? Navine I think you were going to share something?

Participant 6: Yeah. I have a suggestion actually, I don’t know if it’s feasible, but it would be very practical if the users could take a picture of their plate and you would design an algorithm that can recognize the food item. I’m not sure how it would work, but maybe based on the reflection, the color, the shape etc. And then [unintelligible 00:52:24] database with nutritional values and then put together the estimation of the plates.

And then another comment was, from what I understand, this application wouldn’t be so focused on the details of what exactly someone is eating, but more the overall proportion and making sure that people are eating a lot of veggies and not overeating grain for example. Or only eating one food group in which case I think getting into the details of how high the food pile is or what type of food exactly is on the plate is maybe not as important because people will stop eating when they’re full.

And I guess we are, I mean plates are a certain size. I don’t know how to explain it, but if you eat in a regular plate or regular bowl, unless you’re really really planning your food, I don’t think most people would have issues with that although I could be wrong.

Participant 2: No she’s right. I’d totally do that. [Unintelligible 00:53:29] have all sorts of different things and I don’t know really how much I’ve been eating because I don’t have a good look at what I’ve been eating. Like small bowls for just eating a bunch of random stuff, but then I’m left trying to remember what exactly is all around and stuff that I ate small bowls, that doesn’t really relate to the common normality of eating from a plate.

Participant 6: But what if you’re having a smoothie for a meal? A lot of people have smoothies for a meal right? Protein shake. What happens then? That’s also there.

Participant 2: Maybe add it to a snack category or like a dessert category and have like maybe –

Participant 6: But that’s also a meal in itself if you follow different diets.

Participant 2: Yeah it is a good meal if you can, if you get a good one. There’s just lots of different shakes though, it’s like the milkshake and there’s the vegetable shake. I mean there’s different varieties, but if you put like an option for shake and then have the different varieties on like a side of the selection that they can just go click click with their finger or whatever they’re using on the app then that’ll be easy for them.

Facilitator: Yeah so speaking about those other foods, we’re going to talk about our next question which is how to track the other foods that are not in the guide. Like the, I know that the bread, like white bread that John was mentioning, the unhealthy bread. Or like some desserts, I think [Gwen? 00:55:02] was mentioning about the desserts that were not in the guide. How do you suggest tracking those foods? Like fat sources. You could see them on the chat box and how do you suggest them to be tracked?

Participant 2: Well I know a lot of people are very picky if they’re considering nutritional values. And they really will stay away from some sort of foods. Now you can always suggest some foods to them in their diet as like if they’re eating one certain thing and they’re putting in their meal a lot, maybe they just really like it, maybe you’re not meant to take note of that and work with it.

Other times they may be more accepting of incorporating something else instead of that one item because they might get tired of it and that’s just what happens, people’s interests wane and it goes towards other things. You might be able to work with that with giving them a new suggestion at that point and saying do you want to work this into your diet more or do you want to more often work things into your diet that are different than what you normally eat. So maybe you’d be able to give those suggestions more often.

Facilitator: But they’re not necessarily always unhealthy foods. They maybe like mixed dishes. How do you track a mixed dish? Or they could be like supplements. How do you track them?

Participant 8: Maybe if they’re always doing one meat thing say, hey, would you like to try different meats? Would you like to try a mushroom instead? Or something vegan or something [unintelligible 00:56:33]? Do you want to put those supplements with your meals or are you including supplements with your meals? That’s one way of going about it and then say OK is it this vitamin? Is it that vitamin? Do you have the values of the vitamins that you had and then how much is one vitamin if they want to go that in depth? Now that’s not really something people normally do. Normally [unintelligible 00:56:57].

Participant 3: There is a bar code reader, is there is a bar code reader and you just tell the portion it would automatically calculate it. It’s what you [unintelligible 00:57:10].

Facilitator: Many foods aren’t necessarily packaged. Like some international foods.

Participant 3: Since you talked about supplements so everything comes though Health Canada right? So a [unintelligible 00:57:24] or something of that so [unintelligible 00:57:28].

Facilitator: Yeah John I think wants to share something.

Participant 7: Perfect example, I live within 15 minutes of this bakery and it’s absolutely superb. They grind all of their own grains, they make 20 different types of bread, only one of them is wholewheat. All of the others are just as good and their baguettes are well guaranteed I feel good because I bought the wholewheat one, but I’m bringing home at least one baguette at the same time along with it and it’ll be gone before the next day whether I have it with peanut butter or with cheese and a glass of wine.

But again, as you say, it isn’t necessarily part of the meal, part of a regular meal. And I’m not sure how to measure that unless you have x number of meals per day and snacks and then other.

Participant 2: Yeah that’s a difficult one. I mean you’d be measuring the slices if that’s [unintelligible 00:58:27] like how wide are slices and I mean if you had to ask the baker for all the details on the food they might give it to you, but a lot of them are kind of secret about how much of what they put in their breads, just because it’s trade secret stuff and that’s how they make their income, but I mean being able to measure that out with the duration of how long they cook it, I mean that kicks water out so that’s going to change it.

I mean you can go by the how well it’s cooked and how dark it seems on the crust, but there is, I bake bread myself and there are varying differences in cooking from even 50 degrees, from 350 to 400 and it’s just like that, but it’s totally different on the inside. So that’s something that’s very hard to tell. It’s very interesting that you bring that up. But I think we have to just generalize if we were to think about that.

Participant 6: Yes, I think there would be a similar challenge in friends for example where it’s not typical to just keep one plate with everything on it, but you’ll have the entrée, then you have the main dish, then you have cheese, then you have dessert, then you have drinks. But in that case, again if you could somehow work in the ability for this app to take a picture of it, even if it’s different plates.

And then again because I think your focus is more on the big picture and the overall proportions. So if you could use that and then put that together in maybe one diagram, one pie chart with the relative proportion of different food groups, that would be very useful.

Participant 2: If you had a way also, if you could take a picture of their hand and then have like a ruler system or have the measure their width across their hands or width in height so that only they have to put their hands in front of the camera while they’re pointing the camera at a food so that their hand is near the food and then it automatically has measurements based on that. So you’d never need a [unintelligible 01:00:35].

Facilitator: Yeah. Just measure the portion, not the ingredients and the plate, right?

Participant 2: Well I’m thinking it would measure the, you know like length and width and possibly whatever else it can see. But of course there’s always the human error factors that you can work in with. OK they see something that’s not exactly right, they can just click and click something right and it changes. And the quicker they can do that the easier it’ll be for them.

Facilitator: Exactly yeah. So any other thoughts how to track other foods? Those other foods that are not in the guide?

Participant 1: I think the picture idea is a good idea and then having suggestions of is it this type of bread or is it that type of bread? You know they’re just picturing a bread so they can get more specific because it’s such a general thing, bread. Like it just looks like every other bread almost, you can’t really tell what’s inside it. Like a tomato you know it’s a tomato, a grape you know it’s a grape, but these other you know like bread is a little ambiguous with what’s going on inside there. So it’s good for people to be able to take those selections or input their own if they don’t see their selection to better specific their interests.

Facilitator: Yes great. So is there any other thoughts? Any new ideas?

Participant 3: I was just wondering what happens to the data privacy for such applications? For example, M had this thing taking a picture of the hand. In such a case that’s considered personal health data. Though it is really not, but then since it’s a [unintelligible 01:02:30] application it is considered that. So what really happens to all this data? All this images? And this is just food for thought. I don’t know how –

Facilitator: The idea of the application is to keep those information just for the users themselves unless there would be some option to sharing those with other people that they could choose and yeah they definitely would be just under users [unintelligible 01:03:04].

Facilitator 2: And I think you mentioned the app being a government app, but is it definitely linked to the government [unintelligible 01:03:12]?

Participant 3: Oh it’s not a government app.

Facilitator 2: This is like, especially right now it’s strictly for research it’s not like to Health Canada at the moment.

Participant 3: Oh that makes it even worse because then you know you will have a – Because then you will have Facebook login, Google login and they’ll take data on that list from the [unintelligible 01:03:31] so at least you can be a little assured if it was a government application.

Facilitator 2: Yeah definitely.

Participant 2: I think you can make it so that only, like say if you like [unintelligible 01:03:46] is one of the worst ones and that guide [we won’t say? 01:03:50] everyone says it’s alien, maybe they would only be able to see that you logged in or how long you were logged in for and maybe let that be a concern for people. Like there is options on lots of different sites and apps now to avoid submitting third party data and that’s exactly what that is.

And you can opt out of it in very many apps or whatever, websites. Just like people used to opt out of cookies which people still do and some people consider it a privacy issue and at the end of the day you’ve just got to consider OK, do you want to sell your info or do you not? Do you want to get bonus rewards for selling your info or having this which you can’t really do?

I think it’s actually against the law, but people choose to submit it because they think it’s worthwhile to get advertisements because they like seeing advertisements and that’s a thing. [Unintelligible 01:04:42] advertisements, they might go for it and then you might benefit from being able to provide that data, but if they don’t want it and they don’t want to see the advertisements, it’s just the perfect decision for them to not submit it.

Facilitator: Yeah sorry for the interests of the time, feel free to send all your thoughts or ideas for us, I’m sending them by email or even I could call you and tell them on the phone, but let’s keep to our, let’s stick to the questions that we have here, sorry about that, but I know that people have other stuff to do so I don’t want to bother them much. So the other question goes with so what, how do you suggest tracking beverages within the application? Do you think there should be a different classification for beverages like we have sugar sweetened beverages, we have juice, water, coffee, tea?

Participant 2: They should be your commonalities, there should be like your hot beverages, cold beverages, there should be the snack beverages like the sodas, energy drinks, power drinks. There should be like a health drinks section, there should be like casual eating. Like the stuff you, like when you go out like the milkshake or whatever. Like that other stuff, like it’s a possible cold beverage.

So you’d see cold beverages, you’d see [unintelligible 01:06:14]. Maybe stuffed with milk or stuffed with, that’s blended drinks. I think they should measure everything with like the level of the bottle because visually that’s good. Then people go with visuals, good to see that. If you say it’s a 500 millilitre bottle you dip it down on a chart that there’s half left right? You know you still have 250 millilitres.

And regardless of where it actually is on the bottle it’s still a pretty good estimate, like you’re going to finish the bottle eventually and then I mean sometimes you might not, but if you do it’s going to go through the whole 500 mil, you just run the level to the bottom and it’s done. You don’t need to have the exact shape of whatever.

Facilitator: Yeah. I see John was shaking his head.

Participant 7: Yeah my problem is I think you have to go back to M’s original suggestion where there has to be a list. People are worse than anything else at evaluating what’s in a drink. When it comes to juice parents think great for the child, you know give them the 24 ounces a day. People think I like kefir as a drink. If you buy the unsweetened one –

Participant 8: [Unintelligible 01:07:24] has no nutritional value, but it’s sweet.

Participant 7: Actually kefir is completely nutritional, but you buy the variant beside it with the fruit added and it’s worse than coca cola and it becomes very very difficult again even a comparison if you’re picking it up off the shelf, the different portion sizes that they’re basing their evaluation of the content of a container on. Is it a half a cup, the one you thought was better, they were measuring an eighth of a cup and those things are what really become difficult whereas if you give me a list of the start with the 20 or 30 top or biggest purchases.

Facilitator: So do you think that it should be classified in an other section other than the meal that you are consuming? Yeah great. Yes Gwen?

Participant 2: I think being able to add a beverage would be great.

Facilitator: Good idea.

Participant 6: My suggestion would be since the Canada food guide says to mainly stick to water, maybe there should just be an option where you track your plate and relative proportions. And then if you just have one drink or one cup of juice, I don’t think that would have such a big impact in the long run. So maybe only have the option to say oh today I had, I don’t know, a lot of alcoholic beverages for example.

Or today I had a lot of sweetened drinks or some sort of option like that. Or today I had a lot of dairy just when maybe it’s over one or two cups. Otherwise my question would be do you think the Canada food guide would recommend tracking things at this smaller scale? Or do you think one or two drinks are not necessary to track to still stick to these guidelines?

Facilitator: Yeah. I see that Mohammed is sharing something. Do you want to explain it a bit more?

Participant 9: Yes I really annoying when I think about what should I eat for the lunch or what should I eat for the dinner? I really love the app so that it recommends me for this night you have this option and pick one of these options. I can choose one of those options and the app can calculate the nutrition of my meals as well as the average.

Facilitator: So what if you just have some beverages? Some beverages that the app wouldn’t suggest to you? How do you want to track them in this application?

Participant 9: In terms of nutrition?

Facilitator: Yeah just tracking the beverage that you’re having. How do you want to record it? Do you think it’s necessarily needed?

Participant 9: Maybe at the end of the night or at the end of the week it can collect the data from me or in some specific time. But if I want to enter the data right after the beverage of that I take it’s really annoying I think.

Participant 1: I think it’d be a good idea to have a big like broad white wall area of what exactly every person has ate and so they can, like so they can see exactly how much they’ve eaten in say a week and what all of it is so that maybe if they grab one of their items that, like a can of cola right? Say they buy a case of 24, say they keep going to grab one, all they’ve got to do is click on that one cola can and maybe add another on of like in the white wall section where it has all the other meals and this and that. But because they clicked the can they were able to put another on easily instead of having through a menu to try find beverages, cold drinks, sodas and then click can of regular cola.

Like that’s four steps compared to being on your white wall and just choosing what normally you’re eating and maybe having a pop up saying, hey, look at this section. We think you need a little more vegetables. We think you need a little more proteins or grains. Or we think you need a little bit more like fruits in your diet. We see the section that’s missing here or this section that could use improvement or could give you a better healthcare lifestyle or that could suits your living styles or living wants or your weight wants.

Facilitator: Yeah. So, yes John?

Participant 7: With beverages as well, if there was some way, most of us stick to one or two regular choices so rather than having to have a long list, if there was a simple white add your own preferred beverage. You know I like, someone mentioned oat milk, I like soy beverage, unsweetened soy beverage. Well that’s 80 – 100 calories per cup and if I’m having two of those a day it really does have a potential to change the nutritional picture for me for the day.

Facilitator: Interesting. Any other ideas? Navine? How are, any other ideas? Do you want to share?

Participant 6: Honestly so far everybody mentioned some of the thoughts that I’ve been thinking of. The picture thing, but stuff like I can’t, I don’t know what to say regarding this because most of the things that I thought about is already mentioned.

Facilitator: Yeah I see your point.

Participant 6: But if I [unintelligible 01:13:50] I think I would for sure send it to you via email or anything.

Facilitator: Yeah that would be amazing, thank you for that. I appreciate it. So, yes Howard?

Participant 8: Yeah my only thought would be, I’m listening closely, is the Canada Health food guide is really an idealized type of guide. In the real world people have anniversaries, birthdays, cakes, have fun and there’s no fun in it.

Facilitator: Definitely.

Participant 8: It’s sort of unidimensional. So I would look to more the real world and perhaps people can feel free to eat what they want and then have the sort of gold standard and see if they can’t just move themselves a bit in that direction if they so chose. But I’ll go back to the Canadian Diabetes Association where they found that they need to incorporate the real world in. So they do put in cakes, desserts, stuff like that, just ask people to be mindful of it and moderate in their amounts and not do it all the time. So that would be my only comment on this.

Facilitator: Yeah we definitely will.

Participant 2: I agree on that. One of my cousins, great cousin, he now owns a cannabis shop, but he’s diabetic and he actually got a wife who gave him a Go Fund Me for a pump that is automatic so now he doesn’t have to worry about it. But he used to, and this is why I’m bringing it up, he used to get really sick because he didn’t care about what he was eating and he would just go for the regular sugar coca-colas and then he’d just pass out because he was too sugar high. We would always be measuring him while he was sleeping [unintelligible 01:15:29] you know because he’d be getting out of himself.

But like I think there should be a little bit of a warning for people if they’re diabetic, say if they’re eating too much sugar, just to help them out you know. Like say you’ve had this many in the past, like this many sodas in the past two hours, you know maybe try and, maybe it’s a good time to check your levels you know, if they are diabetic. Let it be an option like do you have any conditions?

Maybe ask them at the start when they sign up and you know they go through the whole thing of what their goals and wants and how their body is. And then just accommodate that you know because it’s hard for them. Sometimes you don’t want to deal with it and if you can have something that can tell you that you might be at risk then they might realize they’re kind of going beyond the bar with what they’re trying to handle. Because some people try and handle it. They try and handle their problems and that becomes the norm for them.

Facilitator: Yeah exactly. So speaking about the beverages, I’m not sure if you’ve noticed the Canada’s food guide didn’t include milk in it. So how would you suggest tracking milk or in general, some dairy products? How do you suggest to be tracked?

Participant 1: I think by when you get them and then when you finish them, that’s probably the worst thing to say.

Facilitator: So you know there are so many different kinds of like low fat milk, low fat dairy, high fat dairy. Should they be different shaded or yeah? Yes, John?

Participant 2: I think definitely they should. I mean from 1 percent, 2 percent, 3 percent, 4 percent, skimmed milk to like anything of like creams, I don’t think creams is too much of a problem, but some people drink them, they use them. They take shots of it. So including those differences can help and I know people who will go from, who will strictly go from 1 percent and not drink 2 percent because they think it’s too fatty and it’s only a percentage, supposedly, away from it. But I mean it’s to each their own.

Participant 7: There’s got to be a way to differentiate because I don’t drink milk. On occasion yes, but not regularly. But I like yoghurt, I like cottage cheese, I like regular cheese, I like kefir and again, I’m careful in choosing each of those except regular cheese on the fat content. So there’s got to be a way to show that the dairy is being taken, but as well what fat level?

Participant 2: Yeah I think it’d be great to add that on like the white wall idea where they can see the differences in the milks that they’re having. Like so they say, oh maybe I’m drinking a lot of 2 percent, maybe I should drink more one percent and visually connect with them.

Facilitator: So it should, do you think milk could be replacing the protein product?

Participant 2: Oh there were studies done on that. You’ve got to check those out because they definitely saw there was, the proteins were just [unintelligible 01:18:45] into the people’s bodies differently in the short term, but over the long term it was basically the same result. They had to shed a few more pounds of fat with the milk because it got into the body quicker and so the body wasn’t able to push it out as quick and it got used to the milk quickly so it would be able to better efficiently process it into their body so there’d be more in their body left over from the same amount of time compared to other stuff.

Participant 7: As well it’s the Canada food guide and the majority of people in the world, if I understand correctly, are lactose intolerant. Northern Europeans are sort of the different batch out there that absorb and use lactose more easily and shouldn’t the Canada food guide be showing nutrition as opposed to marketing for the dairy industry?

Facilitator: They definitely should, yeah.

Participant 9: Yeah that’s why I think that’s probably why dairy is not in the Canadian food guide.

Facilitator: Actually there was just a small bowl of yoghurt under proteins, but not anymore.

Participant 1: I always used to complain if there wasn’t milk on the thing at school. It was just like the pyramid guide which used to be what it was. I remember that. It was weird yoghurt though.

Facilitator: OK so let’s get back to the Canada’s food guide. There was some other elements on the Canada’s food guide, like on the backside of the guide that like being mindful of your eating, cook more often. Do you think we should put those options in the, they should be included in the application as well?

Participant 2: Of course. I mean if you include the options of the Canada food guide then people will think that you’re helping them to adhere to it. You know if you’re just listing it off then it’s also, it can work in your favor that if you’re not doing what the food guide is encouraging, then they can at least have the info from the food guide to help guide the decisions in the way that works with the app and their own lifestyle.

Facilitator: So do you think they’re necessary when people are more, are trying to eat more healthy?

Participant 2: I don’t’ think as in necessary, but like there’s video games that will give you a tool tip whenever something’s loading right? It’ll change so it won’t bet the same thing each time in a row. Like one time it’ll say you know, unsheathe your sword for quicker for better attack. And the next time you load up they’ll say you can jump higher by holding down the button or whatever. Right? You could have those things on like a loading screen and it’s just something to enhance their experience.

Facilitator: Some tips only? So you mean some tips would just pop up on the application?

Participant 2: Yeah exactly what’s said on the guide there. Those seven items, seven parts of words and then just put them in and then just have them somewhere on like the bottom of the app or somewhere in the middle if you want people to really see it and if you want to really encourage it and get in depth with it. I mean you’ll have people making suggestions here and there if you put something mid screen, but you know people like to [unintelligible 01:22:07] something around the side of the screen.

It takes their time away from the loading which is doing nothing and puts it towards something constructive like the reading, their brain’s being active. So not only is their body starting to be active, but their brain’s getting into the groove which is what you app is already encouraging so it might help people to acclimate to the energy usage that they’re going to have to use for the app. Something to build them up to using the app, that’s the point of those things.

Facilitator: Right. So any other ideas how to include those recommendations in the app?

Participant 9: Like there’s a concept called gamification. So they can use that because these are not, unlike the front page, the second page is more relative. If you used to do that you do it whereas the first one is the guideline, second one is not a guideline right? It’s just a general recommendation, if you can you’ll do it right? You can’t just force people, somebody to eat with others. Right? so if you gamify the application and then say like you get one point for say not eating with others. Five points for reading your food labels each time, something like that. It’s much easier and better and still people follow that right?

Facilitator: It should be something like a competition?

Participant 1: No it’s like your own personal rating.

Participant 9: You see a lot of health based application they use rewards because there should be some motivation for people to use the application right? So for example Strava, there is an application. It records your activity right? The same goes with Under Armor’s application as well. So all these they use points, they use goals, all that. So this application doesn’t seem to have any of those so gamification elements if you can bring it into either reward points, something of that sort. It’ll be more motivating for people.

Participant 2: But are we talking about like them to meet their goals that they’re setting out if they can set goals or are you talking about like everybody has a ranking in the thing and you can go be super active or not as active and then put in comparison to each other you’re either complimenting one person or seemingly degrading another person. You know, like I don’t think it’s a great idea for, it’s great for [unintelligible 01:24:46].

Participant 9: No. Gamification doesn’t necessarily mean you compete with the entire Canada right?

Participant 2: No I get it.

Participant 9: I’m just going to compete with myself, but to achieve the goals that Health Canada has set. And it’s not a game against, picking one against the other. It is to achieve the goals that Health Canada has prescribed to achieve that. So if yo achieve that goals it’s just that. So if you have achieved, set a goal –

Participant 2: That’s great, but how do you quantify exactly how well they’re meeting their own goals and Canada’s goals at the same time and how do you say to each person is this enough or is that good or is it great or is it like better? Or is it just a point value that is just an arbitrary thing. Like no one knows how good or great the amount of value a point [unintelligible 01:25:34] it’s just a number to say how many things you’ve done for yourself.

I think that directing it as a concept for self improvement and noting how well they’re doing on the scale of the app itself, depending on what the app values these items at then that is a little better. Like you could have a 400 pound guy lose 100 pounds easily by proper weight and muscle actions, but is that going to be more impressive, like say he set a goal for 200 right?

He doesn’t do so great a goal, but he [is? 01:26:10] impressive value compared to a woman who does, sets a goal of there being ten pounds and she loses 40 pounds and is now in like her perfect weight set that she wants. And it’s also Canada’s like perfect mid-weight average person’s normal weight then that’s what she wanted to get. But is that going to be worth more points than the male who say has not reached his goals?

Participant 7: If, as Mohammed suggested, you are able to share with family or friends or whatever group you’re establishing, then it’s got to work as well as something like weight watchers where people were getting together every week to be able to talk and they weren’t shaming each other, they were simply saying, OK here’s the goals that I’m setting out for this week or next week or this is what I want to achieve over the next six months and it was very much like Marian said, something like gamification because in fact you’re simply setting certain goals, whatever the goals are and the size of the group that you’re setting up yourself and inviting people to share with you.

Facilitator: Yes, Gwen wanted to share something I think. Yes Gwen, go ahead.

Participant 6: Yes. I think it might be helpful to go back to the big picture. I think the reason why Canada made this food guide is so that people are doing well in terms of health, in terms of happiness and that’s very subjective, that can vary from person to person. And I think to really stick to that goal it might be helpful to, within your app, have maybe a questionnaire regularly asking oh you ate this and that proportions of food, how do you feel? How is your sleep? How is your relationship with food? Do you feel like you’re eating well? Do you feel like you’re eating enough? How is your energy level?

Is there anything wrong and then you can maybe even, I don’t know how it will work in times of privacy but add, in the long term, if people are going to use this app for years, do they have any medical conditions that changed over time or did anything else get better?

Facilitator: Great idea. Great. Yeah I love those ideas. Anything else? So speaking about gamifications and I know that it was mentioned some otherwise just rewarding that some incentives maybe. What other ways, what other features do you think should be in the app that help people keep tracking? [Unintelligible 01:28:57] using the app. What features do you think would help?

Participant 2: Recipe sharing. But moderated. Like if someone’s put in like a bad recipe, like a recipe for something from [unintelligible 01:29:13] cook book, then you know people can report it and they’ll be sent a review and you know maybe this [unintelligible 01:29:21] thankful, but you know you just can’t have certain things and I think you need a report feature, if you’re going to have a community interaction feature so that the community can self moderate in a way and assist with your job because you can’t do everything, no one company can catch what the community is disliking on their own. They need the community to dislike it and tell them they’re disliking it. And then the response has to happen from the company and that’s how that works.

Facilitator: Exactly. So other than share supports? Yeah recipe sharing [unintelligible 01:29:48].

Participant 4: I wonder if you follow just like any other social media application. If you can follow somebody who’s been, you know consciously been doing well and replicate that nutrition, what are they doing. Instead of you coming up with something you want [together? 01:30:16] That will be much easier right? Just giving an example.

Participant 1: If there is a way to automatically upload like say a photo of a good meal and upload it to app and then the app automatically uploads it to your social medias that you’re connected with if you want to connect them. Say for Instagram it’ll pop up, put up Instagram and say like the short, detailed description of I ate this or whatever, whatever you’d want to put in right?

And then it’ll say, this was taken on this app. So it’s not only promoting community, but it’s also promoting the app and having more people join and make it a greater community. And maybe say it has these nutritional values in it of what you can ascertain from the company. So it helps people that don’t have the app know what exactly is in the food. Maybe that might bring more people in, might help people to be more health conscious. I think some people look forward to that with the more era where people want to be more healthy and get outside more and look into different alternatives where they’re also cooking at home and kind of lacking in community.

Facilitator: Yeah. Any other thoughts on how to keep people be adhered to in using the app? Keep tracking? Any new ideas?

Participant 6: Regular notifications always work for all type of apps.

Participant 2: Yeah give me push notifications. I love those.

Facilitator: Do you think there should be motivating notifications like positive quotes?

Participant 2: Oh absolutely. I’m always depressed, give me a positive notification, I’d chipper right up. I’d go hey, this thing’s not so bad. It’s actually making me feel alright, I feel mentally stable, mentally sound and then I feel mentally ready and that’s a big thing because if you don’t feel ready for nothing, it doesn’t matter if it’s good or bad, you won’t do it. You won’t get in touch with it.

Like sometimes I’ve got to check my bank and you know I just don’t want to, but as soon as the notification pops up like hey, you should check your bank. It’s been this time. I’ll go OK well they haven’t said anything bad so I can’t take it as anything bad, I might as well.

Participant 6: Sorry, last suggestion. There’s another app for example, it’s called Head Space it has like meditations on them and I know in their notifications they’ll put really positive messages or tips for the day so maybe you could do the same thing around for example. Oh having a fruit as a snack can help raise your energy if you’re feeling a bit tired and then arrange it so that people get their notification around 4pm when people would start feeling hungry before dinner.

Facilitator: Yeah good idea. So when M was talking about sharing recipes, what other instructions or support can you think of that should be in the application?

Participant 2: Seasonal things. News on what’s new in certain areas of the world. Like you’re going to have people that are using all over the world. So maybe have some specialized operators that can put up a news article about what’s being available in those areas of the world and perhaps bring together the different areas of the world by saying, by bring up a big article saying this one’s available in this area of the country right now, they’re experiencing this way.

Like Japan will have a cherry blossom festival and they’ll do, they’ll add cherry blossoms into the meals. Other places will have, like German beer fest and they’ll say OK well they’re watching their weigh the way they can and they’re incorporating the local, not fast food, but regional dishes that go well with beer. You know so we’re trying to encourage maybe making sure you have your salad which there’s always plenty there. It’s great for sobering up. But there’s stuff like that where you kind of bring people together while incorporating a little bit of innovation for the education.

Participant 9: What if there was a template? Like an ideal food guide for each dietary concern? For example if you’re a pescatarian or you are a vegan, you are a carnivore? For example if you just that it’ll just give you an ideal one and then you can customize that.

Participant 2: That’s a great idea.

Facilitator: I love it, yeah. Any other ideas? What instructions or sport? Yes Navine?

Participant 6: I don’t know if this is related, but just something that popped up in my mind now. Maybe if I just think some good healthy restaurants or places that offering healthy food for students or other people who can’t afford like being in a restaurant or local groceries where you can find fresh veggies and foods, like less [unintelligible 01:35:28] and stuff and for other cuisines, maybe like offering some, I don’t know, spices kind of foods that you can take form other cuisines, the international ones and then you just like you put it as a sort of mind or information or whatever. And then people can like go and see if they can find this thing in their local or international shops. Like something to educate people about the international food and the spices that we have.

Participant 2: So kind of like how Pokémon Go handled places getting on the map, they were able to call in and set themselves up as a vendor which would then attract people. That’s a good idea for like local markets that can sell hot stuff. Likes there’s one girl in Asia who sells bugs in a market and does stuff on Instagram and she would put that sort of thing up. She’d call up and say you can get all your supplies here for all your proteins, new age proteins or something. You know you can eat bugs.
