# Supplementary material for: Perceptions of self-monitoring dietary intake according to a plate-based approach: A qualitative study
Source: PLoS One. 2023 Nov 28;18(11):e0294652. doi: 10.1371/journal.pone.0294652 (PMC10683993; doi:10.1371/journal.pone.0294652)
Supplement: S4 Appendix — (ZIP) [file pone.0294652.s004.zip › Anonymized GP Transcripts/iCANPlate-GP-focus-Group-4.docx]

**iCANPlate-GP-focus-Group-4**

[Start of recorded material]

Facilitator: So this is iCANPlate focus group with members of the general public on July 30^th^ at 11:30 am. So now we’re going to start the questions. You saw the Guide, Food Guide now, so in your idea what would make it hard or easy for you to follow the Guide? Everything that comes to your mind, is it even useful, can you follow that?

R1: Yes it looks to be very, like with me it looks to be very easy to follow. So far nothing is hard.

Facilitator: So do you eat in accordance go the Guide now?

R1: Not really but I’m going to start.

Facilitator: Oh so you liked it, right?

R2: I think the only things that would be difficult is the foods that you haven’t tried before you or that you may not like, so how you coordinate those with the better foods that you should be eating.

R3: I think what’s really different is that everyone has different size of plates at home so the Food Guide doesn’t talk about like the average size of plate or how big your plate can be or can’t be to accommodate the different portions. Like it doesn’t talk about portion sizes and so like the size of plate that can be standardized because if you have like a 16 inch plate and you use a quarter of a plate for chicken versus a 10 inch plate and you use a quarter portion for chicken you’re going to have different nutrient values on the chicken for example, you know.

Facilitator: Yeah so –

R4: I would say time because it takes a lot of time to prepare vegetables especially and fruits and to purchase them and to get them and to prepare them. Time is a big factor.

Facilitator: Yeah, anything else that would make it hard to follow?

R2: I think working with the homeless population or people with low income they may not all be able to purchase all the food that they can get or even get it from a food bank, so I guess socioeconomic factors are in place too.

R3: I would agree.

Facilitator 2: Yeah totally.

R4: Yeah and the availability of the food especially in winter when a lot of fruits and vegetables you’re quite limited unless you go frozen but then it’s cost. It depends where you are in the country too.

Facilitator: Totally. Yeah those are really, all of them are great perspectives; so any other ideas?

R3: I noticed the food guide didn’t include seasoning, like dressings or ketchup, mustard, salt, pepper, I don’t know, hummus and all that’s sort of stuff. So I was like – because we add a lot of sauce to stuff for flavour. We add like flavours and stuff to cooking; we don’t eat our cooked food raw a lot of people, so from that perspective it’s like to ask people to eat their food without any flavour is a little difficult.

R4: And also how it’s prepared because it depends if you’re cooking with oil or you’re grilling or you’re baking or you’re roasting.

Facilitator: Yeah even if they didn’t add how to follow the oil, it is really important I think, yeah, good point.

R4: You also don’t have fats in it.

Facilitator: Yeah exactly. Any other ideas come to your mind?

R3: They didn’t have like snack options for kids.

Facilitator: Ah yeah.

R3: Because kids – like sometimes kids are difficult to feed with their palate and they do eat things like cookies and crackers and all that sort of stuff so I think they have to be mindful of that that kids like will be eating unhealthy things sometimes, but like you have to accommodate them in order to accommodate a kids palate.

R4: Also the food guide doesn’t tell you which options are healthier such as cheeses, even vegetables your starchy as opposed to your non-starchy. It doesn’t mention that in the Food Guide.

Facilitator: Exactly, yeah. So R1 are you still a fan [laughs]?

R1: Yes I am, yeah.

Facilitator: Yeah that’s great. OK so let’s move to the next question. What techniques, for example if you wanted to make some changes to your diet how would you monitor yourselves to stick to those changes, like would you monitor yourselves, what techniques would you use?

R1: Like what techniques, sorry, like would you use for what sorry?

Facilitator: If you want to make some changes into your diet –

R1: Oh.

Facilitator: – who would you control yourself, yeah, for adhering to those changes?

R1: I guess eating less carbs, less, fats, less –

Facilitator: Like you would set some goals?

R1: Yes, absolutely.

R2: I think with –

Facilitator: How would you do that? Sorry, sorry R2. Do you set the goals in your mind or you would use applications or something like that?

R1: Like yeah I would make sure it’s in my mind first and then I would have it written down obviously, absolutely.

Facilitator: OK, yeah R2, sorry to interrupt you.

R2: That’s OK. I think with dieting as well my technique would be kind of everything that I put on my fork into my mouth, like I’ll subconsciously or consciously, you know, see the advantages or disadvantages to having that. I know it’s not every single bit but you can kind of, when you get your plate ready you can say OK well I have this, this and this and that’s part of the Food Guide.

Facilitator: Yeah so being mindful of what you’re eating, yeah. What other techniques? [Pause] like some people maybe go for a support group, they would just try to make dietary changes with their peers or their family members. Yeah have you tried them?

R3: I found it helpful to make like a public announcement to like a friend that I’m going to make some changes and then set like one smart goal that I’m working on, it’s like an acronym where you make a specific goal that you want to do. And by making like a public announcement to a friend and stuff it’s easier to maintain than just like – and then of course writing it down as well and like posting it on the fridge or on the wall and stuff.

Facilitator: OK, yeah. How about journalizing, have you ever tried them, like tracking what you eat?

R4: I –

Facilitator: Yeah R4, yeah go ahead.

R4: I’ve done that, I’ve written down everything I’ve eaten. I find it cumbersome.

Facilitator: You did it with a paper and pen?

R4: I’ve done it, yeah mainly with a paper and pen, and I found it really cumbersome. Although I found that I ate most – like a lot of the meals were the same each day, so like breakfast was the same so I wouldn’t have to record it, I’d just sort or draw a line through. But I found it really cumbersome especially I was counting calories, and I just found it got overwhelming after a while. It took so much time.

R2: I actually used an app called FitnessPal, MyFitnessPal and I found it – at first I thought it was going to be cumbersome but it was good because it was drop – like you’d just have to put a few letters and it will drop down everything that you can possibly want to put; so it was. And then you’re connected with like friends and people or family so people kind of pump you as well. And you also put your fitness, what you’re doing in it as well and then it tracks it and it was really a fun app.

Facilitator: Yeah anybody else has tried one of those applications?

R3: I did try fitness pal but the problem is I became very unbalanced with it so like I was underestimating on purpose my food intake with the energy that I was expending so it became very unhealthy, and so I had to like remove my app because the app was able to tell you whether you’re consuming too much or too little and I was using that on purpose to under‑consume food and over-expend energy and that became a little addictive. So it wasn’t great so I had to put that aside.

Facilitator: Yeah any other adverse effects or pros and cons you can think of? So both of you just tried MyFitnessPal right?

R2: Mm-hmm.

R1: Yip.

Facilitator: R1 did you try it as well?

R1: No I’ve never used an app before, no, yeah no.

Facilitator: So any other pros and cons for those, for the applications?

R1: [Pause] No.

Facilitator: OK. So do you know of any diet self-monitoring application that currently resembles, yeah that just, or misses the plate method, like the one that Canada’s Food Guide emphasised on? Do you know of any? Yeah OK, no, I think it’s a no. So let’s see hour first mock-up of the application, it’s not developed, it’s not completely developed yet so we’re just having it showing. Let me know how do you view the app working when you think of eating, when you think about your eating throughout the day? How do you see it working?

R3: [Pause] I don’t know where you’d put things like tea and coffee [pause] but the rest of the stuff like meals and like lunch and supper could be added pretty simply. I like the idea that you don’t have to specifically write like the type of food you were eating but you just sort of generally estimate how much of the plate you used. [Pause] But the liquid might have to expand a little bit on the variety of liquids available so like juices, tea and coffee. People do drink soda, diet soda, orange juice for breakfast and that sort of stuff.

Facilitator: Yeah we’re definitely going to talk about it after as well. Yeah we need more ideas, yeah, so any other ideas?

R4: It doesn’t – it’s just meals it doesn’t include snacks. It looks simple but I’m not sure if I can work it with the proportions and things. I mean –

Facilitator: How hard – what makes it hard for you, like how you would visual things?

R4: Yeah, yeah. I guess what you do is you put the food on your plate and then you look at your plate and then you sort of estimate I’ve got this much of protein, this much of vegetables. I image you mean vegetables and fruits. And it doesn’t give fat, that’s one category that’s missing, yeah.

R2: And I think also the whole plate thing is kind of geared for regular people, for people who have like health problems or whatever, like for myself I am diabetic so I eat six little meals a day instead of three big platefuls. Or I’ll eat different things at different times so I may not have a whole bunch of protein in one sitting, so I don’t know I would enter that.

Facilitator: Good idea. So – what about you R1 how do you – what do you think about this first mock-up, so you could just –

Facilitator 2: Yeah sorry R1 had an emergency, he told me over the chat so he had to leave, yeah.

Facilitator: Oh OK. So we’ll get back.

R4: What about things like if someone has desert, like [pause] things like that I don’t know where that would fit in unless that would go under your grains I think, you know.

Facilitator: Yeah totally.

R4: Sweets.

Facilitator: So yeah what other foods do you think that are not put in this Guide, it cannot be placed?

R2: I don’t know if it would include like all types of food because different ethnic foods may – like how are they going to get in there as well, like and there’s a lot of different fruits and vegetables, like you know, people coming and bringing their ideas over from like Africa or something. They have a lot of different foods I find and there’s Canadian food, so you’d have to think of that as well.

Facilitator: Totally.

R4: Also it doesn’t it doesn’t differentiate between the vegetables, so I mean you can have all potatoes or all, you know, all the root vegetables which are counted differently than lettuce and tomato and things like that.

Facilitator: Yeah totally, exactly. So you mentioned some desserts, fats, cultural foods, or even I know before R3 mentioned the seasonings, snacks.

R3: The other thing that I was thinking about if there’s a holiday even and if you expect that there’s going to be more consumption or there’s going to be more meals served and you’re grazing or snacking to celebrate a holiday. You don’t have one plate that you’re serving from and that you’re eating from, you’re having a little appetizer plate that you’re filling as the evening goes along. So how would – would one put in multiple small plates or how would one put that in like for wine for example? If someone was having a glass of wine at a birthday party and they were having cucumber sandwiches with, I don’t know, mini hamburger sliders or something, so you know like appetizers how do you put in an appetizer?

Facilitator: Oh do you have any suggestion how to put them in the application? Like those other meals that we just mentioned that are not in the Guide, how could they be tracked in the application?

R2: [Pause] I think just by expanding the three colours, so like not to make it that’s really confusing but like maybe add another colour for, you know, desserts and snacks or another colour for, you know whatever like cultural or events or whatever. I just think it’s kind of – with the three of them it’s a little bit – it’s not giving you the total picture of what you may be eating because you don’t know where to put stuff.

Facilitator: OK so the idea I think of the Guide that we’re going to follow is, yeah just those there categories. So for these, for your suggestions I think it maybe could be another category. For example on the plus that we had on the top bar of the app, we had a plus sign, maybe people could track those other meals in that plus, right. Do you think it’s a good idea to follow the plate’s method even with those, with other meals or it can just be put in the application?

R3: I –

R4: Oh go ahead.

R3: I think it might be too much to try to take control of if you’re putting too much in the application and like – I don’t know if you’re – if the average person were to say they eat dessert every day, I don’t know what the percentage of people that eat dessert every day. If it’s a large proportion then maybe add a section for dessert in the application. But because holidays are kind of, are not far and fair between, like they’re not that often, maybe have a section for that in the added section where you can add things like sodas, wine, cocktails, appetizers, mini sliders, I don’t know, hors d'oeuvre and stuff.

R4: I also think that it should indicate the size of the plate because as mentioned by R3 you could – I mean you can’t really tell how much you’re eating, is it a small plate or a large plate. So I think that in the app it should tell you the size of the plate.

Facilitator: Yeah that’s exactly what I was going to ask now, that how – what preferences would you use when you’re going to filling the plate portion, yeah the plate proportion what preferences would you use to measure the meal portion? Do you think that it should be a reference?

R4: I [audio gap 00:22:23]

Facilitator: Yeah R4 do you want to add on it?

R4: You’re asking –

Facilitator: For example – references, for example, you know, like the hand references.

R4: Yeah, oh right.

Facilitator: Maybe you could use those –

R2: OK.

Facilitator: – or even we could define some standard references like the cups or yeah tablespoons, something like that. Which one do you – what do you think about the portions?

R4: Um [pause] that’s something I have trouble with, like you know a lot of diets tell you to use a hand or – I have trouble visualizing that. It’s just a spatial thing that I have. Probably weight or cup size would be better. I mean that’s just for me but might not before everyone.

R2: And I think to even simplify it even more what popped in my head right away was maybe spoon size because the you could say I had –because when you’re serving yourself you can serve with a spoon and figure out how big it is and then, you know, say I just had two spoonful’s of broccoli or three spoonful’s of chicken.

R3: And I think there should be an ability to address the plate size, so like somehow – sometimes in some apps you can like enlarge the size of the application to make it larger than their indications of like 16 inch circle or 10 inch circle or something like that, or like Tupperware, like people are eating at their table at work and they’re eating out of a Tupperware container. So you can’t necessarily say I’m eating a quarter of my Tupperware, it’s all mixed together in like a Tupperware container, right. So there should be like an ability to adjust I think.

Facilitator: Any other ideas? Those are really great ideas for us.

R3: I mean scales and spoons and weights and stuff can be – and then like measuring cups and stuff can be very overwhelming for people. But some people find it helpful and some people don’t. I think you just have to make it as simple as possible.

Facilitator: Yeah that’s the idea, that’s exactly the idea of this application.

R4: Yeah.

R3: Because serving spoons are different sizes for different people. And unless someone’s using like a measuring cup for example to serve themselves like a quarter cup of cooked rice for example and measuring out their portions of cooked rice or quarter cup of chicken nuggets or something, that can be accurate measured. And they can definitely use a spoon and put the broccoli in a measuring cup and measure how much broccoli they have, so that’s a really great idea. But again not everyone has the same sizes I guess.

R4: Perhaps what you could do is you could make it so that it is basic or you can sort of somewhere – like if you want to do measuring you could press on a button where you could measure it. Like you can do it at different levels that the person could choose how much they want to do. They could just do it by the basic just pressing on the plate or, you know, like it so that it’s – people can standardize to what they want.

Facilitator: Yeah.

R4: You know like where you sort of have almost like an index and, you know, measure or – but you don’t have to use it if you don’t want to.

Facilitator: Yeah exactly, great. So I see R5 I think, if I’m not wrong and please correct me if I’m, yeah I’m wrong, is joining us. Hey can you hear us R5? [Pause] Hey how are you doing?

R5: Sorry there’s a garbage truck just passing by so I’m just keeping it on mute right now.

Facilitator: Oh thanks, no worries. So we’ve started our meeting at I think 11:30 but it’s fine, it’s totally fine. We’re really glad that you joined us. So we’re talking about an application. First we talked the Food Guide, the Canada’s Food Guide that has a plate method to visualize the – to track the diet. You can see it here that half of the plate that the Canada’s Food Guide suggests is filled with fruits and vegetables and a quarter of the plate is filled with wholegrain food like quinoa, wholegrain pasta or wholegrain noodles even. And the other quarter of the plate is filled with the protein for that like eggs, poultry, fish and lean meat, your tofu, yogurt, legumes, seeds and nuts, and these are the foods that are, that can be tracked in the Food Guide.

And at the back side of the Guide you can see some other advices, some [unintelligible 00:29:07] like being mindful of your eating, cook more often, enjoy your food, eat meals with others, use the food labels, and limit foods high in sodium and sugars and saturated fat, and be aware of marketing. So these are some qualitative advices on – from the Food Guide. And we are going to just quickly show you the application mock-up. So this is the first mock-up of the application that you can – that we have developed but we need some other perspectives on it to develop it further. And so at the top bar you can see the little fork and knife that shows you the meal that you’re consuming. And the little drop is water consumption that we’re going to talk about. And the plus is where you add other meals.

And the idea is to be tracking app based on the plates concept that the Canada’s Food Guide stressed on, you know, using half of the plate for fruits and vegetables and a quarter of wholegrain and the other quarter of a protein product. So do you see the colours at the bottom, and that you can zoom in or out, and they show you – the green colour shows you the fruits and vegetables, and the pink colour shows the protein product, and the orange colour shows the wholegrain product. So being all those there we were talking about some questions and some perspectives and we asked about some perspectives of other people here in this focus group about this first mock-up of the application. So now we are going to talk about beverages, that R3 had some ideas about them. How do you suggest them to be tracked, do you think there should be another category for beverages or they can be included in the, you know, like the meals?

R5: Yeah I think it should be separate especially since it could be something that’s actually dehydrating you even if you’re drinking it; yeah it should be a separate category.

R2: I noticed there was an icon saying water, maybe that can be expanded a little bit to like, you know, not everyone drinks water unfortunately so, you know, you can have a drop-down of, you know, if it’s juice or mil products or pop or, you know, tea or coffee or whatever.

Facilitator: Alcohol even I think.

R4: Yeah.

R2: Yeah and alcohol, yeah.

R3: I really like the colours that you guys chose for the veggie grains and protein, so that was like – that made a lot of logical sense like visually so that was really good. So that was really like easy to understand. And I think what is working really well is the visualization of the app is really simple, right. So if we – somehow we have to maintain the simplicity by not adding too much. On one of the apps that I use, it’s not necessarily for food but it’s for like [pause] just for fun to like set goals and stuff I have one app that has a little emoji that pops up every morning and it tells me to eat a balanced meal every day. And it’s a cute little emoji that jumps up and down and leaves me a message in the morning. So it the app could send like a positive encouraging message that they can to repeat in the morning or not or people can input their own message if they wanted to.

If someone wanted to have a goal like I need to drink an extra two glasses of water in the morning that could come up on the app every morning, or I need to eat more vegetables at my meals, and then you get like a silly little stick emoji that comes up, that might be kind of also encouraging.

Facilitator: So what about the beverages how do you think they should be tracked?

R4: I think that –

R3: That’s – go head.

R4: I think the beverages should be the way they are with the drop of water, but I also think that it should have a drop-down menu and then you can just put in, you know, whichever one it is that you’re drinking.

Facilitator: Yeah and how do you suggest measuring them, like cups are fine or other references?

R4: Cups or glasses, yeah.

R2: Yeah.

R4: Yeah I would say a cup or a glass. You could say like eight ounces or something, or maybe instead of cup or glass just put eight ounces, so you can put down two eight ounces or three eight ounces or half an, you know, like a half. So you can just, like right beside it you can just put that in, almost like you just put a tick. Something very simple, but I would leave it with just the water because water should be the primary thing you’re drinking and it’s sort of a way to remind you.

Facilitator: Yeah exactly and that’s what the Guide suggests, emphasized.

R4: Mm-hmm, exactly.

Facilitator: Isabell I see you’re nodding, are you – you agree with all this or you have any other ideas that you want to add?

R5: I do like the idea of having it in ounces because everyone has like different sized glasses at home, right. So if you put it buy the glass then you might not actually be recording how – the accuracy of how much you’re actually drinking.

Facilitator: OK. So talking about – while we are talking about beverages I think we need to talk about milk specifically. So how do you tracking milk because it was not in the Guide and it cannot be – yeah how do you suggest to track it? Should we be in the protein products or should it be in the drinks and beverages that you’re tracking or it should have another whole category?

R4: [Audio gap 00:36:05] It should probably be in the protein.

R2: Or maybe do a separate category for dairy. I know they took it out but I don’t understand why because there’s so much dairy that people eat and drink so.

R4: But it’s protein.

R2: Yeah it’s still protein.

Facilitator: So how about when it comes comparing the fats in the dairy products, how do you suggest to track them? Do you think they should be separated, for example high fat dairy and low-fat dairy or it’s no different?

R5: [Audio gap 00:36:52] I mean it kind of folds into but the fat and the protein categories now doesn’t it. If you’re drinking skim milk or whole milk [laughs] it’s a completely different fat content that you’re consuming. Maybe it has to fall in both.

R4: Or maybe again keep the screen very, very simple the way it is because it’s really good, but again like you could press on protein and it could have sort of almost high or low fat that you could tick off. I don’t know how you would do it but something like that. Like each one should be able to have a drop-down if you want and then you can sort of see sort of how much of each thing you’re eating; something like that because I think it’s important to record if it’s high-fat or low-fat.

R3: What it’s missing is it’s missing plant-based milk and plant-based proteins, so people that aren’t like – are eating soya-based meats and sausages and plat-based, oat-based, soya-based milk, almond-based milk and stuff like that.

Facilitator: Yeah but the plant-based proteins were in the Food Guide. They were – yeah and they had emphasised [unintelligible 00:38:33] plant-based proteins. So do you have any suggestions on what R4 mentioned, do you think there should be – when you’re going to track your proteins there should be something that pops up and asks about fats and how – would do you think about it?

R4: [Audio gap 00:38:56] I think it depends, like are you just trying to get an app that follows the Food Guide or do you want to go beyond that?

Facilitator: Yes we definitely what to do be –

R4: You know people eat healthy, because it’s very, very basic and it doesn’t give you much information and that’s why I think, you know, if you put protein it could come up high or low, you know, and it give some examples. But it’s almost like you just – or it comes up automatically or you press on a button and it comes up. Or the same thing like with your vegetables high starch and it could give some examples and you could just tick off, or low and you could just tick them off like you don’t even have to think about it. And that would sort of give you an idea of where you are sort of in proportions of high low foods, starchy foods, not starchy foods, that kind of thing.

Facilitator: Yeah great idea.

R3: Because it would almost be like as if it you could select the protein section and determine if you’re – if let’s say you had ground beef for supper whether you fried it in oil, whether you baked it, whether you fried it in margarine, whether you deep fried it and breaded it, or whatever, like just provided an option.

Facilitator: And how do think they should – for example if they had some for example some fried vegetables, how they would track the oil that they were using when frying, how do they [unintelligible 00:40:56] do that?

R2: I think in that situation it should be like by the spoonful and that calculated to how much that would be in fat-wise or calorie-wise.

Facilitator: So for example when you’re tracking fried vegetables the vegetables would jump into the plate and the oil would go to some other category, right?

R2: I think so, yeah.

R3: Yeah, so by the spoonful or by the capful of the bottle.

R4: You know I’m just thinking in this app, you keep it very simple but when you press on let’s say vegetables they’re all – you know like in a grocery store they show the pictures of all the vegetables, well what would happen if in the vegetables you just pressed on all the vegetables that were on your plate and they just showed up on the plate. And then with your up grains, you know, let’s say you have bread or you have noodles or you have whatever and then you just press on those things and then it pops up onto your plate; and the same thing with protein. And then it might even go further and you could get portions like the amount of each thing, you know. But yet you’re starting with this very plain – because a person could just keep it that way or they could go into more detail.

R5: I think maybe when you’re going into the different categories too as well as having the drop-down list with the visual pictures there might also – it might also be a good idea to be able to search it, right. You know it might be something obscure that doesn’t necessary show up in the top of the drop-down and then all of a sudden you’re having to scroll all the way to the bottom to find it. And then in regards to the fat I think it also depends on let’s say you’re frying a piece of chicken and you’re taking that piece of chicken outside of the pan and then putting it on your plate, or if it’s like a stir-fry where you can’t really take the vegetables out of the oil because the oil is now absorbed. So that I think needs to be considered as well.

Facilitator: Yeah great. OK so when we were talking about the Food Guide you saw that there were some other elements that the Guide has emphasised on the back side of the Guide like being mindful or your eating, cook more often or be away of marketing something like this. Do you think that it should be tracked on the application as well, for example tracking some moods and feelings?

R2: I think that’s kind of getting a little bit bigger picture than what you really want. Like R4 said, you know, it always should come down to what is the simplest thing because when you’re thinking of the general population you kind of have to make it very simple.

Facilitator: So for example how about some super-simple notifications when you want to track something, something would jump up like pictures to just track the moods. Do you think even it’s helpful if you track your moods?

R2: I don’t think so.

R3: I mean mood and food is very known to be related. I mean this is a lot of research on that. But people might not appreciate the link if they’re putting the effort to track their food they might not like make the cognitive link between I’m eating a sad comedy movie and so I’m eating this type of food to compensate for the sad comedy movie or whatever. So like it might not be beneficial but it might at the same time, but it might also take extra time so people might not – they might feel like oh my god this is something else I have to do. I’m not going to bother doing anymore because it’s asking me to track my mood while I’m having supper and it’s just like totally way too off, totally way too much extra time.

R4: I personally have never found the mood to be of any help in doing any diets or recording. I just never – I mean I guess I know, you know, if I’m down or I’m up. I don’t – personally I don’t find it helpful.

Facilitator: Interesting. Yes R5 did you have something to add? I saw you unmuted yourself so I thought you have –

R5: Oh no it’s just the garbage truck has passed me now.

Facilitator: Great to hear that [laughs] yeah great ideas. So how about cooking at home, should it be tracked?

Facilitator 2: [Audio gap 00:46:35] Yeah like for example you can track the times you amount of times you ate a meal outside like at a restaurant or at a friend’s house versus like you staying at home and then like cooking the meal yourself. Do you think it would be helpful to track, like tracking yourself cooking your own food?

R2: I don’t think so. I think that’s just more information that’s going to make everything too [pause] too much trouble, like you’re shoveling on more and more in the app and people are going to be like yeah I don’t know what to do. So I don’t see the difference, like it’s going to all come down to the food. And if you did eat out they usually always have the ingredients on all that stuff that you can look at or write down or whatever and maybe you can enter that, but I don’t understand why you would track if you’re eating at home or not. That’s just me.

R4: And I think it would be quite valuable to have it and I think that it could just pop up, you know, restaurant or home and you just tick off. You know it was just sort of one of – like you know if you have all your little vegetables popping up and then at the bottom it would say restaurant or home or something or the whole plate or something at the end. And I think it gives people an idea of how much they’re eating out. But that’s –

Facilitator: Great.

R5: I think it also depends on, you know, really far into the minutia and the detail you’re wanting to go, right, because if you’re cooking at home then you know how much salt you’re putting in, how much sugar you’re putting in and you’re looking at that. If you’re eating out you have no idea, right, unless you’re eating something out of a box which might have the nutritional info. So, you know, if you’re looking to go into that type of detail then yes I would say it’s important but if you’re just looking at OK is this balanced with the protein, the vegetables and the grains then no.

Facilitator: OK great. So do you think there should be any instructions or support in the application, like instructions like recipes or some support group?

R3: [Audio gap 00:49:13] I think the recipes would be good but they would have to culturally sensitive and not be geared towards one particular culture because Canada is multicultural. Support groups are a great idea but someone has to maintain it, right, so if the app is willing to set up a support group and people come then that would be great.

Facilitator: So how about like some portion size guides, should it be there?

R3: Like a titbit of fun facts sort of thing?

Facilitator: Like the portion sizes we just talked about, should it be like an instruction about it or guide about the portion sizes and how do – how would people measure their portions.

R2: [Pause] I think –

R3: Um –

R2: Oh sorry.

R3: Go ahead, go ahead

R2: No, no go ahead.

R3: I think people have to be ready to learn, so if you throw too much information at them when they’re not ready to learn they get really turned off, so if you have information it could be like an option where they’re wanting to look at it when they’re ready; but not make it like you have to look at this. Because if you just throw documents at people’s face and say “You have to read this, this is important for you” they’re going to be turned off. But if they’re – if they have information and they’re wanting to learn then they’re more receptive to the information. Thanks R2.

R2: I think – OK. I think again we’re making too much out of a simple app. Like I think there should be basic information like for instance a chart of measurements. That’s a really idea because you don’t know maybe what you’re putting in sometimes. But not to go on and on and on with support groups and everything like that, this is supposed to be an app for tracking food and not, you know, reading 12 pages of stuff about, you know, stuff that may not be – because then people – like I know myself, like if I see that I would probably be turned right off of the app right away.

R5: So I don’t know because I joined in late, sorry. I don’t know. So the [pause] interface of the app shows the plate, right, with the breakdown. Now – so is that the same every time you open it or are you able to get mini windows at the end of the seven days as to what your plates look like throughout the week, right, and just –

Facilitator: Yeah we definitely can.

R5: – yeah and just give you some form of oh OK so I had a little more protein on this day but I balanced it out, you know, but I balanced it out over the next five days because I had more grains and vegetables that way kind of thing.

Facilitator: Yeah you could say your progress even, right.

R4: You could just have an app at the top that says more information and then people can just go to that if they want more information. But I agree that it should be kept quit simple, you know, at least visually it should, and if people want more information then they can drop down and they can look at, you know if you they want to read more about something. But it’s not on the screen where they have to read through it.

Facilitator: So what do you think would help people to stick to using the app, there would be more engagement or they’d keep tracking with the app? What features in the app could help them?

R5: Like notifications that you’re on track, right, like a little sound or a star that says “Good job, you’re on track today” or “You’re on track this week”, right, or a reminder that says, you know, it’s now Thursday and you’ve only had so many vegetables this week, right, just to, you know, give you that oh OK sorry [laughs].

R4: Or you could just have like emoji’s at the bottom. It could be, you know, a happy face or a mid or a sad face or something just to give you an idea if you’re on or not.

R3: Little, like little prizes, different prizes that pop up and be like, just for tracking a meal during the week or something, you’d get like a little magic prize or something that pops up.

Facilitator: What prizes do you suggest?

R3: Uhm [pause] maybe it’s like an anime character that has like a thumbs up or you get like a bouncing ball that bounces around or just something –

Facilitator 2: So like in-app stickers I’m guessing then?

R3: Pardon?

Facilitator 2: Like in-app stickers or something or like in-app like [unintelligible 00:55:03] fees?

R3: Yeah.

Facilitator 2: Like because I know there are like some apps that have like the in-app trophy so “Oh you won this badge or this sticker”?

R3: Yeah, yeah, yeah, yeah.

Facilitator 2: Makes sense, yeah thank you.

Facilitator: [Pause] How about some like competitions or support groups or user feedback, would they be helpful to keep people tracking? [Audio gap 00:55:32] Like game notifications, do you think it’s helpful?

R5: Yeah exactly, so then if you sign up and you let your friend know that you’ve signed up and you both kind of make it a competition so you can keep each other on track. It works for some people, some people – you know it should be an option whether you want to or not. It shouldn’t be like an automatic thing.

R2: Maybe have like somewhere where you can click on friends or whatever, so then you can kind of – I know with FitnessPal you were able to kind of compete a little bit with how much exercise you were getting or the consumption of calories or whatever, and that made it fun. So yeah something like that might be, might work.

R5: Yeah it’s almost like oh I got like 2,000 steps today more than you did kind of thing.

R2: [Laughs].

Facilitator: Great. So what about R4, do you think it would be overwhelming in the application or –?

R4: For me it would be. I think – and then if I didn’t do so well according to –you know with someone – it depends how competitive you are. And for me it would be discouraging, you know, it would be something that’s discouraging. So I wouldn’t like something like that but that’s just me. You know it could be an option and, you know, you could sign up for it or not.

Facilitator: Yeah it could be an option.

R4: Yeah but again keep everything really simple because if you start having too many options then you’re going to go nuts.

Facilitator: Agreed.

R4: But then again when you set it up you could customize it to your own self which is really a good idea. So the first time maybe you have to read a little bit and, you know, but once you’ve set it then it’s OK. And you can change it if you want to.

Facilitator: Oh yeah.

R5: Yeah I like the idea of a very simple main interface with possible buttons at the bottom for, you know, it’s a basic interface and then you add this feature or add that feature or whatever, right, whether it’s the competition with somebody else or if you’re now adding your micronutrients and macronutrients to the tracking, you know, and like vitamin intake –

R4: Or even like the picture of the foods and stuff like that, like if you want that as part of it then that can be part of it. Or you can just have the very, very basic.

R5: Yeah.

Facilitator: Great, yes R5 did you want to –

R5: Yeah I was just thinking too, you know, it’s really dependent on how much detail you wanted to go into, but you know lots of different vegetables have lots of different vitamins and nutrients and things like that as well so you can say OK well you’ve have a lot of spinach this week maybe you want to balance it off with some carrots or something else, right.

Facilitator: So there should be some suggestions for people, right?

R5: Yeah I don’t see why that’s a bad thing. You know for me I know I’m very picky about my vegetables and my usual go-to is broccoli, you know. And so if somebody says “OK you’ve had enough broccoli this week you should try something else, these are your options” then, you know, it might push me to try something different.

Facilitator: Right [pause] OK so what do you think – what features in the application could help people improve their confidence to follow the – to keep tracking [pause] they would be confident to keep tracking?

R2: I think if they’re doing well I think the idea of emoji’s or something might kind of encourage them to do more. And then if they’re not doing well I would think then a specific type of emoji I guess would kind of help perk them up a bit, you know, to say “OK well you’re not doing this but you’re doing well with this” or whatever so.

R3: I like that idea.

R4: Mm-hmm.

Facilitator: Yeah what else do you think would, might help people to feel confident about tracking their diets?

R4: You might want to have it so that – well I liked your idea where you could put on the screen like a whole week, but you might want to have it where it tracks the days, like you know, this is day one or this is day 70 so that it sort of helps people, you know, stay on it.

R5: Yeah you could have an internal calendar so if I’m inputting it on this date it’s like this is my breakfast, this is my lunch, this is my dinner on this specific date.

Facilitator: Yeah and R3 was talking about holidays and events do you think – yeah like having an extra option for, you know, a holiday so that, you know, like notifications would be off during those days. Could that be helpful for keeping people confident?

R4: It could. You know you could have something that just pops up and says the holiday, something about it’s one day or it’s two days, you know, you can go back to your – like to try to get the person to go back to the regular – almost saying it’s OK to splurge. It’s OK to have a holiday, it’s OK to have a birthday and have extra; but then, you know, you go back. And if it’s just sort of something, you know, that sort of says you’re OK but, you know … –

R3: I sort of just had like a brainwave. Sometimes when people are not feeling so well they have to fast for blood tests and stuff, so on the app they might have like a period of time where they’re not eating and the artificial intelligence might go into like panic mode and be like oh no this person is not eating like what’s going on. So like somehow [audio gap 01:02:50] not that you have to record something like that because they’re fasting for whatever reason like for a blood test or something, but like make it OK if they don’t log meals or they skip a few days, like I guess or skip a few meals. Just make it like not [pause] that the AI system is not like going into panic mode.

Facilitator: Yeah, great.

R2: I think also as we’re doing today and in some sort of incentive. I don’t know whether the app or whatever would be able to afford this but like make it that you win little emoji’s or whatever and you have to collect a certain amount to get into a draw to win something like a $50 gift card or something. I know that usually always works for me.

Facilitator: What do you guys think, do you think that incentives are helpful for [pause] yeah people and keep them confident or yeah keep them tracking?

R5: I mean I think so. I mean otherwise loyalty programs wouldn’t exist, right, and people stick to something because they’re like OK well I’m getting so many points, I’m going to get a free flight, you know, that kind of thing. So yeah, you know, I’m keeping – I’m being very consistent with the way I’m logging, you know, I’ve now had, you know, 90 days of non-stop logging and I get, you know, three emoji’s which now applies to a $50 Starbucks card or something.

R4: I agree that these, you know, loyalty programs work but I think that rather than a monetary kind of incentive which is external I think it would be better if it sort of said, “You’ve recorded 60 days straight” and then sort of a cute emoji jumps up or something and says, “Good for you, keep on” or something like that rather than a monetary competitive kind of thing. And the other thing is I was thinking of R3 when she said, you know, people sort of skip a meal because they’re ill or whatever. It could – I don’t know if this can be done or not but is it possible if could somehow monitor so that if somebody – if it’s missing meals something could pop up on the screen that says, “I noticed you missed a meal, are you OK?” And then it says, “I hope you’re feeling better” or something. Or if a person has overeaten or whatever then it could say, “Have you had a celebration?” you know, “Congratulations” or “Great” or “I hope you enjoyed your holiday” something like that. And then, you know, you’d go back; but it’s – almost it becomes like a personal kind of interaction.

Facilitator: Great. So in terms of accessibility what features do you think are required to ensure accessibly for all adults, like for all users, people with impairments [pause] yeah what features do you think would help, like fonts or audio version or [pause] language?

R5: Well I mean obviously it should be multilingual, possibly have text to speech and vice versa, right, have the captions underneath [pause] yeah.

R3: Something that also is helpful is some apps are able to vibrate when you press certain buttons so I don’t know if it’s possible but if, for the sensation if you [pause] I don’t know where it would be applicable in this application in particular but if someone is visually impaired then they can have the phone vibrate when they open the app they would know that they’ve opened their app, and it would be like, yeah I’m opening this particular app.

Facilitator: Yeah interesting. I never thought about vibration, yeah very good.

Facilitator 2: That’s so great, thank you for that.

R5: Also be able to zoom into the – zoom the screen because some apps don’t let you zoom into the screen so [unintelligible 01:08:06] can’t [unintelligible 01:08:08].

Facilitator: Yeah exactly.

R3: And I guess it would have to be Android and iPhone [pause] what do you call it?

R2: Compatible?

R3: Compatible, thank you, thank you R2. So people can download it. Because it’s with the Food Guide ideally it would have to be low-income or free of charge but I guess as an app you’d want to make a little bit of money.

Facilitator: Actually this app will be free of charge –

Facilitator 2: Yeah.

R2: Oh wow.

Facilitator: But thank you for that.

R3: So that would be great, yeah. But what – I’m just brainstorming here at the top of my head here, if people can connect the app to their playlist while they’re connecting and logging food then they can like have their music going that they like and log their food and be like, yeah I like my playlist, I’m logging my food, this is entertaining. I don’t know.

Facilitator: Yeah interesting. [Pause] OK so it’s almost at the end of our discussion today so any other features you can think of that should be in the app and we haven’t talked about it yet?

R3: I guess if you speak to any organizations for people with disabilities and see what they find helpful for the application [pause] so people that are visual impaired, hearing impaired, and see what they are finding helpful for applications to make them easier to use.

Facilitator: Unfortunately we didn’t have the – enough resources to support people with disabilities to be in this focus group but definitely that would be super-helpful for us. [Audio gap 01:10:33] OK any other suggestions for us, any other thoughts?

R4: And just I think it’s a good idea and I wish you luck with it.

R3: Good luck with it.

Facilitator: Yeah so the idea is developing the application based on your, the general perspective and we have some like for the experts that are registered dieticians and we have focus groups with them as well and we will gather perspectives on the application as well and we’ll use these ideas to develop the app. And we will launch the application in another study to pilot it and yeah it would be great if you are interested in participating in that study as well and we will get back to you with the application.

R3: Sure.

R5: Sorry if you are working with dieticians already then it might be a good idea to have a button or something in the app where it links you to, you know, Dieticians Organization Canada if you were looking to actually go further in your, you know, health journey, right, to find a dietician to work with etcetera.

Facilitator: Yeah interesting, exactly.

Facilitator 2: Yeah that would be a great resource to have for people who are struggling with meal planning, for sure. Yeah thank you for that.

[End of recorded material]
