# Supplementary material for: Perceptions of self-monitoring dietary intake according to a plate-based approach: A qualitative study
Source: PLoS One. 2023 Nov 28;18(11):e0294652. doi: 10.1371/journal.pone.0294652 (PMC10683993; doi:10.1371/journal.pone.0294652)
Supplement: S5 Appendix — (ZIP) [file pone.0294652.s005.zip › Anonymized RD Focus Groups/iCANPlate-RD-Focus-Group-7.docx]

**iCANPlate-RD-Focus-Group-7**

[Start of recorded material]

Facilitator: This is the RD focus group #7 on August 6 at 2:30. So, the first section will be more about the behavioural techniques and using the Canada’s Food Guide and plate guide in general. So, the first question will be do you suggest following the plate method as illustrated by the new Canada’s Food Guide to your clients?

Respondent 1: Yes.

Respondent 2: Yes.

Respondent 3: Yeah.

Facilitator: Alright. Across the board, I’m seeing some nods. Alright. So, what makes it easy or hard for your clients to use this plate method?

Respondent 1: [unintelligible 00:00:48] Some people don’t like that because they don’t want more specific, same as the old one. But it's a lot easier for people who don't have measuring cups and all that to go off of to just use the visual guidance.

Facilitator: Sorry, you cut out a little bit there. Is that just me or for everyone? OK, wasn't just me. Alright. Rebecca, you said that they liked the visual, but that it wasn't super precise. So, it could have been more difficult to use in that case?

Respondent 1: I mean, it's not as precise. So, it helps when people don't have mentioned cops and that type of thing to measure the specific portion sizes. I found some people get better from that sense. But at the same time, I've also found some people want one cup, two cups, half cup. So, then they're not sure if I have a really big plate, is that the same as the picture or do I have to [unintelligible 00:01:54] measure my plate to the picture. So, it has a little bit more ambiguity but there's pros and cons, I guess.

Respondent 2: For my clients, we have a lot of food insecurity. The visual sign on the plate, but they'll look at they'll be like, “I don't know what these are”, not the fruits of vegetables so much, but especially the grains, it’s like brown stuff and this stuff. So, I find maybe for some populations that doesn't feel like relatable visually but the spacing is it easy, if that makes sense.

Respondent 3: Yeah, I agree with all of that. When it was first released, we noticed that it's hard for some clients to choose what to eat. And I remember there was some comments about how it doesn't go with all the backgrounds or nationalities, it's not for everyone.

So, I think it's a little bit hard for some clients or patients. Plus, the fact that some clients would prefer to tell them exactly the amount like someone mentioned. But overall, it's easy for others who are OK or who have a good nutritional background to just go with the quarters and the half or vegetables and fruit.

Respondent 4: Sorry. One other thought I always have. Anything that's even remotely like a prescriptive template for how a meal should look, I always get a little bit nervous and these are just my thoughts around like just the Canada’s Food guide, in general.

We all say we love pasta and pizza and bread and say someone who just wants a big bowl of pasta for dinner, just making sure that person doesn't feel then a lot of guilt around not making half their plate vegetables every once in a while. That isn't what they need that day.

And I guess just with the app, strike that balance and ensuring that people are still having a healthy relationship with food and every meal doesn't have to look exactly the same. And sometimes you might eat more vegetables at lunch and not as many at dinner and that’s all OK.

So, I tend to when I'm counselling people around meal planning and how to eat, it's more about going with like more of a mindful eating approach, which they do incorporate and I enjoy that as opposed to this is how your plate should look at every meal.

Facilitator: Yeah, definitely keep those thoughts in mind for the rest of the focus group.

Respondent 4: Oh, sorry.

Facilitator: No, no, it's great that you brought it up now. Keep those in mind for later.

Respondent 4: Yeah, I was just thinking with the first question because I honestly don't use it much because for those reasons.

Facilitator: Alright, so the next one will be about which diet tracking methods or applications do you currently use with your clients or patients?

Respondent 5: Well, as you know, SLS there, I use the quinoa application. So, it's very visual and we can work – yeah, really just with the picture, we can do a lot. So yeah, quinoa application.

Respondent 3: For me, when I was doing some internship with a private practice dietician, she used to use MyFitnessPal and give one day menu or sample meal plan to a client. And I found it helpful when it comes to choosing like amounts. Some people prefer cups or grams, like chicken breast or how much yogurt or vegetable. So, that's what we were using and it was easy to just enter or choose the options on the website or even the application.

Respondent 6: Yeah, same. I have a variety I tell people use whatever method works for them. And I have some people that like to write it out in a journal or on a piece of paper. I have some people that use MyFitnessPal. There's another app, I can't remember the name I have clients use, but you take pictures of all your meals just to show like a rough idea of what they had. So yeah, I have some people that use a checklist. So, we made a checklist of aiming for protein, veggies, fruits, your starches, your fluids, and like just kind of checking off boxes throughout the day. So yeah, we use a big variety of different methods, because different methods work for different people in terms of tracking.

Respondent 4: Yeah, I would second that. I have clients who prefer to write things down in a journal. I also use a platform called Practice Better. And within there, there's like a food journal that some clients like to use, and then they can easily send it to me. And then, as you mentioned, I also have like a checklist for whatever their specific goals are.

If that's like eating a certain number of fruits and veggies each day, then they can check them off. And then some people really like to get more specific about tracking like calories and everything. So, they have tried MyFitnessPal, so yeah, kind of like lots of different methods depending on the client and what works for them.

Respondent 5: I will say too, sometimes with our food journals, depending on the client's goals, I also get people to track say they're working on gut health, I get them to track like digestive symptoms and stress and feelings. Same with like any emotional eating or if they're struggling with binge eating or anything like that.

I also get them with their food tracker to also track their feelings and emotions or circumstances around food sometimes just to identify if there's anything that they feel needs to be addressed, especially with like gut health and stuff like that. So, I find that can be helpful too in terms of food trackers, not just the food itself, but also if they have extra space for circumstantial things or symptoms.

Respondent 4: Yes, 100% and also like hunger and fullness just like after meals.

Respondent 5: Yeah, absolutely.

Facilitator: Anyone who hasn't spoken yet, do you use any tracking methods in practice at the moment?

Respondent 7: I've used for IBS or irritable bowel, same thing, Kara, the tracking app for symptoms and food and that one as well.

Respondent 5: I don't use many, more like checklists and also like tracking symptoms and just kind of using pen and paper to be honest. Sometimes, I just worry a little bit when it's like specific numbers involved because I do like to focus more on the intuitive eating piece and focused on that hunger fullness versus exactly how many calories or a meal or whatever it is. So, I usually try to steer away a little bit from more of the like very specific ones that track numbers and things like that.

Facilitator: So, building a little bit on that. You mentioned that you individualize the tracking methods based on your clients a lot. But what makes it easy or difficult for your clients to use the tracking methods that you've mentioned?

Respondent 5: Some feedback I've gotten from people who have used MyFitnessPal and stuff in the past is they like when things are scannable. So, if they have a protein bar or a yogurt cup, they can just scan the label and it'll input it for them. Yeah, I think the main thing that I get from people is it needs to be as easy and fast as possible.

So, whether it's a scanning thing, or that's why a lot of my clients like just taking a picture of their meal. Those kinds of things, I think, make it easy. I think the more you have to search through a database to try to find the food and match it up, that makes it a lot less likely that people are going to stick to tracking for a few days, if you're looking for a three to seven day food journal.

Respondent 3: Yeah, same for me. A lot of clients prefer to just take the picture instead of entering all the information about what they just ate. So yeah, I agree with that, it needs to be simple. The less they do, the more information we'll have, and the more pictures they will take.

Respondent 1: I also found when people had to – they were making recipes, and they had a hard time finding recipes, it really turned them off from tracking because then they weren't sure. For example, if it was a curry, if the curry that was on the tracking site is the same as the curry they made. It was more or less, if it was the same ingredients. So, I found that was a very big barrier. So, it sounds like the picture is a good idea.

Respondent 3: I find that it's might get hard for some people, especially with eating disorders also. They get really focused on the number of calories. And like someone mentioned, if they don't find an item, because especially with MyFitnessPal, sometimes it's not clear. And sometimes it's grams and not cups. So, they get so stuck with this. So, the easier the better and it's not always the case for everyone.

Respondent 6: For my clients, we have significant issues with technology access. So, many of my clients don't have a phone, or data on their phone, if they have any sort of device or if there's a cost associated with an app. Lots of literacy issues or language barriers. So, I would say for the vast majority of people, it's all paper based. We have a few clients who could sort of manage the technology. But for the vast majority, it's not a realistic option.

Respondent 5: For myself, I would say whenever I'm doing like a diet collection for – it’s research and focused on right now, but it's always just retrospective an FFQ or 24-hour recall or something. I guess the issues around the remembering exactly what you ate or trying to summarize how you normally eat, just that retrospective nature is challenging. So, anything that you can do kind of on the spot is probably going to be a bit more accurate but not feasible for our purposes.

Facilitator: Right. Building off of that question, which tools are you aware of either paper or technology based that currently resemble the new Canada's Food Guide and the plate method specifically?

Respondent 1: I can't think of any in particular, I can't think of any apps I guess. But I have seen things similar to what Emily and Clarissa were alluding to where it was on a page would have 10 apples, six carrots and every time you eat a fruit cross off an apple or every time you ate a carrot you cross off that. So, I guess in terms of picture or diagram, I've seen those but not so much like anything with the plate model per se and then that type of thing.

Respondent 5: No, I don't think I've seen any, either.

Facilitator: OK, good. That is what we are hoping to hear meaning that we're on the right track for this because it is a very clear departure from before and from where we were coming from with all the serving sizes, and all those things on the last Canada’s Food Guide.

Showing the app’s prototype

Facilitator: OK, so now anyone have any comments just based off of this brief video?

Respondent 2: Sorry. Did you say you could change the proportions?

Facilitator: Yeah.

Respondent 2: Yeah? OK.

Facilitator: Yes, you can change the proportions.

Respondent 4: I think the protein and grain colours are too similar for me.

Respondent 6: Do they see like a percentage number or is it just visual?

Facilitator: At this point, it's just visual. But that's something that we could envision for sure.

Respondent 7: Kind of bringing back to what someone mentioned before, is there like a possible list that they can reference as well, like, say, they don't know where lentils are going to fit? Is there like a potential food list of like different proteins, different grains so that they know, “Oh, I ate this much quinoa. This is the category I'm going to put it in”? Is there like reference points for that?

Facilitator: Not yet, but that is something that we are envisioning, and we've been hearing would be useful.

Respondent 3: For me, I'm just wondering, how will the app analyse recipes where the ingredients and the food groups aren't separated?

Facilitator: Yeah, great question. That's the next question that we will be getting to as well. So, thanks for bringing that up. So, any other gut reactions to this quick mock up? Alright, so I'll stop sharing now. And the first question about this is how would you view this application working to record the meals throughout the day considering breakfast, lunch, supper, and snacks?

Respondent 1: So, for the current one that you have, are you able to input something and then save it and then input your next meal? Or are they going off of their whole day? They're going to input once because just based on what it showed it look like that's a one-time thing.

Facilitator: Based on this, it would be every meal and every snack. But that's something that we can discuss as well, if you think maybe a full day would be more representative, or more useful. That’s something we can discuss.

Respondent 4: For me, I would say definitely not sending notifications or alarms because that bothers clients. So, if they could have the possibility just to take the picture, when the time of the meal comes, I think it would be better than sending notifications.

Respondent 6: I just have a question. Because I know at the beginning, they showed that the size of the plate can be made bigger or smaller, and is that to reflect like the size of the meal? Or if it's smaller, would it that'd be indicate that it's a snack?

Facilitator: Yeah, that's our next question. So, with regards to the portion size of the meals, how could that be reflected within an app like this or within proportion based tracking?

Respondent 6: Yeah, I guess my response would be like, if the size of the plate reflected kind of the size of the meal, then maybe that's one strategy but there are challenges.

Facilitator: Yes, definitely. Alright, so I've been hearing two different options. So, we had every meal that could be tracked, or doing a summary of a full day. What are your thoughts on that?

Respondent 1: I think every meal, for me, that would be easier because by the end of the day, I don't remember what I've eaten, especially I tend to snack through the day. And if I'm not writing it down, good luck trying to get it back out of me, by the time I'm going to bed. So, after a meal or snack for myself personally.

Respondent 7: I think every meal or snack as well, because I think if you ask somebody to think about their day and like, what proportion was protein, what proportion was grain and veggies and fruit, they would have no idea how to figure that out. I think every meal is definitely easier.

Respondent 3: I would also say every meal, as it's easier for them to track compared to at the end of the day where like they might forget or just feel tired. So, every meal works better.

Respondent 6: I just had a question like, what if they were eating something that didn't necessarily fall in the food groupings? Like, what if they had chocolate or something? What did they do in that occasion?

Facilitator: So, I'm going to turn that question to you guys. What do you think they should do with the other foods that aren't represented on the Food Guide? What other foods can you think of? So, Emily mentioned chocolate. Which other foods would there be?

Respondent 1: Stuff like the dairy ones were on there. So, if I want a piece of pizza, not sure where I throw the cheese.

Respondent 6: Don’t they have that in the protein category?

Facilitator: Yeah, they do, but it is a very tricky one. So, that dairy piece for sure.

Respondent 1: I think at point I saw one where they had that separate glass of milk, and that was where the dairy was supposed to go. And people were like, “Well, what if I don't drink milk? Does that still count? It was a huge confusion with where to put those things.

Respondent 4: Other foods like oils.

Respondent 5: Yeah, I agree. I think maybe fats would be a nice piece to add. Maybe making it possible, like you have the three main categories but making it possible to add like they can customize their app to have what sections they want represented on their plates.

So, if they want to be able to incorporate fats, if they follow a lower carb diet, for example, and they want to represent that more in their plate for them, maybe having another colour for [unintelligible 00:22:34] pickling of other foods, but you don't want to give them a negative name, either. So, the foods that bring us some joy, usually. But yeah, maybe having more than just the three colours and being able to have like five or six based on the foods, two different food groups.

Facilitator: So, what would those groups be? You mentioned fats.

Respondent 5: Some kind of other, I don't really know what to name it, but things like a baked good or like you said chocolate. I don't know. The things that don't really happen.

Respondent 4: Maybe just the miscellaneous or like add on.

Respondent 1: Yeah. At what point does like a baked good fall under your other category versus like the carbohydrate. How do you make that call?

Facilitator: Yeah, super tricky question.

Respondent 4: Yeah, what is your meal is not on a plate?

Respondent 2: What about like soup or cereal?

Respondent 7: Or a smoothie, I don’t know. And I don't really see why has to zoom in and out because I guess what you're trying to say is if you have a big plate or a little plate, but really, it’s not the actual size.

Respondent 6: Yeah, it’s just the distribution. It’s not the size.

Respondent 7: Right. And the person wants you to think they're eating this much. So, they make it like a tiny circle. I don't know. It just seems weird.

Facilitator: Yeah, that's the feedback that we're looking for. For sure. So, how would you feel that it would be better represented? We mentioned the proportions, the just proportionality?

Respondent 7: Or having a little cup on the top of the plate and they can type in what the fluid is.

Respondent 5: I'm trying to think like say you had like a smoothie. Would you, on your plate, put like three quarters fruit and then say you put dairy in or something? Just envision it on a plate? I don't know. It's a bit confusing. So, I guess you just would want to make sure there's like maybe examples for them to follow of different types of foods that -

Respondent 1: - Yeah, they could change the shape to be like a cup. Check off that they did have like protein options with the milk or whatever it was and fruit, but it looks more like a cup. I don't know, or glass.

Facilitator: Mm-hmm. So maybe having different food vehicles. So, a glass, I heard a bowl, different sizes of plates.

Respondent 2: A square plate.

Facilitator: A square plate? OK.

Respondent 4: Measuring cups, I found really helped with visualizing big time, they really help and they are convenient and available everywhere. So, I find this really helped many clients. And this way they did start counting calories or really tracking.

Facilitator: That brings us kind of back to how would we represent portions on a plate method, or on a cup method or bowl method?

Respondent 6: Yeah, that’s hard.

Respondent 4: Maybe like more visual examples that we can refer to like a tennis ball.

Facilitator: Alright, so having visual representations of what -

Respondent 7: Yeah, I agree or I don't know, like your hand or something? Or like how many, I don't know, I feel like that gets used often as well. There's lots of examples.

Respondent 6: But if the Food Guide is no longer even recommending those portion sizes, then what's the point of bringing that into this tracking method?

Facilitator: Yeah, also a great point. We did do a paper version of the study prior to developing an app and people really found it difficult to know how much their plate to fill up if there wasn't that portion equivalent. So, that's why we're asking that question.

Respondent 7: The portion seems like it's more important for researcher, like the people who are like analysing the app to be able to spit out like a meaningful, as opposed to like it being helpful for the client. I don't know how much value it brings to the client. So, maybe it's not necessary. I don't know, I guess depends what it's being used for.

Facilitator: So, at this moment, it is for self-monitoring. So, for the members of the general public to use to monitor their own diet. There could be kind of a dietician behind it as well, but it wouldn't necessarily be an assessment tool at this point. That brings us back to the portion and whether or not it's important.

Respondent 7: Like you could leave it as it is and just do like their proportions of the meals, but maybe is there any way they could, I don't know, type in somewhere, like if you hovered over the green part and they could make a little note, like one and a half cups of salad if they're interested in measuring it, but not a requirement necessarily?

Make it an optional feature, because we are looking more at just the general proportions of everything versus precise measurements. And a lot of people need to get away from precise measurements anyway, because they get obsessed with it.

Respondent 4: Yeah, at the end of the day, the proportion is a guide, but they need to listen to their fullness level too. So, I'm not sure if it's that relevant.

Respondent 6: I guess you just don't want people to feel frustrated. They won't use it if I just can't figure out how to visualize that. But maybe if somebody had like an optional feature, we could just have the basic one and then maybe like 2.0 or something.

Facilitator: With the portions you meant? Yeah, OK. How would success or how would a successful day be defined in this context?

Respondent 7: Makes me nervous defining it as successful.

Respondent 3: I would say, a successful day would be applying the plates in a way as visualizing their meal. And at the same time, eating like what they really want or felt they want it without like restricting or labelling foods. And another thing would be listening to their hunger cues, like the unsatisfaction, along with applying the plate method.

Respondent 6: So, I just asked is the app planning on asking what goals might be for using it? Because if it's something like my goal is to increase my vegetable intake, then that could be measured at some point at the end of the day. But that would be if they have specific goals, like asked at the beginning when they're getting started.

Respondent 5: I was going to say progression like trajectory. So, I'm someone who eats no fruits and vegetables. And now, it’s on the plate. That's a success.

Respondent 1: And I'm thinking too, kind of depends on, is their goal just to start tracking their intake? Maybe it's successful if they even use the app every day, and that could be enough for success for somebody. So, I guess kind of like Brianne said, Is there a part where you can set your goals so that you can get that positive reinforcement, but at the same time, you don't want to shame anyone, if they haven't quite gotten to their goal because it can be difficult.

Facilitator: So yes, goal setting is a huge part of self-monitoring. So, which goals would you think would be interesting to incorporate in an app such as this one?

Respondent 5: I think more vegetables than protein foods and grain foods in terms of proportions. So, that would be one.

Respondent 3: Going colourful with fruits and vegetables, like trying variety, not just greens or just orange like purple, red.

Respondent 6: Like getting fluids in, like getting staying hydrated would be easy one to incorporate.

Respondent 7: I was going to ask too, if somebody is putting in kind of like what Rebecca said, maybe the success is just at the end of the day, you say they complete and press this button, and it gives you a checkmark so that people get that satisfaction.

But I wonder is there a way too, if possible, if people make all these plates for all their meals and their snacks, could the app average out see, so they could see what their day looks like in a plate?

Does that make sense? So, maybe they did have lunch. That's all passed on and that's fine. And then they had supper, which was a steak and Caesar salad. And then the app can figure out their proportions of the different food groups based on their different meals through the day instead of every meal being looked at individually.

Respondent 4: Yeah, summary of the day, of the week, of the month, maybe. So, to track for progress more than success.

Respondent 7: Yeah, I like that.

Facilitator: So, how could be app kind of summarize, if, in the context of what we've been saying. So, noting every single meal on a plate? How would it be able to put all those meals together?

Respondent 5: Well, I think Clarissa kind of mentioned this, if it could somehow add them up or average it out. So, that you can see at the end of the day, here's the proportion of each different food grouping of everything you ate.

Respondent 6: Because essentially, you're taking a bunch of pie charts. So, there has to be a way to average out three pie charts if they ate breakfast, lunch supper that day.

Facilitator: Would the portions that matter in that case, if breakfast is this big and supper is this big?

Respondent 7: I don't think so because I don't think the app is meant to get into specifics or portions and calories and all that, but just like the balance of their meals, I guess, more than anything.

Respondent 2: Because what you're saying is this is someone, like they're not working with the dietician using this necessarily. So, you don't have that professional or somebody coming into evaluated in a way that we would, but it needs to be easy for them to understand the results.

Facilitator: Definitely.

Respondent 1: I guess, in that sense, it might be fine without the portion size, because even after you make this app, there will still be MyFitnessPal and all those other ones where you can get more specific if you want to. But it is nice to have an option that is slightly more general and doesn't throw numbers so harshly in your face. So, it might be OK to just leave as is with the visual representation.

Facilitator: Yeah, and bring together kind of the proportions at the end of the day, so they could look back on it and maybe month and year, if they get there.

Respondent 1: I wonder there could be a mindful eating like peace, where you're asking someone how did you feel about how you did today? Was this a successful day for you? I don't know, were you at peace with your eating? I don't know how that can be worded, but just maybe success for somebody means they've gone a week without obsessing over their food. Or if they’ve never thought about it before, they've gone a week thinking about it. But I don't know, whatever that looks like for somebody.

Facilitator: Yeah, so that brings us to our next question is which other elements of the food guide, so that backside of the food guide, would be interesting to include in an app such as this one?

Respondent 4: I would say to eat less processed foods. So more insisting on that, so that the protein they eat is less processed. Is it a meal that you cooked? Is it a meal from the restaurant? So that would be another one. But I don't know if you had a question there about the other one.

Facilitator: If anyone has anything else to add about that backside. I can share it again if you want a little bit of a refresher as well.

Respondent 4: Yeah, so the first one was to maybe give more examples of less processed foods. For example, the protein. And the second one was to cook more than to eat out.

Facilitator: I’ll share the second slide.

Respondent 2: And one of them is about eating with others. Like there could be a checklist, like did you make this meal? Or did somebody make it? Is it homemade? Did you enjoy it?

Respondent 3: Limit, I was going to say high sugar content like simple sugars are added sugars. Focus more on complex carbs kind of thing.

Respondent 7: I like enjoyment and satisfaction and mindfulness piece as well. I guess you could also, like people were working with someone explain it in terms of like, “Did you feel hungry? Did you feel full after? Were you satisfied with your meal? Or did you need some little extra?” and starting to like pay attention to their body and their cues a little bit more.

Respondent 5: That’s true. And then it could summarize like, “Hey, did you realize every time you ate this, this and this or every time your plate looks balanced that you…?

Respondent 7: - You felt like this.

Respondent 5: Yeah.

Facilitator: So, what elements would be interesting to track for mindfulness for the enjoyment piece?

Respondent 7: I think hunger cues before they eat and then after and just like in a very non-judgmental way of maybe they ate until they felt really full and it's like, what did your plate look like? Or maybe they were starving before they were eating? And then what did their plate look like?

Respondent 5: Yeah, I agree.

Facilitator: I'll stop sharing now. We just got a little bit of a refresher. Are there any other elements of this backside of the guide that you think would be interesting to include?

Respondent 1: Maybe on the mindfulness piece like where were they eating? What were they doing while they were eating? So, if there was a little piece of environment, mealtime environment, who were they with that sort of thing.

Respondent 7: Yeah, I agree like, “Did you feel distracted during this meal?”

Respondent 6: Or even the timing, I don't know, if you are putting it into your plate. Breakfast, you could put like, 9am and then snack at 11 or whatever. And maybe it could comment on, this is probably getting outside of food guide, but if someone's getting a good distribution of food throughout the day, or they're just eating once. And yeah, the plate looks good, but they just ate one time.

Facilitator: I'm hearing a bit of an analysis piece that the app would bring as well. So, what do you think would be interesting for the app to analyse based on the data it's receiving?

Respondent 3: I would say, nutrients for example, mainly nutrients, percentage of carbs and proteins, especially if someone is looking to increase their protein intake.

Respondent 2: I don't know if they could do that without portion sizes very well. And I think it kind of gets away from the main purpose, and potentially delves into some, I don't know, encouraging like obsessive/dieting behaviours.

Facilitator: What would be interesting to track?

Respondent 1: Maybe again, depending on what the person's goals are. So, for example, if there are the functions for mindful eating that everyone mentioned, that goes with mindful eating. If they see that their goal is to eat without looking at my phone, then it can provide the data on how many meals you have without your phone, that type of thing.

So, I guess it depends on whether or not the app would have the goals function. And then going off of that for what that person might like to see tracked. Because, for example, me, if I never hear from the TV, it's not going to do me any good to hear that I didn't eat in front of the TV. I know that and I didn't ever do anyways. So, just more along to kind of pick what they want analysed based on what criteria you have available in the app.

Facilitator: So, building off of that, what would be interesting to have available. So, I've heard that mindfulness piece, the where, when.

Respondent 7: Mood.

Respondent 6: Maybe sleep. I don't know if that's getting outside of the realm of what the app is supposed to do. I think one would be someone already said, less processed food. So, the option of homemade versus not.

Respondent 2: It's like, you could almost have like a little checklist with every snack and meal and be like, “Was this a homemade meal? Do you feel like you were distracted during this meal? Were you satisfied with your meal?” And then based on whatever they check off at the end of the day, could average it out over the week. And then over weeks, they could see their progress like, “Oh, you know what, I did cook more at home over the month. Or I did step away from the TV and ate with my family” based on just checking some boxes.

Facilitator: So, coming back to that simplicity piece, which would be the key goals that people would be able to track or would it be that personalization again?

Respondent 6: If there was just a general goal feature like they could type in whatever their goal is, whether it's I want my plate to be a quarter protein no matter the size of it for every snack or meal and if they could, “Yes, I achieved this” or “No, I didn't” based on their end of day average, then track their progress that way as well.

Respondent 3: I would say one of the goals would be increasing, let's say vegetables and fruits, according to age groups, let's say, 10 a day or so. Like we used to focus when we used to talk about the food guide in the past. So, maybe that's one goal of increasing fruits and vegetables, getting to that number goal that they set.

Respondent 6: I think you’d probably have to have like suggested goals. Well, I think when people are starting like launching out into eating healthy, especially if they're doing it without an RD or any support, [unintelligible 00:46:08] people always know what they need. But the goal is looking at this new guide, and being like, OK, but maybe having the suggested take off.

Respondent 1: That's a good point, like when they're setting up their profile. Like, for example, I just downloaded like a new meditation app. And at the beginning, you set up your profile and be like, what are your goals, and it just has a list of pictures whether it's mindfulness or anxiety, or stress, or depression, or whatever.

So, they have your list and you pick out of their list. So, we could do the same thing based on maybe different nutrition goals like do you want to increase your protein? or increase your vegetables and fruit, or increase good fats, or do you want to be less distracted? And then we could give them the suggestions and then they can pick it.

Respondent 3: Speaking of ideas, I would suggest also, maybe in the future, when the app is like well maintained, having reached out to RDs like helping them set their goals or like for suggestions of the or if they have any questions, it would be nice to have that option. They can reach out to an RD whether it's a volunteer paid or that kind of thing, it would be a nice idea. So, they would get evidence based information or help rather just looking or searching online.

Respondent 4: That's interesting. And other examples of goals could be increasing the amount of plant based protein, whole grain.

Respondent 6: But would the plate know that it was – like you putting in that a quarter as grains, does it know what it is?

Facilitator: Not at this moment, but that could be something that we could add. So, having a little, maybe checkbox again, that would ask if it was a whole grain or not, plant based protein are not, processed or not, getting into those details as well.

Respondent 7: Processed or not, I feel like it would be tricky.

Facilitator: Yes, definitely.

Respondent 7: But yeah, the other ones sound more reasonable.

Facilitator: Alright, so I'll move on from this question. So, how would you suggest that beverages be tracked in the app? So, we already talked a little bit about that cup, how about things like water or sugar sweetened beverages, juices, coffee, tea?

Respondent 3: I feel like this could be tracked at the end of the day, like at the last meal, because taking a picture of every glass of water you drink could be annoying.

Respondent 1: Or this might be a good time to do that same type of checklist function where you set a goal, how much do you want to drink in a day. And every time you drink a cup, you check off one cup, and then you have nine left.

Respondent 3: I would add that they could just, like especially for tea and coffee, especially if it’s plain black coffee or something like that, they have to keep in mind that that adds as hydration or as a cup holder, especially when it comes to tea. So, like Rebecca mentioned, just having a thing to tick or checkmark or let’s say if they have the goal thing. So, they could be able to track if they reach their goals for the day or not.

Respondent 2: I would envision almost because it's going to be an ongoing thing for the day. It's like if you have a little cup, like icon by every plate, but the icon just brought you to a checklist and you had like water milk or milk, alternative tea and coffee. And then they can just kind of same thing, like check off how many they've had through the day.

But then you can just add to it through the day, almost like a pop out. You click on it and you can just add to it through the day and be like, “Oh, I just drink two glasses of water and a coffee with my breakfast. So, check, check, check.” And then that way, if they don't remember everything at the end of the day, they can add to it.

Respondent 7: I think that's a good idea to have it like Clarissa just said, an icon that takes you on the page with a plate that takes you to where you checked it off. Because otherwise, if it's not there, people might forget to add it in. So, just put it right there so that they know that they can use it easily.

Respondent 2: Yeah, I would probably include too, just to be realistic with people like maybe alcohol on my list, because people drink alcohol for their beverages sometimes, too. And it might be something they're monitoring.

Respondent 1: Also, kind of interesting and this might not matter. So, for beverages, like if you did something like that, like you're tracking specifically type and what you drink, but like you're not doing that with food. So, it’s just like a disconnect there. But I don't know, I guess what's the goal that you want to accomplish with fluid?

With fluid, it’s the proportion matching the plate guide recommendation. But with fluid, is it just like do you want to drink enough fluid to be hydrated? Or do you want to know exactly how much water you drank and how much coffee you drank and how much alcohol you drank? I guess then there's two different goals for drinks and for your plate.

Facilitator: So, which goals could be included for beverages?

Respondent 1: Well, just like hydration to be really simple, I don’t know.

Respondent 5: I think like limiting sugar sweetened beverages is kind of a big goal. So, maybe it could be just like really simple like water, sugar sweetened beverage, alcohol. I don't know, I'm trying to think of what you could boil it down to, to make it really simple to kind of achieve the goal that you're actually looking to achieve. But I'm not sure.

Respondent 6: I mean, the Food Guide is just like water or not. That's how it is. It seemed to be presenting it. So, it's like, you get a check mark only if it was water than anything else would not. And that's like kind of problematic. I don’t know.

Respondent 5: Yeah, good point.

Respondent 3: We could also include the milk as a beverage or even milk alternatives for people who can’t consume milk or milk products. So, maybe with suggestions like oat, cashew milk or such things.

Facilitator: So, for beverages like that for dairy, or things that could be considered smoothies, things like that, would those be included on the plate or as beverages?

Respondent 1: Good question.

Respondent 4: Both.

Respondent 1: Yeah. If you're having a bowl of cereal, I count my dairy milk kind of as my protein but then sometimes, I just have a glass of milk.

Respondent 3: I would say both too, because like hydration is available everywhere. Let's say watermelon, stew would have a little bit. So, it falls into both.

Facilitator: Would you track watermelon as a beverage?

Respondent 3: Oh sorry, I'm talking more about hydration. Yeah, because the question, yeah.

Respondent 7: Maybe you should just be like to include a beverage with your meal. Or what was it? Like, dairy water, like whatever it was. Then it's not necessarily tracking but it's kind of getting you to think did I have a beverage with that?

Respondent 1: I do really like the idea of tracking water intake to ensure that you're staying hydrated. But yeah, I guess I'm just still kind of grappling with how important is it to track the other beverages specifically, and how to best do that.

Facilitator: So, I heard many ideas and just for the sake of time, I'll move on to our next section. So, it’ll be more about the features of a proposed application. The first one will be which instructions and support do you think should be provided to users to support their use of the application?

Respondent 1: You know how sometimes, I can't think of a website that does this. But if you’ve updated app or something that when you log on again, it gives you a quick tutorial, “Press here to do this, press here to do this.” If you could do that on the first go of using the app and after updates as well. And of course, have the option to close the update in case you don't want the tutorial. I always prefer to figure it out myself. It’s kind of nice that they have that in case you can't find something. So, maybe just a simple standard thing like that kind of guide.

Respondent 1: I agree.

Respondent 4: Maybe a section where there would be frequently asked questions.

Respondent 6: I guess something like if someone is struggling with disordered eating, or like kind of triggering with maybe some easy resources or websites or support.

Respondent 2: Yeah, it would be nice to have a list of, like she said, like linking to eating websites or was previously mentioned as well, linking to different sources to find dieticians and linking to the different colleges for each province or something, so that people can source dietitians if they need support.

Facilitator: You also mentioned lists of foods previously?

Respondent 2: Oh, yeah. So, whatever, I guess finalized for the different categories of the colours of the plate, probably trying to have a pretty expansive list that incorporates many different cultures and different foods that aren't necessarily on the picture that is already existing as the Food Guide, but includes a lot more as well.

Facilitator: So, I heard tutorial lists.

Respondent 5: It's a bit tricky, because if you offer something like chat, if you want to talk about this or struggling, I think it's the same thing when people call like the Eat Right Ontario that needs to have – you get into the realm of individual [unintelligible 00:58:19] with somebody versus just offering general support. That would be the risk of like the customer service number because all of a sudden, it's like, this is my situation, I've had this and this and this and then it gets a bit tricky. Sorry, that was like a non-answer.

Facilitator: Yeah, so having general information versus individualized. That's our job, right. Maybe referring out to dieticians as well and having that as a feature that I heard before. Are there any other features and tools that could help with users?

Respondent 2: Maybe just like, example, either photos or instructional video that shows I ate this and then this is how I put it into the app.

Respondent 3: Maybe recipe or like how to use a certain vegetable like a recipe if they have that certain vegetable or food that they want to use. Let's say protein, especially with a plan based now in trend. So, how can they incorporate more protein on their plate or in their meals? So, like the recipes, suggestions, such thing.

Respondent 1: Or there could even be like a feature between people using it like how do I increase my fruits and vegetables? And then like other users of it give tips. I just find with clients, they seem to respond better, even if you just say, “Other clients have told me this is what works for them”, instead of like coming from the expert or whatever. That just lends itself better. So, a way they can chitty chat with each other.

Respondent 6: Kind of community function.

Respondent 1: They have to be monitored though.

Facilitator: Alright, so moving on from that. So, which features could help with adherence to the application? So, helping people stick with it and use it?

Respondent 4: Maybe a reward system.

Respondent 7: I just get nervous too with reward systems because what are you rewarding? I guess making sure that there's no, again, guilt or shame or insinuation that they're failing if they don't succeed. What is the reward? And I guess, is that a healthy way to like live your life and eat by rewards? I’m not sure, but we want them to use the app. I don't know.

Respondent 4: Tracking progression and rewarding progression, depending on the goal of the of the person.

Respondent 7: Maybe something could even pop up like, “Hey, let your doctor know. You had half your play this fruit to vegetables all week”. I don't know, something like reportable. Again, you don't want to if they didn't do it, then it's like meh, but maybe something they can share with somebody.

Respondent 1: I don’t know if you guys have ever produced like the Duolingo app for language learning but that one has like “Congratulations, you made it to 10 days”, even if like you made it 10 days of tracking. It says, “Yay, 10 days”, that kind of thing. It's great if you made it there, but if you didn't, that's OK, too.

Respondent 4: Yeah, rewarding the positive. Not telling them when they do something against the goal, but rewarding everything they do that's positive.

Respondent 1: Yeah, like little things like, “Congratulations, you met your protein needs for the day, or you had veggies or fruit with every meal.” Or like we said before, just even rewarding the use of the app. If there's like a thing at the end of the day, they can say day complete and that’s like, “Congratulations”, like she said, with the Duolingo, “you've tracked for seven days.”

I personally, too, will depending on the app, I have it set for some and not for others. But I actually do like a notification sometimes. But I like when I can customize it. So, I know like I have one and I know I have it set for like 9pm at night because I know that's what I'm most likely to use this app versus just I hate when they just send you random throughout the day.

And you're like, “No, I can't look at that because I'm at work.” So, I think having the option for notifications if they want it. So, if they feel they need to be prompted to put their food in while they're sitting on the couch at 8:30 at night, then if they want to set up notifications, having that function as well.

Respondent 2: Just trying to think like maybe even some way to link like if someone like uses their calendar, what if they have like a busy day with meetings? Like maybe a prompt like “Do you need to eat before you go into this busy day?” Help with meal planning and that way.

Respondent 5: Yeah, like a notification, “You haven't eaten anything today. You haven't had any water.”

Facilitator: Building off what Michelle was saying before, could a social aspect help with adherence as well?

Respondent 2: I think so.

Respondent 5: I think so. People like sharing that stuff.

Respondent 1: I think you could as well but then again, just how's that going to be moderated and just keeping everyone I guess, on the right track for lack of a better word. And I guess keep out spammers even.

Respondent 7: Sorry, I'm just thinking you wouldn't want the chat getting overrun by people who are obsessed with really specific tracking. And then it kind of turned into a toxic environment.

Respondent 4: It can be an option because maybe not everyone wants to share their pictures and comments with other people. But if they'd like, they could have the option.

Facilitator: So, I’ll move on to the next question there, again, for the sake of time, so which features are required to ensure accessibility for users?

Respondent 1: It might be nice if you could potentially access the app offline. It'll update the database once the connects to Wi-Fi or something, but not everyone has data, and public Wi Fi can be kind of spotty.

So, you're out or you're at work and you’re trying to enter your stuff in and you can’t remember to the end of the day, that would be a turnoff for me to use it. So, a function that would allow you to use it wherever you are, or even if you were hiking or camping or something and it'll link back to the main database whenever you connect.

Respondent 6: That's a really good idea. And what was written in the chat, like languages and changing the font and things like that as well, for people with visual impairments.

Respondent 7: I really like the simplicity of it of using like mostly symbols and making it very intuitive. So, it's just easier to follow, reminds me of IKEA furniture.

Respondent 5: Maybe having it like, you can't control this necessarily, but like having a free version, and then a version without ads, I don't know, something to make it more accessible. Or like a simplified one, like a light version. And then the one that has the features of the chat and the community support and goal setting. Maybe different version like that.

Facilitator: Alright. So, I'll move on to our last question, which is just going to be about anything that we haven't mentioned yet. So, is there's anything that you think of, at this point, that we haven't talked about that would be useful to include in an app such as this one?

Respondent 1: The only other thing for me when I first saw the app and the colours on the plate, I was like something's quite a little bit off and I couldn't quite think of what. And for me, I realized it’s because there was no border around. It literally fills the whole plate, there's no border on the edge of the plate or something. So, is the food is supposed to go all the way to the edge of the plate? That's what I can imagine someone might think. So, just in terms of the visual of the plate itself, that didn't quite sit right with me.

Respondent 5: Maybe making, if they need to, a small note section, like they can make notes on their day, if they want to. If there was anything relevant like a medical appointment. So, ate out extra or just anything really, that pertains to their day that maybe they want to kind of keep track of, especially if they do – I know it's a self-monitoring tool, but if we were to use it with clients in our profession, it'd be nice to have those kinds of things too.

[End of recorded material]
