# Supplementary material for: Perceptions of self-monitoring dietary intake according to a plate-based approach: A qualitative study
Source: PLoS One. 2023 Nov 28;18(11):e0294652. doi: 10.1371/journal.pone.0294652 (PMC10683993; doi:10.1371/journal.pone.0294652)
Supplement: S5 Appendix — (ZIP) [file pone.0294652.s005.zip › Anonymized RD Focus Groups/iCANPlate-RD-Focus-Group-6.docx]

**iCANPlate-RD-Focus-Group-6**

[Start of recorded material 00:00:00]

Facilitator: So, this is the dieticians focus group on August 4^th^ at 10am. So, the first one will be kind of about the behavioural techniques and self-monitoring techniques that you use in your practice and with your clients. So first off, do you suggest following the plate method, as illustrated by the new Canada's Food Guide to your clients?

Respondent 1: I can go ahead and jump in, I don't know what kind of answer you're looking for. But I guess especially, my expertise being more of diabetes, we do use it. We also use kind of numbers in terms of portions because carb counting is also involved. But I find an easy way for a lot of clients, especially if they don't need to get into the carb counting and the specifics. It's a really easy way for them to kind of be able to plan meals, and I think a lot more realistic of an approach for the majority of the population compared to the previous version, at least.

Respondent 2: I can share from my perspective, so with eating disorders, absolutely not. I think, and we know that Canada's Food Guide isn't applicable to individuals with specific health needs. But I can definitely talk about how the Food Guide might relate to the field of eating disorders.

With vegans, what I would say is that, we probably haven't gotten into it much. But I do use the plate method when talking about certain meals, because I often find people who are on plant based diets assume they're getting lots of fruits and vegetables, because they're eating plant based.

But I often find sometimes that's not the case that it's sometimes lacking. Or if we're working from a principle of volumetric, I still talk about half plate. So, I do mostly focus on using the plate method, specifically for the vegetable portion.

And then with plant based diets, we talked about things, because often you're not doing the protein and the starch is kind of combined. So, I would say personally, for my plant based population, I do use portions of the plate method.

Facilitator: Interesting. How about the rest of you, do you use the plate method in practice?

Respondent 3: I see a lot of diabetes patients in the hospital as well. And kind of agree with Katherine, a lot of the patients that I see are newly diagnosed. And so, I find that's a really good starting point, especially for those people that are not – you’re not looking to do carb counting and whatnot yet. So, I definitely find it to be a helpful tool.

Respondent 4: So, for myself, it would very much depend on the population. So, currently more with older adults are those that are maybe struggling to eat, some of the recommendations are a bit opposite in terms of Canada's Food Guide. But those otherwise, maybe when I've worked with younger populations, just general college students in another job I had, yeah, a number of the principles I found helpful within relation to half plate fruits and vegetables, more whole grains and different messages.

But I think depending upon the individual certain messages can apply. So, even some of the qualitative ones about sort of enjoy your food and eating with others, for some that are maybe struggling with eating that those can work. But the plate would actually look the complete opposite maybe.

Facilitator: Yeah, especially for the older population, if that's not your goal. Get the fruits and vegetables in there. Alright, so what are the pros and cons that you've observed with using this method in practice? So, we've kind of touched a little bit on that. But what is it difficult for your clients to use the plate method?

Respondent 2: I can see from – so, I've worked in vegan nutrition for a number of years and that's always been my expertise. So it's very exciting in my field and with the community when this came out. But it's not very practical in the sense of ensuring – it’s almost like I wish there was an in between what we used to have and what we have now, because of the risk of yes, you’re never going to have an issue with protein.

But there's some kind of key nutrients on a plant based diet like iron and zinc and calcium, where I do try to be a little bit more prescriptive about including specific numbers of foods. So, I would say it's a great overview practicality, it's not always useful.

From the eating disorder perspective, I actually do think there are parts that are good that it isn't so numbers focused. That's useful for teaching, I think down the road about you know, just including a variety of foods. As I said in the actual recovery, it's not particularly useful and they're still about – our national food guides going to play to the greatest need.

And the greatest need isn't – as much as eating disorders are impactful of our nation, or of people living in Canada, it's not what most people are suffering from. So, I mean, the greatest pro is people I think are more trusting of the food guide versus previous versions as well. But I would say I'm kind of playing to more of the cons of how I don't use it.

Respondent 3: Yeah. I'd have to agree with Susan in the sense that, I think for clients, it can be really useful. For us as dietitians, it's helpful to get those numbers. I think we use to use, especially like in returning and in terms of assessing the diet, it's a lot harder to assess proportions. And it really is based more on the subjective measures that clients are providing. So, I'd say that's probably a con in terms of my perspective, as a dietitian, but hopeful to clients.

Facilitator:

Respondent 1: One of the things that interests me in that this focus group here in terms of tracking, and that is I've had experience with eat tracker and using it with sort of college student population. And I could see, in terms of tracking, I don't know how it would be conceptualized within the current version of Canada's Food Guide.

But I know some things are quite challenging in terms of depending upon one's typical food culture, and how they'd eat often with mixed different dishes. I think sometimes it's challenging to conceptualize that partition type view of one’s meals and same portion sizes being challenging.

And then also in terms of what one's eating throughout the day, and snacks and different items and sort of the bigger picture, as such. So, I can it. I really like certain aspects of the new Canada's Food Guide in terms of big umbrella statements and concepts and topics that discussed with that population, and actually better within the current Food Guide.

But in terms of tracking, just sort of maybe have challenges with getting my head around, how would one would go about doing that with the current version and my plate and such. Depending, it's people's such variety within food cultures, and what's eating and conceptualizing one's Food in that way. And also in terms of how that fits with some of the messages like enjoy your food, or some of the qualitative pieces and how that fits in with the tracking piece.

Facilitator: Yeah. So, I think hit a few of our points on the head today and why we're doing these focus groups to get your perspective on what you think it could look like. So, we'll definitely get into those points that you've all mentioned today. So, the second question will be which diet tracking methods or applications do you use with your clients currently in practice?

Respondent 2: So, for me, I actually, I'm a consultant with the tracking company, Chronometer. And so, I mean, I like using them. Because I know the developers and I know what's in it. So, I would say, with the population I use, I'm a big fan of that app, especially, I don't put eating disorder clients on it. But it's useful for me, in some circumstances, because I can turn off certain nutrients. You can't fully protect them from seeing some things, but it's really useful.

The other thing I do use is I chart with the program practice better, which some of you might be familiar with. So, they also have with certain levels, I think, a feature there. And so, I use that one a lot with eating disorder patients because it allows for visualization.

So, I can sometimes see what a person's eating and have more of the qualitative piece like mood and notes and thoughts and hunger and things like that. I used to use Eat Tracker, I would say when I first started my career and when I was more focused on servings.

But I again, especially with my plant based population, I work a lot with infants and young children. So, I need to know nutrient intake. So, that's where I would say an app like Chronometer is way more valuable versus just servings.

Facilitator: Yeah, definitely.

Respondent 3: Yeah, I can jump in and say that I use Chronometer as well, especially with the diabetes population, counting carbohydrates and specific nutrients as well for some that have other specific needs. I've used the You Ate app, which I guess is sort of a tracking more so in terms of mindfulness at meals, and mostly just takes pictures. So, it's a way, when we look at kind of the division of the food and the proportions of one group to the other that I can sometimes use to maybe confirm some of the information clients are providing, but actually get more of a visual idea of what their meals are. So yeah, I think it's You Ate app.

Respondent 5: I have you Eat Tracker in the past, but for my clients, I tend not to recommend – like I don't have any apps that I recommended so far, just because I find that they can be quite overwhelming, a lot of information, and they tend to not focus on the basics, the principal things I'd say. So, I'd rather help them with their meal planning.

I would use the healthy plate method. So that's why I like that focus group, I was intrigued like what the app look like because the healthy plate method, of course, because there's not a lot of people I see that they eat half the plate as fruits and vegetables and stuff like that, but it's to work towards goals. So, I'm intrigued to see if the app is simple enough so that the general public can use it without getting lost in the information.

Respondent 1: Yeah, and I guess I already started off this question a little bit with my prior response, I have used Eat Tracker with a college student population, and it was sort of an exercise to reflect on what one's eating and comparing also to paper record and to Eat Tracker as well.

But yeah, some challenges in conceptualizing portion sizes as such, in relation to what one was eating. I've also used a while ago at a different role, photographs, not so much for tracking in terms of what one was eating, but reflecting on what one preferred foods and of what one was eating on their meal of where they're getting certain nutrients from.

Also, in more institutionalized environments, acute care, using self-tracking of what was served from the menu, and particularly tracking things like oral nutrition supplements, and snacks and beverages in ones that are having challenges with eating enough. So, just a sort of a basic paper version of a sort of a preformed after the sort of the institutional menu, a bit of a variety.

Respondent 3: Yeah, it'd be similar to Janice like in the hospital, it's mostly paper tracking. I mean, we have a database with all of our menu within it, so then we can just kind of run it through that. But honestly, I was more interested in learning about other ways to track more for my pediatric outpatient. So yes, I'm hoping to learn more about options.

Facilitator: OK, great. And do any of you know any diet tracking methods, be it paper or applications that currently resemble the Canada's food guide or the plate method?

Respondent 1: Nothing that jumps to mind for me personally.

Respondent 3: Are you referring to certain specific tools or apps that that mirror the-?

Facilitator: - Yeah, if you know of any tools that allow people to track their diets as per the plate method?

Respondent 3: Not that I can think of.

Facilitator: OK, great. We do ask this question to make sure that we're on the right track for that. If you do know anything, please let us know, but it is good to hear that you don't know of any. So, we're on a track to be able to create something new and novel.

Showing the app’s prototype

Facilitator: Since based on this, what are your immediate reactions?

Respondent 5: I don't have a solution for it. But a lot of people it's like, for example, to have macaroni and they’ll throw a can of carrots in there, for example. So, it’s just a general challenge we have when using the healthy plate method. So, it's when the veggies are mixed up there, it's going to be hard.

Facilitator: Definitely. Yeah, so mixed dishes, in general.

Respondent 1: The first thing that comes to mind is that I can already see certain aspects of Canada’s Food Guide are dropped because it's sort of vigorous sort of messages and not just the proportions, but also the quality. So, even just saying grain, sort of more whole grain or certain qualities within the foods and sort of what types of – yeah. I need to see a bit more.

Facilitator: Yeah, for sure. So, this is all we have for the moment and we're looking at you to see what you would like to see.

Respondent 2: I think right away, it's that if I was a user, I would be like, and I mean, I'm sure you're going to get into this, but people like to know about their macros, whether or not I agree with it, or they want to know, I'm sure you're going to get into this, but if it's like putting in these proportions, and it's spinning out to you that this is going to ensure you're meeting XYZ nutrients.

But I think what Janice was saying is that quality, for sure. I don't like certain food groups, like for sure, I have my own ideas about the dairy kind of food grouping, but I think people can kind of easily see dairy foods are a protein food. But I mean, fruit is another category, where does that fit in? And if I'm having breakfast, that's typically where I'd have fruit.

So, it could mislead people into believing that their overall dietary quality is lacking when it's not. So, I mean, I guess just seeing more. I mean, it looks simplified. I think for people new to this country, it's a lot of whitespace, it’s very clear, that I think it would be easy for a lot of people to use. But yeah, I think definitely needs to see more.

Facilitator: Great. So, I'll stop sharing now and we're going to turn it more towards you. So, you're going to be the ones deciding what you'd want to see on this application, why we're having these focus groups today, and seeing more to be able to create more and add more to this app. So again, this is the very baseline framework of what that main page could look like.

So, what I'm hearing is that maybe you'd like few more categories within the three food groups. So, Susan mentioned separating out the fruits and the veggies potentially, Janice mentioned having more of a qualitative piece on whether or not that grain was whole grain.

Respondent 4: And I think also in terms of how certain key elements and changes to the new Canada Food Guide that I actually really like, that are missed in terms of more plant based proteins, the variety. It really strips off a lot in terms of, I think, a lot of thoughtful consideration in the visual depiction of different foods. And also, that piece about less processed foods cooking more. So, the example of using it with, we’ll call it more college age, and this one, my prior work, and a number of things that I really like about the new Canada’s Food Guide, it’s gone.

Facilitator: Yeah, so our goal would be to put them kind of back in and see how we can conceptualize having those more qualitative pieces as well on the guide. I'm just going to refer back to my focus group guide here, we'll get into the more qualitative pieces a little later. But how would you do this application working to record all meals throughout the day, so breakfast, lunch, supper, as well as snacks?

Respondent 3: I would kind of see it as an entry similar to other apps that exists, add an entry, have a timestamp that maybe the default would be like the time that you enter it, but within the ability to kind of change it, choose what meal it is, and maybe choose the plate. And then I could see kind of the second page or after you chose your proportion, maybe have those elements that Janice was mentioning.

The back of the food guide or was it whole grains? Were there some plant based protein? Like having that more detailed information for people if they want to track like, they could kind of track that as well? Because I agree with all the comments that were brought up, but that's kind of how I'm visualizing it initially.

Respondent 5: I agree with Katherine too. And also, people are driven by goals. So, I don't know like, we could have something more like, for example, “Did you have an orange? Vegetable?” Then you put the entry in there's a little like checkmark, that's like OK, that's met kind of thing. And as you do the entries, maybe like that healthy plate method could fill itself up. So, you're trying to meet the goals.

Respondent 2: I think too, when I see this app, I see it being much more useful as a planning tool versus a tracking tool in some ways because working with certain populations that are transitioning to plant based or want to improve the quality of their diet, we're often doing meal by meal.

So, I could see this app working as OK, how do we build a healthy breakfast, and that maybe you're putting in what you want to eat, and it's spitting out information based on age, gender, activity level, etc., dietary needs, and suggesting that this meal would meet certain key nutrients. And that would could help you in planning the remainder of your day.

That's something I would say is lacking in the market a little bit in the sense that there's plenty of tracking apps, which are very like retrospective, in that you enter the information. We go back, we analyse, and we as dietitians use it to build. But if you can have something that serves, people don't just build a healthy meal, build a balanced meal, and save this meal as this is like, “Oh, this breakfast is like giving me X amount, that this is a key breakfast to have.”

So, that's what I see in terms of what's lacking, and what I'm doing a lot of. I'm not trying to put myself out of work, but what I think as dietitians, is that having people just be able to have the ability to build their own meals based on information they're getting would be a huge benefit.

Facilitator: Yeah, that's definitely a very interesting point.

Respondent 3: What Susan was saying, makes me think of like the FODMAP, the Monash app, where I think there’s that section where you can get the information like on each food and what kind of FODMAP they contain, but then there's a recipe section, if I recall or even some of the GI tracking app. So, it could potentially serve like the two purpose like a section that helps more with planning or gives you idea to help kind of meet your goals, and then maybe a section more for monitoring, in terms of what they actually were able to meet like something like that.

Respondent 4: I do like that suggestion of planning piece. And I think that's also another thing that, although not depicted when you showed Canada's Food Guide and the two sides of that single page, but they've also got, where they're putting recipes on there, and different inspiration within cooking and cook your own meals and such. And maybe being able to get some support in that realm.

I know you did make that caveat at the beginning, like, “Oh, this is not intended for people with eating disorders or not for people with current health issues.” But if the first page they begin with and seeing the plate and reflecting – my concern is where people are left with self-monitoring and just having some sort of reflection in terms of where they maybe do need additional help in some way, shape, or form.

And maybe also being able to see some of these apps in relation to what we do have resources available for individuals. And I think of specifically here in BC, where we have Health Link where you can call to a dietician and that, because we can do a lot, like it's nice enough to say not people with eating disorders, and then also current health issues.

Sometimes people aren't really fully aware in terms of what health issues they might have, and how that impacts on what goals that are maybe applicable to them. So, I guess advocating for our profession a little bit as well, somewhere having a plugin for that, and also reflections on is this applicable for me?

Respondent 2: Yeah, I agree with that. What I would say is, it's great to say that this isn't for – we’re not designing this for eating disorders, but for there to be someone puts in health conditions and their eating disorders, for a pop up to say that this is recommended for the general population, please consult your – I think that there can be a lot to be said of like, telling people that this isn't right for you, this isn't a tool for you because that can go far in I think helping people in that field.

Facilitator: Yeah. And maybe even being able to see some of these tools in supports alongside with having a dietician or being able to consult with someone or and then going on with the app and that because sometimes it's hard to know am I the general population?

And even within reflecting as within one's professional skills, and that sort of contemplating in terms of to what extent would I want to recommend that my plate and an app like that for one's clients, because I think, there's a really good proportion of the population that are living with chronic diseases, multiple chronic diseases.

We have an aging population and that. Anyways, that's a little plug or caveat. Maybe get advice from your health professional before kind of going off on one's own and making one's own goals that could actually be counterproductive and actually causing more harm.

Facilitator: Yeah, definitely. We are going to keep dieticians at the forefront of this project and having nutrition professionals plugged in. So, having that little caveat, I think at the beginning, if we do like a mini screening, for sure would be a great idea.

Respondent 1: And even instead of going straight on to the plan, I don't know what the intention was in showing the plate there. But I think my heart’s up where I’m like, “Oh, all the good stuff with Canada's Food Guide” with some way to be able to reflect on some of those bigger ticket principles of the qualitative piece and somewhere being able to compliment that.

Because when I reflect on Canada's Food Guide, and I've actually kind of noted around and looked at the evidence behind it as such, and there were certain elements of the plate and how its depicted come with sort of bigger overarching topics related to certain chronic diseases and research behind that. But when you kind of strip it down to very basic colours, very basic descriptors, I'm like, ah, is this even evidence based as such? Anyways.

Facilitator: Yeah, so what would you like to see in it?

Respondent 1: Don’t lose the qualitative piece around it as well. And I think focusing too much on just proportions and amounts, you kind of throw some of the Canada's Food Guide out.

Facilitator: So how would you see the more qualitative elements of the guide being tracked on this application?

Respondent 3: Like I mentioned before, I would see it as kind of a separate, like if you want, kind of like the food guide right now, there's the front and the back page. There could be kind of one page on the app that's more for tracking the proportion. And then once you complete that, it kind of brings you to the next one, where those different behaviours are listed.

And whether it's a yes or no, kind of a Likert scale like something you could drag of like, yes, some whole grains, but not the whole portion, or kind of a way to track that separately. And I mean, have people track what they want to within the app but have the option for all elements of the food guide, because I do agree with Janice, that I think it should be captured. That's kind of how I viewed it as two separate things, but still part of the one entry.

Respondent 2: I could also see like a stage wise thing, because I've worked in family health teams and community health centres, and for sure, there's levels of nutrition information that you're providing based on someone's readiness and their nutrition, knowledge and understanding.

So, having it be like, OK, I ate a protein food, and then you click on it, and then it's almost like, I don't want to say like gold stars because there's a game. I was talking about how some of these apps have a gaming approach to them. And sometimes people lose themselves to the gaming approach, and they forget why they're actually tracking.

But if you're clicking on protein, and then it's saying was this plant base? And you check that, and then it's like, you're at a next level and saying, “Did you cook it in a way? Did you eat with family?” So, where it could be just check marks, that you're not losing anything. You're not being made feel guilty, I think that's really key, but you're being reminded of what are kind of those big picture goals.

And same things with whole grains, was it whole grain? I don't know, something like that, where it's almost like you open this up, and then there's like a pop up screen where you can check off because I think checklists are also very effective. What I would be doing is, OK, build your meal. This is your daily checklist, did you include, at least for me, if I'm always asking for at least one cup of plant milk or things like that. So, I think looking at levels and things, those are the qualitative pieces that I feel are missing.

Facilitator: Moving maybe away from that plate, and the proportions on the plate and moving towards a checklist?

Respondent 2: Well, you could start with the plate, but I think having a checklist as well as beneficial. Also, another app is, I don't like it, but I referenced it sometimes it's Dr. Greger has one that I mean, because I just think it's overwhelming for people, but where it is checking off servings.

I don't know if anyone's familiar with it, but it has like specific amounts if it's a plant based one. Did you include X amount of vegetables? Did you include flax seeds? And it's just filling in circles, for example. So, I think that is useful as well for people because it's kind of a measure of proportion. So yeah.

Respondent 4: And in some ways that you're saying, what kind of other things beyond the plate and that even if one kept what was put, I think quite considerably time and effort in terms of creating even that visual depiction that is Canada's Food Guide. But being able to zoom in on certain sections. The whole grain, what is about whole grain?

Maybe having something where you're focusing more within that and getting inspiration to variety, and considering whole grain and being able to focus on that as a goal and something you're wanting to work on.

It’s quenching my thirst with water reflecting on throughout the day, what beverages and I'm consuming and sort of how is that contributing to my nutrition, focusing within fruits and vegetables and maybe trying to promote and get inspiration alongside with focusing on very targeted messages within Canada's Food Guide.

And it's even something where one could work on those different messages or ones that they feel they're struggling with or something they want. I want to eat more plant based and maybe something within sustainable. I think we hear a lot of people and then focusing on that piece, getting inspiration, getting recipe ideas, menu planning, what have you.

So, the possibility to maybe start with a visual depiction of the plate and people could go towards the proportions as one of many different facets within that they could focus on within an app.

Respondent 1: Yeah, I like what was mentioned, and I don't know, I'm kind of thinking of was mentioned before, but like with the chronic conditions. If you fill that out, like if you feel that you have an eating disorder, that a pop up would show up. I think kind of in that same idea, having a way to individualize the app and use it for what your goals are --- related to your goals.

So, if you were to start in create your account, you'll select, “Are you interested in focusing on the water piece, the whole grains”, and maybe that would help in making it simple for that person where one person might want to focus on water and grain, someone else might want to focus on plant based protein.

So, maybe, then the pop ups would kind of be different. So, having that kind of individualized approach, which I think is where we're going with nutrition as well, I think could be really neat to have.

Facilitator: Yeah, so having more of an individual goal setting.

Respondent 2: I agree with that. I think that that's crucial and really helpful that that can be self-directed, or from a healthcare professional saying these are the kind of key areas. So, if you can put that in, and the app just guides you how to build a meal, meet certain key targets that are helping to tie back to the vegetable, I think is really beneficial for various populations.

Facilitator: So, what would you see as options for goals that people could set within this context?

Respondent 6: I think they're all contained within Canada's Food Guide, a number of the key ones. I think there's a lot of thoughtful consideration of ones they're pulling out there, but just, to have those different nuances. Because within the back, what you showed as the backside there, even the website with Health Canada and Canada's Food Guide, they have a lot of the wording and everything, it's all there but it's being able to incorporate some of those qualitative pieces.

I’m just trying to remember if it's this food guide or previous ones. Like advice when you're eating out, or this or that, or different scenarios is such and so dependent upon the individual, what resonates more with them.

Another, and this is totally maybe a side comment, and sort of concern, or sort of maybe opportunity. We mentioned with people with chronic conditions or maybe eating disorders, and being able to ask questions in that of where there might be points of concern with that. Another point, and I don't know, to what extent can be incorporated on the app is people that might be struggling with food security, and how that’s thought of as well.

And that's one thing even I think maybe was even criticized with Canada's Food Guide and how many can afford this or that. And in essence, when you think of if this is for helping at a societal level and such and so of individuals, I think of greatest concern, and not to mention with pandemic probably making it even more challenging with time to come.

Yeah, I don't know, resources for supports for individuals that are maybe struggling in that way. Yeah, I don't know. It just sort of further catches on the complexed nuances of what challenges people might be having with their food, but I know that an app can't solve everything.

Respondent 2: But I do think in terms of goals, I think trending, so a lot of people, for sure, this promoting more plant based is OK, so that's my goal. How does that help? I think also sustainability is that I want to eat a more environmentally conscious diet. So, if I put that in this goal, what are your recommendations? Or, we know weight loss is going to be a huge thing as well. Or, for kids and how to help feed. So, I think it's just really the sky's the limit, I think even from a food security, because I know Ontario had like a meal planning app back in the day, I haven't seen it or used it in many years.

But there was that piece a little bit where you could set your goal, print out a menu, the recipes, the grocery shopping list. Again, I think that's a lot to ask from an app. But I think tying in these pieces, and what Janice is saying is that, well, these are my goals, and then having a you go through like FODMAP. It's like the app that you're mentioning, is you can keep going through, and it has resources and connect with a dietitian, connect with a healthcare professional, where it doesn't have to be specific, or it could be specific to what the goals put in.

But, I mean, there just could be the sky's the limit in terms of goals or like diabetes is one. I don't really work a lot with diabetes right now but I think there's a lot that could be done.

Respondent 4: Just to kind of to do with that point, being able to link clients to resources, and some of them being free resource in particularly, I think people with food security. I think that can be a challenge within itself, even for other health practitioners or colleagues to know where to refer and such.

So, even to have where, regardless, we can be able to make those connections and something like an app like that has kept up to date. That in itself can be super helpful because all of us can link to clients within different ways within our roles and such, but how do people know and to what extent that they can get those supports?

Respondent 5: I'm not sure if it has been mentioned before but just referring to the video you showed us at the beginning. In the general population, I feel like people don't really know like if you put a bunch of food they don't know like, OK, this one goes in protein, this one goes in veggies, just bear that in mind. So, I think Catherine dimension like data entry. So, for example, if the person writes down potatoes, then it would go automatically into the starch section. And it could have like, I don't know have little picture to make it fun because if it's just like ratios, it's kind of boring, I guess. So, if it looks good, the attention is more into it, I guess.

Respondent 2: Something else just popped in my head too, with so many mixed dishes, is if you have a general sense of if you had a plate for the day and you were kind of entering your food, and then at the end of the day, it shows you your plate proportion that of everything you ate in the day, 50% of your food choices came from fruits and vegetables, this amount came from grains. And then it tells you this is your proportion of whole grains, protein wise.

So, almost too that people are just entering their food and it's like throwing it – the app is doing it for you. You’re putting in, so let's say I have like a lentil shepherd's pie and I put it in the ingredients or whatever. And then it threw it on a plate at the end of the day. Personally, I would be interested in out of the food I eat, what proportion of my food is coming from plant protein versus fruits versus vegetables? That's something that I think I personally will find beneficial. I think clients would as well.

Respondent 2: Yes, well, because some people will more eat six small meals instead of three meals or two meals. So, it wouldn't encourage, for example, “Oh, my fruits and vegetable are really low. I’ll have an apple with some peanut butter for a snack.” So yeah, I do like the daily. That's a good idea.

Respondent 1: Yeah, I like that idea of the food entry too. I think a lot of people, they may not enter totally accurately if they're just putting the proportions because people might under or overestimate what exactly they're eating. And then also, like you said, might put it into the wrong category. So, if the app is kind of doing that for them, then that takes away a lot of the error.

Facilitator: So, would you see that data entry looking something like the other apps that are on the market?

Respondent 2: Chronometer does do some pretty cool things nowadays where I'm not sure, like if you go to their site they have – if you set a goal that you want to track like immune fighting nutrients or things like that, I haven't followed it very specifically but I do know that they do things along that showing wheels of Vitamin A, Vitamin C, zinc.

They also have something where it evaluates how well you're meeting all your vitamins and minerals and it says like you're consuming like 95% of your required needs of these foods. And so, I do think Chronometer allows for that.

And the biggest issue is, I would say human error, in that what people are entering isn't correct or then there's also an issue with label. So, a lot of times the Chronometer database, we don't have USDA grade of info information on a food package.

So, it's also the issue with that, that I'm finding. So, it’s showing like incomplete information when it's not. And I've had this in practice for someone saying, “Oh, I'm supplementing all these things, because I'm not meeting them”, but they're scanning everything. It's like, no, it's just not showing up, these things are there.

So, I think that Chronometer is doing something, but their focus is more on vitamins and minerals and actual nutrients, where I don't see there being something that's based on food. So, as in having proportion of showing how much like fruits and vegetables you're intaking in the day out of a certain proportion. I haven't seen that yet.

Respondent 3: Yeah, I can see the issue though, because basically, what we're talking about would be if they enter orange, it would be categorized as fruit. And then if they enter rice, it categorizes grains. I can see the issue though with those like other foods like cake and where do you fit in because that's not really on the Food Guide. It says to limit but that could be something to maybe figure out of how you'd want that to show up, but would kind of fit into that food quality piece. Just something that kind of popped into my mind, but I do like the ideas that were discussed so far and I agree with all of those.

Respondent 4: I think it's funny that popped into your mind, because the same thing did for me, because when the mention of orange. I’m like, “Orange? Orange flavoured gummy bears” because they also have messaging on there, also drink [Crush? 00:42:18] if there’s water, not orange juice.

So, there's even certain aspects of categorizing within those groups of fruits and veg, protein, whole grain that actually have evolved and changed somewhat between previous versions of Canada's Food Guide. So, I can only imagine how confused people could get in terms of being able to categorize and such.

The one thing I have a challenge with focus on the plate and divided half fruits, veggie, protein, whole grain, and focusing on that piece of Canada’s Food guide, I think it sort of misses the point. Because of with that visual depiction, it's more so many people not eating enough fruits and vegetables is something we see in people eating habits and trying to get people to eat more fruits and veggies.

But that piece of actually having it specifically to the plate, and every single meal focusing half fruits and veggies, quarter protein, quarter grains, I have no knowledge to my recollection of sort of evidence or studies of specifically that piece being particularly evidence based. And if anything, I can see the spirit of how it comes from trying to increase people's fruits and vegetable consumption and seeing that aspect of half the plate.

But I think focusing on looking at every meal and how it's the proportions are in that way, I don't see that as particularly the key thing from Canada's Food Guide, I want to necessarily put into an app per se. And also, that what one has at every single meal, you can vary from meal to meal and that's completely fine.

When it comes down to it, it's what the bigger picture of what you have in the end. But I think that that can be missed sometimes when we focus on every single meal you have exactly and that's also a problem I had with the previous Canada’s Food Guide. So, I think the big ticket items can be lost in that focus.

Facilitator: No, that’s a great point. And the evidence that we do have with regards to the plate method is more as a teaching tool, and using it to teach people how to plan their meals essentially.

Respondent 4: I’m aware some food guides where it's formulated more in terms of moving in a particular direction. So, as opposed to seeing the plate and focusing on the proportions and that's the key aspect. But having as goals in terms of I want to increase my fruits and vegetable consumption, I want to increase my whole grain consumption. I want to eat less high sugar, high salt, so on and so forth. I would like to eat more plant based protein. So, more so those overarching methods or decreased my salt in my in what I'm consuming or eating less processed foods. I struggle in terms of conceptualizing how to get that within the proportions piece.

And that's in some ways, I think, focusing on a bit the spirit of the previous Food Guide, and how it was very much proportions, very much serving sizes. And in some ways, with the new food guide, what I really like about it is that we're moving away from that. But in some ways, I think focusing on the proportions and that aspect, and that spirit within the Food Guide, you're kind of missing what I liked that there may be moving away from.

Respondent 2: But just to Catherine’s point, I just wanted to add too that I love the idea of the app doing it for you, because sometimes people just have no idea what amounts I call them like fun foods or sweets and treats. And I think it's, to me, what's so crucial is that, like a French fry shouldn't be showing up on the plate, we shouldn't be misleading people to believing that your process, like this type of food is really contributing to your nutrient requirements.

So, there's so much like grey that what we would count as a fat Canada's Food Guide proportion completely lost in nutrient qualities. So, I think that is a really crucial part is that where you can see and that's even tying in maybe like with the Brazil Food Guide, is you're looking at the degree of processing and categorizing that this is an ultra-processed food. Yes, it's a protein.

I think it is really important to be able to, if we're using this as a tracking to be in line with Canada’s Food Guide. It’s not losing the nutrient quality and showing people this because I think everyone's very interested in how processed is my diet, or what proportion of my food or energy or calories or whatever is coming from kind of sweets and treats and indulgences versus full foods. I think that's something useful that could be captured as well.

Facilitator: So, coming back to those other foods, so we have things like desserts, processed food, things like French fries, chips, ice cream, pudding, cookies, cakes, everything like that. We also have all our fat sources. So, oils, butter, mayonnaise, condiments, ketchup, mustard, and then all the seasonings as well. So, salt, especially pepper, any spices, which could have interesting nutrition as well.

I also have added sweeteners. So, things like sugar, sugar substitutes, as well as any supplements. So, vitamins, minerals, if anyone's putting flaxseed in their oatmeal, we don't have that on the guide either and it's very hard to categorize that with a new group. So, things like maybe more international foods or international snacks and mixed foods that we wouldn't necessarily place on the plate. How would you see those entry into an application or any to a tracking method?

Respondent 3: Yeah, I was just going to say what popped into my mind with the discussions around these other foods was just from my experience in research, the Nova classification system, which is a way to classify foods based on food processing. Obviously, it's not necessarily something that the general population will be aware of, but using some kind of evidence based way to classify these foods because it can become kind of a grey area of where does it fit.

So, I would see it kind of like that. I think it'd be important to differentiate, it wouldn’t just all classify like flax seeds, which are very nutritious and oils, which are healthy. It’s just more so the amount that matters with French fries or cake.

Like to me, I think these need to be differentiated. But using some kind of tool that already exists to help in classifying whether we want to focus more on the processing piece, or whether you want to focus really on these different groups and including oil separate from the sweets. So yeah, I don't know if that's making that much sense, but that's what comes to mind.

Respondent 2: Yeah, and this is probably one of my criticisms of Canada’s Food Guide is that it’s wonderful that it is simplified, but at the same time, it's over complicated, but people don't realize how really complex it is as well. So, I mean, you have to have some sort of – the plate method is just not going to be enough that you have to have some sort of categorization or a way to account for fats and oils or other food group. And what does that mean?

Because I think again, people often when they track is working with eating disorders, even the non-eating disorder population, there's so much judgment people feel from tracking, if it's not in line with what they believe to be healthy. So, they just stopped doing it, because there's so much judgment. So, they lose the ability to even learn about their diets. So, I don't know the right way to account for that.

But I think what's clear is that an app that really has meaningful impact on people's lives needs to incorporate more than just one system of analysis, it can't just be the plate. There has to be the DRI’s, there has to be possibly ultra-processing categorization, that there needs to be something additional, if it really is going to be impactful to meeting what Canadians need and overall wellness in terms of diet.

Respondent 6: Yeah, and I think it doesn't exactly address this question, but related to other things that were brought up, it could be to go about it with a different approach. Rather than being this is a food guide, and no, you're not meeting half the plate, it could be like track for two weeks, or however long, we'll give you your data. And from there, “What's your goal?” Kind of similar to the Apple Watch where you've done this amount of activity or you’ve filled your plate with this amount of vegetable, try for more.

At least that's my approach, as a dietitian, with my one on one is this is the gold standard would be the plate method for most, but where are you at? And what's kind of a step in the right direction. So, I'm seeing how that can maybe tie in with what Susan's mentioning and Janice, where are you at? How can you improve that rather than really setting everyone’s goals it the healthy plate or are those specific guidelines.

Respondent 4: Oh, Catherine, I love what you said and it just got a thought in my mind, because in terms of tracking and it's not with food, but recently I got from my husband, those iWatch or what have you. And the idea of how there's those rings, where they have the goals of how much you're in movement, standing and exercise.

And seeing in terms of how one could maybe have within – and it’s quite rich in terms of the extent of number of goals one could pull out from Canada's Food Guide when you go with the qualitative pieces from processed, whole grains, more plant based.

You could have a whole long list of things, but if you could have where you go through some of those points and maybe some of the goals where people feel like they're struggling with or that resonate with them or they feel something they'd like to work on that you could choose some items of maybe two or three or whatever.

And you could have your rings and have very sort of basic sort of higher level goals in terms of incorporating more fruits and vegetables throughout the day or what have you. Or, I'm drinking more water as opposed to other beverages. So, very high level type goals but something where it's very visual, and rewarding, because my watch, “Yay, you filled your rings or whatever”, you get that kind of satisfaction, like you're accomplishing something.

The thing I like about that is it's kind of higher level and kind of a bit broader strokes in terms of tracking is one of the issues I had with Eat Tracker and concerns there when you look at a very comprehensive, “What am I eating throughout the day?” I track it all. And then you get some results in terms of they would give their calories, how you met within DRIs, proportion macronutrients but then they also put it in relation to number of stars within the different food groups in that.

Having used it within a good-sized group of college students, there's so many errors. One food item and they entered it on the wrong portion size and it completely messed up in terms of micronutrient content. They’re like, “Oh my gosh. I have such and such.” I’m like, “Whoa, whoa, whoa, you made an error in your tracking.”

So, I think when people are left to their own devices in terms of that piece and to kind of reflect on the potential, accuracy and one's ability to conceptualize portion sizes and within tracking it to kind of go more at the broader strokes goals as opposed to the comprehensive, “What am I eating throughout the days and proportions” as such if that makes sense.

Respondent 2: I noticed from when you brought up the first page people could right away alter like their vegetable in the grain. Is that going to then be no longer consistent with food group because people might put it to zero grains? I don’t know, I think there should also be like a rationale of why do we include whole grains? You want to get your fibre in. Because people don’t – I don’t know, they hear the media and things like that and they take these nutrition messages and change their entire diet based on it.

So, I think having guidelines that say, “No, this is why we want you to eat grain. Do not lower your grains past this point, or whatever.” Because I can see right away, people just go and burn to the carbs and changing and just be like, but now it's just a fad diet. Now it's being used, like a fad diet. Maybe it’s good, maybe it shows people, well, then you're not getting any of your nutrients better or whatever.

Facilitator: Right. So, moving on, just to the beverage piece. I've heard water coming up a few times in goals, how would you suggest that beverages and sugar sweetened beverages, juices and things like that be tracked? Would that be similar to what we mentioned before?

Respondent 2: I think that's hugely worthwhile. And I think being able to track people's alcohol would be like, I would love, and I don't know, if we're there yet that we can say put a hard limit on alcohol yet, because I think there's still a lot of debate. But I think having people track their fluid and again, and proportion of what our low calorie or your beneficial choices, including these choices give you this, whereas, I think that's a really crucial thing for people to start recognizing.

Facilitator: Yeah, I think it would just be how do we want to track it in terms of just the types of fluid or account the amount of fluids total based on your needs. So, I think that would have to be figured out in because there's so much I think, that could be monitored in terms of fluid, like the type, the processing or calorie content, like energy density, caffeine, alcohol. So, I don't know that I have an answer.

But, again, making sure it's simple enough and kind of high level like Janice was mentioning, but still these really important elements. And I think when we think of the general population and the general healthy eating goals, definitely alcohol is a big one that can contribute to issues with weight management, or necessarily contribute, but in terms of weight management, some people might not realize like the amount of energy coming from fluids, whether it's alcohol sweetened beverages. So, maybe thinking of that as to what would most of the population benefit from in terms of goals.

Respondent 5: If it's data entry, let's say, I don't know if someone has an iced cap, then a beer, then a cooler, then a beer then a cooler than sparkling water, I don't know if like in the little glass, it can add up the sugar. People don't realize how much sugar there is. So, if it keeps adding up, it's like a why in the whole day, I have like X amount of sugar in my beverages? It's kind of like OK, iced cap, I didn't realize that it was actually like calorie denser.

Respondent 3: And then I also reflect on within beverages, this is embarrassing. I don't know how embarrassing. Milk because it used to be within very much kind of highlighted in terms of, when am I talking about beverages, within the milk and milk alternatives in that group. Would that be considered protein group?

Facilitator: That's actually our next question. So, how would you suggest the dairy and specifically fluid milk or soy milk be categorized?

Respondent 2: So, I can say, of course, being a plant based dietitian for all my career is that there's so much misguidance right now that I'm happy there's not a dairy food group, but I'm not happy that there's not a calcium food group.

Because as countries, we can’t agree on how much calcium people need but I do think that we need to highlight calcium choices in a way that I just don't think we don't need to just – it can just be dairy has calcium. It doesn't have to be, “You need to include dairy and this is why” and just understanding.

So, I have my own food guide I've been using for years of the vegan food guide and I just have a calcium food group. And I do exchange based meal planning with my eating disorder stuff. So, I mean, it's just sometimes I talked about this is also this choice is that this fits in both food groups. But yeah, I’m glad there's not a dairy food group, but I'm lacking in the calcium guidance. My personal takeaway.

Respondent 1: And I think based on what we talked about and are suggesting in terms of visuals, I could see if you enter the different foods, it's categorized as protein where it should be. And then from there, if the app has data, well, you had this proportion of protein that are high sources of calcium.

Again, going to that next step like Susan was referring earlier, depending on how they want to customize it, maybe have that as an option or not to make it a bit more detailed or not, but I definitely think as well, that would be helpful.

And just having it show, again, it’s more high level but still having that information. And whether it's rings or like a column like showing the proportion coming from calcium without necessarily having a guideline present, but at least just to monitor in terms of based on yourself, what's your progress? Are you always consistent? Are you lacking a lot in some days, not and others? I think that kind of information would be valuable.

Respondent 2: Yeah. And I see that too, and being like that you have the Food Guide because people don't realize how much calcium is just spread throughout your day. And that's where I think with the DRIs, people get bogged down into iodine. We can track iodine, while there's no sense, looking from a public perspective, there's no sense in necessarily highlighting that as a nutrient because right away, you're going to get people running out buying supplements.

But if you could have some nutrients, I think that are highlighted, I see a plate method, swipe, Page 2 is like calcium, iron, zinc, whatever. And Chronometer, I do think is worthwhile on this, because right away, I just hover my mouse over it, and it tells me where their sources of calcium coming from. And I think it's useful for people to then see, oh, that daily kale I'm adding is adding a bit. Oh, I didn't know tofu is also giving me protein and calcium.

So, I think it has to be accounted for. I don't think that there needs to be what there was in that, as I said, I'm so glad that piece isn't there anymore. But we need to have a way to capture as I said, it's just not enough to have a plate method. You're not going to capture the full nutritive quality a person's diet should be without that other system being integrated.

Respondent 4: And I think also when thinking beverages, it's kind of putting one's thinking cap on is where does that come from that we're depicting water? And I think it’s from, was it sugar sweetened beverages and concerns of how it's very easy to consume our calories in that form?

But then it's also, reflect OK, who is this exactly? Is excess weight of a concern? Is this an issue for this individual? What are the beverages they’re consuming? Are they may be having a lot of soda or a lot of sugar sweetened beverages? And is there a concern of wanting to switch that out with maybe water more so?

Because it does become a little bit more nuanced when we think of things like mention of calcium. Where are they getting that from? If they're getting a lot of it from their milk, and that, it's not concerned in terms of excess sort of calories or that within what's been their beverages. Then maybe. it's a good thing, actually, it’s something encouraged more so based on the other version of Canada's Food Guide.

So, I think it's it adds that challenge between kind of having broader strokes messages, but being able to put one's thinking cap is, what's the context? Where's this person coming from? Is that a point of concern and going from there.

But they're at the point of sugar sweetened beverages and I know that something that didn't really, it’s maybe a bit on Canada's Food Guide but I know there's something sort of wanting to advocate for more within food labelling was the piece of added sugars in our foods we eat. And in particularly when you have a lot of processed food and that, points like that, I think could be really interesting for people to be able to track in terms of what foods they're eating and contributing there. But, I guess, we do have the point in terms of excess sugar, excess sodium fat within foods.

Facilitator: So, just in the sake of time, I'm going to move on to our last section, which will be about features of the application. So, which instructions and supports do you think should be provided to the users to help them an application like this?

Respondent 4: Registered Dietitian.

Respondent 2: For sure is like having that resource page. I think it's also because to me, if I look at an overarching theme right now is the lack of trust in health care and nutrition. And everyone is a nutrition expert, of course. But I think having guidance of why are we telling you that you should not lower your carb to this? Or why are we saying this?

So, I do think that there should be optional that you don't have to read it, but that there is guidance about what is here because I think people need to know that this information is built on evidence, people need to know, it's not sponsored by the industry. I think that is just really, really crucial to build trust, and to show the evidence behind these recommendations.

Respondent 1: Yeah, I think this might be a little obvious. But I mean, if we're going to reference guidelines from the Canada's Food Guide, having a section kind of explaining that going along the lines of what Susan explained, what is a protein? What's the importance? I think just a section on what's Canada’s Food Guide, what does it entails? What are the recommendations?

Definitely a section on dietician, how we can help, how you can access services. And then we mentioned recipes earlier ideas like meal planning strategies. I think, ideally, if we can get all that in the app, I think that would be great. But obviously realizing, I don't know what's realistic.

Facilitator: We’re taking all the ideas now. We’ll determine what's realistic later, for sure. So, which features do you think could help with adherence? So, helping people stick to using an application such as this one?

Respondent 3: This maybe goes beyond Canada's Food Guide, but I think it's unfortunately, what Canada's Food Guide and that approach – the timely, the hot topics that are really in the media in that and being able to combat misinformation, because the process of updating Canada's Food Guide, it's very slow and cumbersome.

And by the time we get it out, there's a number of other points that are coming up in terms of the media and that. And I think supports around that piece within an app in terms of reliable sources of information and reflecting on sort of, within knowledge translation, mobilization within the latest research within nutrition, I think that really attracts a lot of people and there's a lot of interest there.

But in terms of good reliable sources of interpreting the nutrition research and that, I think that piece could really attract people with that was supported as well.

Respondent 2: I think from talking about adherence is that managing guilt is really crucial, because for me, why people stop tracking is they start to feel guilty. People track when they're doing well, they stop the minute they don't feel like they're doing well. That piece needs to be addressed. It shouldn't be about eliciting feelings of guilt, that's a terrible motivator.

I think that also looking at it, it needs to give people something. So, that's why I look at it as being a meal planning tool. Like having a recipe database, having saved meals in there that you're like, “Oh, I know that this meal I created is going to provide X amount of my nutrients is a really good meal.” I think giving, guidance, personalized feedback that based on someone's eating without guilt and shame.

So, seeing red bars, things like that, even like these are my limits and now I'm going over. Or even being able to say is that changing my goals, I'm going on vacation. Here's a pop up of tips to help you on vacation. So, I think to me, I think the most important thing is that you have to find a way to not elicit guilt in people and to manage that. And that it needs to be, I think Janice was mentioning timely, but also like it has to give you usefulness. You can't go looking for the answers.

And I think this is what a lot of apps fail at is that they spit out this information, but people don't know how to interpret it. So well, why am I even using this? It just makes me feel guilty. But if they can see that, “Oh, did you know that you're eating this much soluble fibre and soluble fibre helps with this, blah, blah, blah?”

So, I think that that's what I see is being like critical for helping people with long term adherence or even just adherence to the point of improving their diets.

Respondent 1: I agree with what was mentioned, I think the biggest thing for me with regard to this question and the adherence, when I think of like research on behaviour change, and I think that celebration piece in that, which goes similar to Susan, where she's saying not to encourage guilt.

But I think, on the other hand, is having these kind of either kind of reward system, I guess within the app, very similar to – I have the Apple watch myself, and I'm kind of familiar with that. And I've never had any messages be like, “You didn't meet your goals.” It’s more like, “Look at how many times you close this ring”.

So, having the celebration, having that reward, having these challenges, even that come up that keep people coming back. And I think that's one thing that the app could give to the person. We don't always take the time to be like, “Yay, I ate my vegetables.”

But I think by having that, it really helps to reinforce the behaviour. And having the app do that I think is a lot more natural for a lot of us. So, I think that would be the most important piece, I think for the adherence, in my opinion.

Respondent 4: Just to add to the piece, where I said a registered dietician, because I realized there's only so many of us and quite a large population. So, in terms of how feasible and realistic that is. But I know, and there's mention of this app and developing for self-monitoring where people can be independent and meet their goals and work on themselves.

But I think being able to see these apps also in the opportunity to complement existing services. And I think things such as in BC where we have Health Link, if there's a way that it can be used as a tool and complement some of those public sorts of openly available resources.

And I’ve even seen mentioning how I've used myself, Eat Tracker, as an education within a college class. And maybe within having as a tool and resource within group scenarios and that. And maybe, within Canada's Food Guide, I know often used very much within schools, where people are introduced and use it there.

So, being able to see an app and complementing within use in that context and resources may be for as an educator and how it can be used to do different pedagogical curriculum and lessons and such. So, seeing other ways in terms of being able to work in terms of self-monitoring, and sort of independent but maybe to complement sort of existing supports.

Facilitator: That’s a very interesting point with the education piece, because Canada’s Food Guide is the key vehicle that kids are taught.

Respondent 4: And then that one, you could tackle it in terms of people who are teaching those courses where you’d address Canada’s Food Guide, you train the trainer. They get information, they get also those pieces in terms of maybe body image and in terms of certain aspects, which would be mindful and maybe eating disorders and things like that.

But where the app could support some lessons in school and used in that way. And then if people find it rewarding at that point, they might actually keep on with it and filling rings or what have you and that. So, being able to see ins and opportunities to get exposure and to get people attracted to using it at different touch points.

Facilitator: Very interesting. I realized that our time is up for today, if you need to leave, please feel free. I do have a few more questions blacked out. So, feel free to stay as well if you can. So, the next question is what features are required to ensure accessibility for all users?

Respondent 6: I noticed something when you brought up the, I guess the sample that you have, like that video. In terms of colours, I think for people that are visually impaired or they're so used to green and that kind of reddish pink, I think keeping that in mind. Colours that have good like contrast with each other, but also, not just visually appealing, but just do, I guess, cater to those with maybe some visual impairments.

Respondent 2: And I think, for sure, making sure it's culturally inclusive. So, if you put in that you’re Indigenous or whatever your background is that the foods are reflected, that it's not just North American, Canadianized food. So, I think that is a really, really key feature that must be included, especially from being adherence and acceptance at this time point in is really important.

Facilitator: And with regards to accessibility, with regards to disabilities, so we mentioned colour blindness, visually impaired people. Any other disabilities that you could think of that we could cater to within an application?

Respondent 2: I mean, I would say having like sound is that play like audio features as well. But I think just being as inclusive as possible. I know apps, the development features, like there are abilities to do that. But I think that nutrition information should be inclusive to all. So, finding a way to make sure that we are meeting those features and testing the language and things like that is I just think is going to be – I mean, I'm sure you guys are going to do that anyways.

Facilitator: Are there any other features that you could think of that would be required?

Respondent 6: No, I'd say I agree with everything that was mentioned.

Facilitator: OK, great. So, now I'm just going to go into a summary question. So, is there anything that we haven't mentioned that you think would be useful in creating an app based on the new Canada’s Food Guide?

Respondent 4: What are you going to do when they update the food guide in a couple years and it looks totally different?

Facilitator: Yeah, we'll definitely have to see.

Respondent 4: No, I’m just thinking in terms of sustainability, that's the one thing I think in the back of my mind, because that's what happened to Eat Tracker as well, all the effort that's put into that. So, that's maybe where – yeah, I guess you need a crystal ball to know what Health Canada is thinking and how they might – but that's maybe where some of the bigger ticket items kind of broader stroke messages is they're different features as a selection of different items.

So, if new Canada's Food Guide comes in, you can add on the updated messaging or such. I don't know. Anyways, that's one thing that comes to my mind with things. Good on you and good luck. But I'm thinking when is the next Canada’s Food guide coming out?

Respondent 2: And I think to that point, is that I mean, I don't know if you're able to, but working with Health Canada, I think is critical. Is that seen? And I don't know if it's possible but it shouldn't be – because these apps, they don't really work with the government, they work with their resources, but they're not working directly with the government.

And I think if an app can be endorsed, because I'm sure one thing that I thought about is, does Health Canada have an app? I'm sure they do, like a food guide app, I'm sure. Oh, they don’t yet? OK, because that's the other thing is like, oh my god. Stop repeating the wheel, especially if it's Canada's Food Guide a duplicate. That's something that I think would just be like a waste of resources in timing when we know there's not infinite resources.

So, I think being supported by Health Canada, and I know from the development piece is to Janice's point is that the food guide is supposed to be a work in progress, that it's not supposed to be solidified. It's supposed to be more regularly reviewed. So, I think it's also essential to have an app that is very much in line with that. And I do have to run though. I don't think I have anything else to add, but if there's anything you need to clarify, just send me an email. I'm happy to chat.

Facilitator: Perfect. Great. And if you think of anything else, as well, please don't hesitate to send me an email.

Respondent 2: Absolutely. Have a good day, everyone.

Facilitator: You too.

Respondent 4: Just to add to that piece and thinking in terms of sustainability, and that because it's funny to kind of giggle, “Oh, yeah, Canada’s Food Guide” But I think it's a very real topic in terms of sustainability in that. If the app is seen in terms of smaller, different modules in terms of different topics that people could maybe have added into sort of goals and rings or something like that, if one could have it as modules where you could even think of, I don't know, your celiac and you have something.

Or certain maybe even therapeutic type considerations where they could be different modules on there, but developed and supported by reputable organizations. So, it's seen as maybe kind of, and this maybe is going to be on Canada's Food Guide. But I think that in itself could be helpful as kind of reputable evidence based type guidance and supports within different topics and different goals.

And then as Canada's Food Guide is updated, I don't see things drastically changing like they're totally dropping of, we know what the bigger ticket items would that would stay on but that's where if it's in a module form, you can kind of update certain pieces and add and subtract.

Facilitator: Alright. Again, if you have any other ideas that come up, please don't hesitate to send us an email with anything else that comes up if you think of anything in the future. It was a pleasure to talk to all through this morning. We will be sending the gift card as an appreciation for your participation today to the email that you provided on the questionnaire as well as to the place that you provided as well..

Respondent 2: One last thought that came into mind is I know we have validated tools for nutrition risk for certain conditions and that. So, I think incorporating that within the app, so that's picked up and that if they are at risk, then that they go to a health professional. I think that's really important that we get in an app like that.

Facilitator: Yeah. So, the assessment piece at the beginning and then referring them out if need be. OK, great. Anything else? Perfect. Thank you so much for being here again, and have a great rest of your day.

Respondent 1: Thank you.

Facilitator: Bye.

[End of recorded material]
