# Supplementary material for: Perceptions of self-monitoring dietary intake according to a plate-based approach: A qualitative study
Source: PLoS One. 2023 Nov 28;18(11):e0294652. doi: 10.1371/journal.pone.0294652 (PMC10683993; doi:10.1371/journal.pone.0294652)
Supplement: S5 Appendix — (ZIP) [file pone.0294652.s005.zip › Anonymized RD Focus Groups/Icanplate-rd-focus-group-1.docx]

**Icanplate-rd-focus-group-1**

[Start of recorded material 00:00:00]

Facilitator: So this is the RD focus group one on July 21^st^ at 7pm. So this first part of the focus group will be about behavioral techniques and self monitoring applications. So first off, first question is do you suggest following the plate method as illustrated by the Canadian food guide to your clients?

Participant 1: I’ll start us off. I would say yes in general I do recommend that. I find it, like especially for initial maybe appointments or assessments, it’s a very like broad overview and helps to kind of explain the different types of foods out there as well if you’re starting someone from scratch. Like what’s a protein based food? What’s a whole grain? What’s a carbohydrate? And all those sorts of things.

Facilitator: Mm-hmm. Yeah, Charles what were you going to add in there?

Participant 2: I was just going to say I love it as well. Especially for clients that, as a starting point, like the old food guide was just too complicated, the number of servings on the plate. Everyone has a plate at home, everyone can visualize what half a plate, quarter of a plate looks like so it’s more user friendly overall.

Participant 3: I have two grades, definitely more user friendly and I like the fact that you can really focus on being mindful with the plate and you know going with your hunger versus how much of everything. But in terms of proportions you kind of know what you need to have more of and it gives you lots of different ideas.

Facilitator: Justine I can see you nodding. Did you have anything to add?

Participant 4: I would agree with the previous statements and then I’ll just add that it really depends on the population that I’m working with. For example if it’s like more vulnerable seniors and they’re eating poorly, but the focus may be different. I don’t think the Canada’s food guide is intended for people with certain chronic illnesses or conditions.

Facilitator: So who would you use it for?

Participant 4: For the healthy population in terms of prevention.

Facilitator: Yeah so it’s not necessarily adapted for all the populations that you’ve worked with at the moment?

Participant 4: Yeah.

Facilitator: Cara did you have anything to add on that?

Participant 5: Yeah sure. Before the Canada food guide came out we had used the plate model kind of in diabetes which is always a good starting place because most people of their plate is carbohydrates so it’s a good shift to kind of get people refocused on what you need to do. Yeah, I agree, it doesn’t work for every population especially if someone’s frail or has [unintelligible 00:02:41] they need higher calories, higher protein. But for something like diabetes or heart disease with no other complications, which is rare, or people who are just looking to kind of eat healthier or we’re talking about mindful eating, that’s kind of like a good starting point for them.

Facilitator: And what would make it easier or hard for your clients or patients, I’ll be using clients for this focus group, to eat in accordance to the plate method or the new food guide?

Participant 2: So I’ll jump in on this and coming from working in long term like, even though I like the plate model, it’s terrible from an institutional standpoint because it’s just not physically possible to balance out all those vegetables within a budget especially when we’re aiming for fresh and frozen. Like we’re aiming right now mainly for frozen. A lot of my residents struggle eating more vegetables.

They just tend to be very heavy on the meat and potatoes. So even though it’s a good idea, the older population grew up eating meat and potatoes and it’s really hard for them to stray away from that at this time when they’re going to be, like it’s more enjoyment, I think it’s more comfort [unintelligible 00:03:57] provide.

Facilitator: So from an institutional perspective, having that half plate of vegetables or fruits and vegetables isn’t necessarily appropriate?

Participant 2: I think for a lot of people that I’ve talked to, either finances is a consideration although there’s ways you can work around that with vegetables, but people still feel that that’s a really big barrier. Or they don’t know how to cook vegetables or they don’t like the taste and those are kind of the biggest barriers or as everyone was saying, it’s like the older mindset of we have lots of meat because of you know that’s the more expensive thing to have and if we can afford it we can do it. So I think just that mind shift, that’s a cultural thing too, is really hard for a lot of people.

Participant 1: I would have to agree with that and being with populations that can, majority of the time, afford fruits and vegetables, even from my standpoint like I buy a ton of fruits and vegetables and it’s ridiculously expensive. It’s the prices are just climbing and climbing. So I find it hard even for myself as a family of four to have that many. I feel like I’m constantly going out to buy more and more to keep up to give everybody enough fruits and vegetables, for sure. But on the other side, if we are promoting more of a plant based diet then that’s kind of balancing I guess, a little bit more. So that’s good.

Participant 3: I’ve found it’s been hard to get people in the mindset of not thinking about serving sizes necessarily. Like there’s always been those bench marks that you need 5 to 7 servings in this category as opposed to now it’s more about proportions.

Facilitator: Definitely save that for further on in the focus group because that’s the challenges that we’re finding with creating an app based on this new plate method and why we’re here today. Charles, did you have something to add on that?

Participant 2: Yeah I was just going to say just with regards to that [unintelligible 00:05:56] points, working in food distribution as well like cost of vegetables just sky rocketing right now, industry average is we’re seeing a nine percent increase in pricing just over the last year. But even within clients that I used to work at my previous job they have their very small set of vegetables so it’s hard to, like they’re eating the same ones over and over again. It’s hard to get them to expand outwards. Working the grocery store, great way to just help them expand it, but not everyone has those resources. And I’d like to throw in the cultural piece. I think with the time we’re in right now having the cultural appropriate vegetables like Chinese vegetables and all that is going to be really essential so that everyone could connect to the plate method even more.

Facilitator: Yeah definitely. [Tanie? 00:06:47] did you have anything to add on that?

Participant 4: I think those are all good valid points for sure and I think that it’ll be interesting to see what happens kind of with the plate method.

Facilitator: Great. I’ll go the next question which is which diet tracking applications do you use with your clients at the moment? Or diet tracking methods?

Participant 3: I don’t usually use any because I found the technology literacy of people is pretty low and even like during COVID when we couldn’t see people in person I would say nine out of ten people didn’t even want to talk to me on Zoom. They were like no, that is way too difficult. So that’s not even an option to broach that conversation with that.

And the people that kind of do use them already when they come to me, I want them not to use it because they’re focusing too much and they’ll use like My Fitness Pal and track too many calories or they’re really looking at all these little things and it’s almost to a point where I’m worried about orthorexia at that point and I want them to kind of look more broadly than being so focused on macro and micro nutrients so then I don’t want them to use an app.

Participant 4: I have to agree with the previous statement as well. I used to use apps with my clients a lot like My Fitness Pal and I found the same thing, there’s way too much focus on calories, way too much focus – I mean once you go into the app for example it already gives you like for some reason everybody’s on 1200 calories, it’s like always the same thing for everybody.

And I find, yeah there’s such a focus for people to make sure that they’re getting either that or I had a ton of clients trying to get like under that because they think that that’s the goal and I think it just creates a ton of mixed messaging so I think that’s a huge challenge.

Participant 5: I would agree and I think one of the things that I’ve done is kind of created my own, either electronic templates or paper formats for clients as more of a habit tracker so if someone wants to focus on getting you know more water in per day, if they want to focus on having two fruits and three vegetables a day or one serving of beans and legumes, like they can kind of follow that and track that for a month or so. I’ve also had people come to me where they’re tracking every single little thing they eat on My Fitness Pal and going more to the extreme with that so kind of a little bit of re-education there as well.

Facilitator: I’m seeing all of the negative points right now about those current apps on the market. Are there anything that it’s positive or makes it easy for your clients to use tracking methods or tracking applications?

Participant 2: I want to say that the positive is like when sometimes they reflect back on it and they realize that yeah I can make these changes. Working at the grocery story, I had a lot of people that were using paper based applications and it is a good way for them to recognize it and be more mindful of what they’re doing which can be both positive and negative at the end of the day.

I do think that for tracking purposes there is high levels of inaccuracy no matter what you use. And I have worked with some companies that are looking at more AI and using your camera to determine how much you’re eating. But it’s in more of an institutional stand point since it’s more defined.

Facilitator: Yeah very interesting.

Participant 5: The times I have had success with it were kind of people who kind of mindlessly ate and then once they actually started paying attention they were like oh, I didn’t realize I ate that much or I didn’t realize I snacked that much and then even just without talking to me about it again were like I’ve stopped doing that because I realized I didn’t need to be eating that snack because I was just grabbing for it during TV or something like that.

And then I had someone who we had to do a [unintelligible 00:11:08] diet and then just to make sure that they were actually getting enough nutrients it was helpful to see what the breakdown was because they were missing some key nutrients so it was helpful to kind of refocus on exactly what foods they need to continue eating so they weren’t going on the opposite spectrum of malnutrition potentially from doing that diet that was necessary.

Participant 1: Us too actually. I’ve done that as well and it’s been really helpful although it is inaccurate, like Charles said, it’s not perfect. I definitely, it was really helpful to give them more of a visual and say this is what you’re doing, this is where you’re lacking and this is where we can make improvements so it’s a great visual to see especially like the pie chart and all that.

Facilitator: On My Fitness Pal you mean for the macro distributions?

Participant 1: Yeah.

Participant 2: I want to add, like it also gives you a sense of like why you’re eating certain things especially like a lot of my clients were always on the road so like they could sort of reflect on their schedule like, oh I’m eating x, y, z because of this reason and they could explain it to me and you had that level of connection with your client understanding like yeah, sometimes you have to eat out, but teaching them how to eat out healthier or making better choices. Great talking points.

Participant 3: About awareness I think is coming back a lot. Getting clients to realize what they’re doing and what their actions are.

Participant 5: I’ve only recommended like recoding intake and things for very short periods of time, like two days, three days because I think it may sort of build some unhealthy habits if it’s done for an extended period of time.

Facilitator: Alright so I’ll go on to the next question. Thank you guys so much for those insightful answers. So do you know any diet tracking tools, paper or mobile, that current resemble the Canadian food guide or the plate method?

Participant 2: No.

Participant 4: I thought there was one that had like a plate and you could fill out what percentage, but it was still based on, like it had a plate picture, but it was based on the old serving sizes and then if you filled out the serving sizes it would fill out your little pie chart or plate chart. If I recall it was a government one, but they’ve definitely discontinued it.

Facilitator: Yeah there’s one in the states that has people track their servings and then the plate fills up.

Participant 4: Maybe that was it.

Facilitator: That may be what you’re thinking about, yeah

Participant 1: I think I had seen that one as well and I think it was like in the states, but I can’t think of anything that accurately kind of depicts or wants you to track things according to the plate that we currently have.

Facilitator: Yeah so that’s why we’re doing this study and what we’re looking for and why we’re consulting with you guys today. So now I’m going to go on to the second part of the focus group today.

Showing the app’s prototype

Facilitator: Alright, so just a quick overview of the app and what it could look like. What are your thoughts just off the top of your head right now after seeing that?

Participant 1: My immediate question is how do you deal with mixed meals? Like something like a stir fry? Yeah that would be really hard to determine the portions.

Facilitator: Definitely. So anything mixed, stews, stir fries, curries. Anything like that would be difficult to break down into those different portions.

Participant 2: I have a question as well. So is it based on me kind of deciding or thinking how much of a grain? So if I think my plate is a quarter full of grains then I would put that into the app?

Facilitator: Yeah. At this moment, yeah. There is a future question that’s coming on about portion sizes and how that would be recommended so keep that in mind for in a few minutes.

Participant 5: And to clarify, you swipe up or down to adjust the proportions?

Facilitator: Yeah. So it would be on these little buttons here to swipe up.

Participant 5: Oh OK. I can see people who aren’t as tech savvy getting confused if there’s like no arrows to show which way, that would be confusing for people I think.

Participant 1: Or even like a plus or minus button.

Facilitator: So next to here?

Participant 1: Yeah that would be good.

Facilitator: Yeah the little plus and minuses to make it super clear.

Participant 3: I have a question. Why did you pick those specific colors?

Facilitator: It’s based on the colors on the food guide. So we tried to pick colors that would represent the closest to what is currently on the food guide. Do you have any suggestions with regards to that?

Participant 3: No, I’m just curious. The pink is kind of standing out a little bit because it’s not something that you would normally see when you’re looking at you know, like healthy colors for example, green, blue, that kind of thing. So it just kind of stood out a little bit for me.

Facilitator: So something a little bit more blue potentially for the protein?

Participant 3: I mean not necessarily. It just really stood out for me. I don’t know, does anybody else kind of feel that way or is this just like a really weird thing that I noticed?

Participant 4: I would kind of agree too and I think it’s maybe like an interesting shade of pink almost. Like I don’t know, but yeah I thought that a little bit as well.

Participant 5: Yeah so did I.

Participant 4: And it almost isn’t as pigmented as like the green and the orange as well.

Participant 2: I’m used to the old food guides where like grains are just yellow and protein, which is usually meat, is red.

Participant 1: Also can we ask who the target audience would be for this app? Like who is your ideal user or is it going to be for everybody kind of?

Facilitator: For now it is the general population so the general public, usually healthier adults, but not necessarily for people as you mentioned at the beginning with higher protein needs or higher calorie needs or anything like that. That would be the people who want to change their diet and eventually we would see ourselves using it in intervention to help people change their habits.

Participant 2: I think it needs captions. Like just tell me like what to do and [unintelligible 00:17:50] to talk a little bit about the grains. Like I press the grain button and I sort of want it to just sort of explode and then like give me a bar that says like more or less where it’s just two arrows. So it’s just a bit easier especially for individuals that have stubbier fingers. They might have troubles with the method that they’re suggesting right now.

Participant 3: And I guess is it going to like break down to your different days? Like is there going to be an area you can go to so you can kind of go day one, day two or whatever? Like maybe it’ll be July 22^nd^, 23^rd^, 24^th^. And then you’ll have your three plates there. Is that -?

Facilitator: This isn’t something that we’ve decided on yet so why we’re having this focus groups is to touch on those points as well. I’ll stop sharing now, you have this visual in your head and the first question about this app is how would you view the application working to record all meals and snacks throughout the day so things like breakfast, lunch, dinner, snacks?

Participant 1: I guess I’ll just start with I guess I just want like I would want it to be a little bit structured on your day and then you can add meal one, meal two, meal three, snack one, snack two, snack three, whatever you want. But be able to like easily click on like a day and go to that day and then have that screen pop up and then you can label it as like meal one or meal two or whatever, I think would be helpful maybe.

Participant 5: Yeah I like the idea of having the multiple, you can see each meal for the chart so then, especially with the colors, then you can kind of see especially if you’re doing interventions throughout, you might change quite a bit and I feel like that would be like a very rewarding thing to see if it changes throughout for a patient and for us too. But it would be rewarding to see, to get that visual change.

Participant 3: Yeah and I think with the world of like apps now too, data is what people want. So like being able to tell like oh day one I had 50 percent vegetables for my whole day or whatever and then day two oh like 40 percent or what not. And be able to track those trends too I think would be important for people like from meal to meal and day to day.

Facilitator: So maybe like summary circles, like on My Fitness Pal as you were mentioning earlier? Maybe like a daily one, weekly one potentially and they could swipe and compare?

Participant 3: Yes. I like that.

Participant 4: I was also going to say, a lot of my clients are very busy people right? I mean most of us are. So something that might be really interesting too is if there’s a spot where they could take a photo of their plate so that they don’t need to do it right away because I find you know, if you miss one or two meals it’s going to be hard to kind of go back and remember everything.

So if there’d be a spot to kind of take a photo and like oh go back to it, I mean further down the line, like Charles was mentioning, a little bit of AI would be really cool so that it would do it for you, but I know we’re not there yet, but that would be really awesome.

Participant 1: I have a question about the sizing of the plate. You said it can expand. How would that translate into like generating information? I think that would be quite inconsistent if you were to, it’s a large plate or a small plate.

Facilitator: Yeah so the solutions that we’ve had for that were two different sizes with plates, but again we’re open to feedback on that. So having smaller plates for example, for snacks and for breakfast and then larger plate if they’re having fuller meals. How would you see that working?

Participant 1: It seems like that might be confusing in terms of, like if the overall intention is about proportion of the three groups. I don’t know. I’m not sure if that would be useful.

Participant 2: I feel like more maybe even labeling like snack size and then small meal, medium or large meal or something like that would be maybe a way around that. I don’t know.

Participant 5: Yeah like ticking a box like did you use a large plate or a small plate or was this a snack or was this a meal?

Participant 1: Yeah almost like a prompt before you even put in your information. Like maybe there’s a couple of prompts before you put in your information and you automatically like just click on what size and whatever else. You can add a few things if you wanted to.

Participant 3: I love that.

Participant 5: I feel like snacks are hard to record sometimes because not everyone does the three food groups for a snack and then I don’t know if you have that visual or not having one of the colors, if that was, it’s kind of almost like a negative thing. You know like oh I didn’t get that balance even though with snacks you don’t necessarily need to get that balance. And I thought that would be kind of a negative experience for people.

Participant 1: Yeah and it might be hard for them to categorize like if they’re having a granola bar or a cookie or a muffin or something like that. A lot of people might go oh no, where do I put that right?

Facilitator: Yeah definitely save that for later. That’s a question we’re getting to for sure. Alright, anything about breakfast that may be similar to the snacks with less of the food groups present as well?

Participant 4: Just thinking for breakfast we haven’t covered dairy at all like yoghurt, smoothies, those sorts of things.

Facilitator: Yeah. We’re definitely getting that too for sure. You’re getting ahead of me. So next –

Participant 5: I think that’s where if, like we were talking about having those daily summaries in the proportions and the daily summary, that would be nice because yeah, as you said more people tend to eat vegetables at lunch or dinner and not as much as breakfast, but if you do a daily summary it might equal out which is really the whole goal for looking at holistic things, not a meal to meal, but maybe more of a day to day?

Participant 3: I think that the comment about milk really illustrates the need to also describe what are in those three categories. Like protein foods, like that’s maybe a new term for some people.

Participant 1: It could almost be a prompt too like when you’re putting in whatever you’re putting in. Like you can choose your meal size and then you can have a list of foods and you choose yeah, I had a bread or I had this or I had that and then it can automatically get placed into a group, maybe.

Participant 4: That’s actually what I was thinking too more along the lines of if there’s a list of food and that way they’re also learning as they go. Like OK this is a protein food so like under the category it’s exactly like the previous comment and you pick your food and it would automatically put it in to like protein or something fun that would like top it.

Participant 2: The only concern I would have for that is like does it almost seem like a chore? Like we’re going to do a mixed dish let’s say. You’re going to be doing a lot of searching if it’s like a stir fry with [unintelligible 00:25:09] and vegetables. Especially like, that’s where I think like the photo, maybe that’s where the photo comes in. You take the photo, you do it later and then you can input that information.

Participant 1: Yeah that’s true.

Participant 3: Yeah and maybe even having like a search category. So instead of scrolling through a list of foods, if you type in CH, chicken will pop up. Or something like that might be helpful to make it faster for people.

Participant 2: My concern is also like what about what we don’t include? It’s like the cultural food component. Like there’s just so many foods especially within Canada we import in. So it might create some barriers in terms of just [unintelligible 00:25:56] all cultures.

Participant 5: What about the fact that a lot of people don’t eat from plates? Like some cultures eat out of bowls and depicting more of a like a 3D image or [unintelligible 00:26:10] like layered or not sitting flat on a plate. The plate method doesn’t sort of work for everybody visually.

Participant 2: That’s true.

Facilitator: And that brings us to our next question which is how do you suggest these digital applications represent their meals portion size on the plate? So translating that 3D image to a flat plate.

Participant 5: I feel like that’s what I struggle with sometimes when telling people the plate model because then they’ll be like OK I can do that I’ll just pile my carbohydrates this high on the plate. And then that’s a quarter right. And yeah people will say that a lot. But yeah, I can of go back to kind of like it’s still your overall portion size when you’re look at it in the 3D image. What is your percentage compared to the other pieces?

But I feel like that’s a hard one for people who aren’t with a dietician and just looking at the Canadian food guide is kind of how to, how do you know that? Like we know that, but if you have a low level of health literacy, how would you know that without having extra education on it which I think is a barrier? And the bowl thing too.

Participant 2: I do like the old school hand model because back when I was teaching kids, like the hand model, everyone has a hand and like it is proportional usually to what you eat. You have a larger hand you’re probably going to eat more. So just like having queues of like alright you’re going to have a grain, do you have a pop up maybe of like what each grain sort of looks like? And then like what a portion might look like around [unintelligible 00:27:46]?

Participant 1: Yeah I like that idea of the pop ups with the portion sizes with the hand model.

Participant 3: But using portion sizes would be in order to generate some sort of information about like calories or like what would be the point of using, defining the serving sizes in the model in terms of the app? Is there a reason to do that? Because if it’s just about how much of your meal is [unintelligible 00:28:25] the –

Participant 2: I just think people have a larger, like a huge problem quantifying it. Like you mentioned like Chinese culture. You’re getting rice in your bowl and everything you’re just grabbing. So it’s hard to quantify that on a plate overall, but then like I can sort of estimate like oh that’s not a fist worth of vegetables I ate with my rice.

Facilitator: Yeah so we actually did the plate method of tracking on paper with our participants and the portion sizes was something that came up a lot because people want to put it and want to be as accurate as possible with their proportions on their plate so that’s why the portion size was coming up. So the first idea we had was with regards to the hand method. Are there any other ideas that you have with regards to how to place and how to decide how much of the plate should be filled with each group?

Participant 5: Sometimes I tell people like if they’re eating stuff a bowl which is super hard to do, [unintelligible 00:29:26] said take a look at the raw ingredients that they’re doing so if they’re cutting up so many vegetables, how does that size compare to the meat and compare to how much grain they have. And sometimes that helps and sometimes it doesn’t but it’s still regardless really hard to do. The hand one I like. The hand model.

Participant 1: The only thing with the hand model for me is that you know we obviously all have different size hands so how does that represent? Like does that change in the app depending on like questions at the beginning or is everybody, you know one handful the same for everybody kind of thing?

Facilitator: That’s a great point and I was thinking actually like at the beginning of the app maybe people should be setting up their profile or whatever and then they can kind of add that information and I was even thinking that’s when they could choose maybe if they want to use a plate or if they’re prompted do you eat most meals from a plate or a bowl or like another method? They could also add that in as well.

Participant 3: I think the CDA used to use some standardized objects like a tennis ball and a deck of cards to do like two and a half ounces of protein or some of those things which are pretty common objects that people may be able to reference could be useful.

Participant 4: Yeah some sort of education in the app where it’s like yeah your fist is a quarter plate grains or however you quantify to translate it, but the app is translating it for the person if that makes sense? So if they have that baseline education as in a quarter of your plate grain is roughly equal to your fist and then people can easily be like oh yeah, I ate way more than a fist or yeah I ate a fist and then they would know that 25 percent or that’s actually a little bit more or that’s 50 percent. So it gets messy too.

Facilitator: Yeah so building on that our next question is what would be considered a successful meal or a successful day? Or what should their goals be with regards to their plate?

Participant 1: I would hope that maybe that could offer like maybe picking a couple of the goals in Canada’s food guide to achieve all of the principles are maybe unrealistic for some people so maybe we could identify some that you’re tracking whether it’s water or working on the proportion.

Participant 3: I was thinking maybe it could even have like goals that popped up. Like if you ate from home or something like that, two out of your three meals, if that was one of the prompts maybe before you put your food in. Is this take out or is this from home and then that popped up like oh you ate all your meals at home today, you cooked for yourself or something like that. That could be something that, a goal that automatically pops up or you ate plant based today by eating chickpeas or whatever it might be.

Participant 4: Do you think it’d be possible that everybody’s success would be different depending on how you set up your profile at the beginning? Because recently I tried out the Noom app because they boasted all this psychology and I wanted to kind of see what that was about and they asked a ton of questions beforehand very much related to what my personal goals were and I really like that. So if you know, someone has one specific goal then that could be more, their success for that day if that makes sense?

Facilitator: Charles, you were going to add something?

Participant 2: Oh yeah I was just going to say like the home versus take out, I just don’t want people to feel guilty. I’m afraid like if we have the word take out people are going to say like yeah, I don’t want to input that in because there is a little bit of guilt with that when we start labeling it as like home or take out, even if it’s healthy I think.

Participant 3: I guess positive feedback is always good like even if it’s not towards keeping your goals. It could just be that, oh you drank more water, good job. Those kind of things are nice.

Participant 4: Even if you are having take out though, just as an example, if you’re having half a plate of vegetables with your take out, like that’s pretty good.

Participant 5: That’s what I was going to kind of add too, most people that I talk to at least the eating enough vegetables and fruits is like the biggest issue that people have and then if we’re talking about disease management or weight and everything like that it’s kind of connected to your vegetable intake or at least fibre. So a successful day would really be for I feel like for a lot of people who would want to use this app is getting those proportions right and it really comes down to having half your plate of fruits and vegetables, but I would do it as a whole day not as an each meal thing because that can be discouraging or ends a lot of tracking too.

Participant 2: I’m just thinking like can we create something like a checklist for the day because like people have that satisfaction of just like crossing things out and then like you cross everything out it is, by definition, a successful day, but it makes you mindful when you wake up in the morning, alright, what goals do I want to set today that relate to the larger goal?

Facilitator: So coming back to those individualized goal setting for each user of the app?

Participant 2: Yeah. Tie it back in. When you’re reminded every single day what your goal is then you ask yourself is this action assisting me in reaching my goal? If I say I’m going to drink more water like alright, am I going to set a schedule where it says like alright, I’m going to drink water or I’ll finish a bottle of water at breakfast, lunch and dinner and then just cross it off as I finish it.

Participant 3: Some sort of rewards are always good whether you achieve something or like in BC we have the carrot rewards which is like a BC Ministry of Health thing and you would get points that you could spend at the store if you did healthy behaviors. And I think it’s been pretty successful, but ultimately it was because you got a monetary reward that translated to something.

Facilitator: Yeah very interesting. So now kind of touching on some of the things that you’ve said, many other foods are not shown on the Canada’s food guide. So which foods would you think your clients would find difficult to represent on the plate?

Participant 4: Soup.

Participant 2: I’m just thinking of like tofu pudding. What would you consider that as? Like it’s heavy in protein, but like I don’t know. Even dessert, what do you guys consider pudding as. It doesn’t fit into any of the categories.

Participant 1: Treat. Treats and extras for sure. I feel like if I was to eat a cookie I wouldn’t know where to put that on the plate right. I think a lot of people would.

Participant 5: It’s little things like oils you know, not from a “bad standpoint” but getting more healthy fats in the diet, extra fats, not coming from food.

Participant 3: Liquid, like smoothies, things where it’s a balanced meal and a beverage, but how do you [unintelligible 00:37:11] them in terms of proportions?

Participant 2: I’m just thinking for like the healthier individual that works out, how do you represent protein powder because that’s going to be humungous. And supplements, any type of supplements.

Participant 1: Yeah and that’s why I think again the individual goals I think would be great because like some people who are younger who are more athletic who, you know some people I’ve seen I want them to have their plate in threes because they just need those extra calories or nutrients based on their training or what they’re doing sometimes.

Facilitator: So I’m hearing a few categories that are coming up so those may be sweets, treats, more grain based desserts coming up and then the fats, the added fats, things like that. You’re also thinking of things like condiments, ketchup, mustard, relish, seasonings. So if you’re having someone with sodium or hypertension how would salt for example, or added salt be represented on the plate

Participant 1: And dairy too.

Facilitator: Yeah. Yeah dairy’s our next question. We’ll get there. Do you think of anything else?

Participant 5: I feel like once you get into asking or wanting to track those smaller things, then we’re getting to the point where it’s like OK then it’s almost getting similar to the My Fitness Pal thing where it’s so specific that we’re tracking too much and I feel like that defeats the purpose of doing the plate model.

Participant 3: So how simple and how complicated are you planning on making the app? Is it really just the three groups or is all that other stuff being taken into consideration?

Facilitator: That really depends on your feedback so that’s why we’re probing dieticians right now. We also have focus groups in the general public to get their perspectives on that. As dieticians, what do you think would be beneficial for people to track that isn’t on the plate at the moment?

Participant 2: I think mainly deserts because I reflect back on the 80/20. That 20 percent is going to represent 80 percent of the stuff that is going to contribute to the individual’s health and like if that 80 percent is healthy then like I don’t mind that last 20 percent being junk food as long as it’s kept in moderation.

Participant 5: I feel like if you’re recording snacks you could almost do dessert in snacks maybe? And then if it was a summary of your day for your plate model and then if you’re eating snacks like potato chips and things like that that maybe the app would categorize as carbs or maybe you would put it in as carbs, then you’re showing that your daily proportion is way out of whack.

So I feel like that would be beneficial because then people can see like oh I thought I ate 50 percent vegetables at each meal and I thought I was doing really good, but then when they look at the daily total they’re actually seeing oh those snacks actually really add up and really skew the plate model.

Facilitator: Yeah that’s a really good point.

Participant 1: Food guide makes a comment about like ultra and highly processed foods right? That might, I don’t know if having a category for that [unintelligible 00:40:58]. That’s kind of hard. I mean if it’s something that does have something of higher nutritional value like vegetables in it, but it’s still highly processed.

Participant 3: Or even protein [unintelligible 00:41:08] could be considered a highly processed food.

Participant 4: I honestly think though the simpler the better because once it starts getting into all the things that we mentioned which we would love as dieticians to be there, I think people are just going to use it and think it’s great for a week and then just be done with it. So I think if it’s exactly the way that the food guide is so you have your three groups plus your water and then maybe like even just some other category because I think it’s really important to put a positive spin on it as opposed to a negative spin on it right?

So if you’re adding fruits and vegetables and your plate is going up and up and you see it going you know, half your plate, that’s great, good for you. And it’s not necessarily a negative thing to be going into the other group, but the other group isn’t contributing to the healthy stuff that’s supposed to go into your plate.

Participant 5: Yeah I like that idea because then you’re seeing your daily total if you’re including other deserts and stuff that’s like a not color maybe then it’s showing you how much of that you’re eating and it could be like a really eye opening experience for people.

Facilitator: So kind of lumping everything together into other foods and having that on the plate or as a separate category?

Participant 1: I would say separate category, but in a way where it’s not negative and it doesn’t you know, like it doesn’t make you feel bad about it, it just doesn’t contribute to the really good stuff that you could get out of you plate. So like the goal or the success, like we were saying before, should be like a really good balanced plate. I think maybe there would have to be like an education piece on the other. I’m not sure how to swing that part, but yeah.

Participant 3: Is there anyway to build this app to target people of different levels of literacy around nutrition? So if like having more messaging, basic messaging for someone who you know doesn’t have maybe the same level of knowledge but have more messaging for someone who’s a bit more knowledgeable, higher food literacy, understanding and the messages kind of would change. Because I think if it was a dietician using it they would probably find that this would be quite basic, but it may be appropriate for someone who needs sort of first tool.

Participant 1: I like that idea because I’m thinking like that could be built into your profile once you set it up, but also too like I’m thinking most of the people who, a lot of people who come to see me are a little bit older and maybe don’t love technology as much as I do or as much as people who are more tech savvy so you can almost build it that way too.

Participant 4: So have maybe multiple different profiles for people based on the initial maybe screening or goal setting? Include different prompts or indices relating to them.

Participant 5: It could be like a setting that you turn on and off. Like a setting being like do you want full prompts or something like that. Or do you want to turn the prompts off and then people can change it too as they move through the app and if they use it a long time and they get used to it and they know, then they could turn it off later.

Participant 4: I think the more that we talk about this the more I’m thinking like this really needs to be a lot more simple. Like the more we’re talking I’m like oh yeah, OK. It really does need to be very simple and maybe like to keep people interested it would be, there would be like little education pieces, like little things that pop up every now and again like I don’t know, did you know? Or whatever. Something like that.

But also I think it’s important that you know the general population, we’re using this for the general population and that’s great, but you know we’re also here to help people a little bit further and to dive a little bit deeper, that’s exactly what we’re here for. So you know, I don’t want to give them too much either, do you know what I mean?

Facilitator: Yeah so having the app as simple as possible and us coming in to fill in those gaps if need be.

Participant 4: Yeah. I mean I think people are so different and there’s just so many different factors that I think in order for it, if it is very complicated I think that’s where a professional really needs to come in and help somebody right? Or else it’s just like way to complicated.

Facilitator: Alright so I am going to move along because we are coming up at the hour mark now, so how would you suggest beverages be tracked in the app?

Participant 2: Separate category.

Participant 1: That’s a challenging one because milk, if it’s cows milk it could be a protein food, but if it was a lower protein it would be more like water.

Participant 2: My fear is just like if they put the almond milk let’s say, along with the beverages and they think it’s the same equivalent to dairy because I’ve seen it several times.

Participant 1: If the goal is just to consume you know, beverages that are not sweetened then it’s ultimately just to get hydration then just like something really simple to reach your total volume that’s acceptable for [unintelligible 00:46:50] if that’s all that’s needed.

Participant 2: I was just going to say like just have a beverage drinking, put a volume, put what it is because then that’s useful information later on. It doesn’t have to be part of the plate per se because you have beverages without food all the time. But it just, just having that pop up just makes it a lot faster and a lot easier for them to input at least.

Participant 3: The big point of the new Canada’s food guide is to push water right? So I think water definitely needs to be tracked and that’s easy. Things like smoothies, obviously it depends what type of milk that you put in so I don’t know if there could be a small education piece on that and then everything else other if it’s not contributing to the plate.

Participant 2: I’ll say a lot of people don’t like drinking water though, they don’t like the taste of water, I’ve heard it several times.

Participant 3: That’s fair and that would be great for like a tip to pop up, “Have you tried -?” Or something.

Participant 5: If we’re looking to keep it simple it would almost be like tracking the water or hydration separately, but having it as hydrating fluids and then just not even tracking the other stuff and are you getting enough hydrating fluids because I feel like people I have who drink pop all the time, that’s in place of water so you’re not going to hit if you’re just filling up these hydrations, hydrating fluid goal, you’re not going to hit that because that’s not included in the hydration. If it had just like a little question mark and you could pop it open to be like a hydrating fluid includes this and this and then just don’t record the other stuff to keep it simple.

Facilitator: Should things like sugar sweetened beverages be included in other foods?

Participant 5: Yes.

Participant 1: I think so, for sure.

Participant 3: And I think like to keep it simple and I think with maybe what people might want to see is having those categories, protein, fruits and veggies, carbs and then other and then having percentages based on just those items. I don’t know. I think that would help keep it simple and be cover all your bases almost.

Facilitator: I’m coming back to that dairy piece. How do you suggest that milk, specifically cows milk and soy milk, be tracked?

Participant 4: To me as a protein.

Participant 5: Is there a picture of milk on the Canada food guide as a protein or is it yoghurt? I can’t even remember.

Facilitator: It’s yoghurt.

Participant 5: It’s yoghurt, OK. They should have added cows milk to the picture.

Participant 2: I agree with protein only because like if we’re going to be honest, when we have milk and soy milk it’s usually with a meal, you don’t usually just drink soy milk by itself. It could be the snack as well, but I can’t see it being just like something you have by yourself, that’s all.

Participant 1: I agree with that and for example if I’m making a meal for my children and I want to get like a protein or some type of a fat in for them you know that might be it, that’s what they’re getting. It’s going to be some type of dairy product.

Facilitator: And how would a liquid be proportioned on the plate?

Participant 2: A fist. I’m just thinking it’s 250 right there.

Facilitator: So one fist would be the quarter?

Participant 2: Technically it’s 250 millilitres, so yeah.

Participant 5: Yeah I like that idea.

Participant 2: Because even with my south east Asian population within long term care, like when talking to them about how they get their protein sort of following the Canada’s food guide, they talk about using yoghurt and like lentil based chipotle and all that. So even though the chipotle would be more of a grain like you’re getting your protein in.

Participant 3: Yeah and the more that I think about it too, I think especially for vegetarians, I think they use dairy products a lot, some people to get protein as well so I think that would be kind of good to be captured there.

Participant 5: So it’s like coffee based beverages, a lot of people consume lattes and things like that or a snack that would be [unintelligible 00:51:24].

Facilitator: Yeah so maybe having the milk from there as a source of protein?

Participant 1: I think with all the new milks in the market now though that’s probably the hardest piece that’s going to, like where do you fit that? Where do you put that?

Participant 2: I think that’s a great area of education, having that little pop up window.

Participant 5: Yeah I still see that as in like if there’s a description of protein and listing the proteins and listing cows milk, soy milk as part of the protein and then if you’re filing out your hydrating fluids and say you counted almond milk or another milk as a hydrating fluid then that would be enough to be like oh that’s actually not a protein, it’s a fluid and they would be separate, but it’s simple enough too without going into too much detail and really categorizing things in too much depth and making it too complicated.

Participant 4: Basically there’d be hydrating fluids, sorry I’m just trying to understand. Hydrating fluids and then soy and cows milk would go into protein and then everything else would go into other? Is that correct? Does that make sense?

Participant 5: That’s how I would see it to keep it as simple as possible without going into the realm of like what My Fitness Pal is already doing it where it’s recording everything. Yeah.

Participant 4: So things like coffee and tea would go into hydrating fluids?

Participant 5: I think it depends on the coffee. [Unintelligible 00:52:59] what you put into it, but tea I would say would be a hydrating fluid.

Participant 1: Yeah that’s a tricky one too because then people are going to be like oh I can just drink coffee all day, this is good, right?

Participant 5: Yeah. If we were going basic simple I would probably just put that as an other one instead. Because if I’m telling either in the hospital or just in general and they need to get fluids and I’m like no, your coffee does not count because it’s going to dehydrate you. If it has caffeine that does not count as your fluid. So yeah I could see that just being as an other category.

Participant 2: I think it’s going to confuse people though in all honestly. I think we’re getting really complex and like for simple stuff I would just put it as beverage because if we’re going to say, it’s almost equivalent to tea in terms of like what it’s doing, you’re going to get dehydrated because of it, but I think the average person is just going to put it in beverages.

Participant 3: I would look at it more as like, like the positive aspect again like pushing the water if water is the thing that we’re trying to get Canadians to get more of, that’s a good, that’s what we should be doing. That should be like the fluid of choice I guess kind of thing. But I see what you’re saying, like where does the stuff go if somebody is drinking it. So I don’t know. I don’t know if it’s like the lazy way, but in other categories seems really good.

Participant 2: Or would be a prompt for saying like this actually belongs in other even though you have it in beverages just to educate them on the app or something like that.

Participant 1: The recommendations, four cups a day or something.

Participant 4: The goal shouldn’t be like making people feel bad about having coffee.

Participant 3: But I like the recommendation can be the max four cups a day. Just because some people might not know and I think that is an education piece. Someone might be like oh my goodness, I shouldn’t drink eight cups of coffee a day. Like it honestly might get some people there.

Participant 4: But then you’re looking at different populations. Like look at a pregnant woman for example, if you’re putting you know, four cups of coffee that’s too much.

Facilitator: And that’s coming back to personalizing it based on the person. Alright, so I’m going to move on to the next section just being mindful of the time. Now this is going to be more about the features of the proposed application. So you’ve already touched a little bit about it, but which instructions support to be provided to the users to support their use of the application?

Participant 3: I don’t know if this fits in this category, but I do really like the idea of a profile and maybe we can make it pretty basic like your age, whether you’re pregnant or lactating, what your goals are, those sorts of things. And I think keeping it really simple and having clear instructions or clear buttons would be good too.

Participant 4: I really like the idea of having a support chat maybe done firstly by AI where you type in a question and you know it’s kind of like a frequently asked question and something comes up and then if your question isn’t answered you know there’ll be a dietician on hand or a coach. Probably a dietician would be better, if possible, would be there to help you.

Participant 2: I’m thinking just captions, audio, video, prompts. Like video games where it tells you like oh hit here, just so like it’s more visual and like it’s more interactive for the individual because whenever we’re using new software or anything especially if it’s new it prompts us to go to certain directions so people are familiar with it already.

Participant 1: Yeah and you could even put out like a really good You Tube video or some kind of like educational video somewhere for showing how to use the app as well.

Participant 5: I support like in keeping it simple. Yeah I think little blurbs for one of the food categories and if we’re coding like beverages what’s the hydrating beverage or something like that, but simple to give examples. And then I feel like if people are in that much doubt being like what this specific food and this specific category and they’re taking that much care to be really specific about it, they’re going to be willing to move on from the app.

So then if they went to the listener like actually I still need more information and the bottom there was like a link to a webpage that directed you to the exact thing you kind of wanted to know, like if you’re like I wanted to learn more about proteins and then it would lead you to something and link you to a website or additional resources. But yeah I feel like if you’re looking for that already you’re going to be willing to go that extra step to click a link at the bottom and find more information.

Participant 1: Yeah links are a great idea.

Participant 2: I like the idea of just like typing in a food and then it tells you where it sort of belongs. Like going back to the coffee thing, like alright it should go in this category so sort of like a master list just so it makes it easier for people not to guess and struggle.

Participant 1: I love that too, but what if there is a food that isn’t there? Then what? Maybe there could be like a “Your food’s not here? Let us know.”

Participant 3: Maybe it’d be helpful for like different profiles if like a parent or a caregiver wants to track like their child’s intake, in some way they could have a profile for their child. And then maybe some way to like share the results with your care provider. Like if you have a primary care physician or a dietician you’re working with you could email them so that you can, it can be used as like a learning tool in a health [unintelligible 00:59:26] setting.

Participant 4: Yes times ten. I love what you just said.

Participant 5: Yeah I think My Fitness Pal lets you kind of do a summary and a breakdown and you have like a pdf or something because I’ve had clients sent that to me and then [unintelligible 00:59:42] together on people we needed to do that for and that was helpful to have that data in front of me when we were both looking at it. So someone holding your phone and being like OK this is what it shows and stuff like that. So yeah.

Participant 4: I really like the idea of a family profile. That’s awesome.

Participant 1: This is intended for adults right? Not for kids or teenagers?

Facilitator: At the moment adults. But good ideas. We may keep that in mind for the future for sure.

Participant 1: But if it was an adult doing it for their child, that would make sense. But a child like self monitoring for themselves, that’s not the intent of it.

Facilitator: No, definitely not at the moment. So coming back I heard a master list with all the foods that would fit in each category to help people be able to place things properly on the plate. [Unintelligible 01:00:50] users feedback so being able to write little blurbs to developers or a dietician, being able to change things or adjust things. And we also have the tutorials that would come up, either video or within the app itself. Anything else to add on that and anything that could support the users?

Participant 5: I said having like additional links if the information provided in the little info sections wasn’t enough.

Facilitator: So like a learn more, click here?

Participant 5: Yeah.

Participant 3: Who would be the owner of this app? Because I mean in BC we have Health Link BC which has dieticians, which provides telephone based support so there could be a good link to that as like a follow up because it’s a free service. I know Ontario has similar or they had a similar service. Those are provincial resources.

Facilitator: Yeah. So UBC will be the primary owners and the application at the moment, but it will be non for profit. So it could be linked to government eventually.

Participant 5: [Unintelligible 01:02:09] have a blurb and at some point you’re like if you’re looking for more information and then it sends you to like Health Link BC or as you were saying Ontario version or give you a province, like a Canada breakdown of the different resource in different provinces.

Facilitator: Yeah.

Participant 1: Even links on how to find a dietician, that might be good. That’s spreading awareness about us too is always good.

Facilitator: And with regards to the portions, so you mentioned the hand method, like would that be something that would be useful to support users as well?

Participant 5: I think so.

Facilitator: A sort of portion guide to help them place foods on the plates?

Participant 4: I think a hand guide is fine because the more that I think about it’s like regardless of how big my fist is it’s my plate right? So I don’t think it needs to be more complicated than that unless I’m missing something.

Participant 5: I think that makes sense and if we show them the hand guides and we’re like your vegetables are supposed to be this, but your [unintelligible 01:03:20] is about to be this. And if we’re saying this is a half and this is a quarter then you know your proportions. If we’re putting it on to a plate you can know that visual and that I feel like translates into 50 percent or 25.

Facilitator: Alright so moving on, so which features do you think could help with adherence? So things like chatting, competitions, gamification, winning things?

Participant 5: I do use the carrot app and it is –

Facilitator: Oh you do.

Participant 5: I do yeah because it’s free points for gas money. So you might as well. And actually you can challenge a friend so I’m such a nerd, me and my dad do this. It’s our activity every week, we challenge each other. So yeah, so that’s really helpful to do and then yeah, having the points connected to something that’s actually useable is quite nice. And I know that yeah they’re doing like I think they do save on food points or petrol [unintelligible 01:04:24] points. Then you can also use your points to enter in a draw for like Amazon gift cards and stuff like that. It keeps me going to have the rewards.

Participant 1: Yeah that’s really smart. That’s really great.

Participant 3: I think that’s a good idea and also being able to like kind of like on Fitbit if you have challenges you can talk to your friends who are in the challenges with you, I think would be cool because I could see so many people sharing recipes or sharing ideas or tips within that. Or even like if I’ve led different group presentations or workshops, people meet within those and they want some way to stay kind of connected until the next group or meetup so I see that as a potential way for those people to stay connected as well.

Participant 4: And also a way to be connected with people that you know would be good. I could see this being really fun for people in a workplace environment with different challenges and things like that and you know workplaces are often very interested in the health aspect so it might be really good to promote to employers.

Participant 1: I think yeah definitely. You could get a lot of employers on board I think with that employee wellness.

Participant 5: I’ve definitely had virtual and in person groups where people talk and you know they’re like oh I need to walk more, do you want to come for a walk because we both need to do the exact same things like that and yeah to see where that app would be helpful for people who meet at like a diabetes class or something like that if they were looking to have kind of they have the similar nutrition goals and they need to work it together. I think yeah, having that camaraderie is really beneficial for actually seeing results and sticking with it.

Participant 4: You know what I’ve seen a lot of too, I’m in so many different parent groups and I see a lot of groups connected to like a Facebook group or something like that. I’m not saying necessarily Facebook, but something where it’s a community group where people ask questions. I think the only problem with that is that anybody answers the questions so like that’s a bit iffy, but if there is a platform where users can get together and ask questions, it’s always nice to hear that you’re supported and someone’s going through the same thing or somebody has the same question or whatever. It’s always nice, but yeah. It would be nice to have a dietician come on to answer some questions too, but I don’t know if that’s going to far.

Participant 3: I find a lot of the apps like [unintelligible 01:07:10] they’re just kind of static, like you lose a little bit, you kind of discover all the functions and then there’s nothing more so I think something to help keep bringing people back whether you have to build in new things as an ongoing process or new content or things to push people to use it and draw them back in, that’s probably something to keep in mind.

Participant 1: Yeah even like maybe as part of an education piece plus part of a way to draw people more in is like recipes that come out and it’ll kind of show you like here’s a recipe for the stir fry and here’s all the ingredients and this is how they come together and this is the proportions and things like that or what not.

Participant 5: In terms of self monitoring withing the app itself, like the carrot app says, it sends you notifications, like if someone’s challenged you it’ll send you notifications or if you haven’t logged on it’s like “Hey, don’t forget to track your steps.” But if there was kind of alerts that remind you like “Hey this app is here, remember to fill it out.” That’s honestly probably the only reason why I remember to use the app for the carrot app. So things like that and then as you said the education and the tips, if it sent a notification to be like “Hey your tip of the week is available now” or something like that.

Facilitator: Alright the next question. Thank you guys, those are great ideas for sure. So which features would be required to ensure accessibility for all different types of users?

Participant 3: I guess maybe like in terms of the technology itself, like the option to use it as an app or like a web based thing. Like if someone doesn’t have a smart phone that they could do this log in through the internet or web page like that might be helpful.

Participant 2: [Unintelligible 01:09:09] accessible, like I was trying to download an app for my parents today and it won’t download because the phone is old there so it has to be able to meet whatever cooking thingamajig android is on right now.

Participant 5: Different languages that could be customized like in your initial set up.

Participant 4: That would be awesome.

Participant 1: Even things like bigger font and keeping it simple. You’ll reach the most amount of people who will be able to user it effectively with that.

Participant 2: I want to say like some sort of audio thing just because people might have different levels of literacy so if it’s verbalized it’s easier.

Participant 3: Maybe some playbacks of different instructions or features.

Participant 1: Colors are always a concern if people have visual difficulties, like if they have colorblindness so they can’t see contrast so maybe consider the colors that are chosen or using pictures instead of colors to represent the foods.

Facilitator: Mm-hmm. So coming back to those colors, what would you guys suggest as the three colors to represent the groups?

Participant 2: Yellow, green, red.

Participant 5: I feel like that’s kind of classic and people associate those food groups with those colors even though the protein one may not always be red, but that’s probably like the most traditional, most recognisable.

Participant 1: I’d be scared to put red with protein because to me red means stop.

Participant 5: That’s true.

Participant 1: Like bad. Red is bad.

Participant 2: I don’t like the brown I can say that for sure. Brown just, it’s not, I’m not a big fan of it.

Participant 1: And the pink for protein is, for me, if you’re vegetarian it’s kind of like [unintelligible 01:11:18]. I don’t like the color.

Participant 2: Yeah that’s true.

Participant 1: It’s not very pleasant.

Participant 4: Even like a different color palate would be really nice. Like a different kind of blue like something really pretty that stands out.

Facilitator: For protein?

Participant 5: Or just doesn’t clash with the other colors because I found like the one example, they kind of clash together. It’s a little bit of an eye sore.

Participant 2: What about orange? It’s like a color that no one really talks about, but a darker orange?

Participant 4: I think orange is a great color.

Participant 3: Like on the plate there’s a diversity of foods right? Like it’s kind of an artistic representation of different foods in the group. Could you just have a picture instead of a color? Have like an image with a variety of foods in that category.

Participant 4: But then how would you split up the actual plate?

Participant 3: Well I guess the image could grow or shrink according to the ratio of the plate.

Participant 2: I like that. And like you can change let’s say for the protein option instead of meat it’s tofu. Or like some sort of like plant based protein so it educates them as well. It’s almost like [Pokémon 01:12:40] just that food.

Facilitator: OK so I think that does the round of that question. So what other features of diet self-monitoring tools could help users that we haven’t addressed yet?

Participant 2: Something about seasonings in all honesty. Like when I was working at grocery stores like vegetables especially how people were explaining it. Like it’s just like [unintelligible 01:13:24] or stir fry, there’s no introduction of ways to make them more sexy and like [unintelligible 01:13:30] the attraction on the plate. Just something to really emphasize on like it doesn’t have to be just bland.

Participant 1: Maybe something we haven’t talked about yet is different colors of fruits and veggies as well. So maybe something that goes along those lines? And variety of foods overall too. Like maybe you’re eating your potatoes, your chicken or beef and your broccoli and that’s all. Like you know that’s most of what you eat. Maybe something that captures variety of foods?

Participant 3: I have to agree with that.

Participant 5: And this new food guide too also has a large focus on kind of like eating with other people and choosing [unintelligible 01:14:20] Like eating your culturally appropriate food because food is not just about nutrition, it’s about an overall healthy lifestyle. So I don’t feel like we touched on some of those other, harder to quantify food guide things, but maybe that could be part of your personalization goal and the other goal options other than the plate model are some of the other more different things like eating with other people and that could be a prompt, like “Did you eat this meal with a person?” Check mark yes or no. But then we go back into the negative thing too. But yeah that back side of the [unintelligible 01:14:59] all those different guides, I don’t feel like we really touched on those.

Participant 2: I like that. Something like I’m just thinking like imagine having like cooking demo where you have everyone on the app gives you that notification that it’s going to be on at this time. Everyone cooks together which is part of the suggestions and like theoretically you could also eat together virtually. And everyone’s been so accustomed to online stuff too. So it’s not out there per se.

Participant 3: Although I think the app sort of has a recommendation around mindfulness right? Like not using technology while you eat and setting aside a time. So I mean it’s kind of [unintelligible 01:15:38] potentially conflicting messaging.

Participant 5: You can always do that, there’s a soccer player in Europe who’s working with food insecurity for children and he started a company and they do a food demo and he gets like a bunch of different soccer stars to like do a food demo with a chef and then they say hey, here’s the recipe, show us how you made the meal. And they just like repost some of the photos of all these little kids and different families doing these meals to help encourage people to eat from home and choose these cheat meals that everyone can afford.

And I thought that was a really cool way to kind of build community. You know like eating together without eating together and it’s like an after effect so then you’re not, you’re being more mindful of your meal because you’re actually doing it instead of focusing, eating on the computer with people so that would take away the technology part.

Participant 4: I think using all the stuff on the back of the food guide would be good to keep users coming back if there’s new stuff that’s popping up all the time and we did talk about challenges and things like that and I love the idea there’s a chef here, Ricardo, who’s done things like that where he’s made this delicious burger and he’s asking children all over to do their own and send pictures. It’s the same thing and I just love that, I think that’s amazing. And it just shows everybody coming together.

It’s exactly like you said, everybody’s kind of together, but we’re not together and it just brings this unity to us and it’s just amazing, but I think that there’s a lot that we can do with that back page and learning about mindfulness and learning about variety and all of that and keeping the plate part very simple. So it’s almost like you can pick you know, like if you don’t really want any of that stuff and you really just want to focus on the plate then it’s very easy and the rest of it is like bonus extra kind of thing. Sorry I’m so dark.

Facilitator: Is there anything else that we didn’t mention that you think would be pertinent?

Participant 3: I think the food guide, it also mentions around like health, like reading food labels, looking at the products that you’re eating so if there’s accompanying tools or things that could support that eventually in the app?

Participant 2: Like when I did that in the grocery store it was more beneficial when we did it live and like had, like even if you had people like show you what their label was, it was more impactful when they had it in front of them and then you walked them through it. So you have the generalized example then you go to the more specifics and then you add the product questions. I think it’s a great way.

There’s the cultural case, since I work in long term care, the cultural piece is like not there there, but I think it is becoming a large part of discussion in Canada is as unique as it is like all our food culture. So I think that also has to be a large part in the foundation of this app.

Facilitator: And what would you recommend for that?

Participant 2: It honestly has to, talking to different cultural background groups plain and simple. Asking them what they’re eating. I would say even, in all honestly I really don’t think people move outside of like 50 foods so like in the top 50 you’re going to have a lot of duplicates among cultures and then you just narrow it down to the [unintelligible 01:19:32] unique.

Participant 5: So I feel like keeping it same, or so simple in terms of like the categories if you opened the blurb about proteins and you’re including like meats, beans, meat alternatives and things like that then you’re kind of, you’re not missing stuff or not including a lot and then if people want to know more like well what’s in that category then there’s like a link at the bottom that will take them to a different page where it’s more in depth. So then you’re kind of a, yeah you’re omitting on purpose to kind of broadly encapture a lot of different cultures.

Participant 4: I like that idea. And maybe the search bar that we were talking about before can help you do that as well like chicken, OK this is silly but chicken, it’s a meat. You know, something like that. I like that idea.

Participant 3: Would there be, I guess like maybe it’s not useful, but maybe dividing, like for someone who’s vegetarian or vegan or like excluding some foods that would never be consumed I guess.

Facilitator: Yeah that could come back to the profile at the beginning for sure. And not suggest chicken to someone who’s vegan.

Participant 2: My only concern with that and this has come from personal experience is like I have a lot of individuals that are Jewish and they don’t eat pork except for bacon. So like I’m just concerned there’s going to be vegetarians that have certain exceptions and then they won’t be included.

Participant 1: I guess you’d have to specify that it’s very strict. Like strict diet.

Participant 5: I feel like just easier than going into all the nuances and the people can look it up of what they, if they’re looking up a specific food they can Google it to figure, or like get led to a link that would have more detail because I’m hoping that no one would be offended if they were vegetarian or vegan and if they clicked on the protein and it said like you know meal alternatives, meats and beans and lentils. Like I don’t think that would be offensive, but I’m not also vegan so I don’t know.

[End of recorded material]
