# Supplementary material for: Perceptions of self-monitoring dietary intake according to a plate-based approach: A qualitative study
Source: PLoS One. 2023 Nov 28;18(11):e0294652. doi: 10.1371/journal.pone.0294652 (PMC10683993; doi:10.1371/journal.pone.0294652)
Supplement: S5 Appendix — (ZIP) [file pone.0294652.s005.zip › Anonymized RD Focus Groups/iCANPlate-RD-Focus-Group-3.docx]

**iCANPlate-RD-Focus-Group-3**

[Start of recorded material]

Interviewer: All right, so this is the RD focus group number three, on July 30, at noon. So first off just a question to break the ice is do you suggest following the plate method, as illustrated by the new Canadian Food Guide to your clients? Or have you suggested in the past?

Participant 1: I do.

Participant 2: I also do both with the inpatients as well as with our patients that I see in the Rehab Centre. Unfortunately, the ICU patients are intubated, and it's a different story but yeah.

Participant 3: I would say probably 75% of the people I see I would at least reference the Food Guide. And then depending on how many times I meet with them get into some education on that.

Participant 4: I do most of the time. I definitely think it's a good base for people to know what they should be eating. So usually, they'll be some session where I'll teach the food guide and kind of apply it to kind of the way they should be eating and their food preferences as well.

Interviewer: And what makes it easy or hard for people to follow this plate base method.

Participant 1: I think it's very foundational. It doesn't require you to prescribe any specific food. So it allows the individual to kind of apply any of their foods, whether it's cultural, personal, whatever that may be. And just kind of apply it without weighing things out, which I know can have some consequences. But I think some challenges as we have some clients and patients that want more direction, want more of that prescriptive approach. In that case, it may not be as helpful.

Participant 2: I agree with what Participant 1 said. I also find - because at the rehab here, we do have some clients that have like brain injuries. And they do find some of the colours and everything a little bit overwhelming. And then they get a little bit confused. And I've had a couple people ask me like, “Oh, do I have to separate my food, the way it is demonstrated on the plate?” And so I find that, in general, it is easy for people to follow. But obviously, with certain health conditions, it can be a little bit confusing.

Participant 3: [cross talking 00:02:27]. I was just going to add that it's nice that the plate model is really consistent with the just the basics tools from diabetes Canada. So I find it's nice to, I guess be able to refer to that as well as just showing how the diabetes way of eating is really how it's recommended for other people in Canada to.

Participant 4: I would say an overall just because it's flexible. And I feel the old guide with all the different portions was kind of a too much like a one size fits all. Well, this is more general. There's general guidelines, but saying like there are personal factors that come into play, depending on your age or physiological state, conditions you have. So it's, I like the flexibility of it. I feel like I have gotten comments where people are like, “Oh, should I really be having, this much food on my plate”? So I'm like, “Well, no, you'll need to have 10 different kinds of vegetables and 10 different kinds of protein”. But otherwise, I think it's definitely user friendly.

Participant 5: Yeah, I think it's easy, just because the proportions are kind of easy to remember. And usually when I give that model, I usually also combine it with the hand, like the handy portions. Like you know, size a fist, and then to two handfuls for veggies. I think at least for the people I work with sometimes they're actually trying to eat more to gain muscle or weight. So I guess in that sense, I think it's a good base to start from. But for example, I sometimes might tweak it a little bit for example, having a little bit more carbohydrate or a little bit more protein.

Participant 6: Usually I worked with a lot of clients, I had a lot of food insecurity. So a lot of them also didn't vegetables. So when I showed it to them all the time, it's kind of explaining it's a goal. This is kind of the type of pattern we want to work towards. So maybe they start off with half a plate of starch and then try to work some vegetables in if they haven't had any vegetables in a long time. And also just showing them a different things they can have it like - and I think the visual with the different colours has been very nice and just the proportion and have - they gotten rid of the numbers. I think is a lot more user friendly to most people.

Participant 5: It is harder for some of my clients when I'm working with them because it is kind of like - it's a great starting point. A lot of my other people in this focus group have said, I wish there was more space on the side to write some notes. And let's help individualise it because I found that when I was working, I would use this because it's much more colourful for my people with diabetes. But then it's, OK, so you shouldn't be doing half of the fruits, though. And so then we're kind of like writing all these little comments on the side, and then you very quickly start to run out of space. And then it gets very cluttered. So visually, I wish it was a little more space just so that we could write some of the individual remark.

And then also, just because it doesn't say how big the plate is, so people say, so if I have a - are we talking like an eight inch plate, 10 inch plate, 12 inch plat? Or how many eggs is a quarter plate? Are we talking whole eggs, half eggs? And for my clients, then who use plates, don't use plates and use bowls instead, so that OK, so what does this translate to in a bowl? How are we still divided that bowl? Or how do I should be eating according to the food guide in that case?

Interviewer: Yeah, those are some great things to keep in mind for the rest of the focus group for sure. So I'm going to be just moving on to the next question. So which diet tracking methods or applications have you used with your clients?

Participant 4: I just use a typical food journal. I don't necessarily ask people to consistently keep one. It's more I use it as like a starting point, like in the beginning, just kind of for me to have an idea what they're eating. But yeah, I made my own like template, what they pretty much, yeah, what they're eating? When they're eating? How much they're eating? They don't always put in the details. And then I'll kind of ask them the details. But yeah, just a food journal that I kind of customise which includes like food, but also factors outside of food, like how they're feeling.

Participant 5: For me, I've mainly used My Fitness Pal. And I like that you can have like the share option. So you can kind of see like, what types of foods you're actually eating. And also Chronometer is another one I've recommended. And I guess it's you, it's also because most of the people I work with, they're more comfortable with technology. But in my previous position, I get them to do like just old fashioned pen and paper like a food diary.

Participant 2: Yeah, I usually recommend pen and paper, if I'm asking someone to record what food they're eating. But I also find that a lot of people get really bogged down in the like minutiae of that. And so I don't often ask people to do that. And also, that can be really difficult for people who have like spinal cord injuries and things like that. Because they might not have use of their extremities. And so they might not be able to do that. So I feel like I don't, that often ask people to actually like track what they're eating. But if I do, it's pen and paper.

Participant 3: I'd say older clients tend to prefer pen and paper as well. A handful like I would say, maybe 10% would, I'd suggest like using My Fitness Pal or the E -tracker was a nice option from dieticians of Canada, because it was simple. I found like clients like that. But overall, I still find people prefer a pen and paper or emailing a journal on what they're eating like just in a text of a message.

Participant 1: I've also seen some patients just send pictures of their meal. There's also another app, that's called the Better app. And that's sometimes used in private practice. And I find that to be quite useful because you can turn off the macro and micronutrients functions that the clients can't see that. So for some people that get bogged down with the numbers or might have history of disordered eating might be a trigger for them. So just to filter that out, and just really focusing on writing with the foods are so that we as dieticians can look at more than macronutrients separately. Open it up if it's appropriate for the patient, I find us a really good function.

Interviewer: OK, great. And do any of you know of any tools or have used any tools or applications that are based on the plate method or that resemble the current Canadian Food Guide? No, OK, great. That's the answer we're hoping for, for sure.

Showing the app’s prototype

Facilitator: And just based on what I just showed, did you have any first comments on that? Any thoughts?

Participant 5: I don't know if the moving, like pulling up and down is very intuitive. I felt like most apps if we're going to be doing that kind of function, it's more of that pinching motion. Where like it because if you're zooming into something where you're kind of like doing that motion versus up and down. So there might need needs to be some kind of tutorial for that, especially if we're trying to target older users as well. Also I guess, if we're kind of changed the plate size is that just so visually, you can see it larger? Or it doesn't actually mean that your plate is bigger? That would be my question, which I don't know, if you've delved into those kinds of things?

Interviewer: Yeah, we've definitely thought about it. But that's something that will be getting to through the questions as well yeah.

Participant 1: I also think there should be a numerical association with when those things change that if people don't really have the dexterity to control the portion sizes, maybe they can aim for a number at the same time.

Participant 2: Yeah, I agree. I was going to say that for older users, even though like zooming in like the pinching might not be very intuitive for them. So if they have one of those things that has like a number, you can say, like 25%, and then have like a plus and a minus arrow, buttons next to it. So you can like increase the amount or decrease the amount. That might be a little bit better for older users, or people who don't have the dexterity to do that kind of stuff on the screen.

Participant 5: The colours would be another thing. So if anybody has, like any colour deficiencies, and those colours are quite muted. So they might not be able to tell the difference between which ones are the carbs, which ones are the veggies and fruits and which one’s other proteins?

Interviewer: Yeah, great point for sure. So the first question about this is how would you view the application working to record all different meals throughout the day? So lunch and dinner? Maybe pretty straightforward, how about breakfast?

Participant 5: Breakfast would definitely be harder. Especially I think, because with breakfast protein sources, it gets a little bit murkier. So with if somebody is eating a sandwich, a breakfast sandwich with cheese in it. Is that cheese, like the protein? And if somebody is - especially with like plant proteins, like things start to blur the lines, like, Is it like just the protein? Is it something else? And also, people don't typically eat breakfast in that way.

Participant 6: I'm wondering what happens if people don't eat breakfast? Do they just skip counting that plate for that meal?

Interviewer: Yeah, that would definitely be an option not to put anything for a meal.

Participant 3: It might be tricky to for people who do breakfast like via smoothie, like trying to visualise like, what percentage is fruits versus like the protein there where it's going to be like in a cup, just a thought.

Participant 2: I wonder if it'd be possible for those sorts of things. If instead of having a plate, you'd be able to change what the like vessel was. Like have a bowl or a cup to help people like better visualise what they're doing. Because I know a lot of people for even for lunch and dinner, they tend not to eat off a plate, they tend to eat out of a bowl and have all things mixed together. So it might be interesting if they could have different options of their food vessel, if you will.

Interviewer: So you mentioned a ball a plate, you can have different sizes of plates.

Participant 2: Yeah, or like a cup for a smoothie.

Interviewer: OK, any other ideas of vessels?

Participant 3: Like reusable containers like a rectangular, I don't know a Ziploc container or something like that.

Interviewer: Like a lunch Tupperware?

Participant 4: Going back on the proportion. I think so we mentioned before, but let's say depending what you're having, you can only be having one or two food groups. So is there a way that like, I don't know, you're just having a fruit smoothie, for example, like there's a way that I can just like fruits and vegetables and it's not the grains? And then kind of how does that translate into quantities right? Because for only eating fruit while you're having one banana versus one banana plus one peach plus strawberries makes a difference.

Interviewer: So the next question is, how would you have users represent their meals portion size on a more 2D model? You had mentioned the diabetes Canada model, is there – do anyone have anything to add on that?

Participant 4: Yeah, I would say the models but also like - in like meaning, I don't know if I love the whole like half a cup of [unintelligible 00:14:50]. I think that's hard for people to translate. Looking for example, like I don't know, let's say a fruit is a fruit. A slice of bread is a slice of bread. Easy user friendly portion sizes, like in units.

Participant 5: When I have people do food journals for me on like word doc or a pen and paper, I usually do like some common objects that, I think the average person might know the size of. So, for example, if I'm asking about peanut butter, I'll say no, is it like, like a half a ping pong ball size. Like so compared to a ping pong ball. Like you're kind of grabbing that knife and putting it through the jar, like, what does it look like? Or like a golf ball, a softball, baseball? And then I have a few who have never played sports, and don't know what those things are. Then I'll sometimes say OK, can you take a picture or compared to the size of your phone. Or like a deck of cards, or something that they may have around the house.

And for those who take pictures for me, I actually will have them sometimes just put a reference object into the picture to just so I can see relative proportions of things.

Participant 6: I wonder for some apps, I think that if for the first time they use the app, if they can take a picture of like the size of their bowl, or like their cup and have like a ruler measured against it so that the app can kind of capture the size of it. And then from there, if it can proportion out how much like in terms of a portion size. Because I find a lot of people have challenges with the portion sizes. Like someone else has already mentioned, like what half a cup is, one cup is, and Participant 3 mentioned the different sizes. But I think because everybody's bowls and plates are different, and we are looking at proportions, its still is like a different amount of food that everybody is consuming.

Interviewer: Coming back a little bit to that. What would like a quarter of a plate represent? Your opinion, like a quarter of a plate of protein, what would that portion be?

Participant 6: So it would go back to like the typical like the deck of cards, or if somebody just has a higher energy requirement, and might be more. So we are going back to proportions. But I think sometimes when people are trying to track I guess more of like the caloric intake with the macronutrients, it might be helpful because everybody's plate sizes are different. Even if they are comparing, some people still get confused. I'm just wondering if there's like a more user friendly way to kind of, to make it more like consistent in terms of the app can kind of account for like different sizes.

Interviewer: I think the size of the plate changing based on what they actually have at home can be really useful.

Participant 5: I think it also depends on - I can't remember the original question was for this discussion right now. But I think, it also depends on what the ultimate goal of the app is to? Or is it just to compare it to the new Food Guide, which is kind of weight neutral. So was this not talking about specific portions, there's no ultimate goal. It’s just too kind of be better. And in that case, like maybe relative portions of vegetables to protein to starches, regardless of plate sizes, it's still appropriate. Because it doesn't actually really matter what size it is, because it's always the same plate. If we're shifting proportions relative to each other, they're still going to see improvements in their health, rather than changing the plate size. So I guess it really depends on what the ultimate outcome of this app and the guide truly is.

Interviewer: Yeah, definitely a great point. For sure. So the ultimate guide is diet, self monitoring. So allowing the person to make their own goals. We did do a preliminary study, based on the plate method in a paper tool. So we had participants draw on their plates, what it could look like. And they had a very difficult time with the portion sizes and being able to know. Like, oh, one slice of bread is a quarter of a plate? Or as you said before, two eggs, will that be a quarter of a plate, an eighth of a plate or anything like that? Which is why we're asking this question now. But again, it would depend on the participant’s ultimate goal and what they want to do, from a dietician’s perspective.

Yes, if they change that proportion, they will have benefits on their health. But from a user's perspective, it's very difficult to know how much too actually shade on their plate if they don't have a guide of their portions.

Participant 5: And this just reminds me of like the kind of like carb counting where there's actually like a guide of like, how much like bread is actually considered like a portion. So I don't know if that might be helpful having some sort of guide for the amount of I guess protein, carbs and vegetables.

Interviewer: That would be considered a quarter of half of like, for example.

Participant 4: I'm also wondering if you could do like a drag and drop option. Meaning for someone who finds it easy enough to follow the plate, they can just do the plate. But let's say for someone, as you mentioned, it's a bit harder for them to kind of visualise, OK, what's two eggs with a slice of bread? Or let's say, they're just having bread with peanut butter, but no fruit. Like, what if there's, you could have, let's say, bread on the side or fruit or whatever, and you just drag it onto the plate and then automatically will kind of generate what the plate is.

Interviewer: That can be fun too. Little pictograms and things like that. Any other thoughts on the portion sizes?

Participant 1: I think if they had like realistic dimensions of specific food items, and like options, but the plate or the cup or whatnot that actually reflect what at home, like I think provide more accuracy in terms of just the point of reference. Like egg sizes don't generally change. So by having like, specific dimensions, relative to the plate, like just allows them to connect a little bit better. So it's just something that came to mind.

Interviewer: Alright, moving on from there. What would be considered a successful day, when using an application like this?

Participant 4: I think depends what the goals are. I was also going to ask you like, is this application made for the general public? Is a made for let's say, diabetics who want to track their carbs? Like I think success really matters on what the patient's goal is?

Interviewer: Yeah, exactly. So at the moment is for the general public. So kind of anyone not really - we're coming from a dietitian’s perspective for sure. But it wouldn't be necessarily an assessment tool, as a food journal would be. It's more for the client to be able to track their own diet and make their own changes. Going back to the question, what would the successful day look like, on this application?

Participant 1: I think if the application can incorporate questions about the mindfulness, or some of the habits that Canada's Food Guide, focuses on. Like, were you mindful with what you ate today? Or did you eat dinner, have a meal with a friend or share it with family? Like some of those things might - I think would be helpful, just because they align other pieces of the food guide.

Participant 4: I think success depends on what the, what you're working towards it the dietitian, as I mentioned. So [unintelligible 00:23:07] is an option. But let's say they're the app, the client has a version, what if the dietitian can have a version where they set goals on the app. So let's say the client wants to eat more vegetables, you can put kind of a goal of the vegetables, and that could be success. But for somebody else, it could just be they're not eating enough, and they want to eat more. So I think it's hard to have a general goal, because everyone's really different. So I feel like if there's a dietitian option to kind of customise that goal, or even if we don't have access, if it's the patient, but with the conversation with a dietitian, they have the option of setting that goal. I think that could be a good idea.

Participant 5: Yeah, I agree. I feel like it should be individualised. And I also feel like, it might be like a, like a changing target. Like for example, if I'm not used to eating vegetables, and maybe I'll set my goal as maybe just a quarter of the plate as vegetables to start with. And then maybe that will be considered successful if I meet like that quarter portion. And then over time, maybe I can increase it to like half the plate. And maybe having the plate model as we said in beginning, as a base. But then obviously individualising that based on your needs. And I think having that dietitian opinion would be helpful as well, Participant 4 said.

Interviewer: So what other goals could be present on a plate on the app?

Participant 3: Meal frequency could be a goal to. It might not have anything to do with the portions or proportions, but it might even have to do with how many meals are showing up on their app at the end of the day. Are they just getting like one meal a day when the goal is to try to eat twice a day? So frequency

Participant 5: Whether or not their eating like in front of a TV or a screen or while working. I know that's especially with COVID, that's been a really big challenge for a lot of people just too kind of take themselves away from a screen and actually have like a dedicated eating time, could be a really good goal.

Participant 6: I think also fluids is important because water is something we focus a lot on upon. And for a lot of patients I used to see, a lot of them were drinking, sugar sweetened beverages. Even just increasing the amount of water and just making sure they're hydrating. That could also be part of the goals.

Interviewer: Yeah, moving on, on that, how would you suggest that beverages be tracked within the application?

Participant 6: You can ask like what type of beverage they had. Whether they had water during the meal in between meals, and they could just track it all at the same time. I think sometimes people kind of forget to drink water. And it's just something, if it's on the app and like just reminds them, oh, I should probably have some water at the meal. Even if I'm just rinsing my teeth in between, like if - just to make sure people are hydrating properly.

Participant 5: Or maybe even if you like the client sets an individualised number of glasses of water or maybe cup just because the glasses can be ambiguous. And then maybe they can I don't know, if there's say, I want to aim for eight cups of water a day. And then say there's eight icons of glasses of water. And maybe they can kind of like click how much they ate that day. And sorry, just to backtrack to your previous question, I didn't get a chance. But I also want to say that I think another like goal to aim for is food quality. So even if they're meeting like their carb proportions, is it whole grains, etcetera. And also, for proteins it can be like choosing lean proteins, just because I feel like the fat content isn't really inherent in those groups.

Participant 6: Having maybe some –

Participant 4: In terms of –

Participant 6: Sorry, go ahead Participant 4.

Participant 4: Oh no, I was just going to say back to your question about tracking the fluid. I think Participant 2 said before, like, kind of having different containers. So I feel like yeah, you could have like glass of water, you could even have like plastic bottles or those reusable water bottles, because I think people don't always intuitively know how many glasses of water. But let's say if someone's drinking out of those water bottles all day, they'll kind of know. OK did I have one, did I have two, did I have three? Or also including, let's say, cups of coffee or tea that people drink that too. I think kind of having those containers or those different options. I don't know juice bottles, things like that, that people just drink in general.

Interviewer: So tracking kind of on the same interface as the meals would be tracked on.

Participant 4: Yeah.

Participant 5: It'd be really cool if and I think I'm diverging from your original question now. But I think it's a really cool if the app could come like this, Participant 1 was saying was, you could like, initially, when you're starting up the app as part of setup, kind of choose like the plate that kind of most, or the dishware that most resembles what you have at home. Especially with like beverages. Because I find that either people have like short glasses, or they have those tall glasses or those like big "Starbucksie", reusable straw container kinds of things. And so you can kind of select that. And then if at each meal, you manually doing things you could like take a picture of your whole meal, including the glassware, and then you got AI to kind of analyse it for you.

And then they can kind of correct if they need to. If like the - if it misread starch as like vegetables, or if I've missed veggie protein as a starch or something, it can correct it. And then again, with AI, depending on how much money you guys have, it could like to look to see like if it's like whole grain or if it's like looks more refined? Or if it looks like there's like leafy vegetables, is there variety in the vegetable portion? Or is it all the same colour? Like that would be super cool. And then you can look at trends over time to kind of - then that could be linked into like the successful day. But I know that would be really expensive. And I don't know how much money you guys have in your lab.

But like I think if we're talking about like the best way, especially I think because there's so many health and fitness apps out there now. It's going to be really hard to kind of get people to use this app. If it's too close to all the other apps out there currently, like if we're tracking our glasses and kind of still having to input all these things, then people will say, well, why don't I just use X, Y, Z, instead? Because I already know that, I already use it. It's already on my phone.

Interviewer: So AI definitely a lot of different options that we can do with that. Unfortunately, not necessarily in our budget for the moment. But something to keep in mind for the future as well. How would you suggest that mixed dishes - kind of building on what you were saying - if we take a picture of foods, they wouldn't necessarily be able to see what mix dishes would look like. So things like stews, you mentioned soups, pokey bowls, even, Justin’s favourite food. How would those be recorded in the app? Justin is getting ahead of us. So for mixed dishes would be more things like stews, lasagna, pasta dishes, things like that. How would those be recorded in the application?

Participant 5: I think that would be probably difficult for some people, especially if it's something that they didn't make themselves. If it's something that they made themselves, then perhaps if there's the option for like the bowl, or something like that, they could build it into the bowl. But I guess one of the potential things that we could do as dietitians is help people with foods that they didn't make themselves or foods that they're purchasing outside of their home, to try and estimate as best as possible. But I think that that would definitely be hard for people.

Participant 2: Maybe if there was an option, so if it's not like that defied plate kind of thing for the meal, maybe there's like an option for them to select, like, it's a mixed dish, like it's hard to say. And then I find that with mixed dishes like generally, they're like, it's like lasagna, it's or like shepherd's pie, or some kind of casseroles. So maybe you could have them the option of like, this was homemade, this was store bought. And then for store bought, then the app would just have like a database of like some - a good average of what like a general lasagna would be if it was a store bought one. And then if it's like homemade, maybe they can input a recipe or something like that.

But aside from that, I think even some dietitians, when we kind of look at mixed dishes, we're like, well, I don't know what's in there. So I really can't tell you if it's following the plate guide. Because there's, it's so hard, to know exactly all the ingredients unless you actually know the recipe.

Interviewer: Alright. So now we can get to the question that Justin posted in the chat. So many other foods aren't shown on the Canadian Food Guide. So which foods would you think your clients would have difficulty to represent on the plate? And how do you think they could be represented on the app?

Participant 2: I think that things like snacks and desserts aren't really in the current plate model. So if people have things like, like chips, or muffins, or whatever they have as a snack. So I wonder if maybe there could be kind of like, what Participant 3 was saying in terms of being able to choose that it's other, a mixed dish, I wonder if it also be able to put an option and that like other it's something that's not – that doesn't easily fall into one of the three groups that's represented on the plate. And then you could just have it and you could maybe type it in or you could have a couple broad categories like dessert, bakes goods, things like that. That you could kind of identify and then still try and get an approximate portion size, yeah.

Interviewer: To have another foods kind of category on itself?

Participant 2: Yeah. And then maybe some broad categories like, no, like chips, baked good, ice cream. Just a couple of broad things so that people could - that way, it'd be a little bit more clear. Because if we're looking at it, that the -like a week of their meals or something, at least it doesn't just say other, it would have a little bit more information as to what it was. That could be one way that they could track it. But otherwise, it doesn't really fit into the Canadian Food Guides model.

Participant 3: I think it's a good idea, [cross talking 00:33:52] - sorry go ahead.

Participant 4: I thing on top of that, that's where the drag and drop could come into play. So let's say if it's a small bag of chips or a muffin or a cookie or, I don't know, something like that, like a bowl of ice cream, I think that's when the drag and drop can be very useful.

Participant 3: I was just going to add that fats and oils are a little bit trickier to kind of track or be represented on the new play model too. So they could be eating half a plate of salad every day at lunch, but is there like a half a cup of dressing on top of it? It's not really clear. And other food grouping too is like the seeds. Like flax seeds or chi seeds, like things that are as I guesses obvious, maybe it's like peanuts or walnut, things like that. But like those added seeds, like maybe they want to show that those are part of their eating style, but they don't really know where to put them.

Participant 5: Or specialty products, right. So I have a lot of people who are trying to reduce carbohydrate intake right now. And so they do a lot of the Kato stuff. So like if we're eating Kato chips, like cheese chips. Where does that fall in right? We wouldn't really encourage that to be like a main protein source or nor do they kind of consider it a protein. But kind of where does that fall into things or when people are kind of doing like a protein bar for a meal or adding a protein powders into their beverage? Because they can't get it in like protein elsewhere? How do they kind of incorporate that?

Interviewer: That's what we're asking you guys. So if anyone has any ideas, please feel free to share or think about it.

Participant 2: I wonder also, if there could be an option for someone just to type in what they had if none of the categories fit what they had. And if there's a couple, like other broad categories, but it's something really specific, like Kato cheese chips, or like a really specific protein bar or something. If they could type it in. And then like Participant 4 was saying, with the drag and drop, maybe they could then put that on their plate to indicate that they have the other meal. And it could just be like a different colour than the grains, proteins and fruits and veggies. That could be one way that they could do it.

Participant 1: Or is thinking if you can enter the macronutrients. If you have the food label, it can kind of transpose that onto the plate. So if it's like 15 grams carb in this bag of chips, then you enter that in. So write that it's like Kato chips, but on the plate, it shows that your protein section just got a little bit bigger. Just so that it somehow goes back onto the plate.

Interviewer: Yeah, that'd be really interesting. Difficult for sure, but very interesting.

Participant 5: Or it might just have to be like a bunch of like, prompting questions. So like, after they kind of initially do it, then it kind of goes through this set of questions. Like, we noticed, you didn't add any water? Like, did you have an – or you didn't add a beverage? Did you have anything to drink with your meal? The next prompting question would be, did you have any other foods right as an example? And then was it like a logic sequence? So just having maybe these questions regardless of what happens on that meal, and then maybe they don't get like that ultimate feedback of what that day looks like but if they're working with a dietitian, then hopefully the dietitian gets all the information on the back end.

Interviewer: Yes. So kind of having that other as a category on the plate and then adding themselves kind of more details about it or breaking it down into separate categories as Participant 2 mentioned.

Participant 4: I like the question aspect that Participant 5 said because I feel like it kind of depends, if you're having a meal versus a snack. If you're having a - most people don't consider let's say chips as part of a meal. So let's say through the options say, OK am I eating breakfast, lunch, supper, snack, whatever. And then you're selecting snack then you'll have kind of the other category where let's say if you're selecting meal, it's going to assume you're having like some type of I don't know plate model. So I feel like that could be a good idea that kind of a distinguishable between a snacks and meal. Because it's normally generally most of the time where the snacks won't necessarily be like a plate model, while the meals for the most part it is.

Participant 6: If we're doing the question thing, though, I definitely would not want the other foods in quotations so that we're kind of de-stigmatising that category, yeah.

Interviewer: Definitely because we don't want to make it seem like flax seeds and hemp parts are not part of a balanced diet or chips even stigmatising any foods as well. For the purpose of today, other foods, are just a very broad category, but breaking it down could be a great idea as well. So how, what are the main sticklers on the Canadian Food Guidance, dairy? So how would you suggest that dairy and specifically liquid milk products be tracked on the application?

Participant 2: Or milk, like liquid milk products? I think we were talking before about potentially having some diagrams of cups. I think it would be cool if you could fill the cups with things other than water. Like if you could fill them with juice or milk or whatever coffee tea soda, whatever it is that they're drinking. And that way that could help with tracking kind of liquid milk and also other beverages. And you could have the option if they're drinking like soy beverage or other things like other alternatives. You could have the option to fill the cups with those things.

Participant 4: I will say besides from milk to maybe included as a protein category, like even though, technically could be a bit of a carb, too, we kind of have the same thing with let's say legumes, for example. And technically they're part of the protein side. And I believe that on the plate itself, like cheeses and yoghurts are in the protein section.

Participant 6: A little bit of a tricky one there, so dairy itself, and soy milk would be in the protein section. And then the other beverages could be, as Participant 2 mentioned, just something that you're filling a cup with.

Participant 4: I think, especially for beverages is good to fill the cup. Because let's say otherwise, you could be confusing, especially with the milk alternatives. You can also say soy milk could be a protein, but almond milk isn't. So I think the cup is very useful, at least for the beverage part.

Interviewer: And should any beverages be included as part of the other foods?

Participant 2: I do think that maybe there could be an option in the other foods for beverages that might not traditionally fit in the other categories for things like soda, milkshakes, anything that anyone might drink. Just so that they can kind of fill those cups as well. And at least have it tracked.

Participant 6: Kampuchea.

Interviewer: Even tea and coffee, things like that they're not necessarily equivalent to water. And so Participant 1 had started mentioning it earlier, but what other elements of the Canada's Food Guide should be included in this application? Have the mindful eating, eating as a group?

Participant 2: Yeah, I think going with what Participant 1 and some other people had said, with the prompts, I think that would be really cool. You could prompt them to ask them, “Did you eat this in front of a screen? Did you have any water with your meal”? Things like that. I wonder also, if we could, if you could put in like suggestions. Kind of like, try and choose whole grains, or try and have mostly water as your fluid or like at least half of your fluid should be water, things like that. To get people thinking about their habits, and kind of prompt them because I know it's easy to forget these sorts of things, yeah.

Participant 5: For some of the other ones, I think, I can't remember the last time because I forgot to actually, I didn't look at the back. But I think there's something about like food marketing and stuff, too. And there's like food labelling. So kind of how the - is it the – what was the app that used to train people on like, healthier behaviours, and rewarded you? The Carrot Rewards app. Yeah. So kind of like carrot where like you were every now and then kind of have these surveys, right. So it could kind of kind of "gamify" it a little bit too. So for people who download the app, every now and then you have like a push notification on like, like, how well can you read food labels?

And then to kind of go through this thing. And then if it turns out they're not great at reading food labels - I know like Health Canada has like a whole like label reading guide and course or something like that. And you could link it to that. Or it could be something called like, which ones are like examples of really bad food marketing, or something. And so I guess so that it's not super repetitive, where after each meal, they're answering like all these prompting questions. Because then I guess you got the risk of going on to like, just like robotic mode or like no and no. And you're not actually reading the questions anymore. So for those other aspects of the food guide, it could be through like push notifications or some other kind of engaging aspect.

Interviewer: I can give a little bit of a refresher just by sharing the backside of that again. So this is what it was. Being mindful of your eating habits. Cooking more often. Enjoying your food. Eating meals with others. As you mentioned, the food labels high in sodium, sugars and saturated fat and being aware of food marketing.

Participant 2: I think it would be cool if for some of these things that like linked to maybe like websites that could provide ideas. So like with the reading labels, I know there are some stuff online that could have that or like for cooking more often. If it could link to some recipe sites. I know there's what's it called, the Unlock Your Food one run by Dietitians of Canada, I think that's what it's called. Where you can put in your goals. And it gives you some recipes and some menu planning just to give people - because it's great to tell people to cook more often. But if they're not good at cooking, or they don't know what to cook, then you can kind of direct them in a, or like push them in a direction that has good recipes. These are from Dietitians of Canada. So hopefully they have some good stuff in them. I think that would be helpful for people.

Participant 5: And this might be a bit unrelated, but I think there should be like going off of it, I think there should be some sort of education component to the app. Just because at least from the patients I've spoken to, even though there's the Canada Food Guide has like photos, sometimes clients don't actually know like what a carbohydrate is especially or even like a protein or they get confused with it. So I think maybe even having like, maybe like a list of foods in each category, or some sort of education component. And also, I think that would also help them when their reflecting on their food diary. And kind of seeing whether or not they actually made healthy choices and maybe like the function of the foods as well.

Interviewer: That's actually our next question. So which instructions and support should be provided to users to help them use the application? You mentioned the lists of foods. Anything else that you guys think about?

Participant 4: I would say like an intro video, maybe. Kind of like teaching you what's a carb? What's a protein and what's a fat? What is the guide? These could be optional, because some people don't really have patience. But yeah, kind of like when they log on and make and make an account. They could watch like a short intro video that teaches them how to use the app and what each food group is.

Interviewer: Or maybe like little icons of different foods and might be more user friendly, instead of like a list.

Participant 5: I think also, it's just something that states this is for like the general public. And that if they do have other health conditions like this, in no way like replaces. Because even in my practice, when I talk about the food guide, like oh, but I had diabetes, and it's not right for me. Well, you're no longer the general public. And so people kind of forget that, these tools are for like people without any chronic conditions. And although it seems like more and more often, the general public might have chronic conditions. Like I think, again, those lines are also kind of getting blurred. So just kind of reinforcing the who it's intended for and how it's meant to be used, which I'm sure you guys would do anyways.

Participant 1: Also think that it should connect to any supports available depending on the province that the person is living in. So say in BC, if you have any questions call 811. Or health BC or wherever the province is, whatever the province provides, in terms of dietitian support, I think, would also be a good resource to include.

Participant 4: Yeah, to second what Participant 1 said, like here in BC, we have Health-link BC, I don't know what you guys have in Quebec. But I also think that if you're working with a dietitian, if there was the option to maybe put that dieticians like contact info or something in the app, so that if they needed support, they could maybe like send a message from the app to us or something like that. I don't know how easy it is to actually have happened realistically, because I don't know very much about app development. But if you could get, like if you could send a message saying like, this is my plate from today, I have a question about this. And then we would be able to help. That could be something that would be helpful, I think for people.

Participant 6: I'll link back to the Health Canada website. I'm sure if they're using the app, they're well aware of what the Food Guide looks like. But there's tonnes of resources on the Health Canada page about like mindful eating and like less distracted eating and cooking at home. Like there's I think a page for each one of those points on the back of the guide. So having a link right to the website might be helpful.

Interviewer: Definitely.

Participant 1: Just to add to what Participant 6 mentioned, I think a lot of people don't understand why the Food Guide is the way it is. And I know Health Canada has done a lot of research as to why they make those recommendations and how they align to either the EMDR or meeting the DRI. So like for those who are interested in might be useful to include links to read up on all of that research and all that work that Health Canada has done.

Interviewer: Supported a little bit more to.

Participant 5: Maybe also just some common, myths around the Food Guide. Do I have to have dairy? Or all those things that that we always hear day to day about how the Food Guide is kind of like made because of corporations and industry. So just kind of reiterating and even if we're just kind of going through like, the important nutrients that we get from each category, right, and so why it's important to have them and then again, like linking either back to the Health Canada or other some other reputable website.

Interviewer: So coming back to the educational component, you guys were mentioning a little bit throughout. What other things could be part of that educational component of the app?

Participant 2: I wonder if - I know that the app is for the general public, but I suspect that people who have chronic conditions will probably stumble their way onto the app. So maybe there could be some links like to diabetes Canada, or to things like that for common chronic conditions. Or for people for like secondary stroke prevention and hypertension, it could link to – [cross talking 00:51:16]. Just to give them some information. So that even though we might have some sort of disclaimer saying like this is for the general public for generally healthy people. And if you do have a chronic condition, then it's probably best to speak with a health care provider, but they still might want to do research on their own. And I feel like its best if we direct them to proper resources. Because we all know that there's unfortunately, tonnes of stuff on the internet, that's probably a lot incorrect. So if we could have like Heart and Stroke Foundation, Diabetes Canada, like good, vetted proper evidence based resources, then I think that could be helpful for people as well.

Participant 1: I agree with Participant 2. Any type of provincial or federal partner also helps just bluster the - not the reputation, but like the credibility of the app too. Especially with people don't really know what it is. And it's going to look very simple. It's great to have some of those third partner connections.

Interviewer: For sure. Alright, so which features do you think could help with adherence to using the application? There are many before had mentioned, not making it just a sequence of answering no, no, no every single time. The "gamification" as well, that you've mentioned before? Is there anything else that you think could help people stick with the app and use it long term?

Participant 1: I think positive reinforcement and positive feedback. I know the Carrot Rewards app was re-launched through Optimity, I think. Which I think was just earlier this year. And they do a lot of like, positive feedback with like, either little trophies, like looks kind of similar with the “gamification”.

Participant 2: I think one of the other things that could also be helpful is like push notifications that you get. I have an iPhone, I'm not sure how it works for other brands of cell phone. But I'll get notifications about like, hey, Participant 2, come in and fill this out or something. Or at noon, it could say like - if I've put nothing in it could be like, hey, Participant 2, like have you agent today? If so, come to the app and put it in. Just too kind of remind people because, I'm guilty of that, if I don't have it kind of in my face, I sometimes forget.

Interviewer: And how about any, like social elements of the application?

Participant 2: I think if there could be like a community board or something where people could have discussions. Of course, I think it'd be beneficial if that was to be moderated to make sure that no incorrect information was being disseminated too widely. But if people could talk to other people and maybe like cheer people on. Send awards to other people, like you get a gold star from me because you did a week of logging your breakfast or something like that, just to keep people motivated. And then they can also talk to other people, maybe that they don't know personally but that are also in the same situation like trying to get better eating habits.

Participant 3: I share the link in the app to. Share a link in the app so that they can invite a friend to start tracking along too.

Participant 5: A challenge component kind of like when Fitbit first - like you can challenge your friends to like a who can get more steps in a day. So something like that, like how many days in a row, can you log in? Or how many days in a row can you hit like half a plate of veggies? So maybe like, having them be able to set a goal and like challenge your friends, like in a fun way, could increase that. Back to the other question, though, of what other kind of supports. One thing I hear often when I talk about the healthy plate model and Canada's Food Guide is cost.

So a lot of people do think having too much vegetables, especially with COVID, and everything increasing in price, they are worried that they're just not able to afford. So if there can be education and supports around food security, and links to food banks. Or there's so many other like food waste apps, now we're looking, you can get like cheaper produce. Things can be used just within the next day or two, but at a discount.

Interviewer: Yep, that's a great point for sure. Alright, so the next one is which features are required to ensure accessibility for all users to using the app?

Participant 4: Maybe the language I know, specially, or in Quebec, here, a lot of people are French. So maybe having like different language options. That's all I could think of. But I'm sure there's more.

Participant 2: I also wonder if you can change the size of the text for people who are older and might not be able to see as well as smaller text. And also just thinking like in my specific population, which is spinal cord injury, if there was some sort of like talk to text, functionality. So that people who don't necessarily have use of their upper extremities or might not be able to have the fine dexterity to use a cell phone. They could kind of like say out loud something and then it could be converted into the app, I think that would be really cool.

Participant 3: Or if the questions - if there was like a speaker box at the end, so the questions could be like, read out loud. For people with lower literacy, or even visibility, just reading the questions. It's hard sometimes.

Participant 2: And I guess it's more technical, but maybe having like a desktop version, as well, if like, a client doesn't have a phone.

Participant 5: In addition to French, just because Canada is so multicultural having different languages, kind of like how the food guide is in different languages, would be great. And also, again, like just like making sure that any colours used are kind of heavy contrasted. It's something that's always on my mind, because my partner is colorblind, and he always complains that he can't see things. When there's supposed to be a difference. He always complains and drags me to look at a screen for him. So just making sure that we remember that population as well.

Participant 6: I think all in addition to the languages, because a lot of our population is very culturally diverse. If there can be a component where if people are adding the specific foods, like if they're typing it in, or a drag and drop to also reflect foods of different cultures. I've noticed before with some of my clients, they miss categorise the food group. So then it might be helpful to just have things pop up. And then if it's in lists of different categories, they can kind of see like, “Oh, this is actually a vegetable, I thought it was a protein”, like I've had that happen on a lot of occasions. So I think it is also a bit of a learning opportunity for them as well.

Interviewer: Having that cultural piece of different foods from different cultures, in like the running lists or in the category as an example. All right, so moving on from that question, which other features of diet self monitoring could be helpful in general. So at the end of the focus group today, is there anything else that came up that you've thought about throughout or now that could be helpful in the tool?

Participant 2: Along the lines of intuitive eating a lot of people now are just paying attention to like how full they feel after a meal. But, are they feeling good? Are they feeling quite stuffed? Did they eat quickly? So those aren't exactly part of Canada Food Guide right now. But I think it's something that people are being more and more mindful of when we talk about mindful eating. So maybe having some kind of feature where they can also track those components.

Participant 6: So with all of the tracking, is the feedback relate to the user like on a daily or monthly? Or how is the progress or the changes of how they're different proportions? Like if they're eating more vegetables, how is this relate back to the user?

Interviewer: That's a great question. We haven't determined that yet. How would you think would be useful for them?

Participant 6: I think because goal setting is something that should be something people have in mind when they use the app. And because change takes time, they can set up if they want, like monthly updates. And then over a longer period of time, if there's something graphically that can be shown like this is where you were three months ago, a year ago, I'm assuming that they are using it long term. And then seeing kind of the progress they've made and seeing how they want to adjust the goals as they go along.

Interviewer: Like multiple different checkpoints and options and comparisons, to see how far they're coming?

Participant 5: The other thing that kind of comes to mind is the new Canada's Food Guide, it is more plant forward, right? We're including, we're calling it proteins now. We're including more legumes in that protein category. And a lot of people that I see they're kind of interested in that plant base eating, they're not aware that not all plant proteins are equal. So and then I start to see a lot of lysine deficiencies. And so maybe something in there just saying, it's purposely done that it's only legumes are kind of in that plant protein piece. Or kind of linking them again, to let us more education on how to follow a healthy plant based diet if they're completely eliminating all animal proteins from their diet, just so they don't start to feel sluggish, low energy, etcetera.

Participant 2: Something else that I just thought of that's not necessarily this question. But in terms of like the other supports that you could offer. For people who are using the app to try and develop healthier habits, but are not already connected to a dietitian. I wonder if there could be like a link to like, find a dietitian in your area, if they are interested in develop, like getting more professional help. I think that could A - put us more out there as professionals. And also just help people who are looking to make more improvements or make other changes, and they just want more advice.

Interviewer: All right, is there anything else that you think of at this moment, that we could include any app, regarding anything.

Participant 3: Just a thought, if there's a bit of an app community, like on Facebook, or whatever, it might be need to link in some like, little, I don't know, 15 minute workshops, or like little education pieces on some of the components of the app. So whether that's like troubleshooting, or maybe it's about like variety in your eating or mindful eating, like things like that maybe just to engage the users in a live way, just an idea.

Participant 5: Also through a different media, maybe not Facebook, or Instagram or anything like that?

Participant 4: Building up on that, out of any, any of you have used like Flow, which is like the period tracking app, but they have some like videos in general, just like well being and like all kind of just different ideas. So I don't know if you guys would like this or not, maybe have like different types of nutrition videos out there, or like physical activity or just things that relate to general nutrition and wellbeing. And the reason that I brought that up, because I know in that community they also have these like secret chats or these forums. So I don't know, just a thought.

Interviewer: For sure.

Participant 1: It's also thinking if the app can incorporate Nutrition Month related themes as well just to give it more of that seasonality and haven't kind of throw like depending on the year.

Interviewer: Again, trying to avoid it being static and always the same things, yeah.

[End of recorded material]
