# Supplementary material for: Perceptions of self-monitoring dietary intake according to a plate-based approach: A qualitative study
Source: PLoS One. 2023 Nov 28;18(11):e0294652. doi: 10.1371/journal.pone.0294652 (PMC10683993; doi:10.1371/journal.pone.0294652)
Supplement: S5 Appendix — (ZIP) [file pone.0294652.s005.zip › Anonymized RD Focus Groups/Icanplate-rd-focus-group-2.docx]

**icanplate-rd-focus-group-2**

[Start of recorded material]

Interviewer: This is focus group with dieticians number two on July 22^nd^ at 5pm. So the first section will be on self-monitoring apps and the use of the plate method in practice. So the first question that I have for you will be do you suggest following the plate method as illustrated by the new Canadian food guide to your clients?

Participant 1: I think I’ll just break the ice and say that I personally don’t talk about the plate specifically to the geriatric population, but with the food service in place the food is, the plate I should say, the plate method is followed. I know that you guys are developing an app for personal use and I think that one of the hurdles that’s an individual has when they see the plate is when the meal that they prepare isn’t in a plate format. So for instance like a sandwich or a casserole.

So I don’t know exactly how you guys are developing the app, but if there’s a possibility of showing like a casserole, but then breaking it down and showing if it was put out in a plate I think that that’s something that could be very useful for the users.

Participant 2: Could I jump in on that? I agree. I don’t use the plate with my current clients. When I used to work in the CLSC I did specifically for patients who were diabetic, who’ve got diabetes I should say. Personally I see why they use it, but I agree with the criticism of it’s too sectional and I don’t think most people eat like that and so I think that it can be a little bit hard to conceptualize how someone could actually apply this way of eating with whatever culture is theirs and whatever recipes are theirs you know, so I think that’s a really fair point that I wanted to expand on.

Participant 3: Yeah so maybe I’ll jump in. You know in sport dietetics we’ve been using a plate model for many years. We developed the United States Olympic Committee and the sport dieticians you know, work together to develop three levels of a plate of which the first level, which is more for the aesthetic sports, lower energy sports is actually what you guys see for you know the latest iteration of the food guide and the level two and three plate actually then have increasing proportions of the plate given to the carbohydrate rich food specifically.

You know, your [unintelligible 00:02:53], your legumes, your starchy foods, your different types of grain cereals etc. And it also, what I like about it is that it doesn’t, it talks about, in sport we’ve got to talk about, we have to come down to a nutrient level, we’ve got to talk about the importance of carbohydrate foods, we’ve got to talk about protein for recovery and how fat plays a role in immune function etc.

So even though they’ve gone from mixing a bit of food and nutrient words in this guide, it’s similar to what we’ve been doing in sport dietetics and in diabetes education as well. You know, we don’t talk so much about, we talk about carbohydrates and then which food groups are sources of carbohydrates and proteins etc. So I don’t find it, I actually you know when the office of nutrition policy and promotion came out with a plate from the athlete perspective, it just worked really well. It sort of gave further support for the level three, two, one plates that we have to talk about fueling your body for performance and how proportionality really plays a role.

But in counselling for the general population which is often much more diverse in ethnicity and in culture around food, then typically you find in athletes because it’s a very, you know athletes are, it’s not an equitable playing field, you don’t find people of, it’s not quite as diverse right so it’s not open to everybody. So the problem is that when you use that plate as a way to talk about food, different cultures, there are many cultures that don’t eat from a plate. And they look at it and go how do I use this?

Like we put something in, you know we put dishes in the table and we’re all picking from them and eating different things. And like this doesn’t really work for me or I eat in a bowl and everything’s layered on top, like I don’t get it. So that whole plate thing only works for the cultures that work from a plate and conceptualize food from a plate so that’s kind of my feedback on it.

Participant 4: I use it in diabetes education. I think it’s something traditionally used, but I think we use it as a very basic level of understanding proportions of nutrients to others. So emphasizing more vegetables than let’s say protein etc. So it’s not that it’s like your plate should look like this, but understanding how the portions should maybe be a little different depending on what you’re eating.

But again we use it again also to try and identify what carbohydrates are compared to other nutrients so it’s good for that. But I think looking at the plate there’s more to it right? Like it’s hard to just send somebody with the plate method handout when there’s so much more to it and I think that’s our role as dieticians to, we should never give a handout without explaining it further. It’s a role to kind of individualize it and give credit to other cultures etc. like that.

Participant 5: I think my opinion on that is pretty similar to what Gabriella just said. I use it in private practice mostly because I think it’s a good starting point to talk about macro nutrients and how the, well let’s say in this case, the plate can look like with you know making sure that you have enough vegetable in your plate or maybe later on you can talk a bit more about vegetarian options or fibres.

So it’s a good starting point to see how your daily plate can look like even though it’s not mixed. Sometimes it’s nice to have more of a visual separated and then when it gets easier to put it in every day living you can make the food and usually my client don’t have any problem with that, but they like the idea of separating things first just so that they know a bit more of the quantity and what it looks like.

OK I thought I had enough vegetables, but I didn’t or I thought I had enough protein, but it’s really not a quarter of a plate, but yeah it’s a good starting point. I think what we need to explain why and not focus on the plate for every follow up I think.

Participant 6: And I agree with Sandrine and Gabrielle. I’m not using like the plate to guide my customer. Like I’m just creating courses on line so I’m just taking that as an example for them to follow, just to have a rough idea of what to use. And also a bit like Sandrine was saying, I’m telling about like using that proportion type of thing just for making the food available to the kids.

Like let’s say you want the kids to eat more vegetables, just put more vegetables available to him instead of like more meat or more protein and stuff like that. And yeah, so it’s just more [unintelligible 00:08:12] and I don’t have any like course or thing that focus on that. Just I’m using it as a tool more than like I mean, yeah.

Facilitator: Yeah so I’m hearing a lot of the plate as a baseline tool and then I think of what the guide is having as a general recommendation to the rest of the public. And as you did mention, dieticians are going to be the key factors in explaining how it’s going to be used and this why we are consulting you today to help develop what an app could look like based on this guide. So next up is which diet tracking methods or applications do you currently use with your clients?

Participant 1: I use Keenoa.

Participant 2: I’m not using any.

Participant 3: Sorry what are we using as what?

Participant 2: I’m not using any app.

Participant 3: And Sandrine [unintelligible 00:09:03] you said what?

Participant 1: Quinoa. The one with the K.

Participant 3: Yeah so I’m from McGill and I use Keenoa a lot. And I’ve been working with them to help try and further that development. It’s a really fabulous tool for the dietician you know. It really is. And my clients find it not, like with any database application it’s the challenge of finding things, but the fact that we as dieticians can put in recipes for them that they’ve given and then they can choose them when they’re having trouble pulling it apart, so I’ve yeah, really great tool.

Participant 4: Can you type that in the, so I can see how you spell it?

Participant 3: Yeah. K.E.E.N.O.A. I’ve put it in. It’s supposed to be so that you can say it in French and English.

Participant 2: I don’t use in my line of work. We’re still doing everything on paper. [Unintelligible 00:10:13] and it’s just check, check, check on percentage wise and then I do an estimate. I will say if people ask me, like my friends ask me what app I recommend they use, I tell them to use Chronometer because I think it has like a pretty decent database. I like it a lot better than My Fitness Pal because it goes into a lot more detail. And so you can really kind of pin point where the problems are.

Participant 5: My situation is pretty much the same as Danielle so I personally don’t use an application in my line of work, but I am familiar with Chronometer and that would be the one that I would have suggested. I wasn’t even aware of Keenoa so I’ll be looking forward to seeing how that works.

Facilitator: So what has made it easy for your clients to use these applications?

Participant 4: I use an app, but I use a different app called Carbs and Cals. It is not just carbohydrates, you can choose the nutrient. We use it more for carb counting, but you can use it for all sorts of nutrients and it’s a really good visual.

Participant 5: Is it made for diabetes only or it’s for the general pubic?

Participant 4: Yeah it’s anybody. You can choose what nutrient you want to focus on and which ones you don’t want to use so often my patients might just choose carbs and fibre, but like encouraged all nutrients if they want it, but yeah. So it’s a good one.

Participant 3: To kind of piggy back off of what Gabrielle said I’m pretty sure it’s Carbs and Cals, but what’s really great about the application, if I’m not mistaking is that it’s very visual. So sometimes people have trouble visualizing the portion size and that could be a barrier to having the correct proportions whereas I’m pretty sure the application has images in a plate so you can visualize it better.

For instance teaspoons of peanut butter or what not then there would be like those little individual packs just so that people can visualize. So I think that obviously we want the application to be user friendly, but I think that a lot of like visual images are really helpful.

Participant 1: Exactly. You can choose from, as you change the picture size and it’s on a standard plate so people can visualize it in their plate and as you change the picture it changes the nutrients composition.

Participant 3: Yeah. And I think the nice thing about the Keenoa app is that it actually integrates in the multi-past method for dietary intake. So it prompts the person to say, oh you chose a hamburger. Oh, what kind of bun did you have? Oh did you have condiments on it? You know so it’s that whole multi-past method which is hugely important if you know, to do a proper dietary assessment on someone like a 24 hour recall which is what we use it for.

So that’s really integrated in, but it’s done in a way that’s really user friendly compared to a lot of the if you’ve worked with any of the 24 hour recall tools that are online which are you know, overwhelming for people to do so because this also, you know they can just put a picture in an artificial intelligence can, recognize some of the pictures and then prompt them, it also makes, it facilitates the participant’s use because they can always bail and just say I’m going to put the picture in and then you still have a lot of information in the picture even if they don’t do all the detailed reporting.

And by the picture then you can start to ask them questions when you talk to them about oh well, so I noticed in your picture you had this so tell me a little bit more about it and oh, how big was that plate? So you know, super, super useful tool. And the other thing that makes it easy for clients is that it encourages, you don’t put in calories, carbohydrate, protein, like they’re not seeing nutrients unless you want them to, but when you’re first using it you’re just saying, hey, record your food.

So it gets, it changes their motivation so often they will, if you go and record on your typical My Fitness Pal, you’re going to start saying, oh how many calories have I had? And do I put that down or do I not put that down? And then people change their behavior. So by not letting them see the nutrients and then also including questions on their sense of fulness, how hungry they were, the satiety, like the clients really get into it and it motivates them to keep going and then they start to see patterns.

When you go over with them they go, oh wow, you know. Like I’m choosing that every time, I’m saying like if I don’t get enough in the meal before, I’m not satisfied. But anyways, it sounds like I’m trying to sell you guys on Keenoa, I’m not. I’m just saying that these are all the things that I’ve found through using it, are just really amazing at, really helping understand their food experience so we can work better with them. Yeah.

Participant 1: I totally agree with you, Beth. And I really like the intuitive eating question. I think this is one of the things that separates Keenoa from other apps. I don’t know much more the other apps that I know that I know that they’re not as much intuitive eating. And since the question is always there, it kind of forces, I don’t want to use this word, but it forces the client to ask if they are really feeling hungry or if it’s more an emotional hunger or anything else so I like [unintelligible 00:15:53].

Participant 3: And you can turn those off too right? You can turn of the intuitive [unintelligible 00:15:55]. That’s one of the few, the limitations is all these different mobile apps, there’s very few that are available in other than English. So this is a great thing you know, we’re in Quebec and we have, or Ontario, we have you know two languages so it’s great.

Facilitator: OK so the next question is with regards to any tracking tool so paper or mobile that current resemble the plate method or the new Canadian food guide. Do you know any?

Participant 3: That resemble the plate method?

Facilitator: Yeah.

Participant 3: Yeah I have a paper tracking tool which actually has plates on it, small, medium and large so they can pick and do their, you know divide it up and show proportionality for people who prefer, there are people who want to do paper based tools. So I have designed those to use with my practice.

Facilitator: And if you don’t mind sharing, how does that work for you?

Participant 3: It’s good for just getting people to think about proportionality on their plate and then you know realizing there’s not a lot of things on their plate sometimes. Like there’s no vegetables there. But I often find it’s good with kind of what Danielle said that having also a check off paper based tool that allows them to go by sort of categories of food to you know with kind of like the diabetes exchange where you know you have serving sizes of food and they can check off how many they got and then they look at the plate and go, oh you know, in that plate that I have this and this, this is how many servings I had in each of those proportions of foods on my plate. So it’s kind of like the two together so that works pretty well.

Facilitator: Very interesting. Does anyone else know any tools that are based on the plate method? No? OK well that’s why we’re here so that’s good.

Showing the app’s prototype

Facilitator: I’m glad we’re [unintelligible 00:18:03]. Do you have any questions about that?

Participant 2: I think it makes sense. I think that’s a good idea because I might personally love having all the little details and all the minutiae. You know I know I got this many milligrams, but that’s irrelevant right for your average person. So I think keeping it simple, having something that’s just a guide is just something that’s going to induce less anxiety, be more meaningful to your average person than having a list of things that you’ve missed or overate.

Participant 5: So this is just an app that’s for a person to be able to get a sense of are they needing some type of Canada’s food guide recommendation? It’s not a tool for a dietician?

Facilitator: Not necessarily. It could be, but gold standard for sure will want all that information, will want that 24 hour recall data. We needed to make the changes, but this is more from the user’s perspective. Maybe with the dietician later on, but no for now it is for the general public in general.

Participant 1: I think that the fact that it’s not too in detail is actually really positive because maybe some individuals that look for those applications maybe be a little vulnerable or have some tendencies towards restriction or what not. And so I think that the fact that there’s not too much information and it’s not too overwhelming is actually positive.

And furthermore the other aspect is I think it has to be stated that it needs to be looked at globally, that not every single meal is going to be in that perfect proportions of the Canadian food guide. Obviously we’re not trying to like go against the recommendations, but just that we live in a reality where there’s a possibility that there’s a meal that you’re not going to have half of the plate that consists of fruits and vegetables and I just think that that’s something that individuals need to hear too without undermining the main message.

Participant 5: I agree.

Participant 1: The app, maybe this is how I am, but I’m not looking for an app to spend a lot of time in the app. Like I don’t want to, maybe this is just me, but I don’t want to track every day. I like a reference though so if it’s like comparing something or looking up something, but like to go in and track it it’s not going to be maybe of use to me, but if I can use it as a reference and looking at the app for something while I’m making my meal, but to go in and add details to me that would be a little more a novelty thing right? Like I would do it in the beginning, but I probably wouldn’t continue.

Participant 3: Yeah I guess my question would be like I look at the food guide and to me it’s not representing what your plate needs to look like. It’s representing at the end of, it’s a dietary pattern. Like I look at it as more of a pattern you know? Like when we had the food guide you know with a rainbow, we weren’t saying your plate had to look like a rainbow at every snack or meal right?

We were just saying you know, when you look at your dietary pattern you probably need a little more of these foods and these foods. So I’m wondering how, you know if you’re using a static app of a plate, you run the risk of maybe having people think that they’re supposed to achieve a certain look at snacks and meals which to me would be totally inappropriate.

It would be like saying oh like I can’t have an apple because it doesn’t look like the plate. Well I didn’t get everything on there? OK well so I failed. So like I’m just trying to understand what the concept would be because it can’t, to me if it’s based on you have to look like a plate and it’s a visual of a plate, I think we’re going to, to me that’s giving not the right message.

Participant 6: Yeah about what [unintelligible 00:22:19] said and what Beth just said, maybe it could be cool to have like some graph that gives you an idea of the proportion that you eat throughout the whole day or a few days or a week, you can maybe choose the option of what you prefer to be able to have like a more like complete idea of what you’re eating instead of just meal per meal or snack per snack.

Participant 3: You mean kind of like a bar graph so as they put their picture in it fills up you know the graph for the vegetables and then the fruit and then is that kind of what you mean type thing?

Participant 6: Yeah like a [unintelligible 00:22:57] with like the proportion that you need to respect. Like they say your line for the vegetables and the food needs to be twice as high as the protein or the [unintelligible 00:23:07] or something like that. I don’t know. I’m not doing the app, but I’m just thinking.

Participant 3: So like a sense of they’re half way through their day and they go oh my God, I’ve eaten all the protein rich foods I probably need, but I haven’t had any you know, fruits and vegetables yet so maybe I better focus – Like is the app trying to do that? Like to try and help people accumulate a pattern that would get variety and like I’m assuming it can’t prescribe because that’s getting into a whole level of consultation that an app shouldn’t be doing, but yeah so are you going to tell us a little more about what you’re thinking about?

Facilitator: No, not really. We’re getting your ideas on what you think it should be which is why we’re consulting dieticians as the experts today.

Participant 2: Is this app being created in partnership with Health Canada?

Facilitator: No. Not at the moment.

Participant 6: Yeah and the idea of like having a goal, let’s say like oh today or yesterday I didn’t eat enough vegetables or protein so like you can try to reach that goal so you’ll be more willing to use the app again and again and again to just you know, like having these little rewards to get your line correctly, something like that. So if you want to encourage people to use the app, like putting a reward system like that.

Participant 4: Almost like a pedometer, but for nutrition. You know like personally I use a pedometer on my phone and like it helps. It works, you’re like wow, I took three steps today. Like it makes you want to do it so if there was a similar [unintelligible 00:25:03].

Participant 3: I think rewards really help for making sure that the person stays on the app or goes on the app more often and that could be also a notification just as simple as well you didn’t put anything in your app now, are you eating something? I know we talked about that we don’t want to be on this app too often or just keep track of things, but if it’s been let’s say a week so hey, what’s up? Do you have something else to put in the app? So that could be interesting I think.

Participant 4: That’s a good point too. I think meal regularity, like how often, again there’s a lot of controversy around how often we should be eating, but you can choose like I want to be someone who eats every six hours. So you can choose that in the app. And I use that with patients right now right? Like you need an alarm or someone to remind you to eat given the situation. Like that doesn’t exist anywhere else so if the app could be helping you with reminding when to eat as like a little ding, like notification. That really helps.

Participant 5: Or not even to eat, but just to question yourself on eating, that could be also another direction. A question that’s not obligating to eat, but more on oh are you hungry right now? Because usually about four hours after your meal you’re supposed to be hungry or something like that.

Participant 3: Or if could be something completely different that gets away from it being sort of liable as a professional practice you know, you’re giving recommendations that they’re supposed to meet, but you don’t know anything about them in terms of well maybe they’re not, like why should they be eating then? Do you know what I mean? Like they’re not taking insulin, they don’t have to.

But what about just having an app that was more focused on if the goal is to encourage healthy eating patterns which is what the food guide is about and we know that you know people aren’t motivated to do a lot of these things around you know eating well together, don’t eat in front, you know eat mindfully, eat with other people, share your food blah, blah, blah. What if it was more of a motivational app around, and it was sort of a bit of [citizen? 00:27:13] science where the app was used as a way to collect you know how people prepare their meals so you get more of a sense of what is a plate, what does an eating style mean to somebody in terms of following the food guide?

You know and having people share their pictures of here’s, yeah I saw the food guide and here’s how I eat and you know this is like this is my culture and this is how I eat and there’s things all over the table and people reaching in to get things and then you have a sense of oh wow, that’s how you know they’re seeing the food guide and applying it in this situation or that situation or in this culture or I don’t know, like that would also be from like I know this is part of a research study so you know it would also be really interesting research to be able to get Canadians to contribute to what the heck does dietary guidance mean to them instead of saying oh we’re trying to tell you what dietary guidance is.

Well I don’t know the intent of the food guide from the office of nutrition, policy and promotion and so I certainly wouldn’t want to be doing an app to say that I know you’re trying to promote and I’m going to promote it here. I’d be more interested in trying to better understand like what is this dietary guidance? How are Canadians eating you know?

And it could be such a motivational tool and pictures could be shared, it could be a whole web place and you could be doing categorization of oh, look at all these different styles of eating and people could submit. You know like I just think you could gather a lot of, like it would motivate people to want to contribute and then see how they compare and contrast with other people and they might learn new eating styles and try new foods or there was like oh look at that picture, I wonder what that is and maybe people could post, you know even then post recipes or something. Anyways, I’m just thinking in a different way.

Participant 2: I think that’s a really interesting thing to have a community aspect to this because that could foster adherence, keep people engaged and interested, but also from a design and like financial viability perspective if you have a lot of these pictures you could probably monetise a lot of that data and sell it to somebody.

Participant 3: What do you mean?

Participant 2: Well if you have an idea of what people are eating all across Canada, you could probably sell that type of information to the food industry right and you could probably make a lot of money off of that.

Facilitator: Not the goal of the app at this moment.

Participant 3: Yeah I think there’s a whole privacy concern with you know, you’re asking people to submit information. You can’t sell it unless they signed, everybody checked off that you can sell my info.

Participant 4: I think it comes back to like we assume and we hope people eat a certain way, but we have no foundation. What you’re saying, Beth, is like if we were to, if people were actually submitting what they were eating, we would get a foundation of what Canadians eat and build off that. Like I mean like it could be completely different from what the Canada’s food guide is actually suggesting and this would reveal reality and truth and it can open up a whole can of worms which is super cool. This might not be the same act though, but I love that idea.

Participant 3: Yeah and I’m thinking that it’s not that it’s, it’s complimenting what the evidence has shown around dietary patterns that I think the food guide is trying to represent and so then you’ve got this fantastic country with so many different cultures and ethnicities and indigenous groups, like what a fascinating way to use an app to really motivate people to contribute to the ongoing nutritional health of all people in the population. Yeah.

Participant 2: Another point I wanted to make about keeping people engaged would have been or was to make sure to include positive messaging when people are out at restaurants so that they understand that yes you can eat junk food. It’s part of life you know. Yeah so maybe figuring out a way to incorporate that into an app.

Participant 5: Yeah I really agree with the positive messaging. And before I forget I would actually go back to what [Anton? 00:31:52] had mentioned and when he was talking about like graphs or what not. I feel like if people were talking about like bar graphs that there would actually be the possibility of presenting a pie chart and seeing with what you’re eating whether it be like spaghetti or whatever, a sandwich where you can’t visualize it.

And then I don’t know how elaborate it would be if the individual would have to write in what they put in their sandwich or what not or if they could just take a picture and it would be able to get out those key points, but maybe the individual could see that proportions and then for instance if they see that there is a lot of fruits or vegetables missing then maybe that would something that would encourage them to add an extra vegetable or perhaps go for a fruit that they have as a dessert. So I think that if there is a possibility of having that visualization I think that would be great.

Facilitator: So moving along on that the goal of this app will be the self monitoring question so yes, having that social media aspect of it would be super, super interesting for sure, to get those pictures and everything like that. But bringing it back to the self monitoring aspect [unintelligible 00:33:06] thoughts on what I’ve just shown you on this video here?

Participant 2: It seems to me that that this would require the user to already have a bit of foundational knowledge, that they must be able to kind of dissect what they’re eating. It might be a bit challenging. Yeah I don’t know, that’s my first impression.

Participant 3: And what about like a beverage, like if someone makes a smoothie how do they, you know it’s not a plate right?

Participant 2: Or a soup. A Raman.

Participant 5: Will still in a proportion wise, like the pie chart that we talked about so in my case I don’t think it would be a problem because we can see that what are the proportion what I think, I don’t get is that since we’re talking about the Canadian food guide and now that I see that you can adjust the different categories, I’m just wondering where the goals will be if we want to make Canadian eat more vegetable let’s say, well if it’s only a quarter of a plate we don’t need this goal so I’m not sure, I’m not saying it’s wrong because like we said with let’s say athletes or any other population it could be interesting, but I think for a general population I’m just wondering if it’s a good idea or not.

Participant 4: [Unintelligible 00:34:37] also choosing like dish. Like maybe this is, like we talked about before, like can I choose my serving dish? Is it a bowl? Is it a plate? Does it mention a glass or something? It’s almost too basic, but too much assumption that people understand nutrition. I don’t know. It’s not offering enough, but from what you’re offering it’s like even that might be too much in terms of knowledge.

Participant 1: Yeah especially if they have to choose what category goes in, I feel like if, with the old food guide there were like dairy products and somebody who download this app and they just don’t realize that they would have to put that in protein and that could be like a barrier right there. So I think that, as mentioned, like the person does need to have some foundational knowledge to be able to use the guide properly and actually like reap the benefits in the aspect that if they’re entering the wrong information they’re not going to be getting like the feedback that they should be getting.

Participant 2: I would almost, because yeah so we’re talking about the usability of this and the need for someone to have a certain amount of knowledge. I feel like it would almost be more interesting, more user friendly if there was a way to let’s say, take a picture have an AI analyze it and then have that turn it into a plate where someone could then say oh OK, so my hamburger and French fries actually represent these portions and this is the goal. So OK maybe there are some adjustments to make.

Participant 1: This is what I have in mind too to use artificial intelligence similar to Keenoa, but instead of tracking different nutrients we just track the groups and then we can compare to what we’re looking for and [unintelligible 00:3642] a way that is not, well like [unintelligible 00:36:50] or something that’s saying that this is good or bad food, but just that this is not the goal that I’m setting towards some of these groups.

Participant 3: Yeah I guess the tough thing is that artificial intelligence is limited in what it can do with people’s pictures right? You know if you have a plain raw burger with no bun nothing, it can recognize that you’ve got a burger and it’s just going to want to know what type of meat and how you prepared it. But you put it all together in a sandwich it’s really, like you have to say it’s a sandwich for then the AI to then kind of go oh.

And then so if I think back to Keenoa, Keenoa is asking food based questions to break it down for you, so Keenoa is doing that saying you know, was their cheese in it? Did you have mustard with it? Did you have a wholewheat or a regular? AI can’t do that, you have to ask. And again that’s that multi-past method of so they use it to then give you nutrient information, but it doesn’t have to. It can just be there to then break it down and tell someone well you know, how much am I getting of whatever food?

But my understanding is that the food guide is not telling, it used to talk about servings of food, like the diabetes education It’s not doing that anymore. There’s nowhere on that two page handout anything about how much you’re supposed to get in terms of servings of food in a day. So I think, my problem with seeing this plate as an app, is that it’s assuming or it’s saying that everybody has to have this every time that they eat that you have to have a certain amount of you know these protein rich foods, these grains and cereals or fruits. I don’t see fruits on there, it just says veggies, but it’s either or.

But there are many snacks and meals where you’re not going to have a vegetable or a fruit or you’re not going to have [unintelligible 00:38:46]. But there are many, many people that eat a meal that is a protein rich food with vegetables and there is no starchy food in there to speak of whether that’s a cultural thing, a weight thing. You know, a diabetes thing. And that’s a completely normal way to eat. So now we’re taking, we run the, there’s the problem that you can be encouraging people to eat in a way that’s not appropriate for their dietary goals and needs by sticking to this plate.

Facilitator: So I’m hearing that there could be adjustments made depending on the person and their needs?

Participant 5: And when Beth and also Sandrine was talking about goals, I think that like obviously for like the general population the girls is the standard plate. But maybe for an individual that has like never eaten a vegetable in their life, like they’ve just never eaten vegetables, well having half of your plate being a vegetable might be unattainable so maybe having the person set their own goals first and so that they wouldn’t be discouraged.

Like for example Beth was saying if they don’t eat grains in there well then we wouldn’t want the baseline of the app saying that they’re not eating correctly because they’re not eating grains. Or this person is not eating correctly because they only have one forth vegetables when for that person that’s like an accomplishment. So I think that the person would have to enter their goal with what they believe they’re eating baseline.

Participant 1: Yeah I get it. Maybe it’s an unpopular opinion, but I think it’s a good reminder to know that this where we start, this is where we want to go just to make sure that it’s not like a free for all education towards nutrition and make people feel that’s it more I can do whatever I want in this. I’m not sure I’m expressing myself correctly, but I just want to make sure that this app still has some plus value towards nutrition in general and with the plate also, but with other aspects.

So let’s say goals could be something else not plate related. Like let’s say cooking with loved ones and then you share your recipe with others and things like that. But nutrition wise I think the direction has to be clearer so it’s not like a free to all app. I don’t know if my idea is clear now?

Participant 5: No I think it makes a lot of sense what you’re saying that if the person can personalize it to be whatever the want for instance not put vegetables when they’re not necessarily following the optimal plate. And what you were saying just made me think of something really quick, I was thinking about the vegetables again. This plate, like Beth mentioned, only has vegetables, but perhaps the person like, OK so what I find problematic with this plate right now is perhaps the person has one fourth of vegetables, but for dessert they’re going to have a fruit. And so that would be incorporated and that would make half of your plate fruits and vegetables, but that plate right now is not representative of that. So I think that that could be problematic.

Facilitator: I’m going to stop sharing now so we break away a little bit from this plate and I get your ideas a little bit more. Anton, sorry about that. You can go ahead.

Participant 6: Oh I was just about to suggest that it could be a good thing for the person to start with is his or her own goals, but maybe with notification or something like that, maybe each two, three weeks or whatever like oh you reached that goal now are you ready to like step up your game and encourage people to put two new goals like that they won’t stick to the same level. And they might reach up with some point, like the optimal plate, yeah. And about the app is it only that? Would it be like that simple?

Facilitator: No, the goal is not for it to only be that, definitely not.

Participant 3: Yeah and I guess I just put in the chat box that you know we have to always remember that any intervention that we do, that we provide to people is an educational tool regardless of whether we want to use it as an educational tool or not, it is. So you look at people going to My Fitness Pal and I see many people go on and record on My Fitness Pal and they’ll come out saying you know, oh I can only eat 1600 calories a day. And that’s there, and it’s usually wrong you know.

Their kind of daily, but that’s what it’s educated them on and so all they’re doing is try to achieve a certain caloric balance day in and day out. And this is the problem with using that plate as a visual is, and we’re already speaking about it is that oh we’re supposed to get half our plate full of vegetables or half our plate full – No, it’s about a dietary pattern so you see we’ve already, by using the plate, we’ve already educated ourselves or taken it as a learning that that’s the goal for everybody every time they eat a meal that it has to look like that when I really think that that’s not what it’s about at all.

Facilitator: So solutions for that that you were mentioning earlier were kind of the bar graph that could represent the whole day or the whole week. Was there anything else that could potentially replace the plate at a meal?

Participant 3: Well it’s not, it’s letting them putting a picture on right? If the app is supposed to be, again I’m trying to figure out what is it supposed to be accomplishing. Is it educating them? Is it getting them to change their habits or is it just motivating them to eat better, but then what’s the message that you’re giving them to eat better so then that tells you what you want them, what tool you need. So I’m a bit, I’m finding it a challenge to understand what you’re, where’s the [unintelligible 00:45:23]?

Facilitator: It’s a self-monitoring tool so the participant will be the one making their own goals and monitoring their own progress with regards to that. So you guys were mentioning that they would define their own goals and it’s supposed to change their behavior long term to more closely resemble the Canada’s food guide. So that is the reference that we will be using, that generalized plate for a reference that they would strive towards.

Participant 3: So meaning you’re going to introduce your plate as a visual to them to talk about you know, and some kind of voice over or whatever around healthy eating even though you already have feedback that people don’t necessarily associate with a plate that. So that education is going to, that’s probably the most important piece of what you’re going to do because people have to make goals from that or I would say objectives right? Something that they can measure that’s realistic that has a time sensitivity to it etc. So it sounds like you’re still getting back to measuring something.

Participant 4: I think could you not change like if you just added another, like a bowl or something, you could kind of do similar to the bowl like adding layers just on things. I think it’s culturally important that we need to use a different dish. That in itself is more inclusive. But just like Canada’s food guide, like people in the public that’s what they see right if they see the plate.

So if that’s wrong, it’s not the most helpful tool because it’s more of a pattern than a plate, but that’s what people see right? That’s what people know. They don’t maybe access a dietician and they go online that’s what they’re going to get and they’re going to assume that that’s what my plate should look like in a way and maybe that’s wrong.

Participant 3: Yeah. Or maybe they say well, they’re not talking about me. Like that plate’s obviously, it’s not about for how I eat so I just do what I normally do. And so I think what would be important, I hope in your discussion groups you’ve got a bunch of dieticians of different cultures and ethnicities that you know can speak to that.

Facilitator: Yeah, definitely. For sure on that cultural piece it’s a huge aspect of this project and we’ll be integrating that for sure. I think having the bowl is a very interesting idea to have layers in it. We’d had a bowl with proportions as well so the layers could be really interesting to add in with that. And we had mentioned snacks before so how could snacks be represented on a plate method?

Participant 6: Maybe you can have a choice between a plate, a bowl and just snack and just select one that you like on that part. But that comes to the question how, is it like the better way to ask them to judge the proportion by themselves or just for them to enter the food and the app will do it automatically? That that for a snack will be easier. Just enter an apple instead of saying, oh is my apple covering half of a plate, quarter of a plate or whatever?

Participant 2: It could also just be simply just what, which of these groups does my snack represent? Is it a fruit or vegetable? Is it a protein food or is it a grain? Because I think, like Beth was saying, snack won’t generally be all three necessarily. Some people just have a fruit, some people are going to have nuts, some people might have chips, you know what I mean? And so it might be just to just isolate it as a single or bimodal. I don’t know.

Participant 3: That kind of then makes the educational tool right, of saying that it’s not about you’re trying to achieve a plate every time you eat. The plate is there to represent you know, at the end of a day or at the end of a week you know, are you getting a proportion of your intake from a little bit more from vegetables and fruit than you’re getting from grains and cereals, than you’re getting from protein rich foods, but they’re all important.

So maybe the app should be more you know, at the end of a week with all your snacks and meals, where do you get to, like I was thinking maybe that’s what Anton was trying to, was maybe thinking about. I’m not sure, but that you know, so that you get kind of like a graphic representation of the contribution, like Daniellele has said, of all the different foods you chose throughout the week to kind of say wow, you know, like here’s the pictures of all the vegetables and fruit you chose this week and here’s a picture of all the grains and cereals you had.

And here’s a picture of all the protein rich foods whether they were in a snack, in a meal, a big meal, a small meal, whatever. And then here’s all these other foods that didn’t really fit anywhere and then all of a sudden people go, oh my God, like I got most of my intake from chips, cookies, cake you know? And there’s not a lot coming from, like I’m wondering if you know, that would be, and that would show someone at the, and that would show someone at the, and it would motivate them to do it for like minimum three days to get your dietary pattern. How close does it come to the food guide?

Participant 5: I really like this idea because I think it’s really similar to I think what Ina said about if you had maybe a small portion of vegetable, but then you eat a food as a dessert. So then in this kind of graphic you will have this added fruit that will represent a bit more in your category of fruit and vegetables.

Participant 4: I have an idea. It’s almost like, sorry like reverse. Like if you enter what you’ve eaten, your plate at the end gets filled based on what you kind of, you fill the database with information and then at the end of the week let’s say it fills your plate for you being like you’ve [unintelligible 00:51:32].

Participant 3: Oh so that’s the education?

Participant 4: Like the reverse way. So it’s kind of lek using the plate or the bowl as the reverse based on what you’re input in the information. Also I had another thought, but it’s gone. Oh yes, here it is. I think where people get lost is if we assume they know what protein, carb and vegetables are, I wonder if you could do like a mini tutorial, kind of like you know how a lot of apps have like, before using this app, here’s a tutorial. Like understanding what’s part of your food. Is it like a food label tutorial or something so people know that like Greek yoghurt is a protein you know, kind of things like that. I don’t know, that would be interesting.

Participant 1: You have to support usability of the app to have tutorials or little information section.

Participant 4: If we’re expecting them to input information based on the nutrition they should have a baseline of like what nutrition, what nutrients are.

Participant 1: Or maybe the goal is kind of what you said before Gabrielle is that it’s reversed engineered so if people put their information in and it’s all kind of like a bit of a I don’t know, like a puzzle to figure out how, you know all the foods that you eat, where do they go to in the food guide? And so then you learn oh my God, milks, yoghurt, all those dairy foods, they’re a protein food. I thought they were dairy foods.

You know, and so at the end of the week, and they could say, oh at the end of one day I want to know how I did. Or at the end of two days or at the end of a week so they could choose different numbers of days to put it all together, but the app would then show their dietary pattern over the course of how ever many number of days they did it and if they did it and if they did it you know one day to the next day to the next day you could even do a comparison. I don’t know.

I keep coming back to what Anton was thinking about and visualizing the information of, and teaching them that wow, you know here’s what was changing your dietary pattern over the course of these days of these weeks or this. We do it in the spring, summer and fall and see how different your dietary pattern is and how well you meet food guide recommendations. So it becomes a bit of a challenge. You ask people to share their data so that then you can go wow, look at how this person is eating that is obviously eating a lot of Korean foods versus more, I don’t know, like I like that reverse engineering part is really interesting from an educational perspective that someone is then coming out of there.

And then they can make, you can say, so here’s how you compare to kind of the food guide recommendations of a dietary pattern and then it could maybe offer up some potential objectives. Wow, you know, if you had a little more vegetables most days you would actually better meet the recommendations or have you thought of choosing different grains and cereals that are more whole.

Like you could see how you could help them develop their goals around their healthy eating from their summary of their dietary data and then they learn, oh my God, now I know what the groups those are in and now I know where I got a lot of little or yeah. Gabrielle, I thought it was really interesting.

Participant 1: Perhaps there could be like two phases, I don’t know if we’re going in the aspect that like you want rewards or you have to go up in levels like the video game or what not. But like initially if the first part, the individual just entered the foods and then like Beth was talking, there’s like a partially like an educational part where they would find out OK in what categories these foods were in the second phase.

Then they would be able to do the plate that you showed us and be like OK I had this much protein, this much vegetable, this much grains because I feel there’s going to be some errors, like somebody, I don’t know. Somebody has a really big craving of pickles and they’re eating pickles as a snack. I feel like if they would arrive and they just wouldn’t know in what category to put it in because a lot of people don’t even know that it’s a cucumber at the base. And can you even put that in as a vegetable?

So anyways, I feel like if there’s a part, or at least a possibility for the user to choose, if they feel comfortable and they know what category foods go into then yeah, sure they can go right away and do it that way, but if somebody isn’t comfortable with that, the fact that they could just enter the food and that it would process it for them and put it in the correct group. I think that could be helpful.

Participant 6: I have two ideas, first one to go with Ina’s actually. Maybe there could be a trial at the beginning where like you enter all the food you eat. Then you have like an option of three different plates so you ate that burger. Do you think that is your plate? Or is that your plate with different proportions? So like that the person will be more, like will train himself to identify the right proportion according to what he’s eating. So then they’ll be like about to do it by himself.

And the other idea was, I don’t know, it was more about like rewarding and like having a better visualization. So let’s say at the end of the week you can compile [unintelligible 00:57:02]. All the amount of protein, the amount of vegetables and fruit and the amount of grain and put it in a way that like you can see the amount. Let’s say like on the back of a truck, like a drawing. So like you ate that pile of vegetables, you ate that pile so like you can see like oh that much, I ate that much meat or I ate that much grain and that much vegetables and fruit.

It could be easier to have a better understanding of the amount of food you’re eating throughout the week. I’m thinking about that because of like workout things so let’s say you’re doing like five push ups a day, it’s not that much, but like at the end of the day, at the end of the week you did like 35. So it’s like oh, that’s a fair amount so maybe with food you can do something similar for people to be more aware of what they’re eating.

Participant 5: I like that too and I like that like having two parts. Like the first part would be to, so let’s say for the graph you have a 30 percent of your dietary habits that is completed and now we need a bit more data to make sure that we have, well we need more data to put a dietary patter and then when we have that, now we can show you some results.

So now that we know the person is on the app quite often, and that we have enough data and then when we have that, like the plate or the bowl or whatever it is could be separated in groups. And a bit similar to what Anton said, so let’s say the protein, a slice of pie could be separated in let’s say red meat, in veggies alternative or more on dairy and things like that.

And depending on that a person can choose to, how they can change or modify their habits just like the last category that we don’t talk about often, like more of the other food. If the person realizes well about half of my everything that I eat maybe can I just reduce it a bit or is there any other [unintelligible 00:59:31] pie that I want to put more effort in. So I like having the app on two parts so one more gathering information and the other one well now that I know what my dietary habits look like, how can I change it to have some goals that are my own?

Facilitator: How would you guys sees that assessment piece working? So having that first part of the app, would that be entering?

Participant 5: Yeah like entering the info, it could be like pictures again and something with more artificial intelligence that knows that this goes in protein and this goes in carbs. Because like we said I think it will reduce the factor of errors because some people don’t know where to put in which category foods are. And yeah, depending on how many plates will need, this will be more on your part to know how many data do you need to build a pattern, if that makes sense?

Participant 4: You could also have like at, or from the app, where you could have it being like is this a carb, protein or fat and then putting things that you wouldn’t think would belong in those categories so people can go in for the non obvious ones. Like meat people know it’s protein, but they don’t know like my Greek yoghurt example. So maybe just having the ones that people wouldn’t really know in that section so that they can make a better decision.

Facilitator: Coming to that little information button, that they would be able to find more information about each category?

Participant 4: Yeah.

Facilitator: And how about those other foods that we’ve mentioned? So things like fats, things like desserts, condiments, the pickles that you’ve said before, seasonings, added sweeteners, where would those fit in the application or on the plate itself?

Participant 6: Well you could have like a processed food section or something like that for people to give it on the Canadian health guide. It’s said that you should reduce the processed food so maybe it could be a good point to have that section on the app for people to have like an idea of the amount of processed food that they’re eating.

Participant 4: So adding like is it carb? Is it protein? Is it processed?

Participant 6: Yeah.

Participant 4: Something like that where people – Like where does mustard fit? Right? And they go oh, it’s in the processed section.

Participant 1: I don’t know what you guys think about it but unless the person uses huge quantities of ketchup or what not, I’m thinking personally like let’s say I’ll eat a burger in a week, I feel like if that wouldn’t affect the complete summary that I get at the end of the week. If I put like one sweetener in a coffee that I take one time at a restaurant or what not, I don’t think that would affect it in a, like it wouldn’t come out in the graph at the end. I think it’s if somebody consumes large quantities of certain ingredients.

But I’m not talking about cakes or what not because that obviously was going to be represented, but like I’m more thinking of the condiments or stuff like that. Or the artificial sweeteners that don’t really fit in. I feel like there should be a threshold where the person just has like a little bit, it wouldn’t really come up, but if the person has it like every single day in their coffee then maybe it would be worthwhile it be mentioned. I don’t know what you guys think about that.

Participant 6: I think it’d be a good thing to be [unintelligible 01:02:43], but like you said [unintelligible 01:03:43]. So let’s say the app will count it every time you enter it, but if you’re not reaching five grams in your week it won’t appear. Like it won’t be –

Participant 4: But how would it appear? I guess the question you’re asking is like what on the app is going to show, like a plate, but how do you count for the other foods and beverages? What kind of, what [unintelligible 01:04:08] are they going to use to represent processed food, alcohol, hot dogs?

Participant 5: I think what could happen is like a two fold kind of picture. I don’t think I’m expressing myself clearly. So for instance at the end of the week was somebody eating like pizza and going out and having I don’t know, hot dogs and highly processed food, a whole bunch of crackers and stuff like that. Like OK, this is your plate, this is how much fruits and vegetables, proteins and grains you have.

And then you’d have like a light grey shade, like half of your plate over that or just another plate and like half of what you ate was like highly processed food. Or one fourth of what you ate was highly processed food. I don’t know if that makes sense? Like having the plate with the food groups and then having another plate saying this is the proportion of food that you ate that had highly processed foods.

I think it has to be clear too that it’s highly processed because somebody that has yoghurt, that’s a processed food, but it’s not necessarily considered highly processed and I think that that’s important to maybe distinguish between like [unintelligible 01:05:29] highly processed and just processed.

So either it could be like two plates or it could be the plate and then there could be something that goes over it in like a light grey for you to see. I just think that if you go over it and it covers only a part of the plate maybe the person is going to be confused, was it like all my grains that were highly processed so maybe that’s why it’ll be worth while to have it beside.

Participant 6: I like the idea of over the other plate, but the app will have to be really precise, well not really precise, but like to cover like the part of the protein that was processed, the part of the grain that was processed, the part of the vegetables that was processed because you can’t eat like fuller vegetables. Like you can have half a plate of vegetables, but if they’re all pickles and condiments and stuff like that, it might be useful to know that yeah, you’re getting your vegetables, but you’re not getting the best version of the vegetables. So same thing for meat or for grain.

Participant 2: I’m wondering if it’s even worthwhile to count that in the first place? I’m not sure, like not just in general like fast food, like we’re trying to get people to eat a certain way. I don’t think we should be really tracking percentage wise or numbers wise. At most maybe just a little question, like have you had anything you didn’t prepare yourself this week? Or how many times? That way they could maybe get an idea, but not really going into the details of what and what kind of food was it and that kind of thing.

Participant 3: I think we have to really careful. Like if the app is supposed to be representing the dietary guides of Canada’s food guide, you know when they’re talking about processed foods, they’re talking about foods that have added sugar, fat and sodium. So that’s like the difference between I have plain yoghurt and I have sweetened yoghurt. I have regular milk versus chocolate milk you know?

By saying that people eat out and you know it’s all junk food, well the reality is is you can break a pizza down into it’s got a crust, it’s got cheese it’s got tomatoes, it’s got vegetables on it. In one way you’re going to say that’s fast food, that’s not healthy. Well, or a hamburger, but there are many people that are living off these foods that are very healthy because it’s not their entire diet.

And then you’ve got other people who are you know, have all the money to buy all the great foods and they’re chowing down tons of ice-cream, they’re eating cookies all the time, they’re spending you know, lots of money on a mocha Frappuccino with tons of sugar in it that’s you know, and a cookie. So I think we have to be very careful that we’re not bringing in disequities in health here in what we’re trying to get the app to do.

Like it’s supposed to be, I would assume, something that is improving equities in health. So we need to think about the types of, the varying types of foods that people are going to be eating that are varying degrees of processing, but you know for sure, if someone’s eating foods with a lot of added sodium or a lot of added saturated fat or a lot of added sugar, you know this is going to be a problem.

Like I keep going back to the, like what the food guide has on the back you know that you showed us at the beginning and it’s like the front page is to me, again I’m going to keep coming back to it, it’s talking about a dietary pattern. It’s not talking about what my plate needs to look like. It’s talking about, to me, what my dietary pattern needs to look like. And then everything else is about healthy eating. Like how can I cook more often? How can I enjoy my food? How can I eat meals with [unintelligible 01:09:38] and be mindful of my eating habits?

And that’s the dietary guidance. So how does the app, to me I’d be saying how is it capturing that dietary guidance in helping people make goals around the dietary guidance, around wow, you know what? I want to be mor aware of marketing, food marketing and oh my God I’m buying cereal that has all this marketing to children on it. I never realized it and oh it’s also really sweetened.

And oh my God oh that means it’s not the best choice because it’s added sugar. Do you know what I mean? So now you’re putting in a whole bunch of things into that app around reading the label, you know looking at the marketing to these children, choosing less processed foods, trying to have wholegrains more often, throw fruit on my cereal instead of buying sweetened. So I’m just, I’m kind of sort of challenged with if we want it to be reflecting the guidance and the food guide, it’s a lot about behaviors that then influence intake. It’s not about specifically about intake.

Facilitator: So how could those behaviors be captured within an application?

Participant 3: I guess that’s what I’m kind of throwing out to people. We kind of got stuck into sort of the you know, nutrients as dieticians which we do when really, at the end of the day, we know when you start to make more food you eat mindfully, you eat in the company of others, you start eating better. You know even if you’re eating at a restaurant with a whole bunch of processed food you start eating better you know because you’re a little bit more mindful. We see that over and over right, in the behavioral research.

Participant 2: Would it be fair to maybe just prompt people when they log something, well yeah when they log something in, did you prepare this? Did you cook this or whatever and then if it’s a yes or no then you get a you know junk food [unintelligible 01:11:43].

Participant 3: Did you eat this with somebody? Were you eating this with somebody? Dd you enjoy it? Did you cook or buy it? I don’t know like maybe –

Participant 2: Did you eat it without watching TV?

Participant 3: Yeah.

Participant 6: That’s right, yeah.

Participant 3: I’m eating it while I’m putting it on this mobile app.

Participant 2: That’s right.

Participant 6: Well it comes down to where the app, what is the goal of the app because that’s true that with Danielle is that like maybe we don’t need to show the people the amount of processed food that they ate just because we don’t to feel them bad. But if it’s about like setting your own goals and things like that, maybe one of the goals of the person is to reduce it’s processed food. So then it’ll be useful to show it to the person the amount of processed food he or she is eating.

So it’s, yeah for me it comes down to what’s the real goal of the app. And like if it’s the whole Canadian healthy habit, well we’ll need to assess also the behavior and not just the nutrient part. And with multiple questions like that once again you can put your own goals depending on the categories. Like nutrients, intuitive, yeah. Processed food and all those things. Like you can select your own goals step by step. So what are the goals?

Facilitator: Of the app itself you mean?

Participant 6: Yeah.

Facilitator: Yeah. [Unintelligible 01:13:25] dietary changes to follow the app more closely so that’ll depend again, as you said, on the person. If what they want to focus on is getting that plate in then that could be something that the app would have. If their focus would be more on the mindful eating or on the label reading or on the reducing processed foods then that could be something as well.

So you mentioned the check lists of pressing yes or no. If that was something could be based on the recommendations of the back side of the guide? Any ideas on that back side of the guide how that could be tracked and how that could be implemented?

Participant 3: Yeah and that could meet the, you know I pasted into the chat what they say on the food guide around healthy eating recommendations that it’s more than the foods you eat. It’s about where, when, why and how. So you know it would seem to me that those would be you know, the other things to so you’ve got the what, the where, the when, the why and the how. Can the app track those five things? How could it do that in a way that then would help people improve their eating habits because you would need those five things.

Participant 4: If we think about kind of the reverse plate method that I talked about, maybe at the end of the week it can say like how many times did you cook a meal, who did you eat with or how many people did you eat with? I don’t know, just so that maybe in this case like day by day, meal by meal, but more of a general. You know you might miss, OK you might miss, not exactly but it gives you an overall idea and it helps you reflect [unintelligible 01:15:05] –

Participant 3: Like a healthy eating pattern.

Participant 4: I didn’t eat around anybody today. I didn’t eat around anyone all weeks. So just having those prompt questions at the end to summarize your experience with the plate method or the plate.

Participant 2: I think something that could be maybe interesting could be asking them like so like the whole lot when, where, why right? So we know the what. How right? Was this on the go? Was this at the table? Was this maybe at your desk at work as you’re still typing something, you’re doing your report right? So there’s probably a lot of different questions we could ask to get all those information that way at the end of the week or the end of the month, you know the app could then say oh OK, so you know you’re really, you know you’re including a lot of vegetables, that’s great. You know you’re at home, you’re sitting down with your family, fantastic. On the weekends you’re out with friends socializing, but at work you’re not taking time to eat. You’re either on the go or skipping or maybe you’re eating in front of your desk right? And so that could be also a way to get people to be more mindful of –

Participant 3: Yeah. You see that’s very very cool because then you kind of, it’s all about patterns, it’s about your behavioral patterns and at the end of a period of time you know you get in a set, the app gives you feedback on what you ate over that period of time, you know when you were eating it, how you were eating it. And then it could do some kind of basic level of analysis just like Daniellele said around yeah you know what? When you’re eating by yourself you seem to be eating a lot of processed food you know. Or processed food are your number one choices.

Or Anton, when you’re working seven hours in a row and you haven’t eaten for seven hours, you’re always having caffeinated energy drinks after. You know, like I mean I don’t know. I’m not saying you have caffeinated energy drinks, but I’m just saying, like if the app, and then they could say wow and then it would flip and give you the advice around oh well so, you know what could you, how could you, asking them what could you do to improve that habit?

You know to change that habit to give you a healthier eating pattern. Oh I could make sure so then you know Anton would say oh gee, like I go seven hours without eating and then I don’t even remember eating, but oh then I just drink caffeinated energy drinks. I could bring a snack, what could I bring? Oh I could bring a piece of fruit you know. And then all of a sudden, right like he, so they could make a goal of –

And oh that would meet my think of like it tells me I don’t really get any frit throughout most of the week so it could chop off it could meet a number of healthy eating recommendations by just all of a sudden saying oh I’m going to bring an apple when I got to the computer lab to work for the morning.

Participant 6: Yeah. I think what will be important in the app, if you have to enter these like five components each time is maybe to have two or three, like three, four options for each.

Participant 3: Yeah, like a click, click, click, click.

Participant 6: Yeah so let’s say where, you have home and in the car, desk, whatever. But only like two, three, four maximum because it’s too long for them to [unintelligible 01:18:37] each five and you have like 15 option or you have to enter it in like typing. So it’s going to be like three options for each general option then it’ll be like fast food to select.

Participant 3: And you can determine those pretty easily with like pilot testing to get the multiple choice derivatives. That would be the most commonly given ones and then you always leave the fifth one as an other so the NF fillable thing right? So now you know you’re getting wow, yeah that’s really interesting. I want to do this. I want to develop this now.

Participant 1: Maybe you can have a certain number of pre-sets that the person puts in ahead of time. You know it’s like OK what days do you work? I work these days and what meals do you have? Or whatever. That way, when you put something in it’s like at work today did you take time? Yes or no. And that way it kind of can perhaps make an assumption of where you’re already having this meal based on what you’ve told the app.

Participant 3: Ah so the artificial intelligence could then learn about your behaviors and then start to question your behavior to push you to a better behavior if it’s not potentially the greatest behavior. Is that what you’re saying?

Participant 1: Not quite, but I like that idea too. But it was more along the lines of if the app already knows I’m at work from Monday to Friday, on Monday at lunch when I put in my app, sorry my meal, it wouldn’t then ask me like are you eating this at home? Or are you eating this at the restaurant? It knows I’m at work so it’s just going to ask me perhaps another type of question.

Participant 4: Or you could be at different places at work. Like I could be eating with friends at work, in the office, in the car.

Participant 3: In front of the computer, in the lunch room.

Participant 4: I was going to say that like I think it doesn’t change it a lot, you could just probably leave it as an option because it could probably change a lot for people

Participant 1: [Unintelligible 01:20:45] your own option.

Participant 5: But having that like general baseline questions is interesting, not necessarily when there is going to be those options to put in that it’s like very fixed because like you said you can be at work, but go out or what not. But just having those general questions because if you’re single, alone during the pandemic, well it’s going to be really hard to share a meal with somebody. So maybe the AI or what not isn’t going to prompt you in a way of being like you’re never eating with somebody because you’re living in an apartment alone whereas if you entered that like you have a family and you’re writing that you’re always eating alone well maybe there could be a prompt.

Participant 3: Or say [unintelligible 01:21:31] pandemic single eating. Pandemic lonely eating. It could be, that would be useful information too right. Then you’d get, you’d know who’s living by themselves right?

Facilitator: I’ve read a lot of articles based on that data for sure. So coming back to that what question, who would you recommend that people write or note what they’re eating in this type of application?

Participant 3: Well I don’t know if it’s any different. I keep seeing it as a way of again, just taking pictures of the food and giving general information on the food and then so the AI, if people don’t answer the food items and they just do pictures then the AI could do, could try and maybe group things as to here’s what you eat at, you know these meals here’s what you eat at breakfast, lunch, dinner and give people a sense of they can look at it and make their own goals from it right? Whereas if they entered food items and chose from a database it could give them more specific things of at the end of the week your dietary pattern consisted of, you know?

Participant 4: I think it goes by [unintelligible 01:22:53] very at the beginning how people enter the information about food in the app and if this is the question, to be honest I don’t have any other ideas other than Keenoa because I think it makes it easier for a client just to enter something a bit faster and to add more details as a dietician that we know is important, but maybe it’s not important for them. So let’s say the type of bread or the cut for the meat or something like that.

So for me, something similar to Keenoa and to recognize the food having [unintelligible 01:23:31] so that it’s not long enough for the person to enter the information will be very important because when it takes too long people get discouraged and then they will just leave the app and just by experiencing private practice, when it’s been a while that we’re working together, I just tell them to take a picture and not even enter the information anymore because we’re just going to look at like the pattern and not on specific things. So I just want you guys to keep in mind that entering information, doing recuring or things like that takes a long time and people would get discouraged very easily.

Participant 3: What if it was just a check list? Like did you have a vegetable? Did you have a fruit? Did you have a –

Participant 4: It comes back to the question of making knowledge beforehand or what the protein will be like.

Participant 3: Yeah with the picture. So that’s where you could use the picture to say you know it’s got all the foods in it. You could say did you have any of this? Did you have any of this? And then add all the other, like what about condiments? Like it could just be a click, click, click that they do for each time they eat and so that’s the what.

And then they get the sort of more intuitive questions around the where, the how, the when, the why. Right, like it doesn’t have to be you know, dietetic details, it’s just more does the pattern, because our dietary patterns, if they’ve got grain products, vegetables and fruit, protein rich foods and you know, like if you’ve covered those simple bases you’re probably doing pretty well. Right?

Facilitator: And that photo data would be super valuable as well.

Participant 3: Yeah.

Participant 6: And I don’t know if Keenoa is doing that, but because from what I heard, I think Keenoa is doing a pretty good job for entering all the food you’re eating, but is Keenoa like remembering let’s say like you enter a burger.

Facilitator: Yes.

Participant 6: OK so next time you can just select.

Participant 1: Yes. So let’s say you have a left over, you can just say what is the same thing as before yesterday or something. Maybe another idea, like if talk about the checklist, just to make it a bit more simplified could be something. So let’s say you eat some bread, but then it opens up to the kind of bread instead of having something so OK white, brown or rye, whatever. It’ll just be too much of a long list so having like folders that opens up when you click on a specific detail.

Facilitator: And then to get to that how question. So how are they eating? What would that entail?

Participant 3: Yeah so are you eating with others? Right? Like that’s what we want to find out. Are you eating with others or are you eating by yourself? I mean that’s simple. Are you eating with others? Yes or no.

Participant 6: [Unintelligible 01:26:52] with the how or if you’re like eating in front of a screen like TV or a phone, that’s part of a how right?

Participant 3: That’s where.

Facilitator: Where could be table, kitchen, work.

Participant 3: So like you could be saying I’m at home and then it could be a drop down so are you eating at the table? Are you eating in front of the TV, your computer? Or eating at work in the lunchroom, at my desk, in front of the computer. Like you can get like it could be as granular as you want it to be.

Participant 1: Well maybe it could just be as simple as are you focused on your food? Without maybe collecting all that extra data.

Participant 3: As long as that captures if people understand what that means. Because I could say yeah, I’m eating in front of my TV, but yeah I’m focused on my food.

Participant 4: It’s a little to open ended.

Participant 3: But those drops downs, like they’re easy, fast. You just like when you get those on a survey tool you just go click, click, click, but when you have to go oh God, I’ve got to think about it, write it in.

Facilitator: And then that why question. So why you’re eating.

Participant 3: Yeah like that’s the whole intuitive eating right?

Facilitator: Yeah.

Participant 3: So those could even be taken from Keenoa.

Facilitator: We won’t copy paste.

Participant 3: Well it could be an intuitive eating questionnaire.

Participant 1: Yes, but it could also be for other reasons that maybe are not present in Keenoa so I don’t know every answer by heart, but it could be something like because I was feeling x and y and z or just because somebody came over or it could be there’s plenty of other options out there I think.

Participant 6: You can just narrow it down to three types of hunger and describe this type of hunger in the app somewhere and then when you go –

Participant 3: Yeah if you say I was hungry, like I ate because I was hungry, yeah.

Participant 1: I think that would add to like finish off that, I don’t think I would put that after every single thing somebody would eat because that would take a long time, but maybe key points or like three random times during the week or something like that because if it would be every time I think it would be pretty lengthy.

Participant 3: It’s actually really quick and people get used to it. They just go yeah, I was hungry. You know was I super hungry, a little mildly hungry whatever. They just click, click, click or I ate because it was nothing to do with hungry I was bored. And then they get a real sense of being able to make a goal right because you can link, the tool can do the data analysis and link back that intuitive eating of every time you’re angry you’re eating this or when you’re bored you tend to be eating more in front of the TV. Right?

Like if people, like if it’s supposed to help people have healthy eating habits, you kind of need to ask those questions each time to help people make some smart goals. It’s not challenging and it’s not, it doesn’t, few people are going to say oh I got fed up answering those questions. When they do it for a couple of days and then they see the information they get back, they’re like oh my God, I never realized that you know when I wait too long to eat and I get super hungry, I eat way more than I probably need to and then I feel super full after and then I don’t, you know.

So it helps piece the things together. Like that would be very cool if the tool could do that then dieticians could be cancelling people and then send them home and say hey, go and use that app because it’s really going to help you see if you actually can meet your goals of you know, eating when you’re hungry, but now when you’re super hungry. So before you get too hungry. Or yeah. That would be a super little app to have to help with behavior change.

Participant 4: I think a good app, how people gravitate towards it in a way that like I see more of a summary at the end of the week. Like I want to enter information during the week that at the end of the week I’m asked these questions to get my total assessment in a way. Like I think I agree if it was every meal like I won’t do it I don’t think. If it was at the end of the day maybe, but I think at the end of the week I’d rather be asked how many meals did you eat alone? How many meals did you eat, like just asking more those types of questions because I think it will be again giving you a summary of a pattern than me having to put so much information in. If I’m eating six times a day which I might be, it might be a little too much.

Participant 6: Yeah maybe like to be in the middle of the two, maybe the person can set up a [unintelligible 01:32:03] what is his goal? Like do you want to assess which of the five questions, the what, the when, they why, the where? So you’ll be asked about these questions when you enter. So when you’re ready, like let’s say you just start with what then you see all the information that you’re getting back. It’s like OK that’s pretty useful. Now I’ll try the what and the where.

Participant 5: Yeah I like this idea too because the person can choose to only have one aspect at the beginning. If they like the app and they want to like go up a notch, or have like a badge you know, we talked about goals and things like that that people can reach more like a game. If it’s set in let’s say five parts and then you went to the first part and you worked on that while you have this page and then you go through the other ones it could be interesting. And then we can reduce the quantity of the question and the question that people will answer will be questions that they will be looking forward to answer because it’s really correlated to their goal that they’re trying to reach.

Participant 3: Yeah and I think from a public health perspective which I’m assuming this is for. It’s not for people who can afford to go and see a dietician or who have a diabetes education program that they can go to for free, it’s for people, like it’s supposed to be for Canadians right? And so you know if, so it has to be able to give them some direction around their healthy eating or around their eating habits to help them you know, nudge them in a direction towards something that’s going to be more beneficial to their overall health.

So yeah, you know I think this is fantastic if this tool could kind of get around detailed analysis and keep it at a level of the sort of the general what, when, why, how, when so that, and link all that information so the apps, the way to link all that information and it can, the more detail you give the more linking it can do for you and the more granular suggestions it can give to you.

But even with a minimal amount of information can it, you know of the five things, if you just do it once can it give you some direction? Nudge you in some direction. And then that’s really useful for people who’ve got marginal health literacy, who don’t have the skills to enter a lot of information or the motivation and then it’s also there for the people who are super motivated and have good skills and have the technology to be able to spend time and you know give all the detail.

And it can give them more info so it’d be, I think we have to keep that population, public health approach in there because it’s not to replace a dietician, it’s for those people who don’t have access to a dietician or nutrition professional. That’s the 99 percent of the population.

Facilitator: Exactly. And I think that was a great summary and I’m just being mindful of the time.

[End of recorded material]
