# Supplementary material for: Perceptions of self-monitoring dietary intake according to a plate-based approach: A qualitative study
Source: PLoS One. 2023 Nov 28;18(11):e0294652. doi: 10.1371/journal.pone.0294652 (PMC10683993; doi:10.1371/journal.pone.0294652)
Supplement: S5 Appendix — (ZIP) [file pone.0294652.s005.zip › Anonymized RD Focus Groups/iCANPlate-RD-Focus-Group-5.docx]

**iCANPlate-RD-Focus-Group-5**

[Start of recorded material 00:00:00]

Facilitator: This is the RD focus group on July 31^st^ at 11am. So, first off, we're just going to go over a few general questions. So, first, do you suggest following the plate method as illustrated by the new Canada’s Food Guide would go to your clients?

Respondent 1: Yes.

Respondent 2: Yeah, me too.

Respondent 3: Yeah, I would say it’s an easy visual. Yeah, yeah, definitely talk about it at least.

Respondent 4: Yeah, I would say generally, yes. But I would always like tailor according to the customer’s needs.

Respondent 5: Yeah, absolutely. I think it's definitely a good like starting point, at least to gently kind of ease them into what they need.

Respondent 2: Yeah, I was going to echo what Madeline was saying was just yeah, it's good for people who are kind of getting started with learning what healthy eating is. That's a good starting point. And then building on top of that to add extra specific recommendations, but it's a good proportion visual.

Respondent 7: Yeah, I use it a lot, especially with patients with diabetes. Like I see a lot of different patients but I find that if I'm not doing something super specialized, or if they don't have a huge knowledge base on nutrition, it's always a good starting point.

Respondent 8: Yeah, agreed.

Facilitator: So, moving on that, what makes it easy or hard for your clients or patients to follow this plate method?

Respondent 2: I’d say snacks. I’ll say snacks is kind of a confusing part for them.

Respondent 5: Or mixed dishes as well. They'll get confused, especially if they're most often eating mixed dishes or mixed whatever that's most culturally relevant to them. They'll find it hard to know what the proportions are of each of the food types.

Respondent 2: I’ll second that, for sure.

Respondent 4: Yeah, same here.

Respondent 3: I’d say for me, so, what makes it easy is that it's a visual, right? And it's not like you're counting the number of servings anymore. But what does make it hard, I do live in a rural community with a lot of low income. I do see a lot of low income populations and the half plate of fruits and vegetables is not realistic for most. So, just kind of back to that first question, I do kind of tailor it to what's realistic.

Facilitator: Any more thoughts on that? Well, keep those in mind while we're going to be discussing the applications and different modalities that you're thinking of that could be problematic. So, I'm thinking the mixed dishes, the snacks. So, keep those in mind, those little points after and I’ll move on to the next question, which is which diet tracking methods or applications have you used? Or do you use with your clients or patients?

Respondent 5: I use my MyFitnessPal. I think that's the main one that I've been using or my customers has been using.

Respondent 4: I like to use Nourishly.

Respondent 2: I tend to use Rise Up a lot. Very similar to Nourishly. In the past, I have used MyFitnessPal Pal with clients. But yeah, I've gradually kind of transition to Rise Up.

Respondent 1: I normally use either My Fitness Pal or Chronometer, if someone is looking for kind of the more specific micronutrient content or like specific things that MyFitnessPal doesn't provide. And I still have a decent number of clients that really like pen and paper, that they just journal by hand.

Respondent 8: Yeah, I second that I feel like majority of my clients prefer but maybe it's because we do tend to see a lot of older people. They do prefer just pen and paper. We have logbooks for them if they want, or otherwise My Fitness Pal for me as well.

Respondent 7: I'm surprised at how many clients in their 20s and 30s declined when I offer them suggestions, or like, “Do you want to use an app?” They’re like, “No, I’ll just write it down by hand.”.

Respondent 5: Oh, yeah, I was just mentioned that, maybe it's simply just take a photo and then we can talk about during the session.

Respondent 1: I've used My Fitness Pal and Chronometer. I think Chronometer can have too much information for people. And then another one, I've just used like an app called Habits, if someone's tracking something specific, like if they had to track like fluid intake, I've used that just especially if they have an eating disorder background. So, if there was one specific thing they had to track so that they don't have to look at all the calories and everything. You just set your own goal for whatever you're tracking, and then you just can add that.

Facilitator: What are the pros and cons that you've noticed with these tracking methods that you've mentioned?

Respondent 1: A lot of the nutrients aren't logged in the databases. So, you think you're adding everything correctly, like especially on Chronometer, if you wanted to look at a specific micronutrient, unless you're only adding basically raw foods, the database won't really be accurate for those things.

Respondent 3: I find that worse with MyFitnessPal, not having all the data uploaded, and especially because like just members of the public can upload their own information into the database, that you really have to be careful about the accuracy of what you're choosing.

Respondent 4: Probably my biggest thing I hear from clients, when I was using more, like MyFitnessPal, I will also have clients that use it too, but it can be kind of overwhelming to use as well just with let's say they're having a mixed dish and having to input everything in.

There's a little bit more of that upfront kind of work to track it in that way. And sometimes that can just be too frustrating and you have like that all or nothing mentality where it's like, I'm just not going to track it at all.

Respondent 5: And also, the unit of measure. So, customers might just more comfortable with the volume. But in MyFitnessPal, it’s also always in grams or like there's some unique measure that are not commonly used in their daily life. So, the conversion could be inaccurate too.

Respondent 2: I'd say, I guess one easy part about using apps like MyFitnessPal would be to scan the barcodes. That makes it easy for people. But then if you can't do that, then try to find the right product that matches what you're consuming will be pretty difficult at times.

Respondent 7: Another easy thing, if you're eating similar meals very frequently, the apps that save your most common foods, I think that makes it a lot easier for people to track because they don't have to look it up every time.

Facilitator: Yeah, great points all around for sure. So, do any of you know any diet tracking tools, paper or app, based that resemble the current Canada's food guide or the plate method?

Respondent 1: I'm trying to recall. I don't know if it was dairy farmers, they had…sorry, I think there was like one app like years ago that I looked into that was kind of – but now I'm thinking is probably based on the previous food guide.

Respondent 2: Yeah, I remember that one. I think that was based on the old food guide where you could just track like food guide servings without all the other detail. I found that when confusing, because I don't think it was very clear as far as how much a serving was. And what was the other thing? And I don't remember what the goals were as far as like if it told you what you were targeting, like how many servings you were aiming for. It's been years since I've looked at it. So, I don't really remember.

Respondent 6: I’m going to Google right now and see. I don’t have any in mind.

Respondent 7: Nothing comes to mind for me. I don't know. Yeah, I know any other one.

Facilitator: I mean, that's good. That's kind of why we're here. Wanting to develop an app on the new guide because we don't know of anything out there. So, if you do think of anything that looks like it, just let us know. That would be great. But I think it's a good thing that we're on the right track in that.

Showing the app’s prototype

Facilitator: Excuse me?

Respondent 3: I was just going to say in terms of like page two of Canada's Food Guide, like with the being mindful of eating habits. That's one of the reasons why I do like Nourishly to just kind of like raise awareness, I think Rise Up has very similar things. But yeah, once again, it doesn't really track the half plate of produce and stuff like that.

Facilitator: Yeah. So, do you have any rapid fire comments just after seeing this?

Respondent 2: I like the simplicity.

Respondent 1: I'm wondering if the grain and the protein are too close together in colour.

Respondent 3: How does this compare for like colour blindness? I'm not sure what the guidelines are with that.

Facilitator: Yep, definitely a great point for sure. We could have multiple different options of colours and maybe put patterns on a few of them as well. Those could be difficult to read them.

Respondent 3: Something else that I'm thinking about when I'm seeing that is that I'm surprised that it's grains instead of whole grains. If there's some education in the app that you want to be aiming for the whole grains. But then if there is whole grains instead of the grains, then you'd need like an other category or something.

Respondent 5: Yeah, I also want to comment on the grain category. So, sometimes, customers also have some starch like potatoes, sweet potatoes. I would count them as starch and for veggie, because on the food guide, you can also see some fruits, and maybe veggie/fruits.

Facilitator: So, would you see that as one category for vegetables and fruits, or two categories, one with vegetables and one with fruits?

Respondent 1: I feel like that’s a tricky question because if you're looking at Canada's Food Guide, it should be fruits and veggies to be consistent. But yeah, when I am talking with people with diabetes, this app might not be relevant, because we're being a little bit more particular and looking at like the starches versus the fruits.

Respondent 7: I was thinking the same. This would be just for the healthy general population, right?

Facilitator: Mm-hmm.

Respondent 7: I was also wondering, so can you adjust proportions for any of the categories, like the user can do that?

Facilitator: Yup. The user would be able to change it based on their actual diet and what it would look like.

Respondent 7: OK, OK.

Respondent 8: My thought was that with doing the adjustments, intuitively, I would want to adjust on the plate itself, like be able to drag the different sections of the pie chart versus going down to the icon below.

Facilitator: Like kind of a zooming in or dragging them?

Respondent 4: Like dragging it. Like if you wanted to make the grain section bigger, like drag it over to the right.

Facilitator: OK, I like that.

Respondent 8: I was just going to say that you also mentioned that you can adjust the size of the plate on the phone. I'm just not really sure what the function of that would be. You're not making it a size of an actual plate? So, I don't know if that's necessary.

Facilitator: Yeah, that'll come up in our question. So, don't get too ahead of ourselves. So, I’ll stop sharing now. And just the first question about the app would be how would you view the application working to record all meals and snacks throughout the day?

Respondent 7: I feel like it's missing information because depends on if the point is to track just how you measure up to the Food Guide, or if it's tracking all of your food, because we're not including any of the like stuff you would put in the other category like fats or sweets, beverages, things like that. So, you're kind of missing a big chunk of most people's diets with the current setup.

Facilitator: Definitely.

Respondent 4: I think also with a lot of the apps, but at the end of the day, you end up with some information about what percentage of your intake was carbohydrates or protein or how many grams in total. Whereas with this, I feel like I would be inclined to want to just log – I mean, of course, we want everyone to log it truthfully, but I think it'd be a little easier to just be like, “That's about half my plate is veggies.” So, in terms of snacks, it might be difficult to log. So, I think at the end of the day, I'm not sure what the information – I think there needs to be something at the end that shows like how close they were.

Respondent 7: And cumulative like evaluates your whole day and see how do you measure up to those proportions.

Respondent 8: I was also wondering, I've had clients who aren't quite sure what a protein food is or what is in what category. Sometimes, yeah, like spinach is a protein food. They'll identify that kind of thing, which is fine, but I'm wondering if there is like a log, or at least some sort of guide in the app to help them kind of categorize their foods.

Facilitator: Just to be clear, there's nothing in the app at the moment. So, we're asking you what you think would be good to be in the app. So, if that's something that you'd suggest? Yeah, definitely.

Respondent 8: Yeah, for sure.

Respondent 2: Yeah, that's a good point because with starches and grains, it’s often really difficult for patients or clients to know, is corn a vegetable? Is it a starch? Potatoes often will be classified as a vegetable. So, maybe having some kind of like – tapping the starch category or the grain category brings up all the options kind of an idea.

Respondent 8: Yeah, Or a quick search button or something.

Respondent 2: Yeah, yeah.

Facilitator: Yeah, I definitely agree with that. Something like as they're logging it, they're like, “Oh, where does this really fit? How would I categorize it?” would be great.

Respondent 7: And to add on to that, it would be really important to include culturally, again, different cultures food. So, it's not just a classic Western diet, especially since a large proportion of Canada is from different places in the world.

Facilitator: Yeah, definitely have cultural awareness built into the app. So, how would you think see things like snacks being recorded?

Respondent 1: That's more tricky, I think, because how do you differentiate between a meal versus a snack? And do we care about the portion sizes that you're eating, or just like the percentage proportion of your meal of the makeup?

Because you can eat a snack of a quarter cup of celery and if that's all you eat, like, it's 100% vegetables, but it's very different than a quarter cup of celery, like half your plate as part of a supper meal. And then, let's say you have celery and peanut butter. Well, how do you track that? Like, what percentage is protein?

Facilitator: Yeah, that's, again, going ahead to our next question. So, it's really if you were talking about getting that summary plate, it would be important to know what the approximate portions are. Which is why, Michelle, we had the different sizes of the plates, that could represent different portion sizes. So, how could you see that working for different meals and snacks and breakfasts and foods?

Respondent 1: I would see that working if there was some sort of thing on the screen of if you're adjusting your size. It's like, oh, this is like, I was going to say eight-inch plate or something versus a smaller plate. But then people might not know how big is my plate? I don't know. How big is my portion? I don’t know.

Respondent 2: Yeah, I could see that being when you first open the app, that's something that you'd have to input into the app and say, what are your metrics, so to speak? So, how big are the plates you use? How big are the bowls that you use?

And get people more aware of the their own – I forget the word, not cutlery, but plates and bowls that they use so you guys know, well, if it's an eight-inch plate, then we know technically, you should only be able to fit this many portions on a plate. Unless you're piling it on top, but then that defeats the purpose. Yeah, I don't want that kind of stayed within the realm of the question.

Facilitator: So, at the beginning, they would put in like they're kind of presets of plate sizes that they'd be using?

Respondent 2: Yeah. And then that would change kind of the proportion that you guys would be able to fit on the plate kind of thing.

Respondent 7: I like that idea too of just like maybe an extra layer of mindfulness too, like awareness of like, “Oh, I didn't know that my dinner plates like 12 inches versus a nine-inch plate.”

Respondent 8: Yeah. Just going back to the snacks like I almost feel like it might be better if it was logged a different way, like it wasn't a plate. You just added what types of foods you were having.

Respondent 1: Yeah, I second that. It would be hard. The proportions don't really make sense for snacks.

Respondent 2: Unless it was added to the whole day, I guess, like you have your meals. And then at the end of the day, it's tallies up like, OK, well, you add the snacks on top of the meals to get an idea of, well, you actually did make up more vegetables because you didn't eat as much at the meal, but then you ate some for snacks. It kind of makes up for it a little bit. That's another way to look at it, I guess.

Respondent 8: Like a daily summary, like your percent produce, your percent other foods like in a pie chart or something. And then you can see like, “Oh, actually, a lot of my foods don't fit into any of the categories.”

Respondent 1: Yeah, I like that idea.

Respondent 4: I don't know if any of you ever used e-tracker, which has now I think gone. But I think something like with the new food guide, there aren't portion sizes, necessarily. And it's not about that and like counting during the day, because I mean, the whole point was to simplify it. But I did like that with e-tracker at the end, it did tell you how many portions you were having and how close it was to what you should aim for. I just thought it was a nice summary without like having to actually think about like, OK, what is a food guide serving. And then finding out at the end of the day, I had this many servings of fruits and vegetables.

Respondent 1: I did like that.

Facilitator: Like comparing it to a reference standard, just to tell them what they should be aiming for.

Respondent 7: I personally liked the portion sizes and the servings per day. I think that with the new plate method, it is hard for people to know how many fruits and vegetables they really should eat. I find I get that as a question a lot and I try to focus on making half the plate. They're like, but how many should I have? So, I think a lot of people do like having that.

Facilitator: Yes, and that is our next question. How would you suggest that users track their meals portion sizes on the plate? So, what would like a quarter of a plate represent?

Respondent 2: Yeah. I've been thinking about that a little bit more recently, actually. And yeah, I think it's fairly easy to combine both. So, say a quarter of the plate is like two servings. So, say it's a full cup. So, if you want for grains, two half cups, then you know you have a quarter of the plate. So, combining a little bit of both and saying, well, if you know that a quarter of the plate is considered two servings. Then, yeah, I kind of lost my thought there. Yeah, kind of mentioning that in both.

Respondent 3: I think that's a tough question because I think it depends on what the user is looking for. And I don't know like if more people want the portions versus more people want the simplicity of just arranging your portion size, like it was on the app that you guys started.

Respondent: And I wonder like in to add to that, what if there was an option where you could select if you prefer portions, or if you prefer more of like the percentage of the plate or things where they get to kind of navigate like, this is how I like to track. Because some clients love being very particular and some are like, “Nope, that's not happening.”

Respondent 3: Yeah, I like that idea.

Respondent 7: Yeah, I like the idea of choosing your complexity. Like all the apps right now, it's like you're choosing an app based on the complexity you want. But I think if it had the ability to self-select what you really want to do, I think that'd be really helpful..

Facilitator: So, what would you see as the different options to select?

Respondent 7: Like just logging the plate if you just want to focus on that. Or if you want it to be able to input what you're actually like the portion sizes of what you're eating, so you could log it that way.

Respondent 3: Yeah, or percentages, if you wanted to see those numbers. Just different options, different levels of how detailed I guess people want to be.

Respondent 4: And or maybe keeping the picture option like taking a picture of your food.

Respondent 2: Yeah, that'd be super handy. Yeah.

Respondent 5: I think for a portion size of different food, I would still think that it will be depends on the food as well. Like, for milk, we’ll say in cups and for chicken breast, it’ll be in grams or pounds. I will say still, maybe using the ones that are commonly used by people that will be helpful.

Facilitator: Yeah, so the portion sizes maybe that were on the old Food Guide.

Respondent 5: It could be, yeah.

Respondent 2: Yeah, I think that's where my mind goes right away. Maybe I'm conditioned that way. But to think of the old food guide and the portion sizes. And then almost visualize if someone says, OK, well, I drink one cup of milk and then we know that's one serving and then that goes into the plate. And you can tell you that visual aspect of it going onto the plate and filling up, say that quarter, or whatever, however, this fits in the plate. And then they could kind of relate that to the plate and kind of mix both together.

Respondent 8: I think it'd be really difficult with the tracking of fluids like there's anything right now to differentiate if you're having like, does milk fit – I think we know where it fits. But will somebody know that milk fits under a protein food? But then how will that compare? Like if somebody is having like a sugary beverage, there isn't anywhere to log that right now.

Facilitator: Yeah, exactly. I'll keep that question for later but it's definitely a great point, because it's very hard to know, kind of what a cup of milk would look like on a physical plate.

Respondent 2: Yeah, that’s a good point.

Respondent 4: I find the portion piece challenging, kind of with the app, and with the new food guide in general, because part of it is well as dietitians, we like to be prescriptive, and a lot of our clients like that as well. “Like, tell me what to eat and tell me how much to eat.”

And with the new food guide, like they took out the portion sizes on purpose, and the focus is on mindful eating and knowing how much you need to eat in terms of like a portion size for a 6’6” man is going to be very different than a 5 foot nothing woman. So, how do we represent that like that you do need more food physically like to sustain your body. But how do we make it in the app that it doesn't look like you're eating too much or too little.

Facilitator: Yeah, so maybe that kind of individualization piece could be important.

Respondent 7: Well, actually, I think that's such a great point too. Because if we're thinking about the basis of the app is to help bring awareness and just to see like, do these foods even make an appearance on your plate, or as part of your snacks or what have you is if we're trying to build awareness of like, OK, we’ll aim to have a certain portion of vegetables here in there. Do they have an opportunity maybe to add some comments of their hunger and fullness or like that mindful this piece? So, it's a bit more unique for them to kind of look back on.

Facilitator: Yeah, for sure. So, moving on, on that what would be considered a successful day for people within the context of this application?

Respondent 7: I think maybe the proportions on the plate would be one thing.

Respondent 5: I think also being able to track the changes of how their plate proportion changes. Like yesterday, I have like a third of my plate as veggies but next day I have like half a plate as my veggies. I think being able to track the changes could be helpful in arranging.

Respondent 1: A weekly progress report or something too.

Respondent 8: I love that idea. Because then it's more of like their personal like, “Hey, I used to like not eat as many vegetables and now look at me go.”

Respondent 3: I think, yeah, a successful day would be defined as something different for different people too in a way, especially since you can kind of…yeah.

Respondent 7: In my mind, at the simplest level, f the Food Guide says, you should have half your plate veggies, a quarter grains, a quarter protein, like a successful day would mean that you were getting that balance.

Respondent 8: Yeah, that’s true.

Respondent 2: So, I guess, if I could ask the question, would it be more important like meal to meal or be like the whole day? Which I think I know the answer. But like, could you have like a report for this meal was good? You could see tips and tricks that we can be given.

Respondent 7: Yeah, that would be good. You get some feedback on your meals. Because overall, as dietitians, I think we all would say that the whole day and your pattern over a week and month is much more important than the specific content of each meal. If someone's having a salad for lunch, I don't want them to cut down on their veggies, so that they can have a piece of garlic bread with it, too, which would be delicious. But you're getting your quarter plate of grains, right.

Respondent 5: I think variety is another factor. If some of they have like chicken breast every day for lunch and dinner for like 30 days, I think adding more variety is also --- because for the new food guide, you also see like they're different whole grains, different veggie options. So, I don't know if there's a number like specific number of veggies for the week, or for the day that you want to add to your plate, because we don't have the guidance. Just a thought about variety.

Respondent 7: So, something I've been thinking about which kind of coincides with your last comment there is I would like to see some customization like prompts to aim for specific types of goals based on Canada's Food Guide. So, you would have that, but there'd be like a goal setting section like, what are you looking to improve?

So, someone might check on vegetables. And it's like, OK, do you want to try to aim for half a plate, like a third of a plate? It wouldn't have to prompt on the actual numbers, but you could kind of like set your target or it could be like variety.

So, I want to aim to have more types of produce. So, you could kind of have based on Canada's Food Guide, some like suggested things to work on. And then you can kind of click on some goals not to overwhelm people by doing like, “Oh, this is all that you're doing wrong.”

But, oh, I want to work on one or two things. And then you might have an alert in like a week saying like, “How did it go? Increasing the types of vegetables that you were trying to do.” I don't know.

Respondent 1: I love that.

Respondent 8: Yeah, that reminder to work on your goal is a really good idea.

Respondent 3: I think in terms of feeling successful, at the simplest form, I think it's really good. It would bring awareness, it would be super easy to log. There wouldn't be a lot of labour behind it and then you could just kind of look and it brings that awareness about your portions.

I think where I worry about like what a successful day looks like is if somebody is trying to log say a snack and it has no vegetables. And let's say they actually are having a lot of vegetables through the day, but now they have a meal that now sort of shows they have no vegetables. I'd worry they'd feel like they're not succeeding because of that.

Facilitator: So, coming back to that kind of summary piece of putting everything together and not looking at the individual ones as being good or bad, classifying foods that way.

Respondent 5: I'm thinking there should be some like matrix in terms of calories maybe because for the new food guide, we got rid of the servings. I’m thinking maybe like being able to set up a calorie goal would also be helpful. For somebody who is eating healthy, but maybe eating like 4,000 calories per day. So, like after doing this for a month, he or she might end up gaining weight, despite of eating healthy. Maybe have a caloric goal.

Facilitator: That would be really difficult with the format, kind of the plate format, because we wouldn't have them necessarily tracking their portions.

Respondent 8: Yeah, I disagree with the calorie piece. Because if somebody wants that information, they can use MyFitnessPal or one of the other hundreds of apps that are out there. And if we're not getting them to log the specific information about the food like, “I had a 6-oz. piece of chicken”, then you're not going to be able to calculate the calorie content of it.

And also, if it's just logging your food group, as far as whether I have chicken or a pork chop or tofu, it goes in the protein category, it won't know what variety of different foods I'm having. So, it can't really suggest that variety, because I'm just keeping things so basic with what I'm logging. You're just logging the protein, you're not really saying what type of protein you're eating.

Facilitator: Would you think it'd be useful to have that kind of sub classification?

Respondent 8: I like the idea of just tracking based on the food groups, because that's how the Food Guide is set up. But I liked the idea that someone had of like, when you click on the protein, it gives you the examples of foods that would fall in that category, or that you could search for it. And then when you click on chicken, it just logs it as protein. It's not going to log each food individually the same way that you would with MyFitnessPal or whatever.

Respondent 1: Yeah, I would also agree with that. It sounds like the point of this app would be to simplify things. And since there are so many out there that you can track things like calories that additional information with. Maybe the goal of this app would be to kind of make it even simpler than that, right? Just to keep it simple for everyday people who don't want to know all the numbers or be overwhelmed by all the information.

Facilitator: Yeah. And we may not have like dieticians necessarily following all these people on the app. So being able to do that assessment piece would be difficult. But you're right, Nia, for sure to have that if someone's eating 4,000 calories, but it looks like the Canada’s Food Guide that may not be meeting their goals, or what they need. But again, we don't really have the control over that or the power behind it to be able to tell them.

Respondent 8: So, one thing you can do if you're concerned about like the portion sizes and stuff is to kind of put that mindful eating piece on to like one of those little goals. We could prompt like ask me if I'm hungry before and after eating, and then like reading your hunger before and after.

That's not going to be for everyone but that could be some way to get around the fact that there's no calorie or is this too much good for you. But if you're always eating until you’re stuffed and you're eating when you're not hungry, that might be a good indication that it's not the right amount.

Facilitator: So, building on that which other elements of the Canada’s Food Guide or the backside of the guide would be useful to include into the application?

Respondent 2: Did you cook this food at home or did you purchase it? Where did it come from?

Facilitator: I can show it if you need a little refresher as well.

Respondent 7: Like anything that brings more mindful eating into it. I don't think it would necessarily be helpful to bring up the nutrition label part like, “Did you read the nutrition label on this?” Because I don't think that's what we're really trying to achieve with the app. But even just, “Did you eat this meal without distractions?” The whole hungry and hunger scale in.

Respondent 2: Yeah. It could just be like a yes, no kind in the box. Did you enjoy it? That’s a good question.

Respondent 7: Satisfaction factor, for sure.

Respondent 2: Absolutely, which will lead to long term adherence. I mean, if it's foods that they're not enjoying in the moment, I mean, they're not going to stick with it. So yeah, that's a good question.

Respondent 3: I feel like I wouldn't want it to ask if you ate the meal with others, because even though that's something that we want to promote, I think for a lot of people that might be something that you know, if you're ticking “no” a lot and you don't have a way to really change it, especially with the pandemic going on, I think it would them sad.

Respondent 8: Yeah, that's very true. It's not in their control at all the time.

Respondent 7: And in terms of what other elements of Canada's Food Guide should be included in the application, I'd like to see a link to see all of Canada's Food Guide so that there is awareness. And then people can click on it like, just like online where you can actually learn more of the different categories. Not necessarily having to set like, “Did you eat with others? Yes, no?” but like, you can learn a little bit more about that category.

Respondent 7: Yeah, that's true. Like cook more often or wanting people to eat at home, cook at home, but not everyone has that skill. So, if there's a link to more resources or something they can look into to help with that, if they are interested.

Respondent 8: Yeah, because I view this app as a learning opportunity as well, right? Because a lot of people don't really know what Canada's Food Guide is. Or they know the front side, because I talk a little bit more about that side. But then they wouldn't know as much about those other little categories.

Respondent 4: I love that idea of having like, an opportunity for linking to other tools or resources. They do have limited cooking skills, and maybe they want to learn a little bit more about what else can I do with veggies? Or what else can I do with like lentils or other sources of protein? That it kind of leads them to that, too.

Respondent 2: It allows the app to expand as well, right? I mean, at least, it’ll allow the app to continue growing those different recipes ideas, or just different tips and tricks? I mean, I agree with it too.

Respondent 8: Yeah. Or even linking to resources that we already have like Cookspiration or something. I don't know. Just thinking random recipe resources or just linking to resources that are already there. But it'd be good to have that connection through the app since this app is directly about Canada’s Food Guide in a way.

Facilitator: Yeah, definitely. Alright, so I’ll stop sharing this. Here we go. Are any other ideas that come up throughout? Don't worry about what question we're at either, just feel free to jump in. So, you had mentioned the other foods category. So, which foods are not on the plate that you would think would be useful for people to track?

Respondent 8: Snack food, sweets.

Respondent 1: Added fats, like I think not for everyone, that won't be a goal. But if somebody is adding a lot of fats, they wouldn't know where to log it.

Respondent 5: Condiments too like salad dressings, sauce.

Respondent 7: And beverages.

Respondent 8: I wonder, and this is back to I think the one of the first comments someone made, since we're not tracking whether it's a whole grain or not, are we differentiating between that for this app? I guess, just like a general question?

Facilitator: On the guide, it is really whole grains. So, for fine grains, could be a different category.

Respondent 8: Yeah, exactly like some way to differentiate those.

Facilitator: Or within like the starch category, we could have different options. And then coming back to those other foods, how would you suggest that those be tracked within the application?

Respondent 1: I think that's a hard question. And I think it's about like how big of an app you're trying to make this. If we're talking strictly about Canada's Food Guide, like you could simplify your life and just have the other category and not worry too much about the drinks or the snacks, right? I can't remember exactly what it says in the write up behind Canada’s Food Guide about the snack recommendations, like I know I have my own that I tell clients, but you might even skip that section. I don’t know. I see that as being very difficult.

Respondent 4: It’d be really difficult to measure success also, like if you have other category then what does a successful day look like?

Respondent 5: I feel like for other categories, there are certain things like fat oil, whether it’s chips, we don't need necessarily, like the essential other food categories versus like unnecessary added sugar or added calorie stuff.

Respondent 3: I think it doesn't necessarily need to be part of the tracking, as long as those other parts of the Food Guide are incorporated.

Respondent 7: At the same time, I see both sides with the other foods, and I don't know what my opinion really is on it. This isn't a weight loss app or anything like that. But let's say you eat the balanced Food Guide, but then you also eat a whole bunch of snack or other foods on top of it, people are going to get the impression that they're healthy when their overall diet may not be. And I love chips and cookies and all that stuff but you shouldn't have as many calories from your meals as you do from your snacks, like over the course of the day, right?

Respondent 3: I think that the other category should be included. Because I think that if it isn't included, like if it doesn't seem like your food can match what your app can let you do, people might give up on the app, because it's like, “Well, this isn't real relevant to – I didn’t have whole grains, I didn't have produce. I had some protein, and then some other kind of” – I can't think of other examples right now.

Respondent 2: Pizza pocket. Where would that go? Where would that fit into?

Respondent 7: Yeah, any mixed meals like pizza or soup or things like that.

Respondent 3: Yeah, I would keep the other category. And maybe when you're marking the success, the goal is Canada's Food Guide but it’s how closer are you this week compared to last week, instead of having a particular goal for like, the other category should be like 5% of your plate. Don't give them a goal about that, just focus on the goal.

Facilitator: So, should the other foods be a part of the plate, or a separate category?

Respondent 7: I think it's definitely like the other category has to be acknowledged. I'm almost kind of wondering, this is just a thought of like, if let’s say they're able to add pictures, it's still kind of acknowledging, OK, this is the food I ate today, whether or not they fit quite into these categories. But then it's still bringing the mindfulness of, OK, this is what I ate and this is like the vegetables, these are the full greens I had out of this. It’s not putting like an overemphasis on like, you had all these other foods.

Respondent 8: So, maybe you could do something, like there's the plate that represents the Food Guide. But then below it, there's either like a progress bar for other. If you eat a lot of other foods, like it goes the whole way across the screen. Or if you eat a little bit, like you'll only get this much showing up for other more. Or there's icons. Like for every serving of chips, you get one little icon of chips that pops up, and you can see, “Oh, I have 12 other foods, where yesterday I had two other foods logged.”

Respondent 2: Yeah, I like that idea actually. Getting like 100% mark and then you're on your way there. And then if you pass it, it'll continue and you get to see the progress with the days. Because I mean, it's normal to have some other foods in your diet like condiments and, and whatnot. But I think it's when it becomes a big part of it, that's when it becomes an issue. So, not acknowledging it is an issue but I don't think it could fit on the plate. I don't see fitting on the plate, but kind of what you said is having a bar underneath is a good idea.

Respondent 8: I'm just wondering because there's other foods that are condiments, which whatever are usually on the side. But then what about If someone has white bread or white rice, something that's not a whole grain, but it's still a part of their plate, right? They're just not choosing the whole grain category. You know what I mean? That'd be a little confusing, because it's like, well, this was my grain, but it's not a whole grain. So, I guess it doesn't count. It's other.

So, that entire plate would then look like, if it was protein or vegetable or just protein. I don't know, you know what I mean? I don't know what the solution is. But maybe there's like that other like category on a plate, different colour. So, it's not a whole grain, it's not like signified as that but it's still there. It's still food.

Respondent 1: And a spot for a comment. So, if you're clicking on other, maybe you could write down whatever there was if you want to. So, it's not like an obligatory comment but it's like, OK, yeah, like this was white rice, because I ran out of brown, whatever. Oh, this was a pizza pop.

Respondent 7: Rather than nit-picking on the quality of stuff, you know, whether you have white rice or brown rice, it still goes in the grain section of the plate. And then the whole grains is just one of those tips that you can link to in the app.

Respondent 6: I think that probably just add the most simplicity. I do agree with that too, where it's still like, you're still going to have that starch as part of your plate. I know for myself, I absolutely hate whole grain wraps and I'm still going to have the white wrap, but that's OK. But it doesn't mean I never having other whole grains as far as I need too.

Respondent 1: I agree that it makes it more simple. But I also feel like we should keep it the whole grains, because that's what Canada’s Food Guide is saying, right? I don't know.

Respondent 2: But then where would you put potatoes, corn, stuff like that is my question.

Respondent 1: So, in terms of Canada’s Food Guide, I would just say in the produce section like the fruits and vegetables. Like, once again, it wouldn't be relevant for every single client, right? Because there are people that are going to want to be more detailed about that. But I don't know.

Respondent 7: Sweet potatoes and corn, I would put in the grain/starch section, if we're going to call it that. Because otherwise, you would have like half a plate of potatoes, a quarter of plate rice and a quarter plate of protein. And that's a balanced meal.

Respondent 1: Yeah, because technically potatoes in the little picture on the Canada’s Food Guide are on the vegetable side. So, that's where things don't align. I mean, I talk to people with diabetes or all day, every time I'm like, “Don't look at this as part of the veg. This is part of the grains and starches.” So, I do that switch anyway. But I don't know. Yeah.

Facilitator: So, maybe making it like more of a starch category, being able to subdivide it into starchy vegetables, whole grains.

Respondent 4: I feel like it gets like too far from Canada's Food Guide then. I know, we all make that switch and tell people to look at those as starches because I think a lot of us are working with people with diabetes. Technically, if Canada's Food Guide says a potato is a vegetable, should we be telling people to make that switch? I feel like it gets a bit confusing for the general healthy population, like I'm not talking about for people with diabetes.

Respondent 1: And that's true, that's what Canada's Food Guide is for. It's the general population, not for people who have like end stage renal disease or diabetes, or other things that you would be more specific about starches.

Respondent 7: Yeah. That's more my critique of the food guide itself, it’s separate from the app and you're trying to base the app like Tory makes a good point. We can't really contradict what the published food guide says, but it doesn't really make sense in practice, as we all know.

Facilitator: So, definitely keep that in mind when we're developing it, where it's basically on the Canada’s Food Guide. But again, if we need to depart a little bit from it, then that's OK with us, too.

Respondent 8: Maybe a way to, again, back to the variety, if there could be some sort of prompt that's like the vegetables category. Is there a variety? I don't know something like that, something that prompts you to choose more than just one vegetable. I don't know if that even makes sense.

Facilitator: That makes sense, yeah.

Respondent 4: Sorry, I just had a quick thought too, because we were talking about sort of the how do you like track the whole grains versus maybe non-whole grain options to is having that prompt of like when you're saying that you had a certain portion of your starches, just having that prompt of a tip of like, “Oh, it's suggested to have more of your grains come from the whole grain variety.”

Facilitator: So, coming back to that educational piece that we could introduce into it. OK, so the last point about the app is about beverages. So, how do you think beverages could be tracked?

Respondent 4: It has to be separate.

Respondent 8: Yeah, 100%. Kind of like how the Canada’s Food Guide right now has water on the side, I could see it being like a little circle on or glass on the side for beverages. And then, I don't know, again, like another prompt, like make water your drink of choice, something like that, but they can also track that. How many cups of water? Or did I have pop? Or was it juice? With that prompt being primarily advocating for water as main drink of choice.

Respondent 7: Or what if there was like an icon, like a little picture of a glass and every beverage that you tracked, it filled up the glass, so you could see am I getting enough fluids over the day. And then possibly have one for your non-sugar sweetened fluids. So, where you got like water, milk, sparkling water, like fills up the healthy glass. And then if you're drinking juice, pop, something else like that, that goes into a separate cup. So, you can see how much sugar versus non sugar beverages you're having.

Facilitator: I like that idea.

Respondent 8: I like that as well.

Facilitator: And should these other beverages, so the sugar and sweetened ones, be counted as other foods?

Respondent 1: No.

Respondent 7: I don't mind that idea. I keep thinking of other foods, like in terms of tracking like the condiments and fats and stuff that we talked about, if that's not in the app, I don't think that's a big deal. With the other foods, I'm thinking more like the chips, pizza pops, cookies, and then those sugars in beverages would fit in that other category as well.

Respondent 3: See, I kind of like Shauna’s – like when you're saying like having something separate for fluids, I kind of like that idea. So, evaluating foods and beverages separately. So, you could do the tracking of the fluids, and if it's from a sugar sweetened drink or not. And then having like a separate report on fluids versus your food and proportions of the other foods. Where that would get difficult would be smoothies.

Respondent 6: And again, with milk counting towards protein, but then I feel like it'd be confusing. So, I'd be like my almond milk counts then as protein. So, there's no way to be perfect with all that. I think even just having it focused on promoting non-sugar sweetened beverages and tracking your fluid intake, I feel like that would be enough to bring the mindfulness in.

Facilitator: OK, so now the next part of the focus group, I'm just going to be mindful of the time that we have today, will be about the features of the application. So, which instructions and support should be provided to users to help support their use of the application?

Respondent 3: I like some of the things that we talked about already about like a weekly progress report or whatever, the linking to Canada's Food Guide and other resources. If you're looking to add more vegetable proteins, like how do you do that? Oh, here is a link to Cookspiration or whatever. I like goal setting, too, with some perhaps prompts of, oh, if you want to do goal setting on your types of protein, like here are some suggested healthier proteins, whatever. But yeah, what were you going to say.

Respondent 8: I was just going to say maybe upon downloading the app, there could be like a quick tutorial, just a general guide, like Canada's Food Guide guidelines, like a general overview just to kind of tutorial when you first download an app that you quickly click through. So, half a plate of vegetables, quarter plate of protein, whole grains. Just a quick thing, very simple, it's just like bare bones tutorial that you click through when you first download the app.

Respondent 7: Maybe like the tutorial having a little part on how you would be able to record mixed meal just so I can give people an idea.

Respondent 6: I think that's a great point, because that's probably where there'll be more confusion.

Respondent 2: Yeah, maybe some kind of frequently asked questions for some of the nitty gritty details that maybe down the road they'll get through, but initially, it's not super important. Like the almond milk and the milk and how we just figure it all out, having some kind of like, just answered questions.

Respondent 7: This is off topic of the instructions but the mixed meal thing made me think, again, is there a way or it'd be nice if the app could kind of pre-program mixed meals. Then if you have pizza, you can you click on a pizza and then it would ask you, “Did you have vegetables on your pizza? Was there meat on your pizza?”

And then it would kind of assign proportions based on what does a pizza normally look like. It's not going to be half a plate of vegetables on your pizza for the little pieces of green pepper or something. But if it could do the thinking for you, so that clients wouldn't have to figure that out, because that's one of the biggest barriers of a lot of these apps.

Respondent 8: I think that's a great idea because then it is just like, “Oh, yeah, I had pizza. They have it as one of the options here.” We'll see how that kind of falls and helps with that awareness piece. It’s not a huge barrier.

Respondent 3: You could also do another link for Dieticians of Canada, like find a dietitian to kind of promote good sources of help if you want more help.

Facilitator: Definitely. And then the next one is about which features do you think could help with adherence? So, helping people stick to using the application?

Respondent 1: Goal setting.

Respondent 8: The notifications with goal setting like that, it would give you those reminders.

Respondent 4: I was going to say notifications too. But I get easily annoyed with apps that give me too many notifications. But then if I don't get enough then I kind of forget about the app. So, like maybe like to set how many notifications you would like.

Respondent 5: Yeah, maybe the timing of the notification maybe like two hours before your meal. So, remember to log your things.

Respondent 4: Yeah, like do you want notifications at breakfast, lunch, supper? Do you want your notifications weekly with the goals or not?

Respondent 6: Or even just at your meal times like I don't know my phone knows when I leave work every day and my news pops up. So, I feel like if it just gave you a notification like when you're eating your meals you could even like set that in if you want that notification.

Respondent 7: Yeah, I think with MyFitnessPal, I can say I want to supper notification at 6pm and you can choose when you want those to happen.

Respondent 2: Definitely some kind of report at the end they're saying whether the day that week or both, or even every meal just summarizing how you did.

Respondent 7: And the ability to export that report like if you are meeting with a dietitian that you could send it.

Respondent 5: Maybe add some like positive reinforcements or encouragement sentences in app so that people continue to use it.

Respondent 2: This is kind of a joke but could they choose like maybe they like more like, “Come on, you suck today.” Just kidding, like the opposite of positive.

Respondent 5: I used Nike runners. Some whenever I finish a run there will be somebody like a coach or celebrity or just encourage people to continue running. For me, after one person log or finish one day of their journal then there'll be some encouragement recordings from a celebrity or something.

Facilitator: I’ll see how much budget we have. That’d be very cool.

Respondent 7: Yeah, just something like you know, “You tracked seven days this week” and you get a little congratulations pop up for inputting data every day.

And I think the other big thing as far as adherence is simplicity. We all know how much work it is to input every little thing. So, the less input and the less work that people have to do, probably the more likely they're to stick with it. So, we've talked about all these features and we could make it super complicated, but it doesn't really work in the long term, right?

Respondent 2: I think, also, you need some kind of engagement too. So, yeah, it's cool to have a goal, say, three months down the road, I want to be able to have my proportions to be as such. But then also like a weekly progression of OK, so you've done say a quarter of your plate is veggies now. You've got two variety of vegetables, try to next week now up it up to three variety and get a little bit more than a quarter and just continuing.

So, that they know that if I don't do it this week, then that's going to mess up next week's progression. And that's kind of where working out with weights is very beneficial to have knowing the progression of each workout, then next week, I won't have gotten the previous weeks training. So, you won't be ready for it. So, something along those lines to keep them like enticed to continue using it.

Respondent 3: I also like something a little – like if there could be something interesting to increase engagement. So, like, without being too annoying, because I don't like a lot about like reminders and stuff, but like maybe a monthly article or like “Oh, like here are some featured Instagram pages from dieticians” or some sort of thing that makes you click on the app because like, “Oh, this looks like an interesting story”. And then you're reminded that it exists still.

Respondent 7: Or that can even be like a weekly newsletter sent by email, because often you need to sign up for an account and give your email address when you download it an app.

Respondent 3: But I would say biweekly or monthly, but that's just me.

Respondent 6: No, I definitely second that, like less often. I like the idea of like even that newsletter, whether it's like highlighting. I love the idea of Instagram accounts or certain articles or recipes. But that, yeah, probably tops like once a month in my opinion there too, just so I know anytime I get it even on a weekly basis, I'm annoyed that I have to review everything.

Facilitator: So, find balance to find with being annoying and being useful. OK. Good to keep in mind for sure. So, the last question about the features is, which features would be required to ensure accessibility for all users?

Respondent 6: That it’s free.

Respondent 2: You can use it on all phones. Android, iPhone. I was going to say Blackberries, but they don't exist anymore.

Respondent 8: I was just going to say if it's possible to translate it in different languages.

Respondent 7: And have different like culturally appropriate foods. And also, foods that like people on a low budget might have.

Respondent 8: And back to the free thing, I don't know what the budget is for the app. But even pay what you can like have a little sliding scale perhaps. I don't know.

Respondent 7: I think the free is important. I can pay for apps, but I will specifically choose one that's free because I don't want to pay for apps.

Respondent 8: Yeah, I agree. I'm on the same page. But yeah, I don't know.

Respondent 2: Maybe having like if you want extra information and you're really curious about something, you could pay $1 to access that information. I mean, because it doesn't inhibit them from using the app. I don't know if that would be like not fair. But I mean, it's not necessary information to use the app but it’s just extra.

Respondent 7: Yeah, I think I'd be more inclined after six months of using the program of getting a prompt of like, “Oh, help support our thing. Pay what you can.” But like I said, I don't know the budget. If you can do it free, that would be best.

Respondent 8: Yeah, I definitely think if possible to make it as every aspect of it as free as possible would be best in terms of people and accessibility.

Respondent 2: I'm going to have a little quick pushback in terms of let's say, because it is part of investment, like to start and commit to something part of it is investing. And if you're going to invest $5 in this app at the very beginning, then your brain is going to automatically say, “Well, I've invested money into this. I'm not going to just put it on the wayside and not use it. I'm going to spend a bit more time learning about it and tracking”.

As opposed to if you just download it for free, and someone told you to do it, whether dietician or friend, then I would argue that have more likelihood of it not being used as much. And then it’s a loss in the end, even though more people have the app but not using it. So, I guess it's a debate in terms of investment, time and money and then accessibility.

Respondent 7: I've worked with like a lot of clients in poverty and that $5 would be a significant barrier like a $1. As soon as you have to pay something, you also need a credit card or your app store gift card or something like that. If we're trying to get information out there to people and this is the general population, like all the information at Canada’s Food Guide is free, I think everything in the app should be free.

Respondent 2: That’s true.

Respondent 6: I think that if you're paying people expect feedback. Right now, I think there's a new feature, it’s probably not that new. I just don't look at this out that much but Chronometer, they now give feedback, but you have to pay for a premium membership for that. I don't love the idea of paying for premium memberships. I think it takes away kind of from, I don't know just I don't like the divide. But I think that if people are paying for an app, they would expect feedback on what they're logging.

Respondent 7: Yeah, that’s true.

Facilitator: Anything else accessibility wise? So, things like audio version, fonts, modalities?

Respondent 3: Audio is going to be really hard because the proportions, I think they'll be really difficult, but it would obviously create more accessibility if it was possible.

Respondent 7: Yeah, something that works with the visually impaired features. Like on your phone, you can do the settings that it will read all the text to out loud, like if the app worked with things like that. The colour blindness piece.

Respondent 3: Even making sure the font isn't too small. I know a lot of people increase the font size on their phone. So, making sure that the app reflects that if you've done that.

Facilitator: Alright, so now I'm just going to open the floor to any other comments or ideas that have popped up while we've been talking that we haven't had the chance to address so far?

Respondent 4: I had one idea pop up and I don't know if this is helpful or not. So, I have a Google phone. So, things don't always work with mine, because it's not Android or Apple but some people don't have cell phones. So, I wonder if you could actually access this app, but on the internet like on a web page. That's my thoughts.

Respondent 6: I think that's a great idea. I've seen other apps do that. And I guess Cookspiration is an example of that too, where it's a website and the app.

Respondent 2: I have no more ideas. I mean, I'm curious to see where it goes and that's pretty much it for me.

[End of recorded material]
