# Supplementary material for: Perceptions of self-monitoring dietary intake according to a plate-based approach: A qualitative study
Source: PLoS One. 2023 Nov 28;18(11):e0294652. doi: 10.1371/journal.pone.0294652 (PMC10683993; doi:10.1371/journal.pone.0294652)
Supplement: S5 Appendix — (ZIP) [file pone.0294652.s005.zip › Anonymized RD Focus Groups/iCANPlate-RD-Focus-Group-4.docx]

**iCANPlate-RD-Focus-Group-4**

[Start of recorded material 00:00:01]

Interviewer: This is the RD focus group on July 30, at 4pm. So the first part of this focus group will focus mainly on the behavioural techniques and on self monitoring techniques that you use in practice or have used in your internships in any way. So the first question is, do you suggest following the plate method as illustrated by the Canada's Food Guide to your clients? I see some nods. Feel free to jump in, feel free to take yourself off of mute. So we have a little bit of a conversation now.

Participant 1: Yeah, I love the new Canada Food Guide, compared to the old one. Because this new one is more practical, and it's less intimidating. Because the old one had a lot of numbers, even one quarter cup of something and three quarters of that. Which was probably not the best or not the best or not even not even appropriate for my clients. My clients are First Nation Indigenous clients who they - many of them might not have a solid education, in health or even in math. So yeah, I love the new Canada Food Guide. I talk about it all the time, almost in every session that I do with clients for sure.

Participant 2: Can I ask you a question? I'm wondering if you got any backlash about the drinking water that is in Detroit, with a lot of assumptions about your community. But you know, a lot of remote communities in Canada don't have potable drinking water from the tap. So I was wondering.

Participant 1: Yeah, so in my communities, I work with a tribal council. So it has five indigenous communities. I will say most of the time, water safety isn't an issue. So water is still recommended as the best drink. I know all the reserves, I think soft drinks, pop and juices are way more available compared to like, compared to your other healthy drinks. But still, water is still number one of the drink that I would recommend to my clients for sure.

Participant 3: I often before patients are discharged if they're having trouble with balanced meals, or what meal should look I'll do some education before referring to an outpatient. And I find the hospitals not the best place for education, you forget a lot. But the plate method is a quick and dirty, easy thing to go over. And I think it's an easy visual tool until patients are able to better go over their diets with an outpatient dietitian. And they usually find it more comforting than what they thought I would tell them to do.

Participant 4: In diabetes as well, we use kind of a modified plate method. So very similar. The proportions are same on the plate. Usually I use that to promote. And then also we kind of tailor it a little bit though. So rather than having both fruits and vegetables, usually it's kind of veggie focus first. And then the types of the foods within it are also more the whole grains and the nitty picky pieces inside the guide. But yes, I find it so much more useful, especially if they have allergies, or they don't have milk or dairy, way compared to the old Food Guide. It's a lot easier for people to remember which is really good.

Participant 2: My clients are still, well they're all still in patient. And a lot of them haven’t been sort of un-institutionalised for a long time. So a lot of them have a hard time sort of looking at the plate and thinking how do I translate a plate into that [unintelligible 00:04:16]. So I still need to do a lot of work around that. But overall, it's so much easier than I've ever worked with the old food guide. That I can imagine what that would have been like. I think just the visual of it, I think makes food look very appealing. So yeah, I really like it.

Participant 6: I think for me, I use it for a lot of my patients, but I don't use it for eating disorder as well as paediatric population. Just because it doesn't make sense in that context. So those are the two types of population that I probably won't use the plate method with.

Interviewer: And what are any difficulties that you've noticed with using the plate method?

Participant 6: Yeah, so when the food guide first came out, it causes a lot of anxiety in the eating disorder population, because we do push a lot with to four food groups. And it's very hard for them to get enough for weight restoration. And yeah, for their goals, for their specific goals in terms of that. And there was a lot of pushback and especially for dairy products. So that was one challenge. And then with peads, well, I think two and up is what we aim for that would be suitable. But because they're eating more often, it might not always look a balanced plate, depending on what their feeding challenges are. And sometimes that can be - I find, the tool can be discouraging for family. If they don't look, it can be overwhelming as well, if their child only eats one or two things, yeah.

Participant 5: I would say there's also the fact that people try to reproduce what's in the plate exactly. So they're like, oh, but I'm eating excess food, but there's, it's not in the plate. So can I eat it or not? So it makes it difficult for them to know if you can include the food or not into their diet or not. I would say also, some people are asking for portions because the older food guide had portions. So they're asking, “Well, if I just eat a quarter of the plate, will it be enough for me? Will be too much or not”? So that are some of the challenges that I got when I used to have my internship.

Participant 3: To go off of what Participant 5 said, I think some people also look at it and think those are so many foods. How am I supposed to eat all of those foods? And it takes some education. I'm like you don't have to have bread and pasta and rice and all on one plate. It's just to show examples. And I think the other thing is sometimes yeah, when you have a plate it's easy to look at but what if it's a sandwich or a burger or a casserole where it's all grouped together. I think that gets a little confusing for people.

Participant 4: A lot a lot of the ones where they make homemade food and they have soups or they have their family recipes, always there’s the questions,” Was it OK? Is this the right amount”? For sure.

Participant 1: Yeah, for me I think the biggest challenge is that a lot of my clients don't have access to fresh foods. And so the frozen - even though they have to travel one and a half hours, once every two weeks to go to a grocery stores and go to grocery stores and get frozen vegetables and frozen foods. So for them I think saying that oh always info fresh produce, fresh vegetables and foods are only, are as not a protocol. So I think I yeah, there is good and bad of all this food guide, for sure.

Participant 2: Kind of with my guides, I think maybe they're a little similar to your client ones. And I think I'm always - a lot of them will go grocery shopping at Dollarama. And so I'm always talking about some vegetables where this is fine, frozen fruit and veg. But then when it comes to making a snack or something you'd have to put work into making frozen fruit and vegetables into a snack as opposed to some instead like carrots and celery sticks. So yeah, if I find myself repeating the same ideas over and over, and kind of running out of ways to make frozen vegetables frozen acceptable or frozen some, easy for people, like real [unintelligible 00:09:48]

Participant 1: Yeah, for sure. And in addition to the Food Guide, I always give them recipes because, yeah, they are like, “Oh, I know it's an ideal world. Everybody is eating, will be eating like that. Half their plate to be fresh vegetables and foods and whole grains and good proteins”. But they just don't know how to cook. Because for a lot of my clients, what's most available and most common to them are just chips and pop. And yeah at dinner times, it's this crap dinner and that's it. Or instant noodles, that's staples. So yeah, there are definitely some challenges to that too.

Participant 2: [unintelligible 00:10:32]. Health Canada food area at that time, but he was trying to do a press release to it. I can't remember all the examples, but half of the fruits and vegetables on that plate are frozen. So I thought that was really neat. But that doesn't come across, right on that plate. It doesn't come across. But they did put that effort into it but you can't tell.

Participant 4: Especially if you print it black and white right.

Interviewer: Yeah. I mean, it's knowing that they're there as options, but it is hard to access them for sure. And realistically, most people's diets do not look that at the moment. It's the general goal for the general public. And as Participant 1 mentioned, it's not necessarily applicable to all people in all cases and clinical cases, for sure. Alright, so I'm going to move on to the next question. But those are some great ideas that I'd you to keep in mind when you're talking about conceptualising the application. The second question is which diet tracking tools methods have you used with your clients in the past or currently?

Participant 1: I use some apps. So Recovery Record. My Fitness Pal sometimes. Practice Better will be another platform or just plain old paper and pencil. Those are - oh, and Cara app for GI issue clients. Those are the main ones that I use.

Participant 5: I also use My Fitness Pay Pal. No not pay pal sorry. My Fitness Pal. That one, people really like it. I think you can count calories. You can enter food that you're eating. So you can know where you're at during the day and what you can take or not. So I think that one was really useful for some people. And so yeah, that was the one that I think I use the most.

Participant 3: I don't really recommend any apps per se. But if I have a patient going home who has IBS or IBD, or colitis or Crohn's or whatever, and they're going to see an outpatient dietitian, I usually get them to keep a food diary, plus a symptom diary embedded. And then - so the symptoms, the stress level, and then what foods they eat, to help outpatient dietitian. But when they're in the hospital, I don't really - I know what they eat, so they don't have to keep track.

Participant 5: Yeah, I don't really recommend this either. It's just some clients who want to use them. So if they want to, well, I'll give them and support them with that. But otherwise, it's not something I will recommend either.

Participant 6: And in diabetes we focus a lot on carbohydrates. So if they're able to count their carbohydrate, it helps when we're doing insulin doses. So oftentimes, either it'll be a three day paper record, or a lot of them will bring My Fitness Pal or another calorie based tracker. Usually I think it's called like, calorie counter or something that as well. So those are kind of the main ones. In addition to a normal 24 hour recall, you just ask them what they eat.

Interviewer: Any other tools that you use to help them track their diet. Nope. OK, so I'll go on to the next question. It's, do you know any diet tracking tools, paper application or otherwise that currently resemble the Canada's food guide or the plate method? No, no.

Participant 6: Well, I worked a little bit before this role as a health coach for another program. It's the Canadian diabetes prevention program. So it's more of a branch in study. And so one of the products or the platform that they use is from a company in the states that does health coaching. And they're kind of patented or signature meal plan includes a plate, kind of Canada's Food Guide. The portions are a little bit different. But mainly from them. That was the only other place I've seen it.

Interviewer: So presented as a recommendation, not necessarily a tracking tool?

Participant 6: It's they give it as a recommendation. And then their tracker is like, based on a chart, based on the groups that they had, kind of thing. So nothing Canadian.

Interviewer: Anyone else know of any tools that is based on the plate method? No. OK. Great. Oh, we always have to ask that questions to make sure we're on the right track.

Showing the app’s prototype

Facilitator: You have any comments just based on this short little video?

Participant 2: What's going to require the user to know which category their food belongs in? That's our stumbling block.

Interviewer: For sure.

Participant 1: Yeah, for example potatoes and corn. They sometimes they forget. Yeah, because we say starchy vegetables, or yeah, it's kind of confusing sometimes, yeah.

Interviewer: For sure.

Participant 5: I may have a comment, is there a reason why veggies are not taking the half of the plate? In the -

Interviewer: This is just one recording. So this would be someone's plate would look like at their meal. So we don't want them to be perfect at all time.

Participant 3: It might make the font bigger too, I can imagine on the phone, I would not be able to see it very well.

Interviewer: Yes, definitely.

Participant 3: And also, if they're colour blind or anything like that, is there any thoughts about maybe patterning it or something?

Interviewer: Yes. That's a good idea.

Participant 6: I think this would be easy to apply for a plate again. But if it's a mixed meal, it might be harder to use.

Interviewer: For sure.

Participant 1: And what size is the plate that they're using?

Interviewer: Yep, we're going to get into that for sure. All right, so I'll stop sharing now. So the first question would be how would you view the application working to record all the different meals throughout the day? So if we think of breakfast, lunch, dinner, and then snacks, how would you do this working?

Participant 2: Isolated all into one big plate. Because you’re overall daily or weekly pattern is more important than any individual meal.

Interviewer: OK.

Participant 5: Like maybe you could add a percentage of where you are during the day. So you know, at the end of the day, what you're missing or not? Or I don't.

Participant 6: I feel I'd have to do it when I'm eating the meal, because I feel I would - by the end of the day, would I remember what proportions I had for breakfast?

Participant 2: But it would be like – I would combined it all into –

Interviewer: I see, in some, in a final. That makes sense.

Participant 4: Yeah, so I guess building on that. Have an entry for each meal that you have and then at the end have a summary of how it splits up. But I'd also want it to not be structured because some of my clients don't follow the proper meals or meal timings or their shift workers. So it changes day to day. So maybe could be time based or just meal one meal to meal three today.

Participant 3: Yeah and then how do we capture other foods like snacks? As well as I'm just imagining breakfast would be - if you're having cereal or oatmeal and a piece of fruit, I wouldn't know what proportion necessarily that would be.

Interviewer: And then something you said before was what size of plate. So what do you think could be different options for those, for that?

Participant 3: I think that goes back to the comment about if it's not on a plate it's very hard to visualise. So I wonder if there's other options to help people translate that into a plate.

Participant 4: And I guess it goes back to, whether you want it to be specific or not. If you want people to have eight inch, 12 inch plate, you could put different settings for the different sizes. But if not, then it's kind of just small plate, medium plate, big plate kind of thing.

Interviewer: Yeah, so the goal of the app is not to go back into those portion sizes and being super specific, trying to make it as simple as possible. But kind of keep that in the back of your head, as going forward. And please feel free to stay off mute, if you'd like. You can just jump in unless there's some crazy background noise, it should be fine.

Participant 2: You guys hear the background noise here?

Interviewer: Nope. So I was hearing, entering individual meals and snacks, and then getting a summary at the end of the day that we could look back on. Do you have any other thoughts about how those meals or snacks could be represented on the plate and on the app?

Participant 2: Well maybe we could also do like, instead of a plate, you could make a bowl and then people if they're having a soup, they could have the option to sort of try to visualise how much of that bowl with vegetables. And maybe I guess, is it [unintelligible 00:21:29], glass plate.

Participant 4: Because I think I think the toggle, there's almost too much ability to change the plate size. I feel if we're not - because it'll be really subjective per person. I don't know if I'll remember how big I made the plate yesterday versus today, even if it's the same plate. But maybe that could be part of it, too. Maybe there's a preset snack plate versus a lunch plate or something.

Interviewer: Maybe that that glass?

Participant 4: Yeah.

Interviewer: Anything like that?

Participant 2: Yeah if we’re not focusing on portion sizes, why doesn't matter what size plate we’re using? [Unintelligible 00:22:24], someone working in diabetes, I can see why that would actually be really important for you to know. [Cross talking 00:22:32].

Participant 3: I guess it's for the general public, though. Which would make it –?

Participant 2: Public yeah.

Interviewer: Any other ideas and how it could be tracked?

Participant 6: I guess in an, on a app interface, I could see it maybe it's almost it records what the picture that you made looks and then you could for a day, you could just scroll or have it in one shot. Rather than combining it. That could be another way that you could look back. Maybe there's a zoom out of a plate that you made, kind of thing.

Interviewer: Kind of going back to that big picture. The goals. Alright, and then the next question would be, again, kind of relating to what you were saying, and how would you suggest that users of the app, record their meals portion sizes on the plates?

Participant 3: I think for simplicity, that idea of having a snack plate, a meal plate or bowl or whatever might be good instead of changing the size and then forgetting Jen says what you said yesterday you ate when it's the same size plate.

Participant 6: I wonder about the ability to maybe take a photo of what they're eating too. Just from experience when my clients do take a picture in recovery record, it's tells a lot more than just entering the portion size or the food. And if you take a picture and then be able to, use your picture to adjust the proportion or one out later or once you have a better look at it. I don't know if that would help.

Interviewer: Yeah, that's a good idea. And I have a picture as a as a compliment to the plate so how big would a snack plate be versus a meal plate?

Participant 6: Oh, gosh. I mean, I guess what the size of plate that used for tea. I don't know what you guys typically use. I use that. And then I think I think its six inch for lunch supposed to be and then eight inch for dinner. But it's so variable. It depends if it matters or not.

Participant 4: I guess even visually half the size of a meal plate. Like, it doesn't really matter.

Participant 2: I'm confused as to why we’re talking about plate sizes, and we’re not talking about portions sizes?

Interviewer: We did a preliminary study on paper with older adults. And they were really sticking on how do I know what my two eggs looks on my plate? How do I know how much to fill? How do I know how to represent my 17 almonds on my big plate? If I wanted to. So that's why we're kind of going on this because for users, it really does, did help to have that portion size and portion guide, which is why we're asking this question. But yes, theoretically, if someone's proportions are looking more the Canadian Food Guide, it doesn't really matter what portion they're having, they will still improve their diets.

Participant 1: Yeah, that's why I don't really talk about portion sizes, how big of a plate that my clients should be using. Because I use the mindful eating or intuitive eating kind of concept to guide them. So yeah, I don't, I guess I'm a bit too lay back compared to a lot of the dieticians. Might say, well just follow your stomach, whenever you feel hungry, start eating when you feel I'm comfortably satisfied and stop eating. So yeah, I don't really recommend a plate size or something like that.

Interviewer: Yep, very fair. And definitely what we want to aim for everyone with their bodies, for sure. This will be a tool to help people follow the guide, they probably will not have dieticians you to guide them in the ways that we would want to. There are other options of other things to put in the app. And we'll get into that as well. So keep those thoughts in mind for sure. Now, it's just about kind of the nitty gritty of what the plate could look like. So the next question would be what would be considered a successful day or meal? You mentioned that the whole eating pattern would be the most important, but what would be a success for clients or for users?

Participant 4: I can go first, honestly, because we have so many food logs, I think adherence. So if they fill out for a whole day, and they do the day, that's already a win for me. Yeah, if they just able to record it, and they're able to look back, that's the end goal. If they're going to do it.

Participant 3: I think too, I mean, obviously, if it looks the proportions are Canada's Food Guide, but I think even being able to, for people to better understand what food is a protein, like, what does that mean? What food is the grain to even have that knowledge? So when they're eating, even if it doesn't match, at least they know, it was their decision, and they made a conscious decision.

Interviewer: Making it a little bit more educational rather than tracking necessarily.

Participant 3: Yeah.

Participant 6: For some of my clients, who to graze a lot. One of the strategies we talked about is putting everything on a plate. So I mean, I can see this being helpful, maybe just to group their meals and snacks together instead of eating one of her little pieces at a time.

Interviewer: Alright. And then there are many other foods that aren't shown on the Canadian Food Guide. So which foods can you think of that will be shown on the Canadian Food Guide that you'd want us to track?

Participant 1: Because I work with indigenous populations. So some, having some kind of traditional indigenous foods might be helpful. For Alberta, I guess its moose. Moose meat and [unintelligible 00:30:11] might be helpful. And for people in BC I'm guessing Salmon. I don't know. It's more seafood, I think. Yeah, well, those traditional foods.

Interviewer: So having examples with more traditional foods that could fit on the plate? Sorry, you're, you're on mute. I can't carry you're saying.

Participant 1: Yeah, that's true. You're right.

Participant 3: And I guess that could have, sorry, applied to like, any cultural group really having different examples for different cultures?

Interviewer: Can you think of any specific examples for that?

Participant 3: I guess like, for East Indians, if they're eating a roti, like, what does that look like? If they're having dal, which is kind of a carb and kind of protein, what does that look like? Yeah.

Participant 1: Yeah, that's something that some of the dietitians working with indigenous communities here in Alberta are talking about. Because we were thinking of having an indigenous food guide. Yeah. And then I agree with you Participant 3. So probably having a separate food guide for some of the major ethnic groups might be helpful, too. But that's a different story. Sorry.

Interviewer: But it could be integrated as well, into the application making sure we have that cultural awareness and examples, for sure.

Participant 1: I think the mix – sorry go –

Participant 3: No go ahead Participant 1.

Participant 1: I was just going to say like, the mixed dishes will be really hard to represent on the on the plate. And as well as like, we talked about people who don't eat on a plate family meals. You know, in Chinese culture, we have a race and we eat family meals, I have no idea how much that will be representing on a plate. So that could be a challenge.

Interviewer: For sure.

Participant 1: Yeah, a hamburger soup. Oh, how does that apply to the Canadian Food Guide? Yeah it's a challenge for sure.

Participant 2: I don't think it does.

Interviewer: Which is something we have to think about, because not all foods fit on the guide. But we wouldn't want to kind of just let them go and not show them on the app or make them seem they don't matter or aren't a part of our diets. So how would you suggest that these types of foods be recorded or represented?

Participant 2: Whatever happened to the other group? That was [unintelligible 00:33:02], other group?

Participant 6: It’s gone. That's a good question. Do you put that in the ratio to? Is it just an other section? And then we put that in?

Interviewer: That's what we're asking you guys. Would you see it as being a part of the plate or maybe on the side?

Participant 1: When I am seeing this other foods, I always thinking because on the old Canada Food Guide, were talking about cakes, chocolate and all those desserts snacks. I don't know if we're talking about the same things or not. Yeah,

Interviewer: Yep, there are a lot of other foods that aren't on the plate at the moment. So they're things like dessert, so chocolate cake, cookies, muffins, ice cream, pudding, all those things. We also have fat sources that aren't on the guide anymore. So oils, butter dressings, sauces, things like that. We have things like condiments. So like ketchup, relish, mayo. All those things aren't on the guide at the moment. Does have things like seasoning, so salt, pepper, any spices, added sweeteners. Oh my god. So things like added sugar, sugar in your coffee, honey, maple syrup, anything like that. So have things like salty snacks, so French fries, chips, pretzels. Those don't necessarily fit perfectly on the guide. But they do have a huge role to play in Canadians diet at the moment.

Participant 1: I think it would make more sense to be not on the plate and have a separate section for those because yeah, it's -

Participant 5: I agree we need this. It could give people the idea that OK to eat that often. And that's a great part of diet, but it's not. So having it in the plate would not help them to make good decisions. So I would put it in a separate section as well.

Participant 2: I think Participant 5, I disagree with that. And moving just because o0fthe guides that I work with, are super concrete. But I think if I had like the Food Guide plate, because it seems perfect on the food guide plate but then we would still have this other plate, which is probably that. Whereas if we were to put it all on one plate, or maybe the food guide food, they're in my [unintelligible 00:35:39] other foods or maybe kind of dull, gross or something. And then your goal is to make that web smaller and smaller as you go along. I think that's how I could see that working for my guys.

Participant 6: I agree. Because I think behaviour change is what you're looking for. Part of that behaviour change might be seeing how many other kinds of foods that you're having on your plate as well. Or if they're all the plates are mainly other foods, right? Maybe that's part of the learning? I guess it depends how, it's always depends, doesn't it? I guess if there was a way maybe they could specify. Because I can imagine it being difficult if they had a sandwich and they had mustard to put it on. So maybe if they wanted to put what kind of other food that might help them as well to say or figure out OK, it was other but this is why kind of like. Because it depends, right? Whether or not it's important to change it, depending on what the food was.

Interviewer: We have some other foods that we count and some others that we don't.

Participant 4: I think it's important for people to track what they're eating. And that, obviously, we want them to follow a healthy overall lifestyle. But then my worry is it could very easily turn into patterns of disordered eating of I'm eating too many other foods. Part of Canada's Food Guide is cook together. What if friends came over and you bake this really nice dessert? And yes, it's an other food but you shared experience. There were all these other things? It's a tricky balance, I think.

Participant 2: [unintelligible 00:37:34], I 100% agree. Even though I am now contradicting myself.

Interviewer: That's fine. That's the goal. Don't worry about it. I agree. And that's the kind of the line we don't want to necessarily cross because we don't want people to be counting every single one of their portions and being there with their measuring cups. Like I'm only allowed half a cup of rice, something like that. But we still want it to be a representation. If we are going for behaviour change. We want there to be a movement towards that dietary pattern that could look the plate.

Participant 4: But I think it'd be omitted, they're going to be frustrated. They’re going to be I had this and it doesn't match, where am I supposed to put it? If it's for general population.

Participant 1: But these foods are already omitted. And they all forget, it would say limit or avoid. So yeah, I don't know how to best represent them.

Interviewer: Yeah, because the food guide is kind of the goal, what we want to aim for. But unfortunately, most people's diets don't look that. But as Jen mentioned, it is a part of people's diet. So we can't just remove them completely. If we want to be truthful about what the diet actually looks like. Our two ideas, will it have plates with the other foods or have other foods be a section on the plate. And then the second one was just have it as a separate category completely, and not a part of the plate. Any other thoughts on that? If you have any thoughts at any point about previous questions, please feel free to kind of go back. There's no set order really on this. And the next question will be about beverages. So how do you suggest that beverages be tracked within the application?

Participant 1: Beverages are the easiest ones to be tracked. Because this is one can or one bottle of pop or juice or something like that. Yeah, I don't know.

Participant 3: I guess, do we want to distinguish? Does it have to be water or not water? Because if that's part of the guide –

Interview: It is.

Participant 1: And things milk. That would - would that be a protein or would that be separate?

Interviewer: That's our next question we're getting ahead of ourselves. But yeah, so would there - would you see there being different classification for beverages? So things like sweets, sugar, sweetened beverages, fruit juices, coffee, tea, what other classifications could you see for beverages?

Participant 2: Caloric versus non-caloric.

Participant 4: I guess energy drinks are a popular thing for some people. I guess alcohol to, I don't know if you'd want to keep track of that or not. But yeah.

Participant 3: Yeah, I think those are all the main ones. Milk, tea, coffee, juice, sugar sweetened. And then smoothies and stuff, I guess depends on OK counted as a meal.

Interviewer: Again, comes into the tricky category of the mixed foods and mix dishes. So things smoothies could be broken down into fruits, veggies. And other foods, if there's sweetener in there or something. If there's protein powder, the backbone, the protein section. All of these little intricacies that were thinking about and why we're talking to dietitians about it. So then the next question, go back to what Participant 1 was saying was, how do you suggest that dairy and specifically liquid milk products be tracked on the day on the app?

Participant 1: I will still track it as protein, but it wouldn't fit on a plate.

Participant 4: Yeah, I think it needs to be - for them to be easy for them, it needs to be a cup. And they will be able to add a cup.

Participant 5: But at the same time, if people drink chocolate milk, it's naturally protein source because it contains a lot of sugar. So should be considered has sugary drink?

Interviewer: Good point.

Participant 2: And then soy milk has protean. But all the other ones don't. As far as I know.

Participant 5: Yeah, so almond milk doesn't contain a lot of protein either.

Participant 4: Yeah, almond milk, the oat milk?

Participant 2: They're coming out with new ones every five minutes.

Participant 6: Yeah, my client just show me this almond milk that has six grams of protein per cup.

Interviewer: Oh, interesting. I'll have to take a look at it.

Participant 6: Yeah almond and cashew I think.

Interviewer: And something else, I'm sure to add that protein. Yeah, so really, interesting considerations for different foods. And maybe if from what I'm hearing showing a glass of milk on a plate wouldn’t necessarily be realistic. So Jen, and mentioned having a glass as a protein source, are there any other ways that you could think of how this could be represented?

Participant 4: This isn't really how it will be represented when you're recording it. But I think if the plan was to record what you eat in a day, and then have a total end plate, I think the glasses could end up on the plate to simplify it to show overall protein.

Participant 6: Yeah, just a calculation that the app will do. And I think same idea going back to the other foods if we can just have an easy way to track them and then that can represent an overall plate. That could be an idea to have other foods and I'm aiming to have that portion reduced.

Interviewer: So with a total going back to that idea of grouping everything together, even if it's on different types of plates and vessels and cups and things like that. All right. Any other thoughts on the plate and how to record meals before I go on to the next section? OK, so which other elements of the Canada's Food Guide should be included in the application? We have that whole backside of this page, I can share it again, if you guides would like. Which other elements should be on the application?

Participant 1: Mindfulness? Yeah, the healthy tips.

Participant 2: I don't want to [unintelligible 00:45:29] app, more about the back page.

Interviewer: So that's what you would see it as kind of more of a tracker with these elements on it?

Participant 2: I almost yeah. It’s so hard. I guess it could be one of these? I don't know. But I mean, it is so important. How and where and why we're eating as opposed to what, necessarily? So it almost seems like, yeah, the back make us be more important entirely. [Unintelligible 00:46:14] at the bottom. So yeah, I don't know. [Unintelligible 00:46:23].

Participant 4: I guess you could have with every meal or snack, a tikki box of, did you read a food label when you cook this? Or did you cook this meal? Or were you eating at a table or something and then comments, so they can track like, I was looking at my phone. Or I - but then that might get, like, you just check everything after a while and aren't really, truly filling it out?

Participant 2: Sorry, there's a code, I got to go.

Interviewer: No worries.

Participant 6: Can it be a reminder, maybe every day, kind of these are the things to aim for? Because I feel like it might not be realistic for every meal. But more so for a whole day.

Interviewer: Yeah. Maybe some better at every meal. And some of them that are more a reflection on your whole day. Going off with Participant 2 had said she said that how when, where, was maybe more important than the what? So what could be recorded in the how?

Participant 1: So I'm guessing how did you eat that meal? How did you eat that snack? In front of, while you're watching TV, or while you're at the dinner table, enjoying the food with your with your friends and family? Or while when you're driving? And all those, yeah, like, how did you eat your food?

Interviewer: The whole setting where it was coming from? Any other thought?

Participant 5: It could be also how did you cook it because you can grill it. Put in the oil so it could be healthy and not healthy. So that could be another category to add.

Interviewer: OK, so I think we have the – go ahead.

Participant 1: Oh, I just have a one comment to say. Well, I was thinking when you're designing that app I - most often I feel it's nice to have some fancy pictures and fancy recipes. But I found what I hear from my clients, the most likely they know - oh, so in an ideal world, this is the best way to eat. But then they separate themselves from those fancy pictures, fancy recipes and all those healthy tips. They know they were like, oh yeah, this is the ideally we would. But then and then give me some practical, practical easy recipes. So yeah, I don't know what I'm talking about. Just have something fancy but not too fancy, yeah.

Interviewer: So make it a little bit more simple instead of providing some crazy difficult recipes to follow.

Participant 1: Yeah, in my practice, I never talked about a Mediterranean diet. No, that's out of the picture. That's too much for them. So, yeah.

Interviewer: Somewhat like the basics of cooking and prepping elements from different groups potentially putting them together.

Participant 1: And you don't have to have all these cooking utensils. And a blender from KitchenAid. I don't know, these simple tools, yeah. Simple tools and ingredients.

Participant 4: I guess for the food, reading food labels, one, if you were to incorporate it, would you have links to Health Canada or anything teaching how to do that?

Interviewer: Yeah, that could be something that we could include for sure.

Participant 6: Yeah, I almost feel I don't want to reinvent the wheel for the other parts. Because I feel there's so many really strong really good resources, for some of the other pieces. Especially knowing about, like, mindful eating, or eating together with family, those kinds of things. So maybe it might just be about like, providing resources to other really good sources. Because I know that there are places that, spend hours trying to make their little rewards to write that already really good.

Interviewer: Definitely so like referring out in the [cross talking 00:51:35] past. Yeah, for sure.

Participant 1: I think also, if you're going to touch on like, did you eat with others, that might be a good, like, once a week, end of the week kind of reflection. Because if you live alone, and you work from home, you're probably not going to eat all your meals of other people. But then you'll have to make an effort of like, OK, let's have dinner with friends or family or whatever, at least once a week instead of every day.

Interviewer: Yeah, so have Sorry, go ahead Jen.

Participant 4: No, I was just going to say I think it will be repetitive, I think daily’s too much for some of these questions. So I agree with weekly or even like, I don't know, every two weeks or something, rotate the questions, something like that.

Interviewer: So depending on the question itself, it could be more of a long term reflection, rather than something that they automatically be filling out the same thing every single day. What other questions do you think would be useful to have on an app this for tracking? Because you mentioned the more mindful eating business, the what you're doing while you're eating, how you're eating? What else could be on it?

Participant 3: How do you feel about your eating?

Participant 6: Or prompts to remind people to listen to their hunger cues? Did you listen to your body? Did you stop eating when you're full or things that?

Interviewer: The more awareness of your hunger and fullness cues. So it kind of removes that guilt a little bit on what Participant 1 was saying. Maybe? Any other thoughts on that? OK, so head on to our third question. Or third section, will be about the features of the application. So first off, which instructions and supports should be provided to the users to help them use the application?

Participant 4: I think again links to already existing resources on explaining what food is considered or protein? What's a carb? What's a grain? What's a whatever, is Milk a - you know how to classify things?

Participant 1: Can I say something really luxurious. Probably having like, online live dietitian Q&A. I chat box just as that's what I am craving for when I – not about diet. But when I'm searching on other websites, and there are certain things I have no idea of and I really hope that I can chat with a expert on that topic, real time so that I can't get answers to my questions.

Interviewer: Yeah so getting a dietitian support would be amazing for sure. Yeah, being able to ask them your questions. Support from them. And then referring out to other resources where they could get information. Any other support methods or anything we should have users have on the app to be able to look to help them?

Participant 1: Like just a support group? Yeah, peer support group, where everybody can share their questions. But then there is a downside to that is that people can share non evidence based information on there. So if there is no dietician monitoring the form, then it can be another risk to peoples healthy. Yeah so it's positives and negatives to that.

Participant 6: Yeah, I think when you first open the app, I say they download it for first time, there should be a skippable tutorial, on how - OK, here are the three groups, Group A, Group B, Group C how they had a video showing us like, if that's embedded in the first part of the app. That will probably help them know how to use it better. Maybe even having a video or something that they can watch, showing how it's used. Like with the overview, or voiceover or something.

Interviewer: I guess what the video is a tutorial most apps kind of have that.

Participant 4: When they first start, because I mean, if you don't, then people are going to be very confused.

Interviewer: So what would be important to include in a tutorial that?

Participant 4: How to submit the food or how to submit the meal, the whole thing about you need to swipe up for this one and swipe down for that those kinds of things.

Interviewer: Anything else I could support users?

Participant 1: Examples of meals, common meals.

Participant 5: Maybe also some example of simple recipes.

Interviewer: Yeah. Recipes, cooking method, and headset as well. Getting then easy things to do and how to follow the guide in a simple and affordable way to. And which features do you think could help with adherence? So helping people stick with an application?

Participant 6: Reward? Some sort of rewards?

Interviewer: Whatever rewards could work on an app?

Participant 6: I don't know. I mean, I don't common ones now they have like, I don't know badges or congratulatory messages when he was certain plate. Like, good job. You did your first meal, you did your 100th meal. Congratulations. If it was partnered with a certain association, like the PC health ones, PC points.

Participant 1: Oh, yeah, I love that one. I use that. Or if you achieve your goal, or Yeah, if you feel yeah, I usually don't talk about goals. But yeah, so if you feel you've been doing pretty well for the past month, then you get a free, I don't know, a 30 minute dietitian consult. I've been having this kind of free idea to the dietitian. Since I don't that about I don't that either. So some yeah.

Participant 4: Just really going back to the other question. I think when you mentioned dietician, I think being able to share your results with if you have a healthcare provider or a dietitian or whoever, being able to share it with them. PDF or email, something really easy for them to send that.

Interviewer: As from the app itself directly to an RD or healthcare provider?

Participant 1: Yeah. But then the question is a lot of us general population probably are not aware of the fact that they have access to a dietician, to dietitian services, especially for those general population. They may not have a severe health condition then, yeah.

Interviewer: Yeah. For sure.

Participant 3: Because even their GP, if they had one or NP, could be helpful. I wonder, going back to goals, I could see starting at being overwhelming because of you're like, OK, I need to have more protein and have more vegetables and have less, like, maybe if there's preset goals, and you pick one per week or every two weeks or something. And then if your plate starts to match your goal, or your feedback of like, I ate more with other people, or I read food labels. It could send a message specifically about like, congratulations, you increased your vegetable intake, or something.

Interviewer: Yeah so maybe personalised, more smaller goals, so that people want to feel overwhelmed by trying to fix everything at once. Then congratulate them once they got there.

Participant 5: To add to what Participant 3 said, maybe if they reach their goal, you can try to challenge them with something new, for example, next week or next month, try to blah, blah, blah, or something that could - if they have challenged sometimes, it's more of a nice to have. But just a suggestion.

Interviewer: Yeah, a kind of –

Participant 3: Level them up.

Participant 5: Yeah, exactly. So every time you have something new to try. That makes me more excited.

Participant 4: Yeah, I guess. I know, like - I guess it depends also longevity of it. Because like, how much additional content do you have to develop? I know, apps headspace, for example. They have been doing kind of goals, but they actually make it kind of a free talk. So that person can set their own goal. And then the goal that they set kind of comes back later, hey, you set your goal this week, did you achieve it? Do you want to change it? That kind of thing. So I guess depends on like, what longevity you're aiming for. If you're aiming for them to use it for maybe a month or three months till they kind of know intuitively the pattern they're aiming for. Or if you're wanting them to use it, for life kind of thing.

Interviewer: So maybe you have options for them to put their own goals and personalise it based on what they want to do?

Participant 4: I think as an app developer, it'll make it easier for you because you don't have to come up with everything for them, right.

Participant 2: [unintelligible 01:02:33] you have like a menu of options. Were like, say, people you know pick a, whatever goal. And then that kind of guides them through some suggestions. You know, like, what's my goal? Helps you reach that goal.

Interviewer: So kind of, instead of having it be a free text completely, it'd be a little bit of a guided goal setting –

Participant 2: Well you [unintelligible 01:03:09].

Participant 3: But also let them skip things. I think that's part of with apps, if you don't want to fill it out, you should be able to just not, but too.

Interviewer: So make it as personable as possible so that people stick with it.

Participant 4: Because I can imagine someone's like, I know what my goal is, but they don't want to fill out what are my barriers? What are going to be my challenges? So being able to decide how much they want to put in?

Interviewer: Alright, so I'll move on from that question. So which features are required to ensure accessibility to this application for all users?

Participant 4: I guess having it translated into different languages.

Participant 2: Yeah that obviously. And maybe like also a desktop option for guys who have to go to the library to use the internet. Not that I think they would. But you know, [unintelligible 01:04:26] at least.

Participant 3: Or even older adults. My mom hate - I mean, she's not that old but she hates using her phone because the screen is so small she would rather look at it on a computer or –

Participant 6: iPad, yeah.

Participant 1: Yeah, that zoom in and zoom out features might be helpful to.

Participant 4: I think for ones like audio, I think it'll be hard if someone - because it's such a visual app. That's the only challenge I would see like, how do you make it something that someone who can't see it can use.

Interviewer: Yeah that definitely would be difficult.

Participant 6: But maybe if the writing is too small, if you can record it and play something for people who have difficulty to see it that could be an option. So like you can have – to press a button, play. If you don't want to read it, or I don't know.

Interviewer: Like the tutorials and examples and things that could all be voiceover, so they could listen to it.

Participant 6: So like, for example, if you having instructions, it's a long text and you don't want to read it. If you have an audio then you can just put your phone or whatever [unintelligible 01:06:04], iPod or anything, laptop, just on the side and just listen to it while doing something else.

Participant 1: I think to see that picture option, just because it's hard to record in real time for all the meals. So then at least you can go back and kind of look at it later and adjust your records.

Participant 3: To be able to adjust it is also a good idea.

Interviewer: Yeah, to go back and change it or be able to do it later. Alright, so now I'm going to go just to our as last few questions. So is there anything else that you thought about that could help users with an app that would mirror the Canada's Food Guide, for our discussion today?

Participant 1: Oh, make the record printable, so that they can print off whenever they can bring it to their registered dieticians or to their GP, yeah.

Interviewer: Yeah, that'd be super useful.

Participant 6: Yeah, a report a summarised report.

Participant 5: Maybe if a group of people are joining the app, if they can share their progress to the others, they can encourage people.

Interviewer: So more social aspect?

Participant 5: Yeah.

Participant 1: I, for this. The last question I always say, probably include that hunger scale, one to 10 that scale. I find that to be really helpful. Yeah, just keep them more self aware of how much they have eaten or how much they want to eat whenever they feel hungry or not. What reasons or their motivations of eating. Why did you eat this? Was it because it was, you were hungry? Or was it because you were craving for something sweet or was it because it was just lunchtime, you have to eat or something that or it's going out dining with friends. So

[End of recorded material]
