# Supplementary material for: Perceptions of self-monitoring dietary intake according to a plate-based approach: A qualitative study
Source: PLoS One. 2023 Nov 28;18(11):e0294652. doi: 10.1371/journal.pone.0294652 (PMC10683993; doi:10.1371/journal.pone.0294652)
Supplement: Attachment — Submitted filename: Response to Reviewers.docx [file pone.0294652.s006.docx]

**Journal requirements**

When submitting your revision, we need you to address these additional requirements.

**1.** Please ensure that your manuscript meets PLOS ONE's style requirements, including those for file naming. The PLOS ONE style templates can be found at

<https://journals.plos.org/plosone/s/file?id=wjVg/PLOSOne_formatting_sample_main_body.pdf> and

<https://journals.plos.org/plosone/s/file?id=ba62/PLOSOne_formatting_sample_title_authors_affiliations.pdf>

**Response:** *Thank you for supplying the templates, these changes have been made to the title page: Author titles removed (see lines 4-6), unnecessary information (full addresses) removed from affiliations (see lines 8-19), affiliations have also been reorganized to match preferred structure (also lines 8-19), and physical address removed from corresponding author section and the initial are added (see lines 20-21).*

*As for changes to the main body, they are: correction of all levels of heading to correct font size (see lines 23, 53, 63, 121, 122, 130, 154, 165, 179, 194, 221, 233, 257, 258, 278, 298, 299, 334, 354, 387, 459, 479, 491, 497, 504, 506, 511), removal of brackets on table citations (see lines 169, 238, 251), change of brackets from round to square on all intext citations (see lines across entire document for changes), and the removal of funding from acknowledgements (see line 492).*

**2.** We note that you have indicated that data from this study are available upon request. PLOS only allows data to be available upon request if there are legal or ethical restrictions on sharing data publicly. For more information on unacceptable data access restrictions, please see <http://journals.plos.org/plosone/s/data-availability#loc-unacceptable-data-access-restrictions>.

In your revised cover letter, please address the following prompts:

a) If there are ethical or legal restrictions on sharing a de-identified data set, please explain them in detail (e.g., data contain potentially sensitive information, data are owned by a third-party organization, etc.) and who has imposed them (e.g., an ethics committee). Please also provide contact information for a data access committee, ethics committee, or other institutional body to which data requests may be sent.

b) If there are no restrictions, please upload the minimal anonymized data set necessary to replicate your study findings as either Supporting Information files or to a stable, public repository and provide us with the relevant URLs, DOIs, or accession numbers. For a list of acceptable repositories, please see <http://journals.plos.org/plosone/s/data-availability#loc-recommended-repositories>.

We will update your Data Availability statement on your behalf to reflect the information you provide.

**Response:** *Thank you for your query. We have taken the necessary steps to make the transcripts of the focus groups available as Supporting Information files. Please find the transcripts as S4 Appendix. General public focus group transcripts and S5 Appendix. RD focus group transcripts in the supporting information list accompanying the manuscript. DOI will be available after acceptance.*

**3.** We note that Figure 1 in your submission contain copyrighted images. All PLOS content is published under the Creative Commons Attribution License (CC BY 4.0), which means that the manuscript, images, and Supporting Information files will be freely available online, and any third party is permitted to access, download, copy, distribute, and use these materials in any way, even commercially, with proper attribution. For more information, see our copyright guidelines: <http://journals.plos.org/plosone/s/licenses-and-copyright>.

We require you to either (1) present written permission from the copyright holder to publish these figures specifically under the CC BY 4.0 license, or (2) remove the figures from your submission:

1. You may seek permission from the original copyright holder of Figure 1 to publish the content specifically under the CC BY 4.0 license.

We recommend that you contact the original copyright holder with the Content Permission Form (<http://journals.plos.org/plosone/s/file?id=7c09/content-permission-form.pdf>) and the following text:

“I request permission for the open-access journal PLOS ONE to publish XXX under the Creative Commons Attribution License (CCAL) CC BY 4.0 (<http://creativecommons.org/licenses/by/4.0/>). Please be aware that this license allows unrestricted use and distribution, even commercially, by third parties. Please reply and provide explicit written permission to publish XXX under a CC BY license and complete the attached form.”

Please upload the completed Content Permission Form or other proof of granted permissions as an "Other" file with your submission.

In the figure caption of the copyrighted figure, please include the following text: “Reprinted from [ref] under a CC BY license, with permission from [name of publisher], original copyright [original copyright year].”

2. If you are unable to obtain permission from the original copyright holder to publish these figures under the CC BY 4.0 license or if the copyright holder’s requirements are incompatible with the CC BY 4.0 license, please either i) remove the figure or ii) supply a replacement figure that complies with the CC BY 4.0 license. Please check copyright information on all replacement figures and update the figure caption with source information. If applicable, please specify in the figure caption text when a figure is similar but not identical to the original image and is therefore for illustrative purposes only.

**Response:** *The figure has been removed from the submission as it holds minor relevance and was not necessary to get the sections point across. Lines 154-164 indicate changes in wording to reflect the removal of Figure 1 from the manuscript.*

**4.** Please include captions for your Supporting Information files at the end of your manuscript, and update any in-text citations to match accordingly. Please see our Supporting Information guidelines for more information: <http://journals.plos.org/plosone/s/supporting-information>.

**Response:** *Thank you for notifying us about this requirement. We have incorporated captions for the Supporting Information files at the end of our manuscript (Lines 692-698). Additionally, we've ensured that all in-text citations are now matched accordingly.*

**5.**Please review your reference list to ensure that it is complete and correct. If you have cited papers that have been retracted, please include the rationale for doing so in the manuscript text, or remove these references and replace them with relevant current references. Any changes to the reference list should be mentioned in the rebuttal letter that accompanies your revised manuscript. If you need to cite a retracted article, indicate the article’s retracted status in the References list and also include a citation and full reference for the retraction notice.

**Response:** *Thanks for bringing this to our attention. After carefully reviewing the reference list, we identified an issue related to the reference management software we were using, which inadvertently affected the accuracy of the references listed in the initial submission (references #17-21). We have now rectified this issue, and the reference list has been updated to ensure that all references are correct and up to date. All references are now listed according to the PLOS ONE requirements. More details are listed below:*

- *All journal name abbreviations have been included and the reference style is according to the Vancouver style.*
- *For all in-text citations, the reference number is now in square brackets.*
- *Removed references that were not cited in the text (#19, 20, 21) and replaced with the correct references.*
- *References #17,18 are now replaced with the correct references.*
- *Reference #52 initially located inaccurately, is now correctly attributed to its respective sources within the reference list as #19.*
- *Removed reference #27 as I could not find it in journal databases (probably a retracted paper)*
- *We have recently published a paper on the paper-based version of the plate method which we cited in the text and included in the reference list (#32).*
- *Based on the comments by reviewers to emphasize the potential applicability of our research findings to a diverse range of multicultural and ethnic populations, particularly within the context of Canada's increasing immigrant population we added another paper to the text and included it in the reference list (#59).*

**Additional Editor Comments:**

Reviewer 1 comment:
The authors should highlight more on how you one can extrapolate the result to many multi-cultural & ethnic populations which is increasing in Canada day by day due to influx of immigrants. In addition, this would have increased the usefulness of this app in other countries as well.

[Note: HTML markup is below. Please do not edit.]

Reviewers' comments:

Responses to Questions

3. Have the authors made all data underlying the findings in their manuscript fully available?

The [PLOS Data policy](http://www.plosone.org/static/policies.action#sharing) requires authors to make all data underlying the findings described in their manuscript fully available without restriction, with rare exception (please refer to the Data Availability Statement in the manuscript PDF file). The data should be provided as part of the manuscript or its supporting information, or deposited to a public repository. For example, in addition to summary statistics, the data points behind means, medians and variance measures should be available. If there are restrictions on publicly sharing data—e.g. participant privacy or use of data from a third party—those must be specified.

Reviewer #1: No

Reviewer #2: No

**Response:** Thank you for the comment. We have made our data (including all focus group transcripts) available now.

5. Review Comments to the Author

**Reviewer #1:**  **The results are encouraging, however more quotes from both the dietitians and the general public could have added the qualitative strength to this article.**

**Response:** Thank you for the comment. We have added additional quotes to the paper.

**Reviewer #1:**  **Given that the aim of the study was also to explore the efficacy of the app among all major ethnic groups within Canada. I think the authors should highlight more on how you one can extrapolate the result to many multi-cultural & ethnic population which is increasing in Canada day by day due to influx of immigrants. In addition this would have increased the usefulness of this app in other countries as well.**

**Response:** *Thank you for your feedback. We have addressed your suggestion by including the following sentence in the manuscript (reference #59): "In the Canadian context, up to 40% of the population will be represented by visible minorities by 2036 [59]; therefore highlighting the significance of incorporating cultural considerations in the future versions of iCANPlate^TM^." This addition underscores the growing multicultural and ethnic diversity in Canada and the importance of considering these factors in future iterations of the iCANPlate^TM^ app, making it more applicable not only in Canada but also in other countries with similar demographic trends.*

**Reviewer #2: Review of ‘Perceptions of self-monitoring dietary intake according to a plate-based approach: A qualitative study’**
I have reviewed the research paper ‘Perceptions of self-monitoring dietary intake according to a plate-based approach’. First off: maybe I missed this information, but why are certain sentences blacked out? Why aren’t reviewers allowed to see who gave the ethical approval (line 231), for instance? I cannot sign off the ethical approval for this reason (again, for instance).

**Response:**
*Thank you for reviewing our paper. We appreciate your feedback and apologize for any confusion regarding the blacked-out information. To avoid any type of biases and to adhere to ethical guidelines, we initially redacted certain identifiable information in the manuscript. However, in response to your concern and to ensure transparency, we have now made this information visible. You will find the necessary details regarding the ethical approval on line 224-225 and any other relevant information. We hope this addresses your concern, and we are grateful for your attention to this matter.*
